# Expression of the Excitatory Postsynaptic Scaffolding Protein, Shank3, in Human Brain: Effect of Age and Alzheimer’s Disease

**DOI:** 10.3389/fnagi.2021.717263

**Published:** 2021-08-24

**Authors:** Lily Wan, Jia-Qi Ai, Chen Yang, Juan Jiang, Qi-Lei Zhang, Zhao-Hui Luo, Rou-Jie Huang, Tian Tu, Aihua Pan, Ewen Tu, Jim Manavis, Bo Xiao, Xiao-Xin Yan

**Affiliations:** ^1^Department of Neurology, Xiangya Hospital, Central South University, Changsha, China; ^2^Department of Anatomy and Neurobiology, Central South University Xiangya School of Medicine, Changsha, China; ^3^Medical Doctor Program, Xiangya School of Medicine, Central South University, Changsha, China; ^4^Department of Neurology, Brain Hospital of Hunan Province, Changsha, China; ^5^Faculty of Health and Medical Sciences, The University of Adelaide, Adelaide, SA, Australia

**Keywords:** brain aging, cognitive decline, developmental brain disorders, excitatory neurotransmission, neuropsychiatric diseases, neurodegenerative disorders

## Abstract

Shank3 is a postsynaptic scaffolding protein of excitatory synapses. Mutations or variations of *SHANK3* are associated with various psychiatric and neurological disorders. We set to determine its normal expression pattern in the human brain, and its change, if any, with age and Alzheimer’s disease (AD)-type β-amyloid (Aβ) and Tau pathogenesis. In general, Shank3 immunoreactivity (IR) exhibited largely a neuropil pattern with differential laminar/regional distribution across brain regions. In youth and adults, subsets of pyramidal/multipolar neurons in the cerebrum, striatum, and thalamus showed moderate IR, while some large-sized neurons in the brainstem and the granule cells in the cerebellar cortex exhibited light IR. In double immunofluorescence, Shank3 IR occurred at the sublemmal regions in neuronal somata and large dendrites, apposing to synaptophysin-labeled presynaptic terminals. In aged cases, immunolabeled neuronal somata were reduced, with disrupted neuropil labeling seen in the molecular layer of the dentate gyrus in AD cases. In immunoblot, levels of Shank3 protein were positively correlated with that of the postsynaptic density protein 95 (PSD95) among different brain regions. Levels of Shank3, PSD95, and synaptophysin immunoblotted in the prefrontal, precentral, and cerebellar cortical lysates were reduced in the aged and AD relative to youth and adult groups. Taken together, the differential Shank3 expression among brain structures/regions indicates the varied local density of the excitatory synapses. The enriched Shank3 expression in the forebrain subregions appears inconsistent with a role of this protein in the modulation of high cognitive functions. The decline of its expression in aged and AD brains may relate to the degeneration of excitatory synapses.

## Introduction

The Shank family proteins, Shank1, 2, and 3, serve as the core apparatus of postsynaptic protein network (Monteiro and Feng, 2017; Mossa et al., 2018). These scaffolding proteins regulate the targeting, anchoring, and functioning of neurotransmitter receptors and other signaling molecules, especially at excitatory synapses, thereby playing critical neurobiological roles in synapse formation, plasticity, and transmission (MacGillavry et al., 2016; Wang et al., 2017; Bey et al., 2018; Guo et al., 2019). Genome-wide association studies (GWAS) have extended strong evidence that alterations in Shank family genes are involved in the development of various neuropsychiatric disorders (Zhang et al., 2015; Alexandrov et al., 2017; Drapeau et al., 2018; Kanani et al., 2018; Zhao et al., 2019; Tatavarty et al., 2020). The *SHANK3* gene appears to be the most studied among its family members in the context of the link with the risk of neuropsychiatric disorders. Thus, *SHANK3* abnormalities may be related to ∼0.5% of autism spectrum disorders (Kolevzon et al., 2011; Bey et al., 2018; Jaramillo et al., 2020), which include deletion (Bonaglia et al., 2011; Boccuto et al., 2013; Soorya et al., 2013; Chen et al., 2017; Drapeau et al., 2018), point mutation (Durand et al., 2007; Moessner et al., 2007; Boccuto et al., 2013; Soorya et al., 2013; Cochoy et al., 2015), insertion (Bonaglia et al., 2011; Jaramillo et al., 2017), chromosome translocation (Bonaglia et al., 2001*;* Moessner et al., 2007), microduplication (Okamoto et al., 2007; Chen et al., 2017), and frameshift (Kanani et al., 2018). Multiple forms of *SHANK3* mutation are also found in individuals with Phelan-McDermid Syndrome Contribution of *SHANK3* mutations to autism spectrum disorder (Watt et al., 1985; Bonaglia et al., 2006, 2011; Toro et al., 2010; Phelan and McDermid, 2012; Zwanenburg et al., 2016), schizophrenia (Failla et al., 2007; Gauthier et al., 2010; de Sena Cortabitarte et al., 2017), and bipolar disorder (Denayer et al., 2012; Vucurovic et al., 2012; Crisafulli et al., 2013; Ortiz et al., 2015). *SHANK3* gene is located on chromosome 22q13.3 in humans, spans ∼55.1 kb in length, and contains 24 exons, six alternative promoters, and one alternative stop codon located in the exon 21b. Besides alternative promoter and mRNA splicing, the gene expression is regulated by epigenetic mechanisms, resulting in tissue specific localization of its mRNA and protein (Wan et al., 2021). Previous studies in rodents have shown that Shank3 mRNA expression appears to be high in the brain, heart, and spleen (Lim et al., 1999). In the rat brain, Shank3 mRNA expression is particularly enriched in the hippocampal formation, thalamus, striatum, and cerebellar granule cells (Peça et al., 2011; Monteiro and Feng, 2017).

Little is currently known about the regional and laminar distribution of Shank3 protein in the human brain. It is also unclear if its expression might change during brain aging and in neurodegenerative diseases. In the present study, we set to characterize the neuroanatomical distribution pattern of Shank3 protein expression in the human brain. Immunohistochemical examination was carried out, using postmortem brains from donors with a wide age range [12–95 years-old, *n* = 28], with densitometric analysis in selected cerebral and cerebellar regions/lamina. We also determined a differential regional distribution pattern of Shank3 immunoreactivity (IR) in the striatum relative to that of some general [synaptophysin and postsynaptic density-95 (PSD95)] and specific (glutamatergic, GABAergic, and cholinergic) pre- and postsynaptic markers. In addition, we explored age-related alteration in Shank3 expression relative to the general pre- and postsynaptic markers synaptophysin and PSD95, by a western blot, using tissue lysates from representative cerebral and cerebellar cortical regions in three age groups and a pathologically verified AD group.

## Materials and Methods

### Postmortem Human Brain Samples

Postmortem human brains were banked through the willed body donation program established at Xiangya School of Medicine for medical education, following a standard operation protocol recommended by the China Human Brain Banking Consortium (Yan et al., 2015; Qiu et al., 2019). Informed consent for whole body donation was obtained from the donors or next of kin of the subjects in compliance with the body/organ donation regulations set by the Chinese government. The use of postmortem human brains was approved by the Institutional Committee for Research and Education, in compliance with the code of ethics of the World Medical Association (Declaration of Helsinki).

Brain samples used in the current study were from donors who died mostly of clinically diagnosed “non-neurological” diseases, with the demographic information of the cases (age at death, sex, clinical diagnosis of disease, cause of death, and postmortem delay of brain collection) and the grouping of the cases detailed in Table 1. The cases used for immunohistochemical and/or for immunoblotting analyses are also noted. All brains from donors aged ≥ 30 years were routinely assessed for β-amyloid (Aβ) and phosphorylated tau (p-Tau) pathologies in paraffin and/or cryostat sections prepared from the formalin-fixed hemibrain, including the temporal, parietal, frontal and occipital cortices and subcortical structures (Qiu et al., 2019). As a result, the brains from the elderly cases (≥ 65 years) were commonly found to show a certain degree of Alzheimer-type Aβ and neurofibrillary tangle (NFT) pathologies according to the Thal Aβ and Braak NFT/pTau staging guidelines (Braak and Braak, 1991; Thal et al., 2002; Braak et al., 2006; Montine et al., 2012; Table 1). A substantial number of the brains from the aged cases exhibited features of the so-called primary age-related tauopathy (PART), with Braak pTau stages I-IV in the absence of Aβ deposition (Shi et al., 2020). The neuropathological characterization methodology was included in our recent studies (Xu et al., 2019; Shi et al., 2020; Tu et al., 2020), therefore it is not detailed here (to avoid redundancy).

**TABLE 1:** Demographic information of brain donors and staging of Alzheimer-type neuropathoplogy.

Case	Group	Age (y)	Sex	Clinical diagnosis or cause of death	Postmortem delay (h)	Braak NFT stage	Thal Aβ phase	Tissue usage
1	Youth	12	F	Thalassemia	12	N/A*	N/A	IHC**
2	Youth	14	M	Leukemia	9	N/A	N/A	IHC/WB**
3	Youth	14	F	Tuberculosis	10	N/A	N/A	IHC
4	Youth	16	F	Transposition of the great arteries	8	N/A	N/A	IHC
5	Youth	19	M	Infection/sepsis	11	N/A	N/A	IHC/WB**
6	Youth	19	M	Cardiopulmonary failure	5	N/A	N/A	IHC/WB**
7	Youth	22	F	Lupus erythematosus	12	N/A	N/A	IHC/WB**
8	Adult	31	F	Vagina cancer	10	0	0	IHC
9	Adult	38	M	Liver cancer	8	0	0	IHC/WB**
10	Adult	49	M	Lung cancer	10	0	0	IHC/WB**
11	Adult	50	M	Lung cancer	7	0	0	IHC/IF/WB**
12	Adult	50	F	Myocardial infarction	12	0	0	IHC/WB**
13	Adult	55	M	Liver cancer	8	0	0	IHC**/IF
14	Aged	68	M	Renal failure	8	II	0	IHC/WB**
15	Aged	70	F	Pneumonia	12	II	0	IHC
16	Aged/AD***	74	M	Multisystem failure, demented	5	V	5	IHC**
17	Aged	76	M	Heart stroke	7	0	0	IHC/IF
18	Aged	77	M	Multisystem failure	12	III	0	IHC/WB**
19	Aged	80	M	Iron-deficiency anemia	7	III	0	IHC/WB**
20	Aged	80	M	Lung cancer	15	III	1	IHC
21	Aged	81	F	Chronic renal failure	7	IV	0	IHC**
22	Aged	81	M	Cardiovascular failure	6	II	0	IHC/WB**
23	Aged/AD	83	F	Multisystem failure, demented	12	III	4	IHC/WB**
24	Aged/AD	85	M	Astrocytoma, demented	12	III	4	IHC**
25	Aged	86	M	COPD****	6	III	0	IHC/WB**
26	Aged/AD	87	F	Coronary heart disease, demented	6	VI	5	IHC/WB**
27	Aged/AD	88	F	Heart failure, demented	9	V	4	IHC/WB**
28	Aged	95	M	Multisystem failure	14	III	0	IHC

*AD-type neuropathology not assessed; **Used for assessing regional and age-related change in Shank3 expression; ***Cases met the criteria for neuropathological diagnosis of AD (Braak and Braak, 1991; Thal et al., 2002; Braak et al., 2006; Montine et al., 2012); ****chronic obstructive pulmonary disease.

*IHC, immunohistochemistry; IF, immunofluorescence; WB, western blot.*

### Tissue Processing

Each brain was bisected along the cerebral sagittal fissure immediately following brain removal. One hemi-brain (left side, or opposite to the handedness if known) was then immersed in phosphate-buffered formalin (pH7.2) for histological studies. The other hemi-brain was cut into frontal slices at ∼1-cm thickness, fresh-frozen with dry ice, and then stored at −80°C for future biochemical studies. After formalin immersion for 2–4 weeks, the fixed half brain was sliced frontally at 1-cm thickness. The cryostat sections were primarily used in the current study because the regional and laminar pattern of synaptic markers were typically neuropil-like, which were better illustrated in relatively thicker sections (than thin-cut paraffin sections). The olfactory bulb and tissue blocks containing the frontal, parietal, and occipital neocortices, striatum, thalamus, cerebellar cortex, and deep nuclei, midbrain, pons, and medulla oblongata were cryoprotected in 30% sucrose in phosphate-buffered saline (PBS, 0.01M, pH7.2). The frozen sections at 40-μm thickness were cut in a cryostat and collected in culture plates in PBS, and then stored in a cryoprotectant at −20°C before use for immunolabeling. Adjacent sections from the same block were collected alternatively into 24 sets of wells of cell/tissue culture plates. This allowed immunohistochemical processing of multiple sets of adjacent sections from neighboring wells with different antibodies or conditions to facilitate comparative microscopical analysis.

### Immunohistochemistry and Immunofluorescence

Sections from multiple regions of the same brain and three to four cases were processed in parallel in each experiment. The sections were first treated free-floatingly in PBS, containing 5% H_2_O_2_ for 30 min, and in PBS containing 5% normal horse serum, with.3% Triton X-100 for 1 h, to minimize non-specific labeling. Sections were subsequently incubated in PBS containing mouse anti-Shank3 (Santa Cruz Biotechnology, Inc, sc-377088, diluted at 1:1,000) at 4°C overnight. After three washes with PBS, the sections were reacted with biotinylated pan-specific secondary antibody (horse anti-mouse, rabbit, and goat IgGs) at 1:400 for 1 h and avidin-biotin complex (ABC) reagents (1:400) (Vector Laboratories, Burlingame, CA, United States) for another hour. Immunoreactive product was visualized with 0.003% H_2_O_2_ and.05% 3,3′-diaminobenzidine (DAB). Sections passing the striatum and thalamus from four brains were also selected for a comparative assessment of Shank3 IR relative to that of synaptophysin, PSD95, vesicular glutamate transport-2 (VGLUT2), glutamate decarboxylase-67 (GAD67), and choline acetyltransferase (ChAT). Thus, one set of sections was processed as above for Shank3 IR. Additional sets of sections were processed with rabbit anti-synaptophysin (ProteoTech, Inc., #17785-1-AP, 1:2,000), rabbit anti-PSD95 (Cell Signaling Technology, #3450S, 1:1,000), mouse anti-VGLUT1 (Merck-Millipore, MAB5502, 1:2,000), mouse anti-GAD67 (Merck-Millipore, AB1511, 1:2,000), and goat anti-ChAT (Merck-Millipore, AB1447, 1:2,000), respectively. The immunolabeled sections were mounted on glass microslides, dehydrated with ascending ethanol, cleared with xylene, and coverslippered with a mounting medium. One section per brain block/region was also counterstained with hematoxylin for histological orientation in microscopical examination.

Double immunofluorescence was carried out, using temporal sections at the mid-hippocampal levels from two adult and aged cases (Table 1). Sections were pretreated in PBS, containing 5% donkey serum for 60 min, and then incubated overnight at 4°C with the mouse anti-Shank antibody (1:100), together with the rabbit anti-synaptophysin antibody (ProteoTech, Inc., #17785-1-AP, 1:1,000) or the rabbit anti-PSD95 antibody (Cell Signaling Technology, 1:500). The sections were further reacted with Alexa Fluor^®^ 488-conjugated donkey anti-rabbit and Alexa Fluor^®^ 594 conjugated donkey anti-mouse IgGs (1:100, Invitrogen, Carlsbad, CA, United States). Lastly, the sections were briefly stained with nuclear dye DAPI, treated with 0.1% Sudan black to block autofluorescence, washed with PBS, and mounted with an anti-fading glycerol medium.

### Western Blot

Tissue blocks were sampled from the frozen slices of the available cases with the shortest postmortem delays in the groups for immunoblotting (Table 1). The sampling sites included the CA1 area of the mid-hippocampus, the anterior part of the cingulate gyrus, the anterior part of the superior frontal gyrus, the middle part of the precentral gyrus, the middle part of the postcentral gyrus, the primary visual cortex, the mid-level of pons (a cross slice), the oblongata medulla (a cross) slice at the level of the inferior olive, and cerebellar cortex.

Samples were weighed and homogenized in 10 × (w/v) radioimmuno precipitation (RIPA) buffer, containing a cocktail of proteinase inhibitors (Cat#P0013B, Beyotime, China). The resulting lysates were centrifuged at 12,000 rpm for 15 min at 4°C, with the supernatants collected and assayed for protein concentration (Bio-Rad Laboratories, Hercules, CA, United States). Extracts containing an estimated equal amount of total protein were run in 10% SDS-PAGE gels in the same experiment. Separated proteins were electrotransferred onto Trans-Blot pure nitrocellulose membranes (Bio-Rad Laboratories), and then blotted with mouse anti-Shank3 (1:400), rabbit anti-PSD95 (Cell Signaling Technology, 1:1,000), rabbit anti-synaptophysin (ProteoTech, Inc., 1:7,500), and mouse anti-β-actin (ProteoTech, Inc., 1:5,000) as loading controls. The membranes were further reacted with horseradish peroxidase (HRP)-conjugated goat anti-mouse IgG (ProteoTech, Inc., #SA00001-1, 1:5,000) or goat anti-rabbit IgG (ProteoTech, Inc., #SA00001-2, 1:6,000). Membranes were further reacted briefly with the Pierce^TM^ ECL-Plus Western Blotting Substrate detection kit (Thermo Fisher Scientific, #32132), followed by X-ray film exposure or imaging with the UVP ChemStudio/PLUS device (Analytik-Jena/UVP, United States).

### Validation of the Specificity of Shank3 Antibody

To verify the specificity of the Shank3 antibody in immunohistochemistry, an antibody omission test was carried out by using adjacent cerebral cortical sections processed identically except that the Shank3 antibody was omitted in the incubation buffer in the set of sections served as negative assay control. We further validated specificity of the Shank3 antibody by immunoblotting, using a virus-driving *in vivo* overexpression approach. Thus, a plasmid containing human Shank3 gene was constructed, using the adenovirus as a vehicle, which was injected into the rat hippocampus, resulting in neuronal transinfection and overexpression of Shank3 protein *in vivo*. The transinfected rat hippocampi and vehicle-injected controls were harvested, followed by western blot validation, using these rat samples along with frozen human cortical samples.

### Imaging, Quantitative Analysis, and Figure Preparation

Immunolabeled sections were examined initially on an Olympus BX51 microscope (CellSens Standard, Olympus Corporation, Japan) for a validation of immunolabeling quality. For each region/structure of a given brain, sections representing the characteristic regional and cellular pattern of Shank3 IR were scan-imaged, using the 20 × objective on a Motic-Olympus microscope equipped with an automated stage and imaging system (Wuhan, Hubei, China), which could yield a final autofocused, montaged, and magnification-adjustable image, covering the entire area of a glass slide. Low (1×) and high (10× and 20×) magnification images over the area of interest were then extracted from the montaged images for figure illustration. Double immunofluorescent sections were examined on a Nikon confocal microscope equipped with a digital imaging system (Nikon-EZ-C1). Optimal double immunofluorescent labeling was obtained for Shank3 and synaptophysin co-labeling during imaging analysis (EZ-C1 3.70 Free Viewer), with representative data presented in the current study.

For densitometric analyses, micrographs were also extracted from selected Motic images at 4× magnification over the gyral portions of the basal prefrontal, postcentral, and temporal neocortical areas, the molecular layer of the dentate gyrus, and the cerebellar cortex. For each of the above regions, both the gray matter (e.g., layers I-VI for the cerebral cortical regions) and the white matter were included. The images from each brain case were placed together without any photographic editing, and then saving as a gray scale TIFF file, using Photoshop 10.1. These files were used for measurement of the optic densities (o.d., expressed as digital light units per square millimeter, DLU/mm^2^) across the cortical layers or an anatomically defined layer (e.g., the molecular layer), using the OptiQuant software (Packard Instrument Co., Meriden, CT, United States) as established previously (He et al., 2014; Hu et al., 2017), which yielded the total o.d. in a given area of interest. The o.d. in the white matter (WM) within the same image was also obtained and used as the cutoff level to calculate the specific o.d. in the gray matter areas. The densitometric methods applied to the individual brain regions will be illustrated in more detail in the result section. Quantification of immunoblotted protein bands was carried out, using the National Institute of Health (NIH) Image-J software. Optic densities (o.d.) of Shank3 protein and synaptic markers (PSD9.5, synaptophysin) were normalized to the internal loading control (β-actin), expressed as the o.d. ratio to β-actin.

Statistical analysis was carried out to compare means between groups, using one-way ANOVA, together with Bonferroni’s multiple comparison *post hoc* test. Pearson correlation was also used to test the possibility of parallelism between the levels of Shank3 and PSD9.5 among brain regions (GraphPad Prism 5, San Diego, CA, United States). Figure panels were assembled, using Photoshop 7.1.

## Results

**FIGURE 1:**
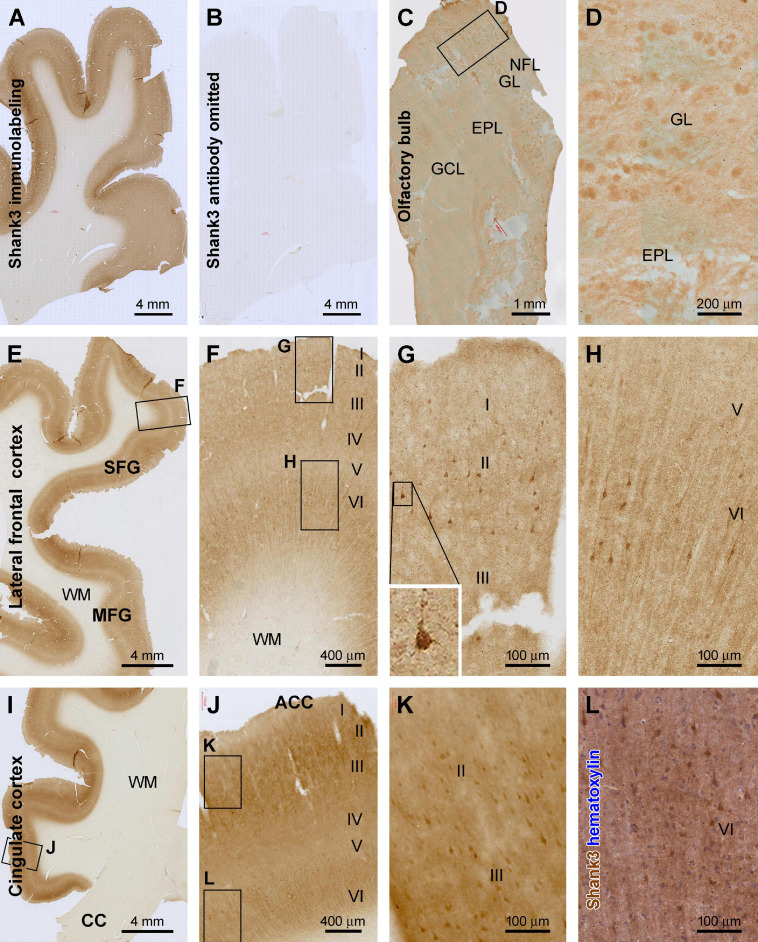
Characterization of Shank3 immunoreactivity (IR) in the olfactory bulb (OB) and frontal lobe cortex. Panels **(A,B)** are images of medial frontal cortex from adjacent sections processed identically except for the omission of the primary antibody in the latter, which resulted in the lack of IR across the entire section **(B)**. **(C)** shows light diffuse IR across the OB, with the framed area enlarged as **(D)**, illustrating the labeling in individual glomeruli. **(E–H)** are low and high-power views of the labeling in the lateral frontal cortex, and **(I–L)** show IR in the anterior cingulate neocortex (ACC). Shank3 IR exhibits largely a neuropil-type pattern with the intensity noticeable higher in layers II–IV and VI than I and V. An apparently subset of pyramidal and multipolar neurons shows greater IR than the neuropil, which can be better recognized in the immunolabeled section with hematoxylin counterstain **(L)**. Additional abbreviations: CC, corpus collosum; NFL, nerve fiber layer; GL, glomerular layer; EPL, external plexiform layer; GCL, granule cell layer; WM, white matter; I–VI, cortical layers. Scale bars and panel orientation are as indicated in the panels.

We observed Shank3 immunoreactivity (IR) in sections from all brains, with no specific IR present in the sections processed by omitting the primary antibody (Figures 1A,B). Overall, Shank3 IR exhibited predominantly a neuropil-like distribution pattern in the brain, which was evident in the gray matter and absent in the white matter. The regional and laminar pattern appeared differential across neuroanatomical structure/regions and was largely similar in all age groups. However, age-related changes in the neuropil and neuronal somata labeling were detectable microscopically in the aged/AD relative to youth/adult cases. In the following result sections, we first describe the “basic” regional, laminar, and cellular patterns of Shank3 IR, using representative images obtained from the youth and adult cases (#4, #6, #9, and #11 in Table 1). We further show examples of the altered laminar and cellular pattern of Shank3 IR observed in representative brain regions in aged and AD cases. Quantitative immunoblot data are presented in support of the immunohistochemical observations, especially regarding the differential regional expression of Shank3 and the decline of its expression with age. Neuroanatomical terms were adapted by referring to published human brain atlases (Brodmann, 1909; Mai et al., 2008; Ding et al., 2017).

### Distribution of Shank3 IR in Major Human Brain Structures

#### Olfactory Bulb

In the olfactory bulb (OB), Shank3 IR was largely present in the outer layers in longitudinally cut sections, covering the bulb and olfactory tract (OT) at low magnification (Figure 1C). Light to moderate IR was seen in the granule cell layer (GCL) and the external plexiform layer (EPL), appearing largely in a diffuse neuropil-like distribution pattern. Notably, relatively high IR was present in the glomerular layer (GL). Thus, individual glomeruli were visualized against the interglomerular areas with lighter staining. Overall, no apparently labeled neuronal perikarya or processes were observed across the bulbar layers. Also, no axonal profiles were labeled in the nerve fiber layer (NFL) (Figures 1C,D).

#### Cerebral Neocortex

**FIGURE 2:**
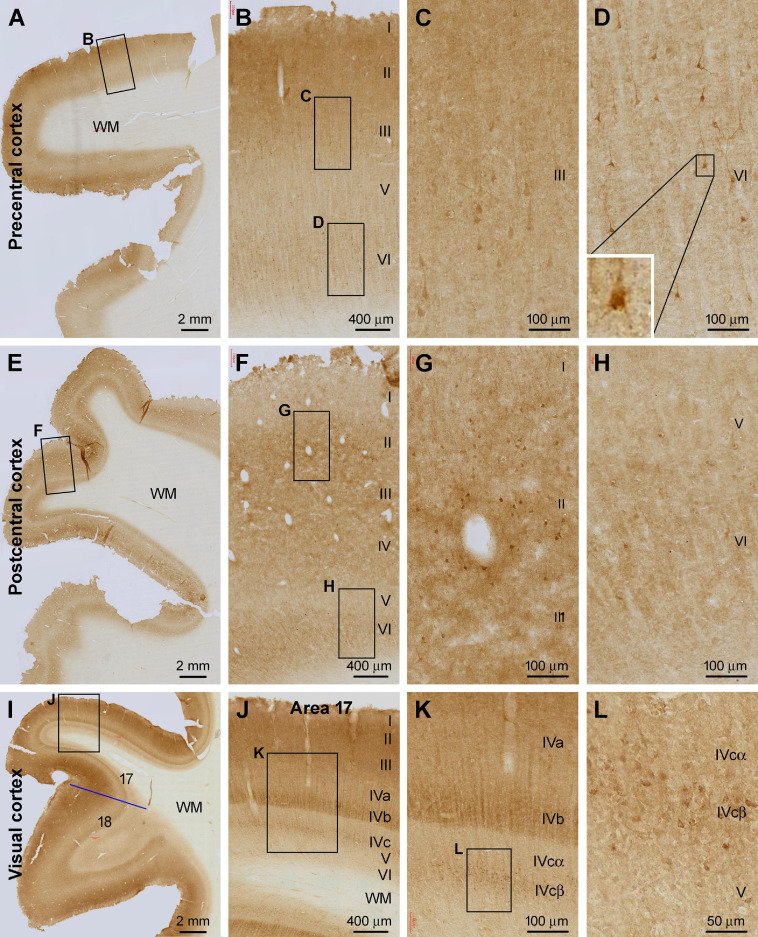
Shank3 IR in the primary motor, somatosensory, and visual cortical areas. **(A)** shows low power view of the IR in the precentral cortex, with framed areas enlarged as **(B–D)**. **(E)** shows low magnification view of the IR in the postcentral cortex, with framed areas enlarged as **(F–H)**. **(I–L)** illustrate the IR in the primary (area 17) and secondary (area 18) visual cortical centers. The sublayers (IVa, IVb, IVcα, and IVcβ) of IV in area 17 exhibit differential intensity. A small group of neuronal somata, along with their apical and basal dendrites, shows moderate to strong IR; most of which appear pyramidal in shape. Scale bars are as indicated in the panels.

**FIGURE 3:**
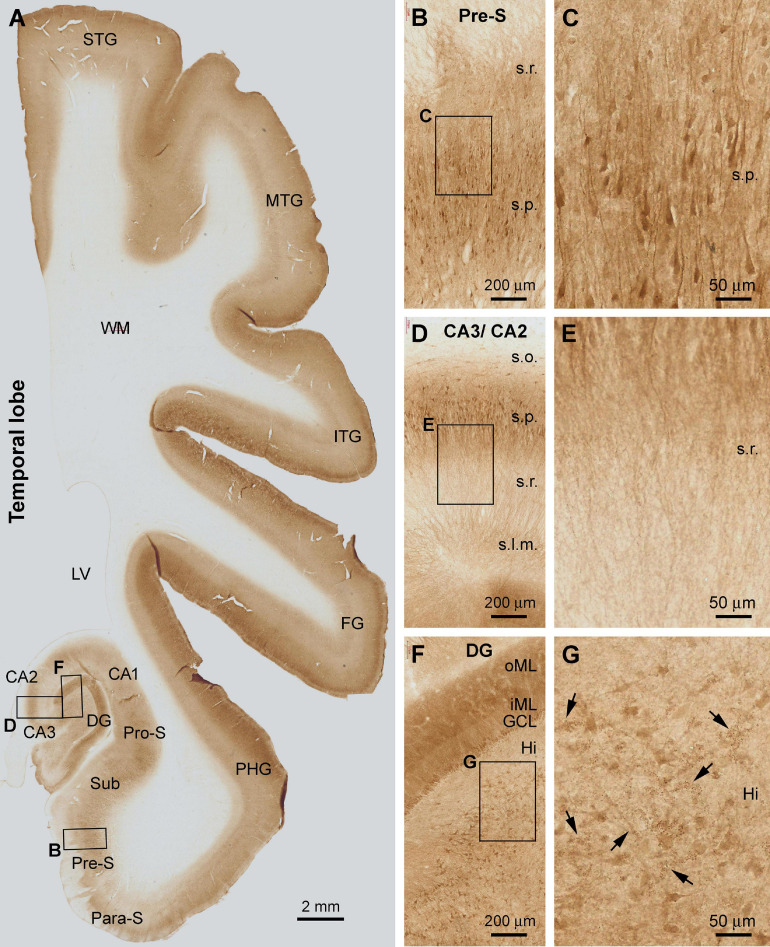
Shank3 IR in the temporal neocortex, entorhinal cortex, and hippocampal formation. The pattern seen in the neocortex and transitional entorhinal cortex is comparable to that in the frontal associative neocortex shown in Figure 1. In the subicular subregions **(A–C)** and the Ammon’s horn CA1-3 sectors **(A,D,E)**, Shank3 IR is present in the neuropil as well as the somata and dendritic processes of the pyramidal neurons over the stratum radiatum (s.r.) and stratum pyramidale (s.p.). The distal parts of apical dendritic processes extend into the stratum lacunosum-moleculare (s.l.m.), whereas little labeling is seen in the stratum oriens (s.o.) (D, E). In the dentate gyrus (DG), the molecular layer (ML) and the granule cell layer (GCL) are strongly labeled. The inner ML (iML) appears lighter than the outer ML (oML) **(F)**. In the hilus (Hi), mossy cells and their dendrites are moderately labeled, as are some cluster profiles likely representing the thorny excrescences (**G**, pointed by arrows). STG, superior temporal gyrus; MTG, middle temporal gyrus; ITG, inferior temporal gyrus; FG, fusiform gyrus; PHG, parahippocampal gyrus; Para-S, parasubiculum; Pre-S, subiculum; Sub, subiculum; Pro-S, prosubiculum; LV, lateral ventricle; WM, white matter. Scale bars are as indicated.

**FIGURE 4:**
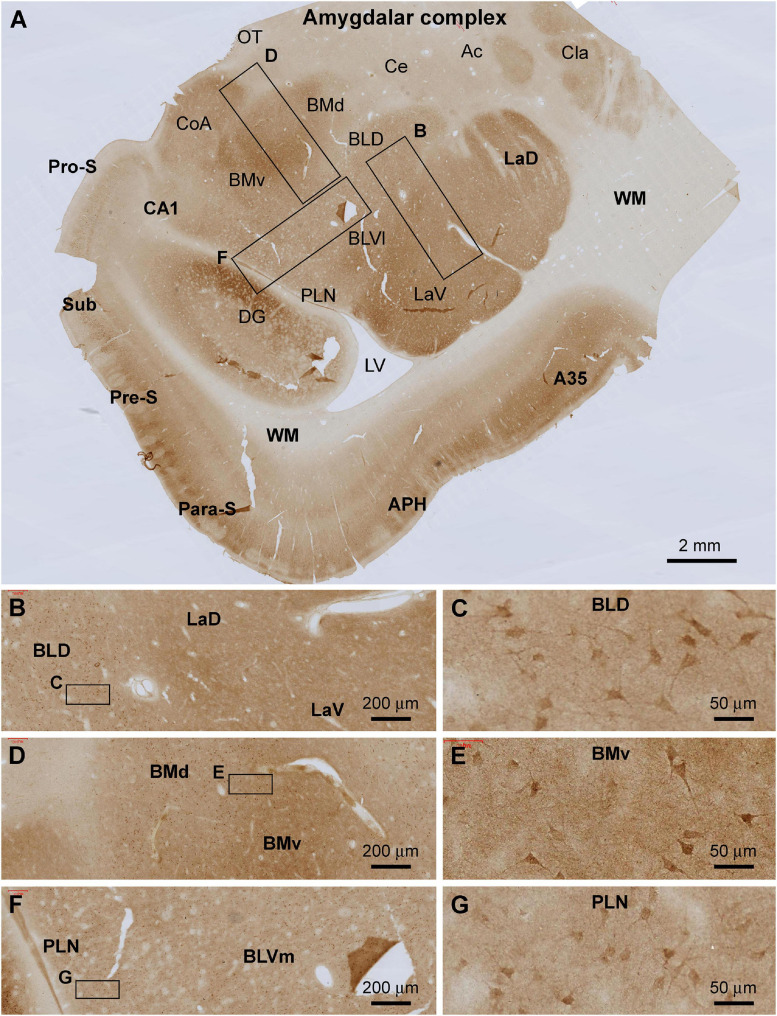
Localization of Shank3 IR in the amygdalar complex. Panel **(A)** is a low magnification image of temporal lobe section at the level between the amygdala and hippocampus, with framed areas enlarged as **(B–G)**. Shank3 IR occurs in all amygdalar subregions with variable labeling intensity. Thus, stronger IR is present in the lateral nuclei group, including both the dorsal and ventral subdivisions (LaD, LaV), and the basomedial group including the dorsal and ventral subdivisions (BMd, BMv). At high magnifications, the IR exhibits a spreading neuropil pattern but also occurs in relatively large-sized perikarya in multipolar shape, including the proximal dendrites **(B–G)**. Ac, anterior commission; A35, area 35; Ce, central nucleus; CoA, anterior cortical nucleus; Cla, claustrum; BLD, basolateral nucleus dorsal division; BLVl, basolateral nucleus lateral division; OT, optic tract; PLN, paralaminar nucleus; others are as defined in Figure 3. Scale bars are as indicated.

Shank3 IR exhibited a similar distribution pattern in all the neocortical regions of the cerebrum (Figures 1–4). Overall, the IR was largely neuropil-like and restricted to the gray matter, with the intensity in the white matter (WM) comparable to the background (Figures 1A,B). In the majority of the neocortical regions, the labeling intensity was relatively higher in layers II–IV and VI than in layers I and V. A small number of pyramidal-like neurons showed otherwise heavier IR than the intensity of the neuropil of the same layer (Figures 1–4). The laminar pattern and labeled neuronal profiles seen in representative cerebral cortical areas are described below.

In the associative frontal neocortical areas, including the superior and middle frontal gyri (SFG, MFG) (Figures 1E–H) and the anterior cingulate gyrus (ACC) (Figures 1I–L), layers II–III and VI showed the highest intensity across the gray matter, while layer IV and the lower part of layer V were lightly stained (Figures 1F,J). In the above regions, some neuronal somata, along with their proximal dendrites, exhibited fairly strong and distinct IR against the moderately labeled neuropil background. These cells were mostly located in layers II/III and VI, relatively large in size and typically pyramidal or multipolar in shape. Therefore, based on their morphological appearance, they could be apparently recognized as cortical principal neurons (Figures 1F–H,J–L). It should be noted that the labeled neurons would represent a subpopulation, most likely a small fraction, of the cortical principal neurons (Figures 1G,H,K,L), which could be realized in immunolabeled sections with hematoxylin counterstain (Figure 1L).

In the primary motor cortical center (Brodmann areas 4 and 6) (Figures 2A–D) and primary somatosensory cortex (areas 3, 1, and 2) (Figures 2E–H), the overall intensity of Shank3 IR was also higher in layers II-IV and VI relative to the remaining layers. A small number of pyramidal and multiple neurons in layers II, III, and VI showed moderate to strong IR in the somata and proximal dendrites (Figures 2B–D,F–H). Those in layer III of the precentral gyrus (PrC) appeared to be the large pyramidal cells in this region. A lack of any vascular labeling was noticeable in the sections of the PrC and postcentral gyrus (PoC), wherein no Shank3 IR occurred across the wall of intracortical blood vessels (Figures 2B,F,G). In sections from the occipital lobe, the border between areas 17 and 18 could be clearly identified based on a pattern transition of the IR over layer IV (Figure 2I). The sublayers of layer IV in the striate cortex (area 17) could be distinguished based on the difference in the intensity of Shank3 IR. Thus, layer IVa showed lighter neuropil labeling than the neighboring III and IVb, while layer IVc could be divided into IVcα and IVcβ, with the labeling intensity lighter in the former than the latter (Figures 2J,K). In contrast to area 17, the laminar pattern of Shank3 IR in area 18 was comparable to that seen in the frontal associative cortex described above (Figure 2I). Some immunolabeled neuronal somata were seen in both the primary and secondary visual cortices, including layer IVcβ (Figure 2L).

In the temporal lobe neocortex, the laminar distribution pattern of Shank3 IR was similar in the superior (STG), middle (MTG), and inferior (ITG) temporal gyri, and in the fusiform and neocortical part of the parahippocampal gyrus (PHG), which was comparable to that seen in associative frontal and visual cortical regions described above. Among these temporal neocortical regions, relatively large-sized pyramidal and multipolar neurons were observed in layers II–IV and VI (high magnification images not shown), also as mentioned precedingly in the frontal and visual cortical regions (Figures 2,3A).

#### Hippocampal Formation

The laminar distribution pattern of Shank3 IR changed around the basal part of the PHG as the cortex transited from the six-layered neocortex to three-layered paleocortex (Figure 3A). Thus, the lightly stained layer V became narrower and eventually unidentifiable as moving from the superior-lateral to inferior-medial subareas over the entorhinal cortex (Ent) (Figure 3A). Furthermore, basally, in the subicular subregions, Shank3 IR became primarily localized to the stratum pyramidale (s.p.) in the para-subiculum (Para-S), presubiculum (Pre-S), subiculum (Sub), and pro-subiculum (Pro-S) (Figures 3A,B). A large number of pyramidal neurons and their dendritic processes were visualized in these subicular subregions. The labeled apical dendrites and branches of the pyramidal neurons extended into the stratum radiatum (s.r.), while fairly intense neuropil IR occurred between the neuronal somata and processes (Figures 3B,C).

In the Ammon’s horn (CA1-3), fairly distinct Shank3 IR occurred over the s.p., localizing to the somata and dendritic processes of the pyramidal neurons. Little or minimal Shank IR was found in the stratum oriens (s.o.) (Figure 3D). The apical dendrites and their branches of the pyramidal neurons were seen to extend across the s.r. into the stratum lacunosum-moleculare (s.l.m.) (Figure 3E). In the dentate gyrus (DG), the molecular layer (ML) exhibited the strongest Shank3 IR. At high magnification, the ML could be further divided into a lightly stained band occupying approximately the inner one-third of this layer (inner ML, or iML), and a heavily stained band over the outer two-third (outer ML, or oML) (Figures 3A,F). Neuronal somata in the granule cell layer (GCL) and many large-sized multipolar neurons in the hilus, likely representing hilar mossy cells, displayed moderate IR within their cell bodies (Figures 3F,G). It should be emphasized that clusters of some fine granular profiles were present between the large-sized hilar neurons at closer examination. These clustered structures appeared to represent the thorny excrescences (TE), i.e., the large complex spine organizations of the hilar mossy cells, according to their anatomical localization and morphological characteristics (Xu et al., 2019).

#### Amygdaloid Complex

Shank3 IR was present across the amygdalar complex largely involving a neuropil-dominant distribution pattern (Figure 4A). At low magnification, the overall intensity of the immunolabeling was noticeably higher in the lateral and basomedial nuclear groups than in the basolateral and central nuclear groups (Figure 4A). At high magnifications, neuronal somata and their proximal dendrites were displayed in all amygdalar subregions, including the lateral nuclear group (La) over its dorsal (LaD) and ventral (LaV) subdivisions (Figures 4B,C), the basolateral nuclear group (BL) over its dorsal (BLD), and ventromedial (BLVm) and ventrolateral (BLVl) subdivisions (Figures 4D–F), and the paralaminar nucleus (PLN) (Figures 4F,G). The labeled neuronal somata appeared to be relatively large-sized multipolar neurons, with their somal diameters reaching or above 25 μm (Figures 4C,E,G). Diffuse neuropil labeling occupied the areas between these labeled neuronal perikarya, which appeared as fine granular elements by close examination (Figures 4C,E,G).

#### Basal Ganglia

**FIGURE 5:**
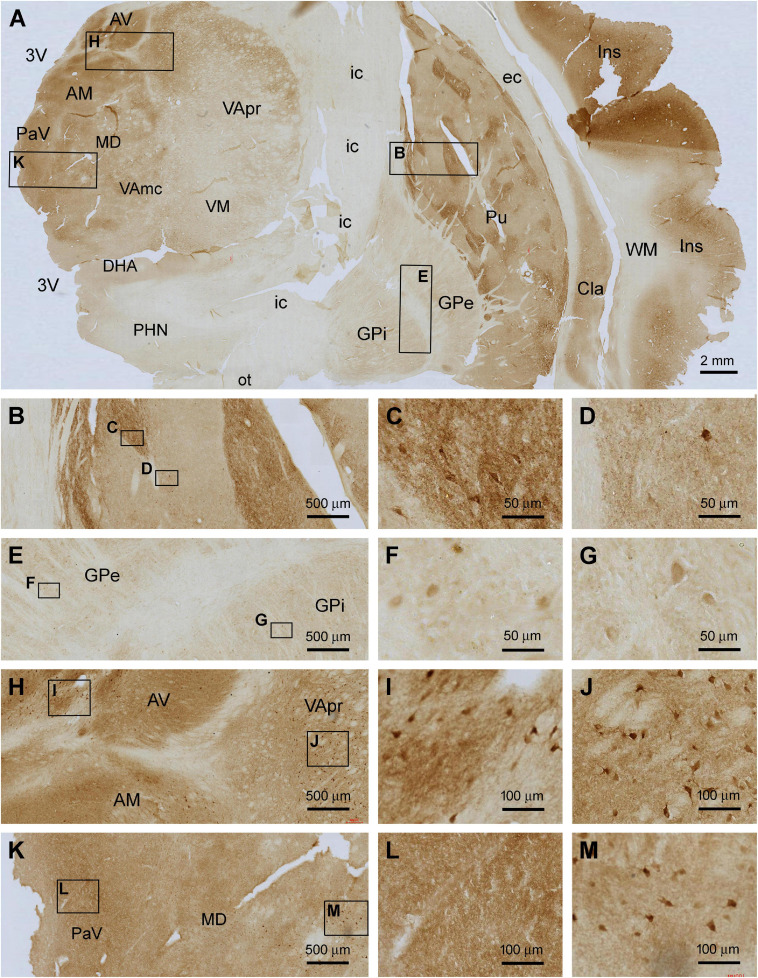
Localization of Shank3 IR in subregions of the basal ganglia and diencephalon. Panel **(A)** is a micrograph of the immunolabeled section passing the insula (Ins), striatum and thalamus, with framed areas enlarged as **(B–M)**. Shank3 IR in the putamen (Pu) displays a patching pattern, with strong labeling occurred in local areas separated by areas with much lighter staining. The former areas contain greater neuropil reactivity than the latter, whereas large-sized multipolar neurons are present in both areas **(B–D)**. Shank3 IR in the globus pallidus (GP) is much lower relative to the Pu and comparable between its external (GPe) and internal (GPe) subdivisions, both containing lightly stained neuropil and neuronal perikarya **(E–G)**. In the thalamus, the overall intensity of Shank3 IR is greater in the medial than the lateral regions **(A)**. The labeling occurs in the neuropil as well as neuronal somata **(H–M)**. No Shank3 IR is present in the white matter (WM), neural tracts or fiber bundles. AV, anterior ventral thalamic nucleus; AM, anterior medial thalamic nucleus; MD, mediodorsal nucleus of thalamus; VA, ventral anterior nucleus of thalamus; VApr, parvocellular division of VA; VAmc, magnocellular division of VA; PaV, paraventricular thalamic nuclei; VM, ventromedial thalamic nucleus; DHA, dorsal hypothalamic area; PHN, posterior hypothalamic nucleus; Cla, claustrum; EC, external capsule; IC, internal capsule; Cpd, cerebral peduncle; OT, optic tract; 3V, third ventricle. Scale bars are as indicated in each panel.

**FIGURE 6:**
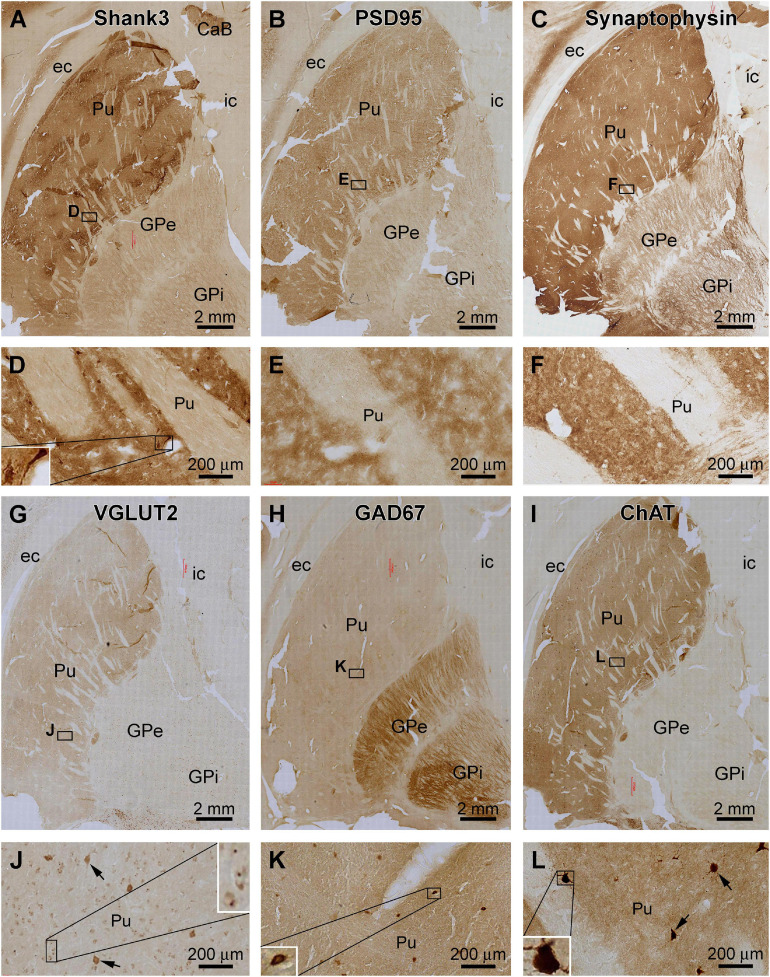
Comparative assessment of Shank3 IR relative to that of general synaptic markers and specific neuronal markers in the striatum. Low power views on the putamen (Pu) and globulus pallidum (GP) and enlarged areas are as indicated. The overall intensity of Shank3 **(A,D)**, postsynaptic density protein 95 (PSD95, **B,E**), synaptophysin **(C,F)**, vesicular glutamate transport 2 (VGLUT2, **G,J**), and choline acetyltransferase (ChAT, **I,L**) IR are heavier in the Pu than the GP. In contrast, the intensity of the glutamate decarboxylase 67 (GAD67) IR is greater in the GP than the Pu **(H,K)**. Besides neuropil reactivity, Shank3, VGLUT2, and ChAT IR occur in a population of large-sized multipolar neurons **(D,J,L)**, whereas GAD67 IR marks relatively small-sized neurons in round, oval or bipolar shape **(H)**. Punctate profiles of VGLUT2 IR are seen around the small-sized neurons in the Pu **(J)**. Abbreviations are as defined in Figure 5. Scale bars are as indicated in each panel.

In frontal plane sections passing the middle range of the insula, Shanks IR was found over the subregions of the striatum, thalamus, hypothalamus, and insular cortex itself (Figure 5A). In the striatum, Shanks IR was largely present in the putamen (Pu), whereas a pale reactivity occurred in the globus pallidus (GP), including its external (GPe) and internal (GPi) subdivisions. Notably, the distribution of the IR in the Pu was compartmented such that subareas with fairly intensive IR were separated by regions with light labeling; the former appeared to occupy less total area than the latter (Figure 5A). At high magnification, the compartments with stronger IR contained many large-sized multipolar neurons (Figures 5B,C), which appeared morphologically characteristic of the large spiny neurons, or the cholinergic neurons, in human striatum (Xu et al., 2019; Figures 5A–C). The overall intensity of IR in the lightly stained compartments was higher than that in the WM and internal capsule (IC), suggestive of the presence of some specific neuronal labeling. There also existed a small number of large-sized multipolar neurons in these regions (Figures 5A,B,D). The pattern of Shank3 IR in the caudate (see Figure 6A) was largely similar to that in the Pu. The IR in the GPe and GPi exhibited a similar distribution pattern, with lightly stained areas divided by unlabeled stripe-like areas that would represent neural fibers tracts. A small amount of lightly stained neuronal somata was present in both the GPe and GPi, which were also relatively large in size (Figures 5E–G). Between the striatum and neocortex, the claustrum (Cla) exhibited moderately intense IR comparable to the insular cortical gray matter (Figure 5A).

The anatomical compartmentation of the human striatum is related to a differential distribution of the excitatory and inhibitory neurons and their terminals (Friedman et al., 2015; Hamasaki and Goto, 2019; Prager and Plotkin, 2019). Therefore, we compared the pattern of Shank3 IR relative to general synaptic markers and a few specific neuronal phenotype markers between adjacent sections (Figure 6). Between the neostriatum, i.e., Pu, and paleostriatum, i.e., GP, the distribution of Shank3 IR (Figures 6A,D) was comparable to the IR of PSD95 (Figures 6B,E), synaptophysin (Figures 6C,F), VGLUT2 (Figures 6G,J) and ChAT (Figures 6I,L). Thus, these labeling were much enriched in the Pu relative to the GP. Weak neuropil labeling was seen in the GP for Shank3, PSD95, and synaptophysin, whereas VGLUT2 and ChAT exhibited little neuropil IR in the GP. In contrast to the above, GAD67 IR was much more intense in the GP than in the Pu (Figure 6H). As noted precedingly, large-sized multipolar neuronal somata in the Pu exhibited strong Shank3 IR in addition to intense neuropil labeling (Figure 6D). However, PSD95 and synaptophysin IR in this region were purely neuropil like (Figures 6E,F). VGLUT2 IR occurred in many neuronal somata in the Pu, including a small population of heavily stained and large-sized neuronal somata likely representing the spiny cholinergic neurons (Figure 6J). These large spiny cholinergic neurons were clearly visualized in the ChAT immunolabeled sections (Figure 6L). Weak VGLUT2 IR appeared also to occur in small-sized neurons in the Pu. However, the immunolabeling was present around the cell membrane, sometimes punctuated or granular in shape (Figure 6J), which might be suggestive of perisomal localization of glutamatergic terminals on small-sized striatal neurons. In GAD67 immunolabeled sections, small-sized neuronal somata were clearly visualized at high magnification, and they were present mostly in the neostriatum but also found in the paleostriatum (Figures 6H,K).

#### Diencephalon

The diencephalon exhibited a mixed pattern of Shank3 IR across its subregions (Figure 5A). The labeling intensity was generally greater in the medial nuclear groups relative to the lateral groups in the thalamus and hypothalamus, which appeared to be largely related to a heavier neuropil reactivity in the former. Thus, there existed fairly dense neuropil labeling in the anterior thalamic nuclear group, including the ventral (AV) and medial (AM) subdivisions (Figures 5A,H), and the paraventricular (PaV) and medial nuclear groups of the thalamus, including the medial dorsal nucleus (MD) (Figures 5A,H–M). In comparison, less intense labeling was present in the ventral nuclear complex, including parvocellular division (VApr), magnocellular division (VAmc), and the ventral medial nucleus (VM) (Figure 5A). However, labeled neuronal somata were found in essentially all above nuclei, most of them being multipolar in shape and relatively large in size (with a somal diameter ∼20 μm or above) (Figures 5H–M). In the hypothalamus, weak Shank3 IR was present in most subnuclei, for example, the dorsal anterior (DHA) and posterior (PHN) (Figure 5A).

#### Cerebellum

**FIGURE 7:**
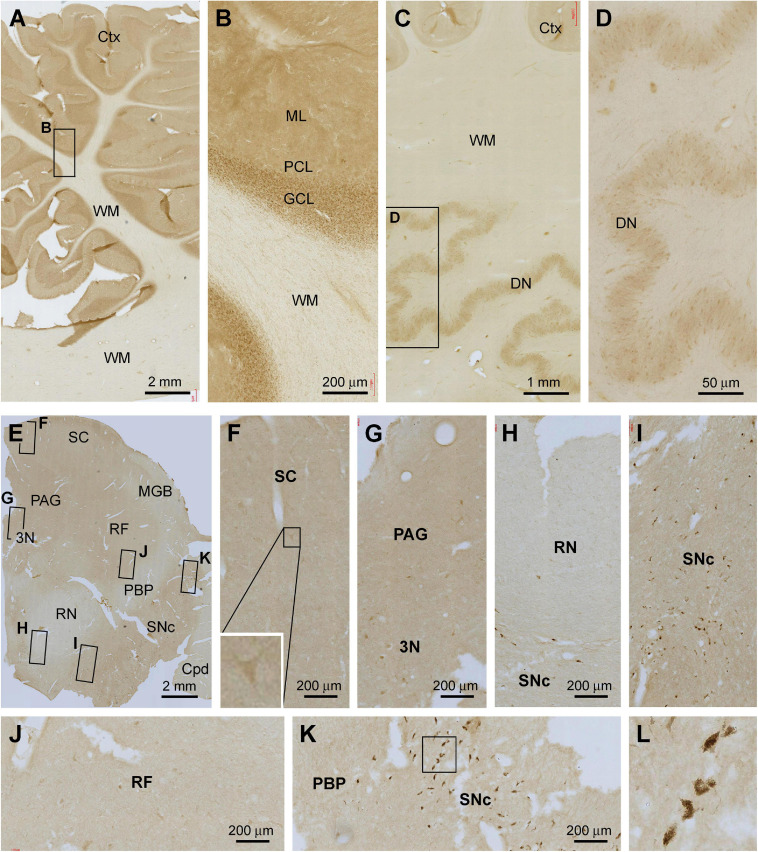
Representative images illustrating Shank3 IR in the cerebellum and midbrain. Panels **(A,B)** show the labeling in the cerebellar cortex, with diffuse neuropil labeling occurred across the molecular layer (ML), cellular labeling in the granule cell layer (GCL), whereas no labeling in the Purkinje cell layer (PCL) and the white matter (WM) **(B)**. Panels **(C,D)** show the IR in the dentate nucleus, with the light neuropil and cellular labeling seen in the mantle of this nucleus **(D)**. Panels **(E–L)** show Shank3 IR in a section at the level of the superior colliculus (SC). Light labeling is present across the section, while individual neuronal perikarya are visible at high magnification in various areas as indicated. The cells in the pas compacta substantia nigra (SNc) are pigmented (unlikely representing specific immunolabeling) **(I,K,L)**. PAG, periaqueduct gray; MGB, medial geniculate body; 3N, third cranial nerve nucleus; RF, reticular formation; RN, red nucleus; PBP, parabrachial pigmented nucleus; Cpd, cerebral peduncle. Scale bars are as indicated in each panel.

The overall intensity of Shank3 IR in the cerebellar sections appeared to be lighter in comparison with that seen in the forebrain regions as described above (Figures 7A–D). Thus, in the cortex, the IR occurred across the molecular layer (ML) as diffuse neuropil-like reactivity. Neither the somata nor dendritic tree of the Purkinje cells was labeled. However, there was moderate IR across the granule cell layer (GCL), with the labeled granule cells densely packed by close examination (Figure 7B). No specific Shank3 IR was present in the cerebellar WM. In the dentate nucleus (DN), Shank3 IR was also weak and occurred in the neuropil as well as neuronal somata inside the mantle. No specific IR was seen in the cavity of this deep cerebellar nucleus (Figures 7C,D).

#### Brainstem

In the midbrain, weak Shank3 IR was present in the superior colliculus (SC), periaqueductal gray (PAG), reticular formation (RF), and pars compacta substantia nigra (SNc), which appeared to largely involve neuropil labeling (Figures 7E–L). In comparison, the IR was even lighter in the medial geniculate body (MGB), red nucleus (RN), parabrachial pigmented nucleus (PBP), and cerebral peduncle (Cpd). At high magnifications, lightly stained multipolar and bipolar neurons could be identified in the gray matter regions and the RF (Figures 7F–H,J). Non-specific pigment staining was seen among large perikarya in the SNc among the likely dopaminergic neurons (Figures 7H,I,K,L).

**FIGURE 8:**
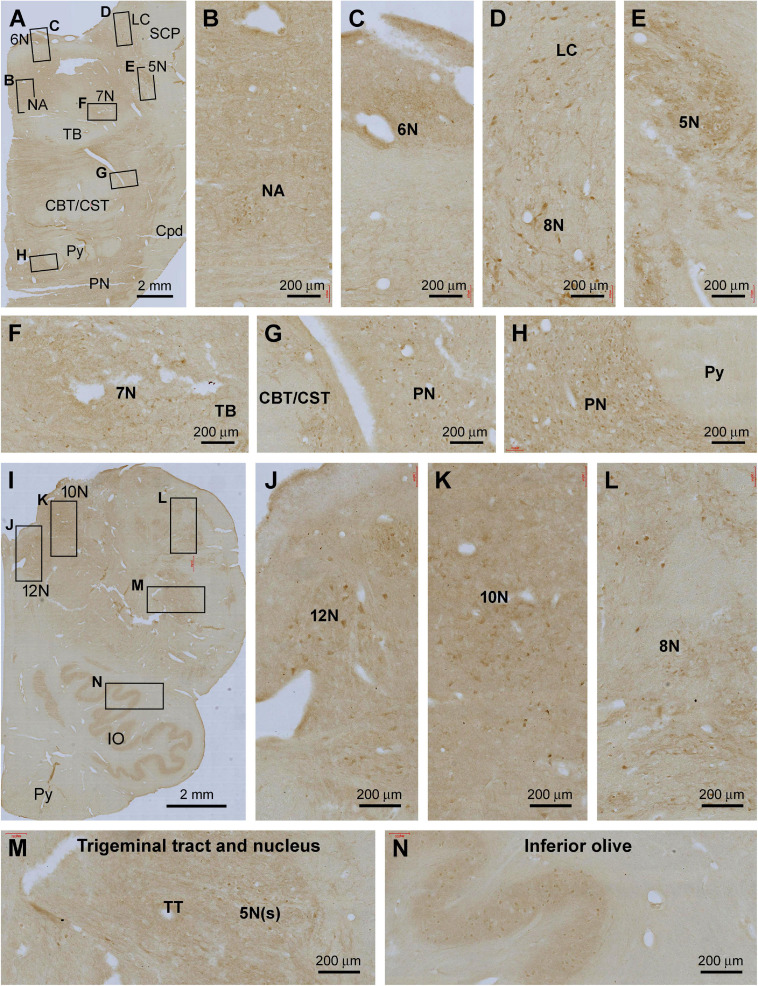
Representative images illustrating Shank3 IR in the pons and medulla oblongata. **(A–H)** show the low magnification distribution pattern and enlarged views in the subregions of the pons. **(I–N)** illustrate the overall pattern and local views of the labeling in the medulla oblongata. In general, lightly stained neuropil and neuronal perikarya are seen at most cranial nerve nuclei and specific brainstem nuclei, including the locus coeruleus (LC); inferior olive (IV) and pontine nuclei (PN), while no labeling is seen in neural fiber bundles **(B–H,J–N)**. 5–12 N, main nuclei of the 5–12 cranial nerves; NA, nucleus ambiguous; SCP, superior cerebellar peduncle; TB, triphorid body; CBT, CST, the corticobulbar and corticospinal tracts; TT, trigeminal tract; Py, pyramidal tract. Scale bars are as indicated in each panel.

In the pons, Shank IR was weak in general across the entire transverse section, with the labeling largely localized to the gray matter and nuclei around the base of the fourth ventricle (4V) (Figure 8A). Thus, diffuse neuropil labeling, along with individually stained neuronal somata, was visible in the 5th to 8th cranial nuclei, the nucleus ambiguous (NA), the dorsal raphe nucleus (DR), and the locus coeruleus (LC). Light neuropil and neuronal somata labeling existed in the pontine nuclei (PN). The neural fiber systems of the pons were essentially unlabeled, including the central tegmental tract (CTT), triphorid body (TB), superior cerebellar peduncle (SCP), lateral lemniscus (LL), pontocerebellar tract (PCT), and the corticobulbar and corticospinal tracts (CBT, CST) (Figure 8A).

In the medulla oblongata (MO), Shank IR was also fairly weak as observed, for example, across the entire transverse section at the levels of the inferior olivary (IO) complex. At high magnification, lightly stained neuronal somata were arranged in groups anatomically corresponding to the facial nucleus (7N), cochlear and vestibular nuclear complex (8N), nucleus of the solitary tract (SoN), sensory trigeminal nuclei (5V), dorsal nucleus of vagus (10N), and hypoglossal nucleus (12N), in addition to the main and accessory nuclei of the IO. No labeling was seen in the cavity or hilus of the IO nuclei and the pyramidal tract (Py) (Figures 8B–L).

### Double Immunofluorescent Characterization of Shank3 Expression

**FIGURE 9:**
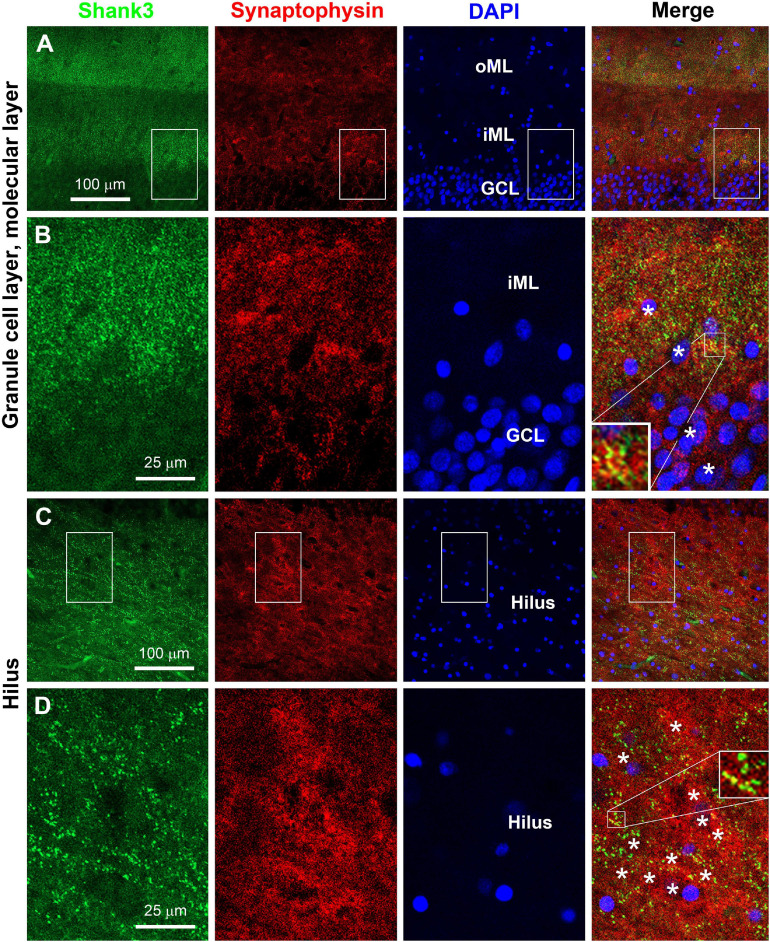
Double immunofluorescent characterization of Shank3 localization relative to presynaptic terminals. Panels **(A–D)** are confocal images of Shank3, synaptophysin, and DAPI labeling in the dentate gyrus with one scan of 1-μm section thickness. Sublemmal localization of Shank3 IR in the granule cells and large hilar cells is noticeable at high magnification **(B,D)**. Synaptophysin positive presynaptic terminals are punctate profiles, with a subset appeared to “colocalized” with Shank3 IR (**B,D**, the most left panels, inserts). Neuroanatomical locations, enlarged zones, and scale bars are as indicated. *: location of neuronal somata.

We attempted to verify postsynaptic localization of Shank3 in hippocampal sections by double immunofluorescence with the mouse anti-Shank3 along with the rabbit anti-synaptophysin and with the rabbit anti-PSD95 antibodies. Overall, the Shank3 immunofluorescent labeling in the sections was not as intense as seen in immunohistochemical preparations involving avidin-biotin signal magnification. We were able to identify the localization of Shank3 IR in neuronal somata and dendrites apposing to distinct presynaptic synaptophysin IR in the dentate gyrus. Thus, Shank3 IR was found in the somata of the granule cells, with puncta profiles localized around the outline of somal perikarya (Figures 9A,B). In the ML, punctate Shank3 and synaptophysin IR were mostly not colocalized, while some appeared to be colocalized (yellowish in merged images), suggestive of a close spatial relationship (Figure 9B, insert). In the hilus, Shank3 IR also occurred in the somata and dendrites of largely neuronal profiles, with a relatively denser distribution around the cell membrane, which was aligned by synaptophysin immunoreactive puncta (Figures 9C,D). Again, closer examination indicated that a subset of presynaptic terminals showed putative colocalization with Shank3 IR, i.e., appeared yellow or yellow-green in the merged image, also indicating spatial closeness (Figure 9D, insert).

### Immunoblot Assessment of Shank3 Expression Across Brain Regions

**FIGURE 10:**
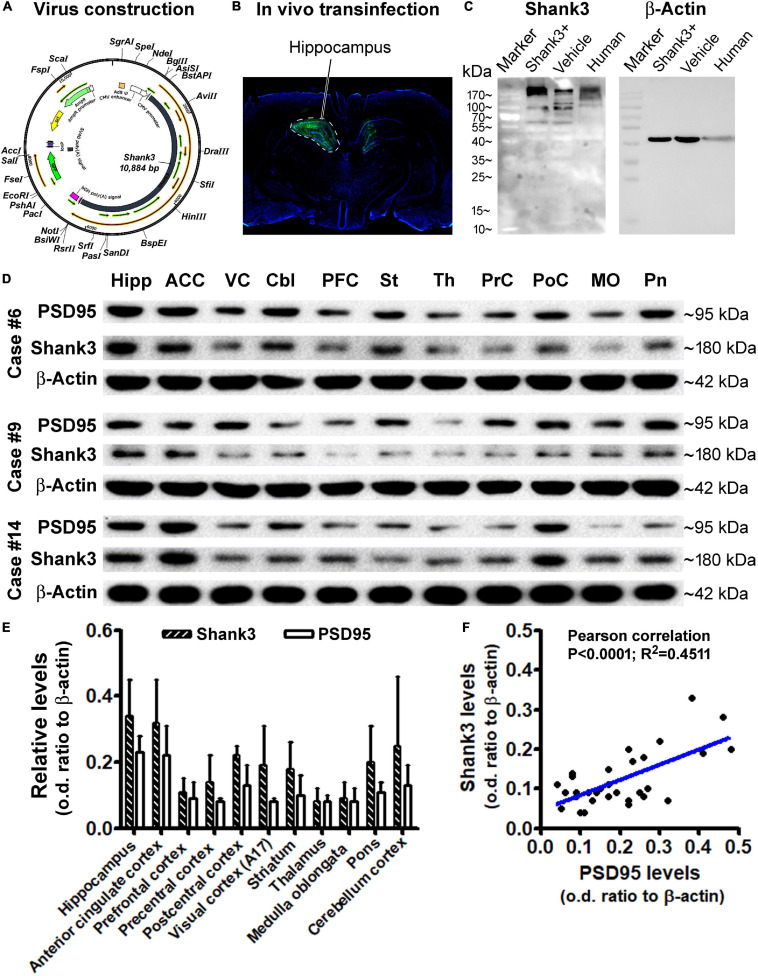
Western blot characterization of the Shank3 antibody and assessment of the Shank3 protein levels in representative brain regions. **(A)** Schematically illustrates the adenovirus genomic structure, including manipulating apparatus and the human *SHANK3* cDNA. **(B)** shows an example of *in vivo* infection in the rat hippocampus 30 days post unilateral stereotactical injection of the constructed virus. **(C)** shows a representative immunoblot image, illustrating the relative levels of Shank3 protein in the hippocampal lysates from rats infected with the constructed virus (Shank+) and vehicle virus, and postmortem human brain. The major immunoblotted products migrate at ∼180 kDa in all samples; several light bands also migrated from 90 to 180 kDa, with an apparently greater amount in the Shank + transinfected sample. **(D)** shows immunoblotted products of Shank3, PSD95, and β-actin (internal loading control) in lysates from different brain regions from three cases, as indicated. **(E)** Plots the relative levels of Shank3 and PSD95, expressed as optic density (o.d.) ratios normalized to internal control across regions. **(F)** shows a positive correlation between Shank3 and PSD95 levels based on the multi-region and multi-case immunoblotting data. Abbreviations of brain regions in **(D)** are as indicated correspondingly in **(E)**.

Using brain material from an ongoing separate study involving the application of *in vivo* virus-driving Shank3 overexpression in rat hippocampus, we evaluated the specificity of the Shank3 antibody by Western blot. Briefly, the *Shank3* gene was constructed into an adenovirus packing system (ADMAX^TM^), containing the green fluorescence protein (GFP) gene (Figure 10A), which was stably expressed in the human embryonic kidney (HEK) 293 cells (Vigene Biosciences, Inc., Changsha, China). Harvested virus with and without (vehicle control virus) the *Shank3* gene was stereotactically injected into the hippocampus of rats (Figure 10B). The infected rat hippocampi, together with the frozen human hippocampal sample, were prepared for immunoblotting. The blotted Shank3 protein product from the transinfected and control rat hippocampal lysates, as well as the human hippocampal lysate, was migrated as a major band at ∼180 kDa, consistent with the predicted molecular weight of this protein. The Shank3 transinfected rat hippocampal lysates contained apparently higher content of the target protein relative to virus vehicle control (Figure 10C). It should be noted that several light bands migrated at 90–180 kDa were also visible, especially when a longer exposure time was used to capture the immunoblotting images (Supplementary Data). Nonetheless, the 180 kDa band was the most heavily and consistently blotted product in human brain lysates. As it also represented the expected Shank3 protein, we focused on this blotted product in our quantitative analyses of western blot studies of human brain lysates.

After the aforementioned antibody verification and optimization of immunoblotting conditions (data not shown), we assayed the levels of Shank3 in lysates prepared from selected regions/structures of postmortem human brains (cases #6, #9, and #14). The levels of PSD95 in the same lysates were simultaneously blotted for a comparative analysis of these two postsynaptic markers. Based on densitometry, the mean levels of immunoblotted Shank3 and PSD95 appeared to be relatively high in lysates from the hippocampus (Hipp), anterior cingulate cortex (ACC), and cerebellar cortex (CBL), relative to other regions, including the precentral (PrC) and postcentral (PoC), primary visual cortex (VC), and some subcortical structures, including the striatum (St), thalamus (Th), pons (Pn), and medulla oblongata (MO) (Figures 10D,E). Based on one-way ANOVA test, there was an overall difference among the mean optic density (o.d.) ratios (normalized to internal loading control, β-actin) of blotted Shank3 protein (*P* = 0.0052; df = 10.22; *F* = 3.68). However, *post hoc* Bonferroni’s multiple comparison test indicated no statistically significant differences of the means between individual brain regions. Similarly, the levels of blotted PSD95 protein did not reach statistically significant differences between individual brain regions according to one-way ANOVA test (*P* = 0.0732; df = 10.22; *F* = 2.08). However, the levels of blotted Shank3 and PSD95 proteins in the same lysate showed a tight positive correlation when plotted against each other, regardless of brain regions and cases (Figure 10F). This result indicated an interdependent relationship between these two postsynaptic proteins in the same regional brain samples. The lack of significant regional difference in the mean levels of Shank3 and PSD95 between individual brain regions appeared to be related to the great variations between the cases analyzed, which might be involved in the effect of age on the expression of synaptic marker proteins (as addressed below).

### Altered Cellular and Neuropil Shank3 Labeling in Aged and AD Brains

Histological study of postmortem human brains could be always subjected to some inherent pitfalls because of the effect of postmortem delay on the preservation of endogenous proteins. Therefore, “pattern recognition” could be a rather practical approach to comparative morphological, especially pathological, examination in human brain research. As described precedingly, Shank3 IR showed fairly characteristic laminar and cellular patterns in the cerebral neocortex, hippocampal formation, and cerebellar cortex; therefore, we focused on examination in these regions in aged and AD cases, relative to that seen in the youth and adult cases.

**FIGURE 11:**
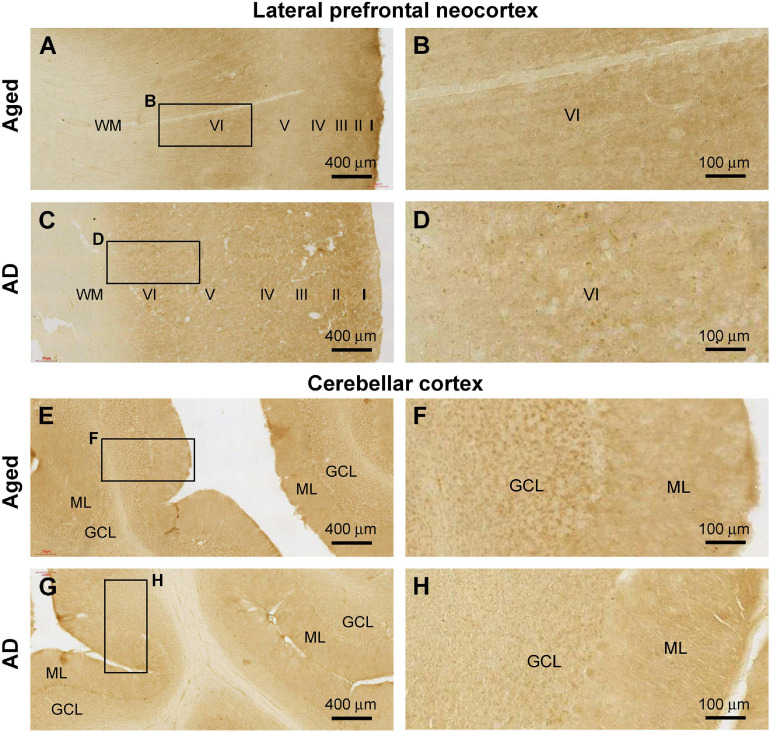
Representative images illustrating altered Shank3 IR in the frontal neocortex and cerebellar cortex in the aged (case# 15 in Table 1) and Alzheimer’s disease (AD, case# 26 in Table 1) human brains. **(A,B)** illustrate preservation of the laminar pattern of Shank3 IR in the neuropil but loss of labeled neuronal somata in the aged brain relative to that seen in the adult (in reference to Figures 1E–H). Panels **(C,D)** show a diminishment of the differential laminar distribution of IR in the neuropil in the AD brain (compare with **A,B** and also Figures 1E–H). Panels **(E–H)** show a trend of decrease of Shank3 in the cerebellar granule cell layer (GCL) in the aged and AD brains, also in reference to the relative intensities of IR over the molecular layer (ML) and GCL seen in adult cases (Figures 7A,B). Scale bars are as indicated.

A total of 14 brains from donors aged above 65 years were included in the current study to study Shank3 IR in the cerebral and cerebellar sections. Among these brains from elderly individuals, seven cases were pathologically characteristic of PART (Shi et al., 2020); one case showed tauopathy together with early Aβ lesion at Thal phase 1, with the remaining six cases had late-stage pTau (Braak Stage IV and above) and Aβ (Thal Phase 4 and above) burdens met the criteria for neuropathological diagnosis of AD according to the NIH guideline (Table 1). On examination of cerebral sections from these cases, we noticed a trend of reduction and even loss of Shank3 immunoreactive neuronal somata in the associative and primary functional areas (Figures 11A–D, as examples), in comparison with that seen in sections from the youth or adult cases (Figures 1, 2). In addition, the neuropil Shank3 IR showed a trend of loss of the laminar variability in the age/AD cases relative to the youth/adult cases (Figures 1E–L, 11A–D). On examination of the cerebellar cortical sections, we also noticed a trend of Shank3 IR in the GCL in the aged/AD cases relative to the youth/adult cases. Thus, in the aged/AD cases, the overall intensity of Shank3 IR tended to be comparable between the GCL and the ML (Figures 11E–H), whereas in the youth/adult cases, there was greater Shank3 IR in the GCL than in the ML (Figures 7A,B).

**FIGURE 12:**
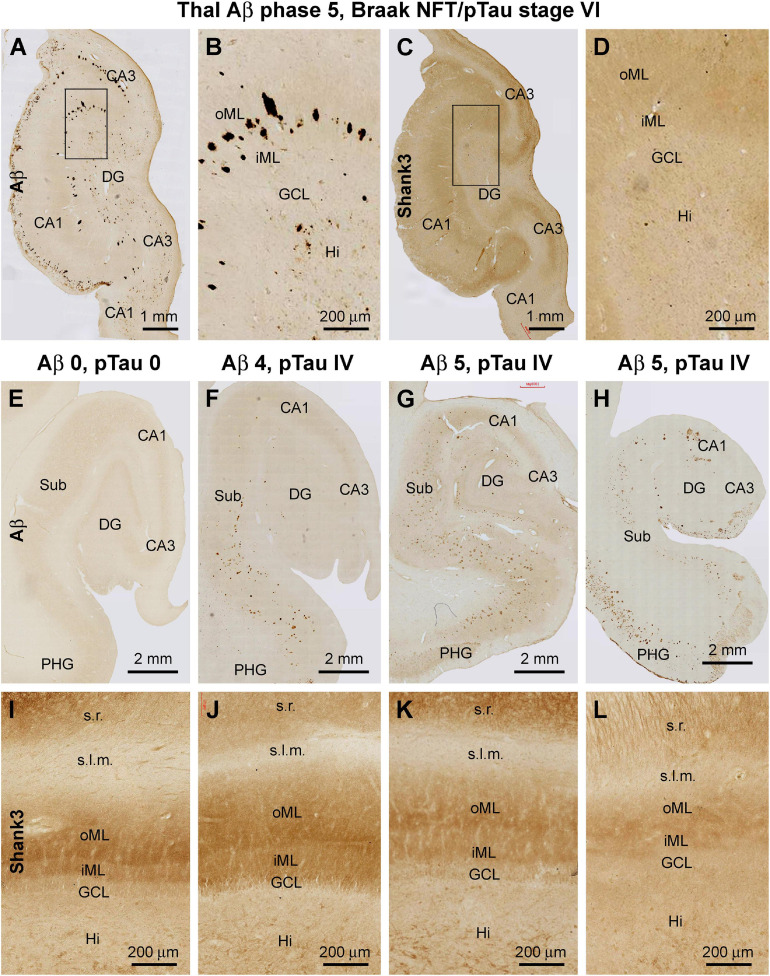
Representative images illustrating altered Shank3 IR, the molecular layer of the dentate gyrus (DG) in human brains with late-stage Alzheimer’s disease (AD) pathology. The Braak staging of neurofibrillary tangle (images not shown) and Thal staging of β-amyloid (Aβ) pathology for the cases are indicated for the groups of panels. Panels **(A–D)** show Aβ and Shank3 IR over the DG in an AD brain with severe brain atrophy; note the loss of Shank3 IR and its lamination over the inner (iML) and outer (oML) molecular layer. Panels **(E–H)** show images of Aβ IR over the hippocampal formation from an aged case without AD-type pathology **(E)** and with plaque lesions with increasing extent. Panels **(I–L)** show the intensity and lamination of Shank3 IR over the ML in these cases correspondingly. Note the decrease of Shank3 IR in the ML along with a disruption of the lamination (including thinning) that appears to be progressive in parallel with the severity of the Aβ lesion. Abbreviations are as defined in Figure 3. Scale bars are as indicated in the image panels.

A more microscopically prominent alteration in the laminar pattern of Shank3 IR was observed in the DG in cases with late-stage AD neuropathology relative to youth/adult cases and even to the aged cases without Aβ and pTau lesions in the hippocampal formation. Thus, in cases with apparent hippocampal Aβ/pTau lesions and parenchymal atrophy, there was an overall loss of Shank3 IR across the DG, with the differential laminar organization in the ML completely unrecognizable (Figures 12A–D). The loss and disruption of the Shank3 neuropil IR over the ML appeared to progressively worsen with the advance of Aβ/pTau pathology in the hippocampal formation when compared between brain cases. Thus, this change in the pattern of neuropil labeling occurred first in the oML then spread to the entire ML in parallel with the thinning of this layer (Figures 12E–L).

### Assessment of Age/AD-Related Change in Shank3 Expression

**FIGURE 13:**
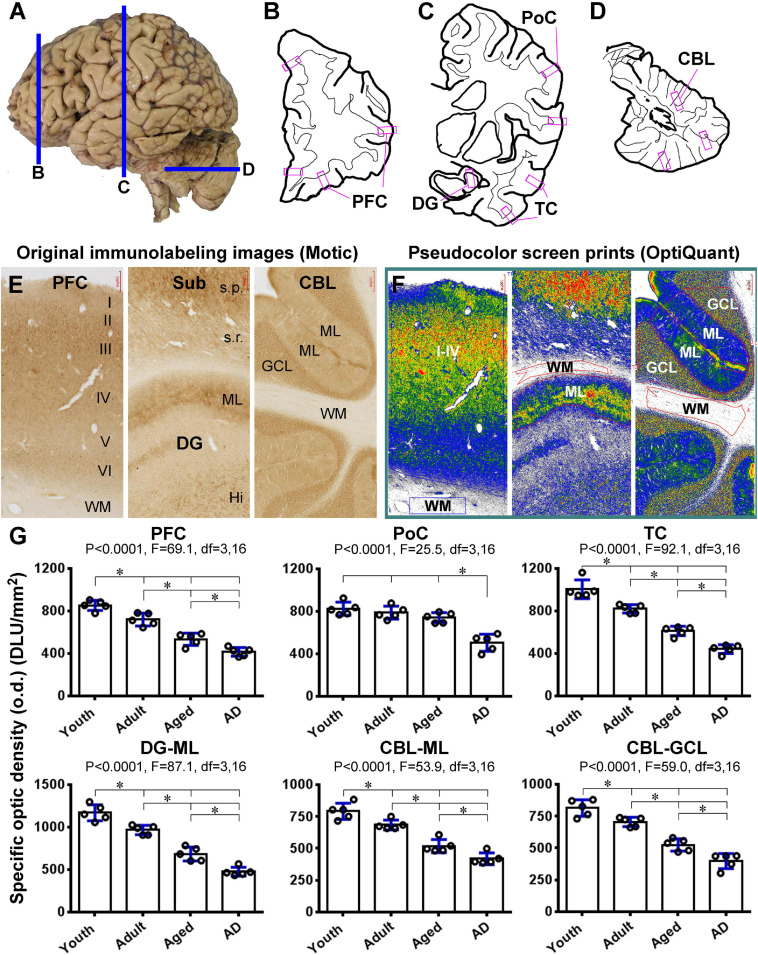
Densitometric analysis of age- and AD-related decrease in Shank3 IR in representative human brain subregions. **(A–D)** show the sampling of brain sections relative to the lateral view of the human brain, with the shapes of a frontal slice passing the prefrontal cortex **(B)**, a frontal slice passing postcentral gyrus and mid-hippocampus **(C)** and a horizontal slice passing the mid-cerebellum **(D)**, drawn schematically. The framed areas marked in purple represent the approximate locations of the “areas of interest” for the measurement of optic densities (o.d.) of Shank3 IR in the sections within the above thick brain slices. Panel **(E)** shows the original images extracted at 4 × magnification from gyral portions of the prefrontal neocortex, the hippocampal formation covering the middle segment of the dentate gyrus and cortex of the posterior cerebellar lobe, using the Motic-scanned whole region images. Panel **(F)** is corresponding pseudocolor screen prints of OptiQuant working interfaces, wherein measuring templates are illustrated. Thus, the o.d. (expressed as DLU/mm^2^) obtained from the gray matter regions (i.e., layers I–VI, ML, GCL) is defined as the total density of the IR, while the o.d. measured in the nearby white matter (WM) area is considered as the background (the cutoff level). The specific o.d. is calculated by subtracting the background from the total o.d. in the corresponding areas of interest. Panel **(G)** pots the means of specific o.d. of Shank3 IR obtained from the indicated neocortical areas (layers I–VI), the molecular layer (ML) of the dentate gyrus (DG), and the ML and GCL of the cerebellar cortex in individual brains in youth, adult, aged, and AD groups. Statistics (one-way ANOVA with Bonferroni’s *post hoc* test) are as indicated. Additional abbreviations are as defined in Figure 3. * indicating significant difference of the means of individual pairing groups according to Bonferroni’s multiple comparison *post hoc* test.

To quantitatively assess the effect of age and AD-like pathology on Shank3 expression, densitometric analyses were carried out in selected neocortical, hippocampal, and cerebellar areas, using the Motic images of immunolabeled sections from the prefrontal lobe, the parietal and temporal lobes at the mid-hippocampal level, and posterior lobe of the cerebellum (Figures 13A–D). Images were extracted from the gyral portions of the above neocortical areas, the middle portion of the DG, and cerebellar cortex, with the regions of the gray and a part of the white matter areas included. The optic densities (o.d.) values (DLU/mm^2^) from the white matter regions were used as the cutoff levels to define the specific o.d. in the gray matter regions (Figures 13E,F). Five brains cases in each of the youth (17.2 ± 4.1 years, mean ± S.D., same below), adult (48.4 ± 6.3 years), aged (77.4 ± 5.5 years), and AD (83.4 ± 5.6 years) groups were used for these densitometric analyses (Table 1).

In the PFC, the specific o.d. measured over layers I-VI was 852.9 ± 42.8, 719.5 ± 53.3, 533.9 ± 52., and 415.3 ± 36.8 DLU/mm^2^ (mean ± S.D., same below) in the youth, adult, aged, and AD groups, respectively. There was an overall significant difference among the means of the groups by one-way ANOVA analysis, as were intergroup differences by Bonferroni’s *post hoc* test (Figure 13G). In the PoC, the estimated o.d. values were 824.5 ± 55.5, 787.1 ± 54.4, 740.3 ± 43.1, and 503.8 ± 72. DLU/mm^2^ in the groups of the same order, respectively, with the density in the AD group significantly reduced relative to the other groups (Figure 13G). In the temporal neocortex (TC), the values were 1005. ± 88.6, 820. ± 39., 611.2 ± 44.1, and 442. ± 41.4 DLU/mm^2^ for the groups, respectively, which showed significant overall and intergroup differences (Figure 13G). The values obtained from the ML of the DG were 1,168.6 ± 94.3, 966.1 ± 55.5, 683.5 ± 81.6, and 476.3 ± 52.7 DLU/mm^2^ for the groups, respectively, with significant overall difference among the groups as well as differences between each pairing group (Figure 13G). In the cerebellar cortex, the values were 790.9 ± 63.4, 683.9 ± 38., 517.9 ± 51.5, and 418.6 ± 46.3 DLU/mm^2^ in the ML (Figure 13G), and 813.2 ± 65.1, 704.5 ± 36.9, 524.8 ± 48., and 397.2 ± 59.4 DLU/mm^2^ in the GCL (Figure 13G), obtained from the groups, respectively. Statistical analysis indicated that the density of Shank3 IR in both layers was reduced progressively in the adult, aged, and AD groups (Figure 13G).

**FIGURE 14:**
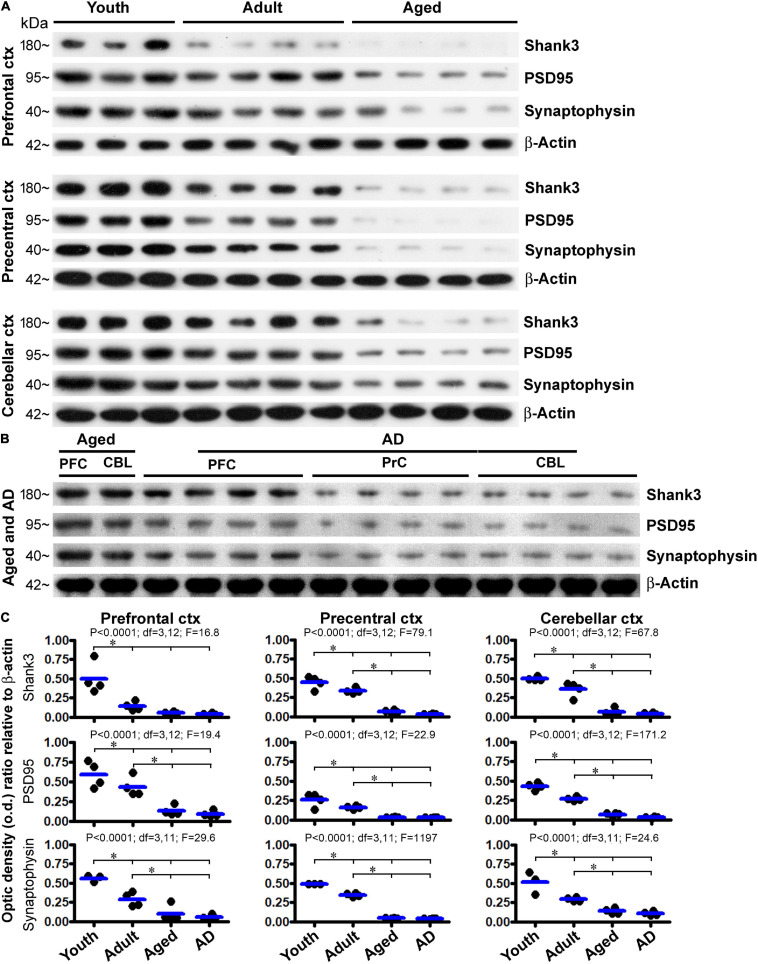
Western blot profiling of age-related decline in the levels of Shank3, PSD95, and synaptophysin in the human prefrontal and precentral cerebral neocortex and the cerebellar cortex. Panel **(A)** shows images of the sets of immunoblotted protein bands, with the cases, groups, and protein markers as indicated. Protein loading was 10 μg for all samples. Panel **(B)** shows immunoblotting images from another set of experiment to assess the protein levels in Alzheimer’s disease (AD) cases. Samples (lanes 1 and 2) from an aged case were included as a reference. In addition, a longer exposure time (than that for capturing the image shown in **A**) was used to capture the image, given the low signal of the blotted protein already seen in the aged group **(A)**. Panel **(C)** plots the densitometric data and results of statistical analyses. *P*, df, and *F* values noted in each panel are based on one-way ANOVA, with * indicating significant difference of the means of individual pairing groups according to Bonferroni’s multiple comparison *post hoc* test.

We also assessed the levels of Shank3 protein by immunoblot, using the prefrontal and precentral neocortices, and the cerebellar cortex, as representative brain regions. Cases were selected to form four age groups, i.e., youth (18.5 ± 1.7 years, *n* = 4), adult (43.8 ± 6.7 years, *n* = 4), aged (76.5 ± 2.1 years, *n* = 4), and AD (86. ± 1. years, *n* = 4) groups (Table 1). Levels of Shank3, PSD95, and synaptophysin in the same set of lysates were assayed in parallel, relative to the loading control β-actin. Briefly, for all of the three synaptic marker proteins, the amounts of blotted proteins in the lysates from the three brain regions showed a similar trend of progressive decline in the adult, aged, and AD groups relative to the youth group (Figures 14A,B). The levels of Shank3 protein in the prefrontal cortex were dramatically reduced already in the adult group, resulting in a significant difference in the mean o.d. in this group in comparison with the adult and aged/AD groups (Figure 14C, top row, left graph). The levels of Shank3 protein in the precentral neocortex and cerebellar neocortex were reduced in a stepwise manner such that mean density reached significant difference between the youth, adult, and aged/AD groups (Figure 14C, top row, middle, and right graphs). Levels of PSD95 and synaptophysin were also reduced in the three assayed regions largely in a stepwise manner from youth to aged/AD groups. The levels of the proteins did not reach significantly different differences between the aged and AD groups (Figure 14C).

## Discussion

### Shank3 Is Expressed in Putative Excitatory Postsynaptic Sites and Neuronal Subpopulations

Excitatory neurotransmission plays a fundamental role in governing neural network function. Shank3 is a known postsynaptic scaffolding protein of the excitatory synapses (Lim et al., 1999; Boeckers et al., 2002; Tao-Cheng et al., 2010, 2015); mapping its distribution and relative density in the brain can provide information on the anatomical setting of the central excitatory synaptic system and, possibly, the functional status of the postsynaptic apparatus thereof. There are limited existing data on the expression and distribution of the Shank3 messenger and protein in mammalian brains, largely involved rodent studies to date. Thus, in the adult mouse brain, Shank3 mRNA transcripts are highly and differentially expressed in many regions of the brain, including the hippocampus and cerebellum (Welch et al., 2004). Notably, Shank3 protein is also localized to central cholinergic synapses and the neuromuscular junction (Welch et al., 2004). In the rat brain, Shank3 mRNA expression increases during postnatal development, especially in the hippocampus, cerebellum, and thalamus, with the transcripts also occurring in the hippocampal molecular layer and cerebellar granule cells (Böckers et al., 2004; Kindler et al., 2004). The latter laminar and cellular localization is particularly in line with the neurochemical characterization of Shank3 as primarily an excitatory postsynaptic protein in the brain (Boeckers et al., 1999; Lim et al., 1999; Boeckers et al., 2002).

In the current study, we used a mouse antihuman Shank3 antibody to map the expression of this protein in the human brain. The antibody labeled a major band ∼180 kDa in immunoblot, consistent with the predicted molecular weight of this protein. The amount of this ∼180 kDa product was dramatically increased in the rat hippocampus transinfected by a constructed adenovirus over-expressing the *SHANK* gene. No immunolabeling appeared in human brain sections in the primary antibody omission test. Moreover, there existed parallelism between Shank3 and PSD95 in tissue extracts from different brain regions containing varying amounts of these postsynaptic proteins. These results together indicate that the primary antibody used specifically labels Shank3 biochemically characteristics of a postsynaptic protein.

We observed a differential pattern of Shank3 IR from the perspective of its regional, laminar, and cellular localization across the human brain as assessed in samples from youth/adult cases with fairly short postmortem delays (<12 h). Overall, Shanks IR was microscopically prominent in most forebrain structures, including the cerebral neocortex, amygdalar complex, hippocampal formation, striatum, and thalamus, all exhibiting a neuropil dominant distribution pattern. In comparison, lighter labeling existed in the cerebellum, midbrain, and brainstem. The IR occurred essentially in the gray matter and regions with mixed neuronal somata and fiber systems but was absent in the white matter and neural tracts either longitudinally and transversely sectioned, indicating a minimal expression of Shank3 in axonal elements. Because no IR was detectable in any type of glial cells, the neuropil labeling pattern points to localization of Shank3 IR in fine dendritic elements and/or synaptic profiles. The laminar pattern seen in the DG particularly fits the notion that Shank3 is expressed primarily in the fine dendritic processes and spines of the granule cells. Thus, Shank3 IR was heavier in the oML than in the iML; these two molecular sublayers correspond respectively to the distal and proximal dendritic arborization of the granule cells. No labeling was detectable in the axons of the granule cells, the mossy fibers, in the hilus or CA3. A similar interpretation of excitatory postsynaptic localization may be applied to the strong Shank3 IR in the olfactory glomeruli, also in consideration of the lack of any evident axonal labeling across the entire brain. Moreover, the differential neuropil pattern of Shank3 IR between the neo- and paleostriatum was more matchable to that of PSD95, VGLUT2, and ChAT than to that of synaptophysin and GAD67. This implicates a close anatomical correlation of postsynaptic Shank3 IR with the glutamatergic and cholinergic rather than GABAergic axon terminals. Consistent with the above, the double immunofluorescent characterization showed Shank3 IR inside the somata and dendritic processes of dentate granule cells and hilar cells, with a noticeable concentration at the sublemmal regions in line with a postsynaptic location. Shank3 IR was also found to closely apposing to some punctate profiles labeled with the general presynaptic terminal marker synaptophysin. In fact, partial “colocalization” of Shank3 and synaptophysin IR at some axonal terminal profiles could be observed at the light microscopical level, which could be related to an overlap (top–down) of pre- and postsynaptic elements.

In the cerebral cortex, amygdalar complex and hippocampal formation, Shank3 IR occurred in the somata and proximal dendrites of relatively large-sized pyramidal or multipolar neurons, especially noticeable in the brains of youths and adults. Large-sized multipolar neurons were also found in the striatum and thalamus, which resembled morphologically the cholinergic neurons assessed in the adjacent brain sections. In the cerebellar cortex, Shank3 IR was most prominent in excitatory granule cells. We also observed light Shank3 IR among subpopulations of neurons across the brainstem, which was often associated with the relay nuclei of the motor and sensory pathways. In addition, neurons in the LC, IO, and the cerebellar DN exhibited weak Shank3 IR in their somata and proximal dendrites. These findings support the notion that Shank3 is mainly expressed in excitative/projective neurons in the human brain, largely in the glutamatergic populations and also likely in cholinergic and noradrenalinergic neurons (Welch et al., 2004). The occurrence of Shank3 IR in neuronal somata and proximal dendrites implicates that the protein can be synthesized in these compartments and then transported to postsynaptic sites. Notably, only a subset of cortical principal neurons showed prominent somatic Shank3 IR based on microscopical observations. Thus, one may speculate that Shank3 is produced among cortical glutamatergic neurons in a use-dependent manner.

### Shank3 Is Enriched in Forebrain Subregions Related to Cognitive Dysfunctions in Neuropsychiatric Disorders

As briefly noted in the Introduction, *SHANK3* alterations are risk factors for several neuropsychiatric diseases. Various lines of genetically modified mice and, more recently, rats and macaques have been generated to understand the underlying pathogenic cellular, molecular, and neurocircuitry mechanisms (Bozdagi et al., 2010; Peça et al., 2011; Wang et al., 2011; Kouser et al., 2013; Lee et al., 2015; Jaramillo et al., 2016; Wang et al., 2016, 2017; Copping et al., 2017; Dhamne et al., 2017; Vicidomini et al., 2017; Drapeau et al., 2018; Kabitzke et al., 2018; Yoo et al., 2018; Guo et al., 2019; Zhou et al., 2019; Wan et al., 2021). These animal models display many neurobehavioral phenotypes ruminant to human symptoms in neuropsychiatric disorders, such as repetitive/compulsive behavior, abnormal social communication and interaction, impaired learning and memory, reduced locomotion activity, and increased anxiety-like behavior. Some studies show structural and functional defects of the excitatory synapses, in particular forebrain regions, such as the medial prefrontal cortex (Drapeau et al., 2018; Yoo et al., 2018, 2019), anterior cingulate cortex (Guo et al., 2019), hippocampal formation (Kouser et al., 2013; Jaramillo et al., 2017), and striatum (Mei et al., 2016; Wang et al., 2016; Jaramillo et al., 2017).

The present human brain mapping study demonstrates that Shank3 expression is enriched in the forebrain subregions in general. Thus, fairly intense Shanks IR was present in the neocortex of all cerebral lobes, with layers II-IV exhibited a relatively heavier stain than V and VI in most functional regions. In the primary visual cortex, layer IVb showed stronger IR than IVa and IVc. There was also prominent labeling across the hippocampal formation, localized to the somata and dendritic profiles of the subicular/hippocampal pyramidal neurons and the dentate granule cells. Large multiple neurons likely representing mossy cells were labeled in the hilus of the DG, with clusters of thorny excrescences identifiable in this region. In the amygdala, Shank3 IR was distinct in large-sized multipolar neurons as well as in the neuropil in most subregions of this complex. In addition, the neostriatum and thalamus exhibited more impressive Shank3 IR relative to other subcortical structures. Thus, Shank3 is highly expressed in brain regions important for cognitive processing, learning and memory, mode regulation, and locomotor programming, which are the brain functions often impaired among major neuropsychiatric disorders.

### Shank3 Expression Is Reduced in Aged and AD Human Brains

Cerebral hypometabolism occurs during brain aging and presents more aggressively during AD pathogenesis (Struble et al., 2010). Decreased brain activity, as measured by positron emission tomography (PET) or functional magnetic resonance imaging (fMRI), is associated with mild cognitive impairment and AD-type dementia (Ruitenberg et al., 2005; Bauckneht et al., 2018; Sala et al., 2020). While Aβ accumulation and tauopathy are related to cerebral metabolic changes (Adams et al., 2019; Carbonell et al., 2020), studies also show metabolic decline in the brain of aged rodents that do not develop senile plaques and tangles (Gage et al., 1984; Tack et al., 1989). Synaptic loss has been considered an early change during brain aging and in the progress of AD (Serrano-Pozo et al., 2011; Cardozo et al., 2019; Chen et al., 2019; Galasko et al., 2019; Lleó et al., 2019; Colom-Cadena et al., 2020; Mecca et al., 2020). Importantly, synaptic activity and brain metabolism are strongly correlated, and the severity of dementia correlates better with the extent of synaptic loss and metabolic deficit than does Aβ deposition (Scheff et al., 1990; Masliah et al., 1991; Terry et al., 1991; Scheff and Price, 2001). Neurobiologically, glucose use and mitochondrial activity are required for axon depolarization and repolarization, transmitter release and uptake, postsynaptic activity, and other events of synaptic transmission (Struble et al., 2010). Specifically, brain metabolic activity is stoichiometrically coupled with glutamatergic neurotransmission (Sibson et al., 1998).

Here, we, extend morphological and biochemical evidence forearly-onset decline and pattern alteration of Shank3 expression in the human brain with the advance of age and the development of AD-type neuropathologies. Thus, microscopically, we could recognize a decrease of Shanks-expressing neuronal somata in the cerebral neocortex in the aged relative to youth and adult cases. The distribution of Shank3 IR in the neuropil over the cortical layers became less differential in aged cases with cerebral Aβ and pTau lesions. A similar pattern change was visible in the cerebellar cortex in the aged cases, involving reduced Shank3 IR in the granule cell layer. In addition, Shank3 IR over the dentate ML was progressively reduced and disrupted along with the increase of Aβ and pTau pathologies in the hippocampal formation. Moreover, we confirmed the trend of decline in the density of Shank3 IR in the prefrontal, postcentral, and temporal neocortices, the molecular layer of the hippocampal dentate gyrus, and the molecular and granule cell layers of the cerebellar cortex in the adult to the aged group, and, furthermore, to the AD group, relative to the youth group. In addition, using the immunoblot method, a significant decline in the levels of Shank3 protein was detected in the prefrontal and primary motor neocortex as well as the cerebellar cortex in the adult and aged cases in comparison to the youth cases. The levels of PSD95 and synaptophysin in the assayed regions were also reduced largely in a parallel manner. These findings are consistent with similar observations reported in early as well as recent studies, strengthening the importance of synaptic pathology in age- and AD-related cognitive decline and dementia (Scheff et al., 1990; Masliah et al., 1991; Terry et al., 1991; Scheff and Price, 2001; Serrano-Pozo et al., 2011; Cardozo et al., 2019; Chen et al., 2019; Galasko et al., 2019; Lleó et al., 2019; Colom-Cadena et al., 2020; Mecca et al., 2020). A potential pitfall of this study is that we did not detect a significant difference between the aged and AD groups in Shank3 protein levels by immunoblotting of neocortical and cerebellar cortical lysates, although the densitometry of immunolabeling in brain sections indicated such a difference. Future study is required to determine a further decline of Shank3 protein levels in AD relative to aged individuals by including more cases, precisive tissue sampling, and measures to control antemortem and postmortem conditions that could affect protein expression and preservation.

In summary, the present study shows that Shank3 is localized to putative excitatory postsynaptic sites and neuronal subpopulations as assessed in the brains of youth and adult individuals. It is immunolabeling in neuronal somata, and neuropil is reduced in the brains of the elderly, along with an overall decline in protein levels. Shank3 expression in the dentate molecular layer is lost and disrupted along with the development of Aβ and pTau in the hippocampal formation. Our findings implicate that Shank3 may be important in regulating excitatory neurotransmission in forebrain regions in supporting high cognitive functions. Shank3 expression is reduced in aged and AD human brains along with a general trend of synaptic loss, which might be related to impaired glutamatergic neurotransmission, cerebral hypometabolism, and cognitive deficits.

## Data Availability Statement

The original contributions presented in the study are included in the article/Supplementary Material, further inquiries can be directed to the corresponding author/s.

## Ethics Statement

The studies involving human participants were reviewed and approved by use of postmodern human brains was approved by the Ethics Committee for Research and Education at Xiangya School of Medicine, in compliance with the Code of Ethics of the World Medical Association (Declaration of Helsinki). Written informed consent to participate in this study was provided by the participants’ legal guardian/next of kin.

## Author Contributions

LW, J-QA, CY, JJ, Q-LZ, R-JH, and TT: brain banking, tissue processing, immunohistochemistry, and data collection. ET and X-XY: neuropathological evaluation. LW and Z-HL: western blot. ET, BX, and X-XY: funding acquisition and experimental design. LW and X-XY: manuscript composition. JM reviewed and edited the manuscript. All authors contributed to the article and approved the submitted version.

## Conflict of Interest

The authors declare that the research was conducted in the absence of any commercial or financial relationships that could be construed as a potential conflict of interest.

## Publisher’s Note

All claims expressed in this article are solely those of the authors and do not necessarily represent those of their affiliated organizations, or those of the publisher, the editors and the reviewers. Any product that may be evaluated in this article, or claim that may be made by its manufacturer, is not guaranteed or endorsed by the publisher.
