# Supplementary figures and images for: Expression of the Excitatory Postsynaptic Scaffolding Protein, Shank3, in Human Brain: Effect of Age and Alzheimer’s Disease (part 2 of 3)
Source: Front Aging Neurosci. 2021 Aug 24;13:717263. doi: 10.3389/fnagi.2021.717263 (PMC8421777; doi:10.3389/fnagi.2021.717263)

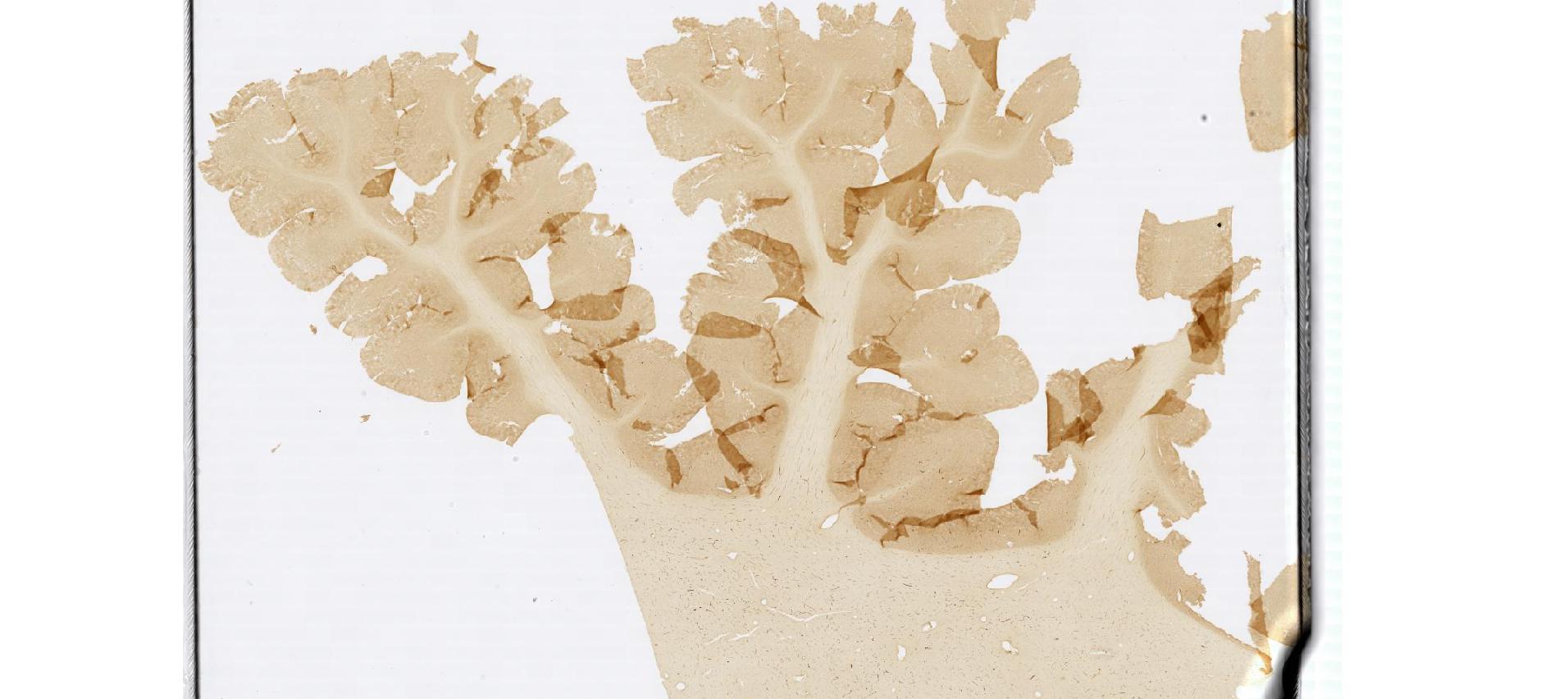

Supplement: Supplementary file 1 [file Presentation_1.ZIP › shank3-immunohistochemistry/case-24/cerebellum.jpg]

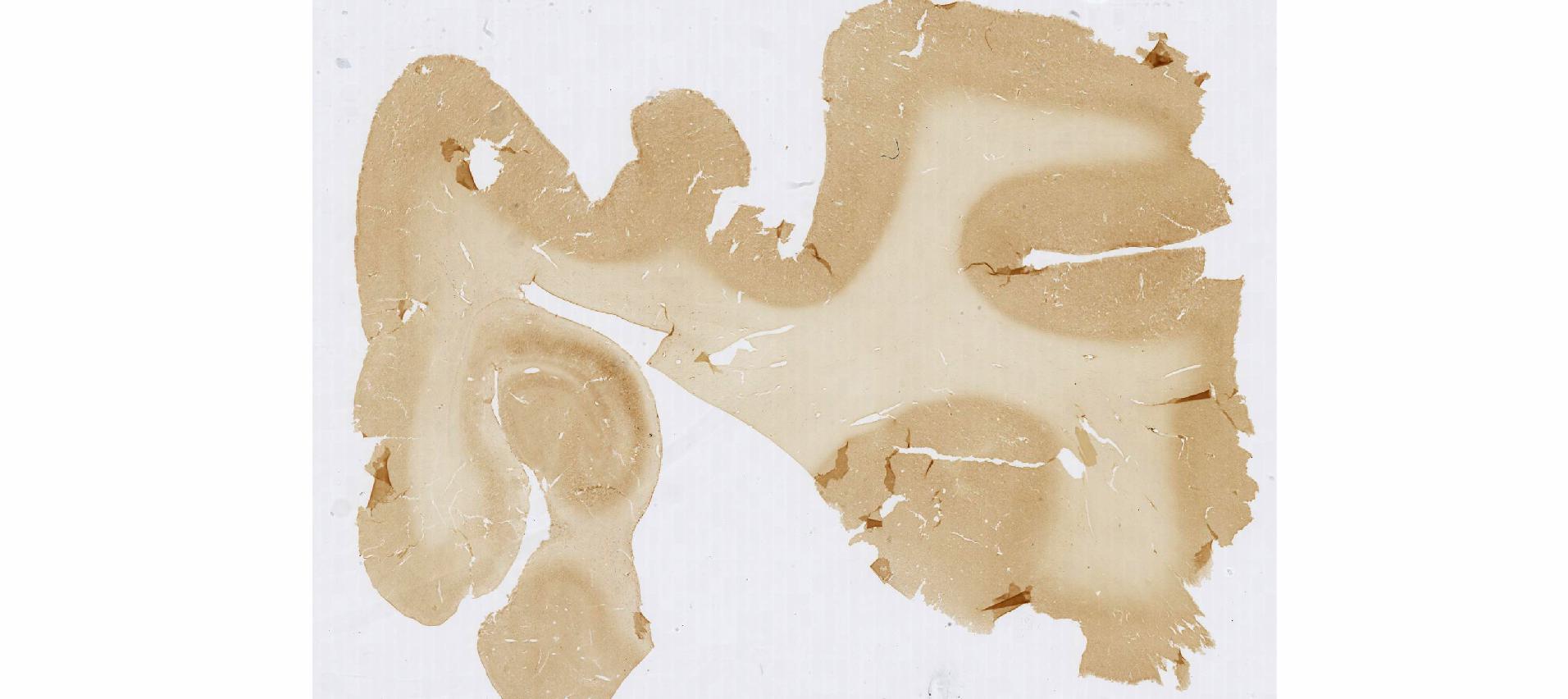

Supplement: Supplementary file 1 [file Presentation_1.ZIP › shank3-immunohistochemistry/case-24/hippocampal formation.jpg]

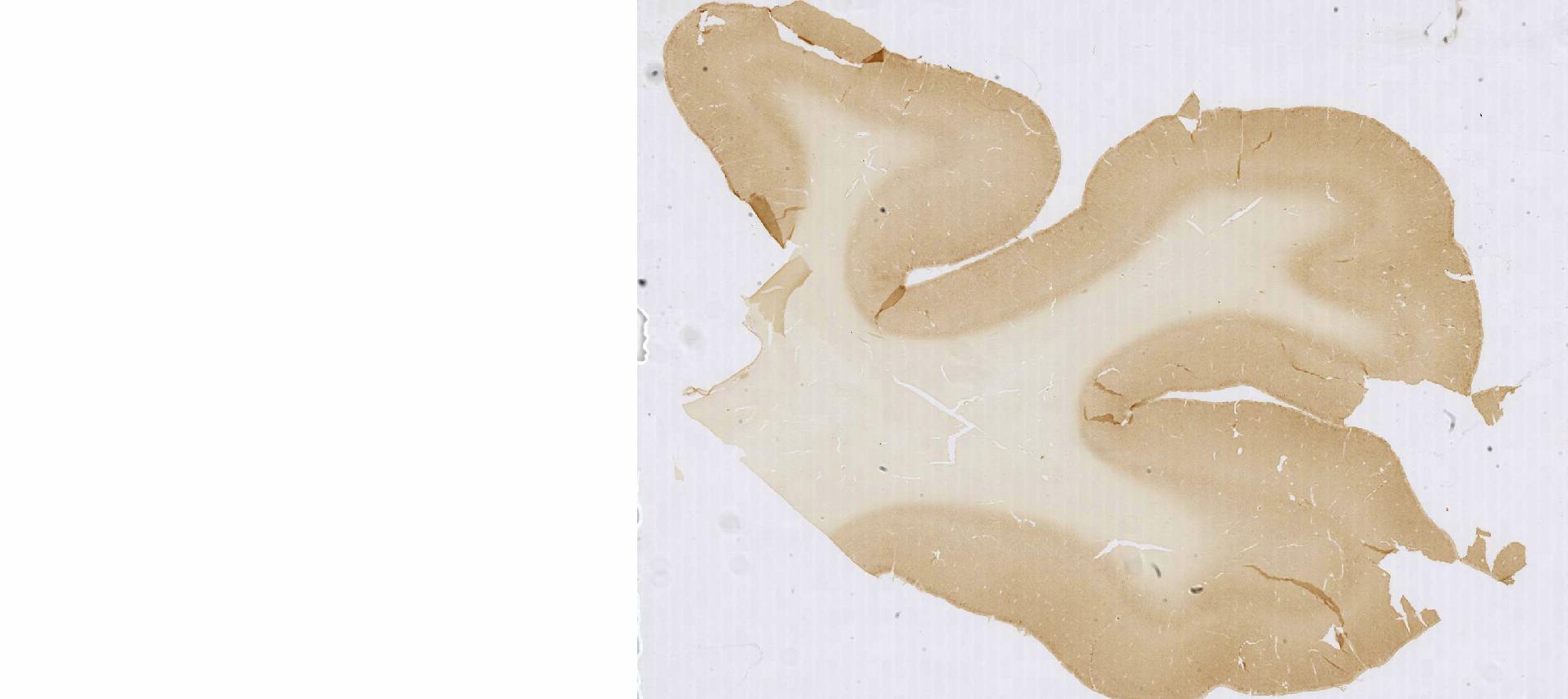

Supplement: Supplementary file 1 [file Presentation_1.ZIP › shank3-immunohistochemistry/case-24/prefrontal cortex.jpg]

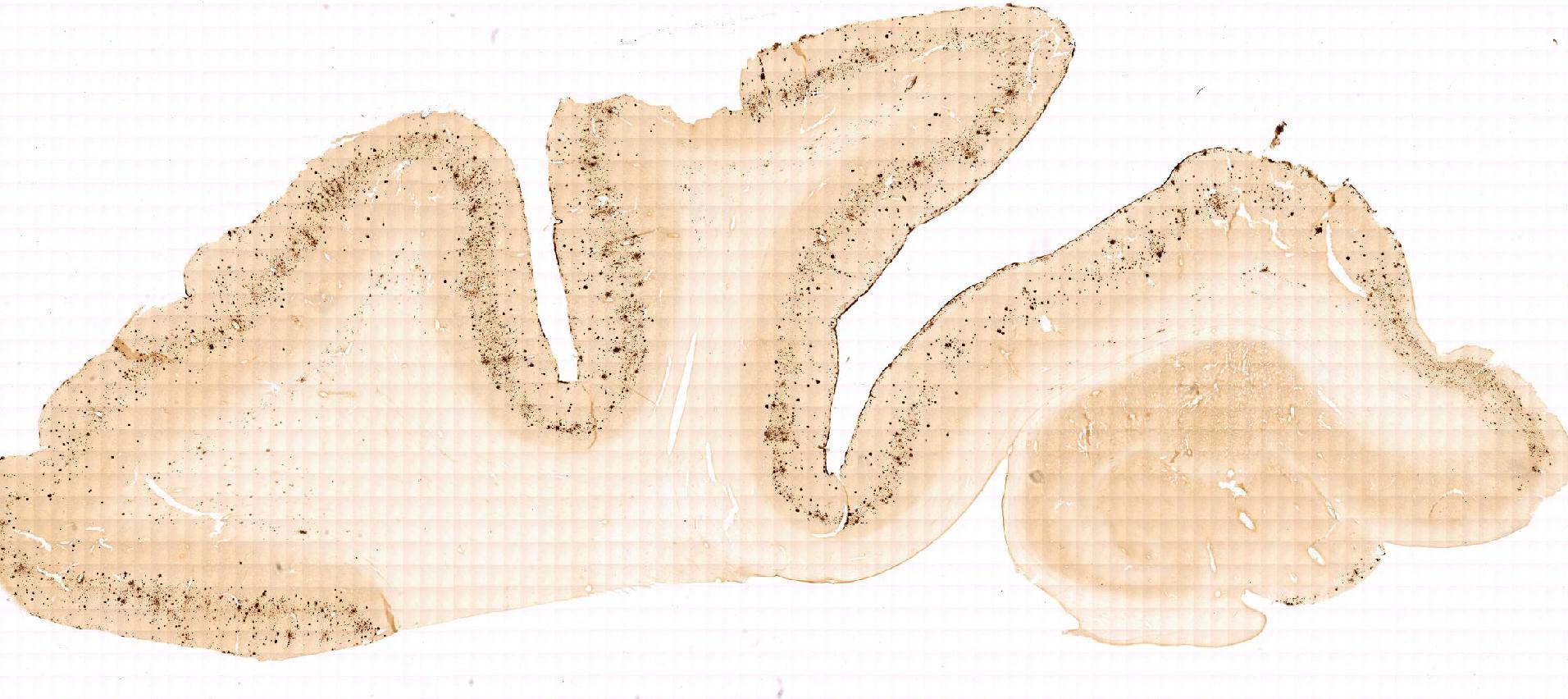

Supplement: Supplementary file 1 [file Presentation_1.ZIP › shank3-immunohistochemistry/case-25/HP-6E10.jpg]

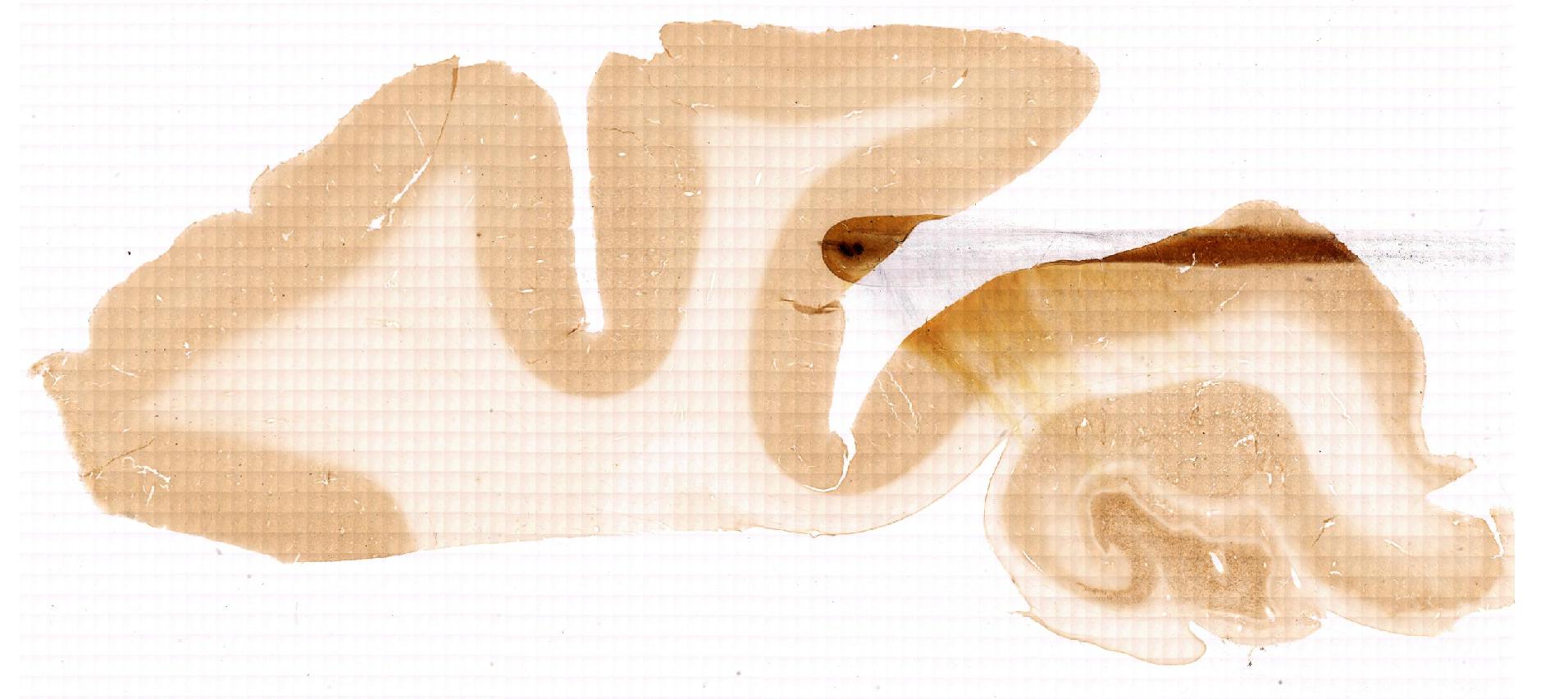

Supplement: Supplementary file 1 [file Presentation_1.ZIP › shank3-immunohistochemistry/case-25/HP-bace1.jpg]

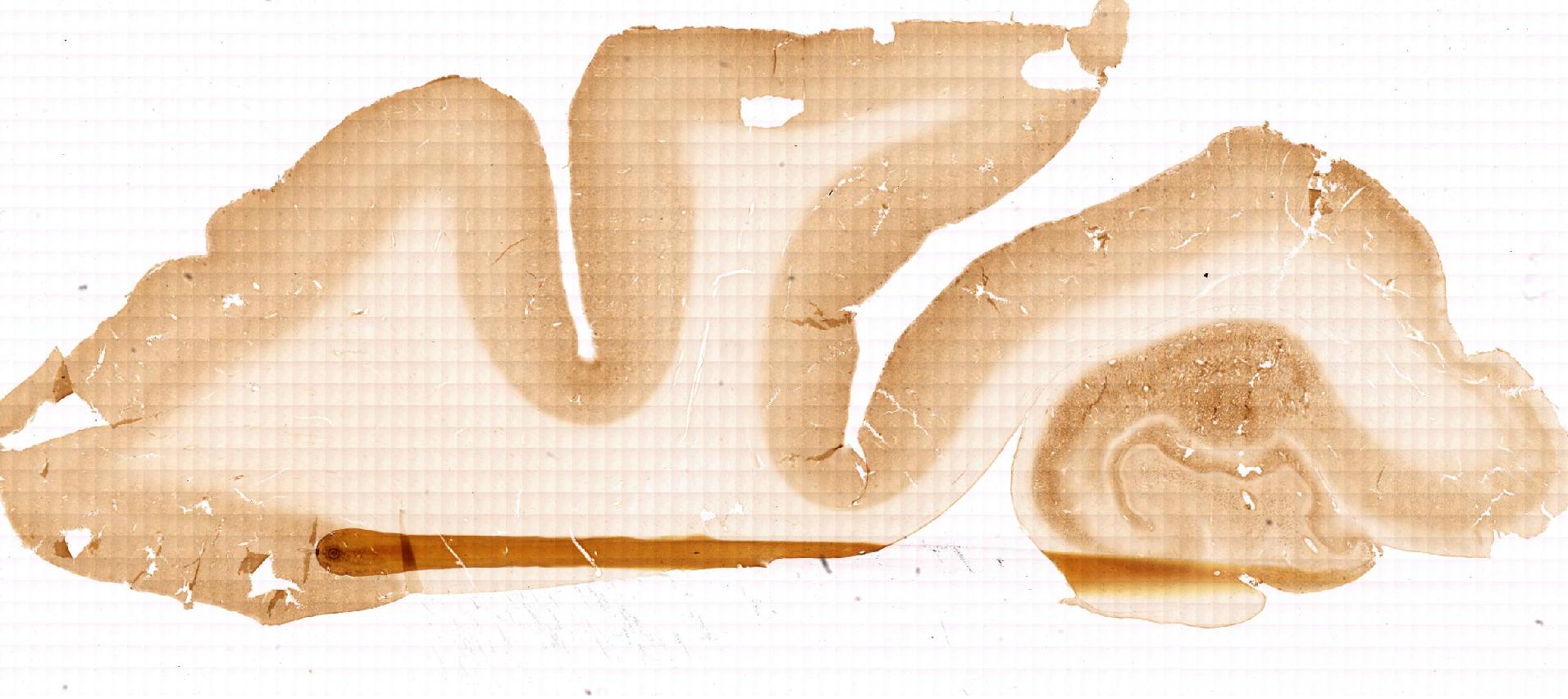

Supplement: Supplementary file 1 [file Presentation_1.ZIP › shank3-immunohistochemistry/case-25/HP-shank3.jpg]

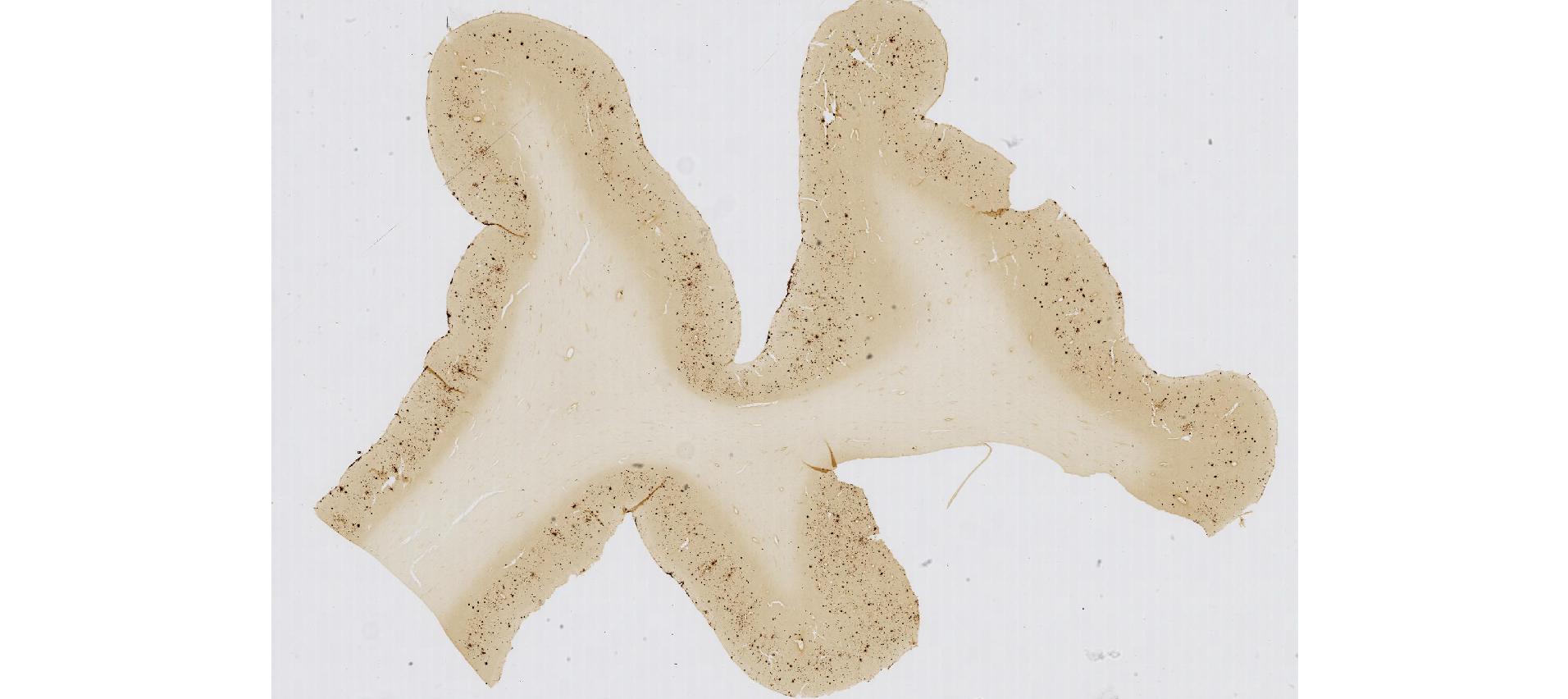

Supplement: Supplementary file 1 [file Presentation_1.ZIP › shank3-immunohistochemistry/case-25/PFC-6E10.jpg]

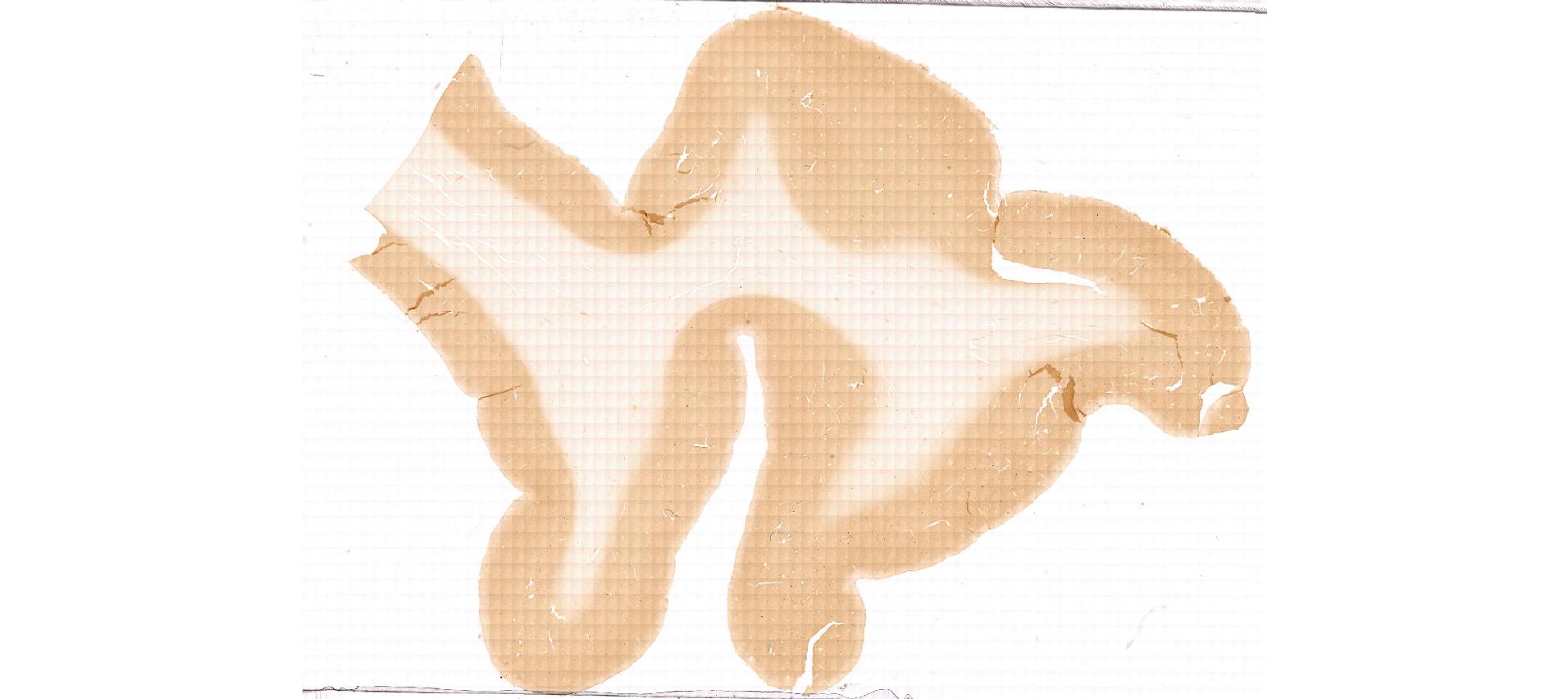

Supplement: Supplementary file 1 [file Presentation_1.ZIP › shank3-immunohistochemistry/case-25/PFC-bace1.jpg]

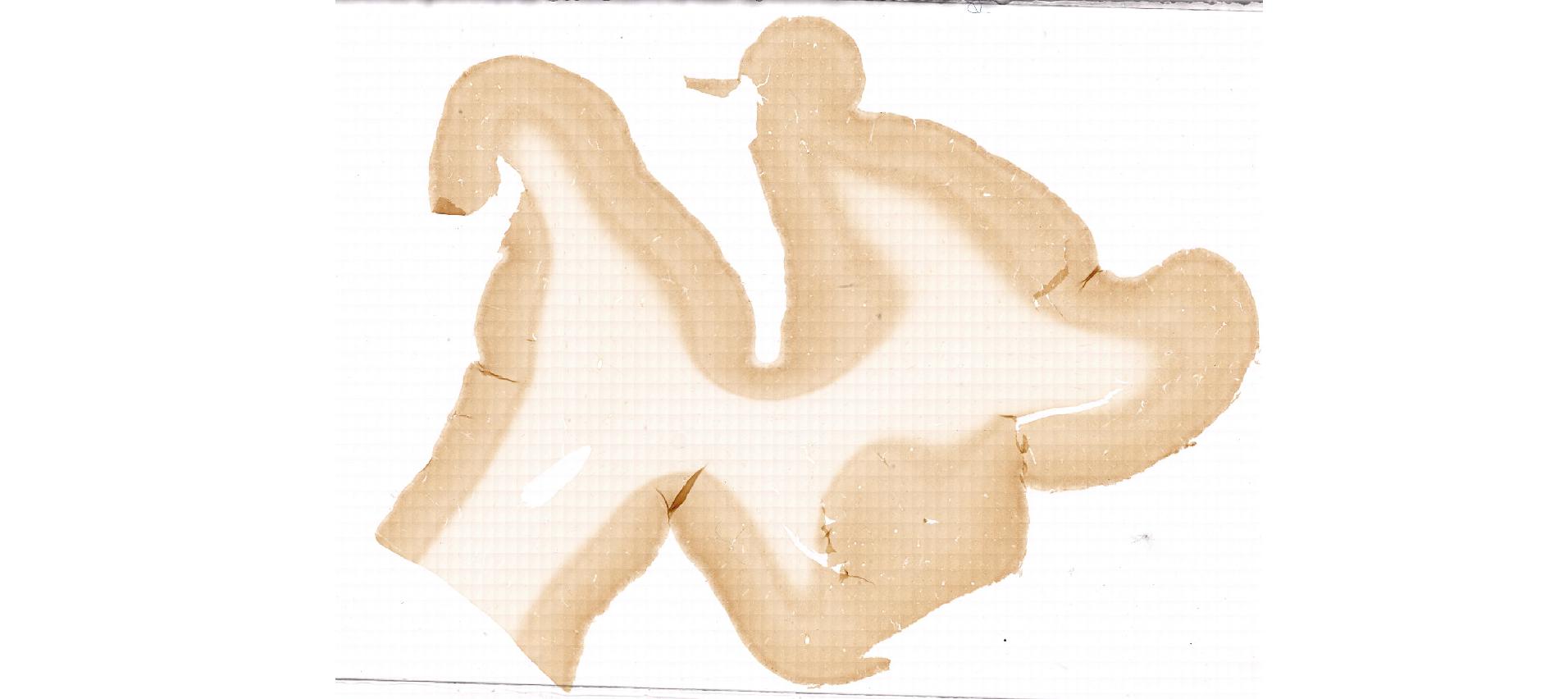

Supplement: Supplementary file 1 [file Presentation_1.ZIP › shank3-immunohistochemistry/case-25/PFC-shank3.jpg]

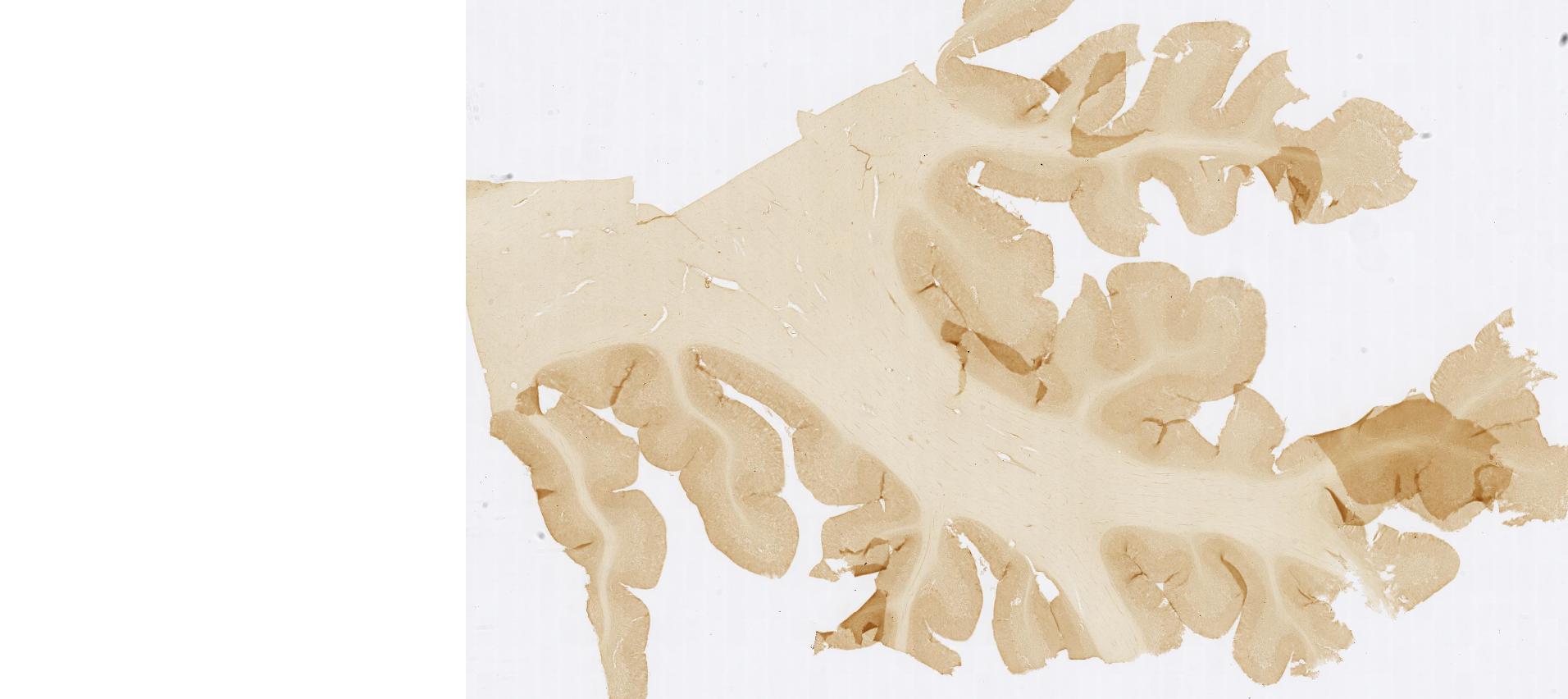

Supplement: Supplementary file 1 [file Presentation_1.ZIP › shank3-immunohistochemistry/case-25/cerebellum-shank3.jpg]

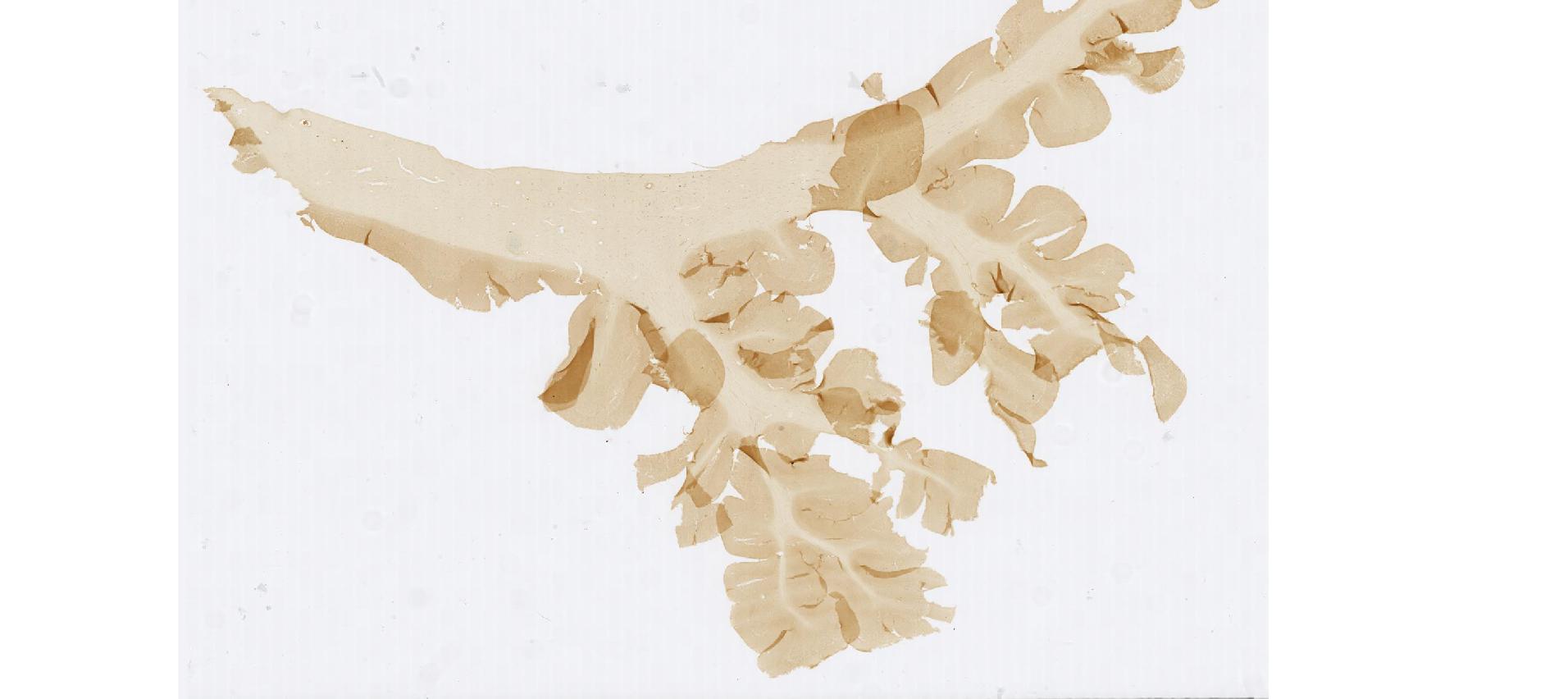

Supplement: Supplementary file 1 [file Presentation_1.ZIP › shank3-immunohistochemistry/case-26/cerebellum.jpg]

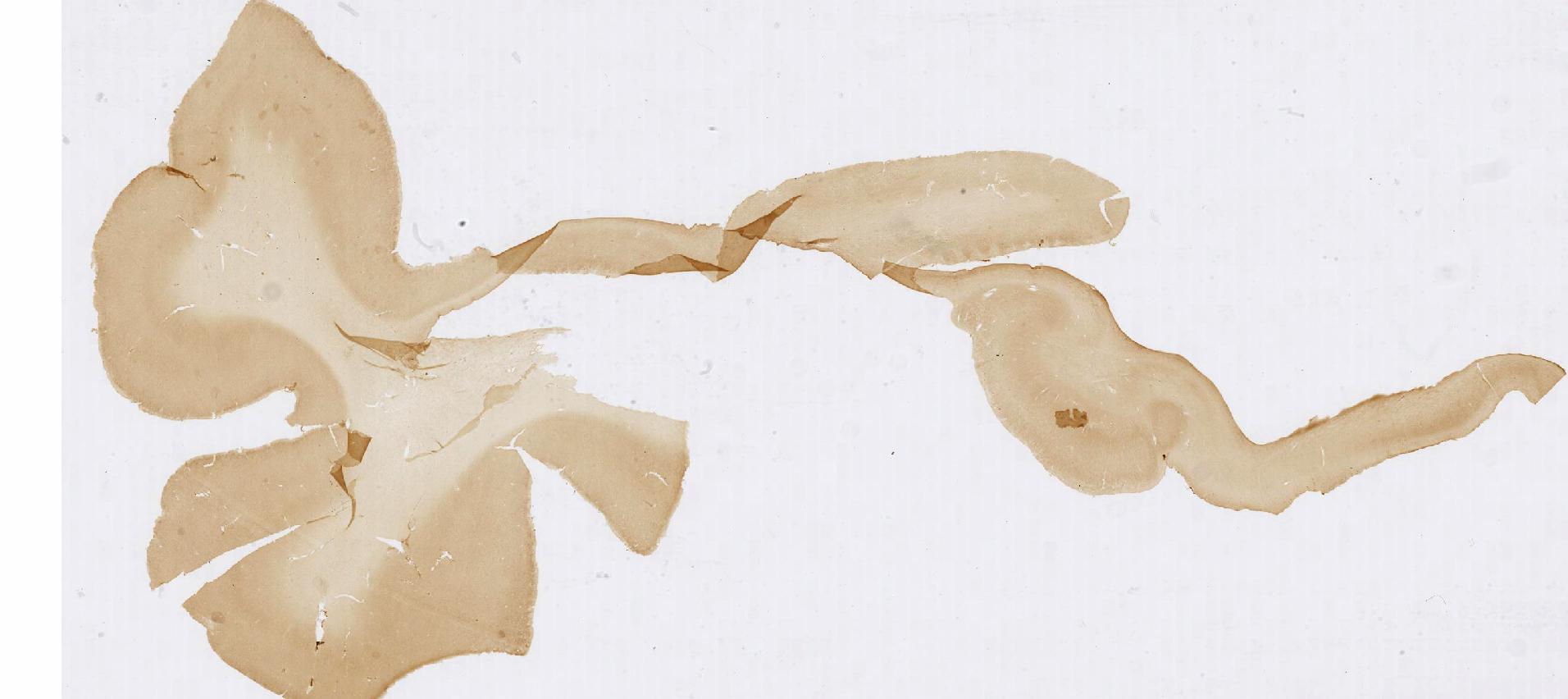

Supplement: Supplementary file 1 [file Presentation_1.ZIP › shank3-immunohistochemistry/case-26/hippocampal formation.jpg]

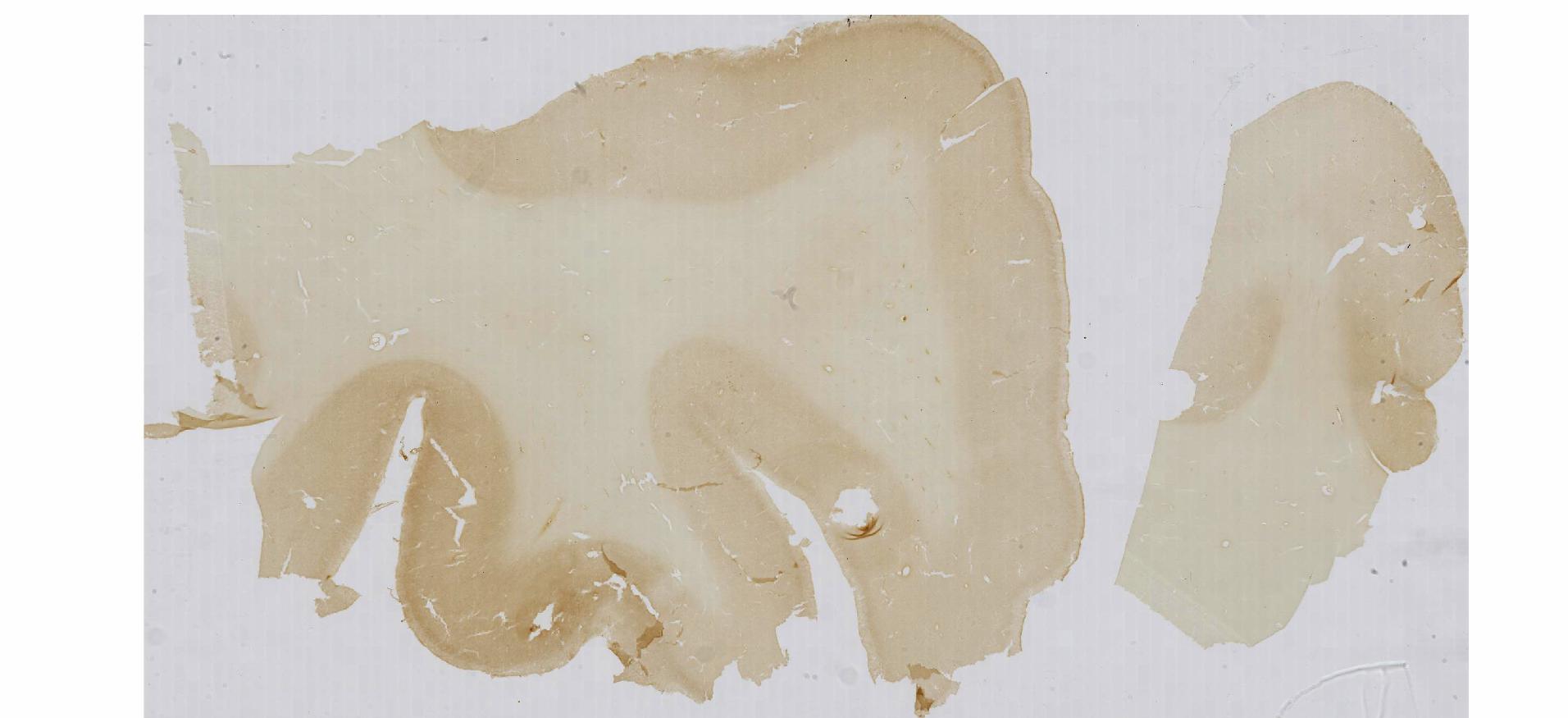

Supplement: Supplementary file 1 [file Presentation_1.ZIP › shank3-immunohistochemistry/case-26/prefrontal cortex -2.jpg]

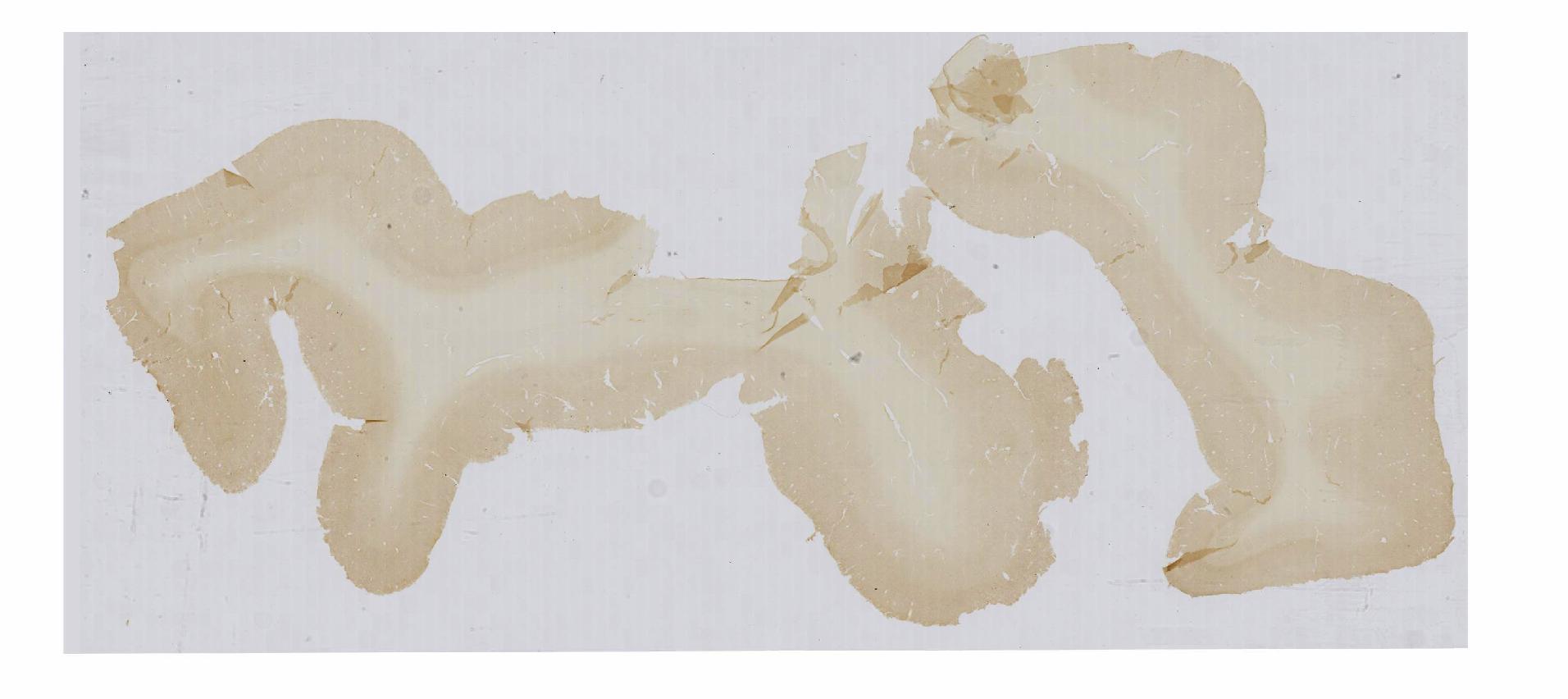

Supplement: Supplementary file 1 [file Presentation_1.ZIP › shank3-immunohistochemistry/case-26/prefrontal cortex-1.jpg]

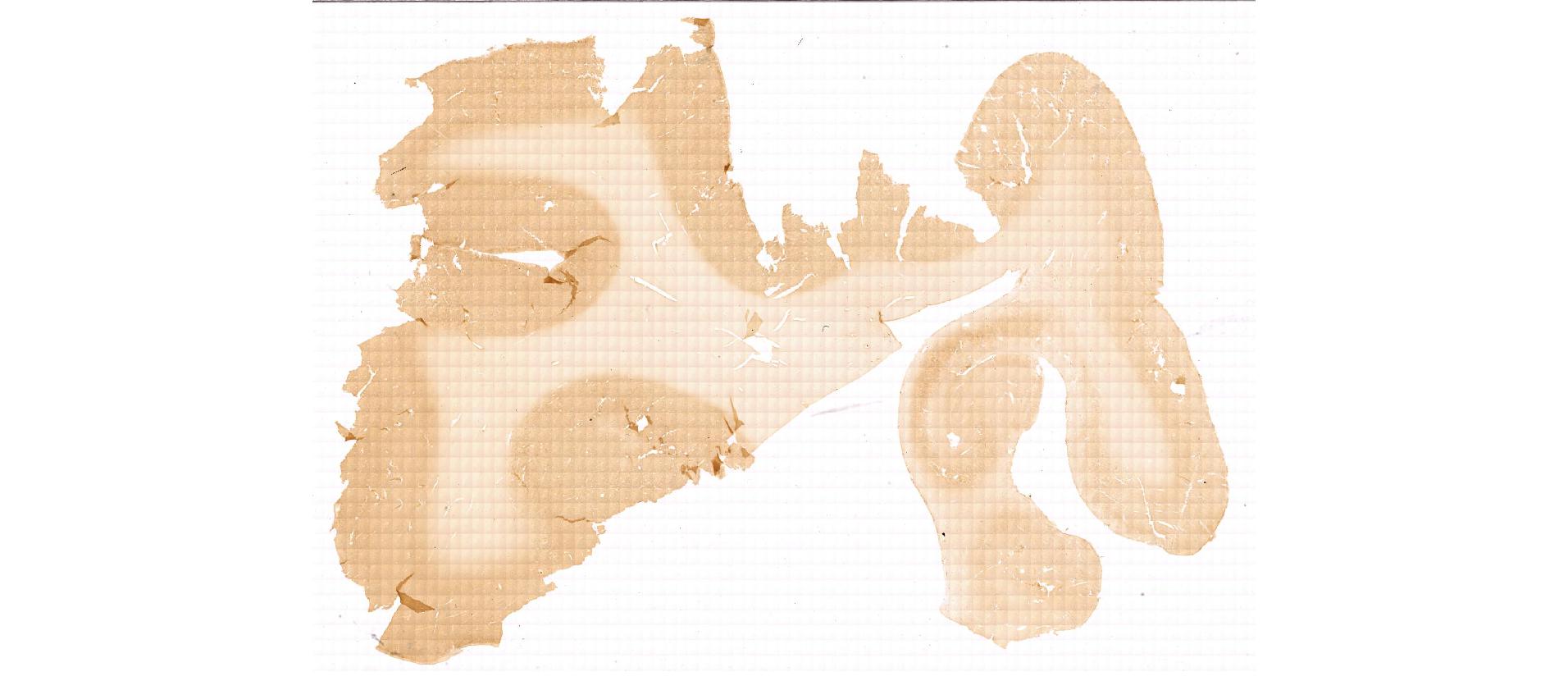

Supplement: Supplementary file 1 [file Presentation_1.ZIP › shank3-immunohistochemistry/case-27/HP -shank3.jpg]

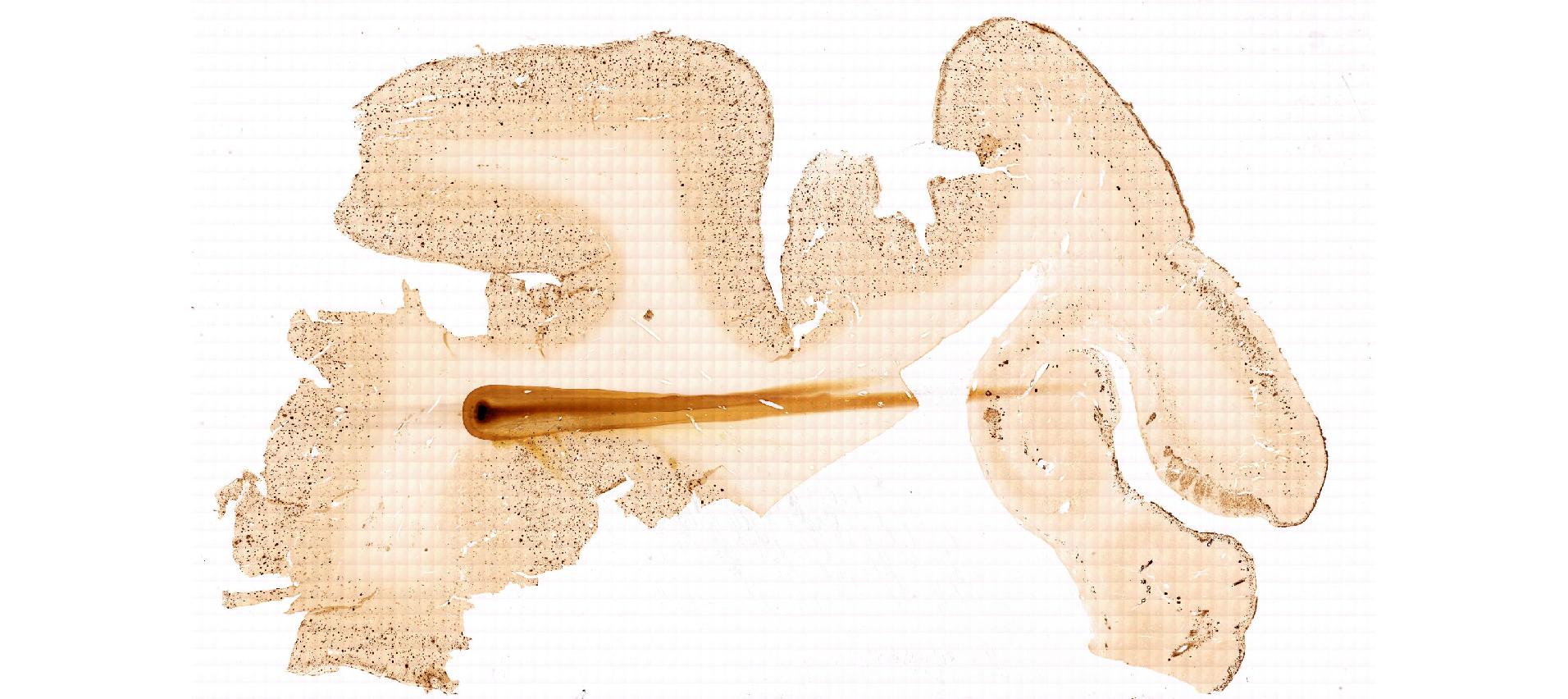

Supplement: Supplementary file 1 [file Presentation_1.ZIP › shank3-immunohistochemistry/case-27/HP-6E10.jpg]

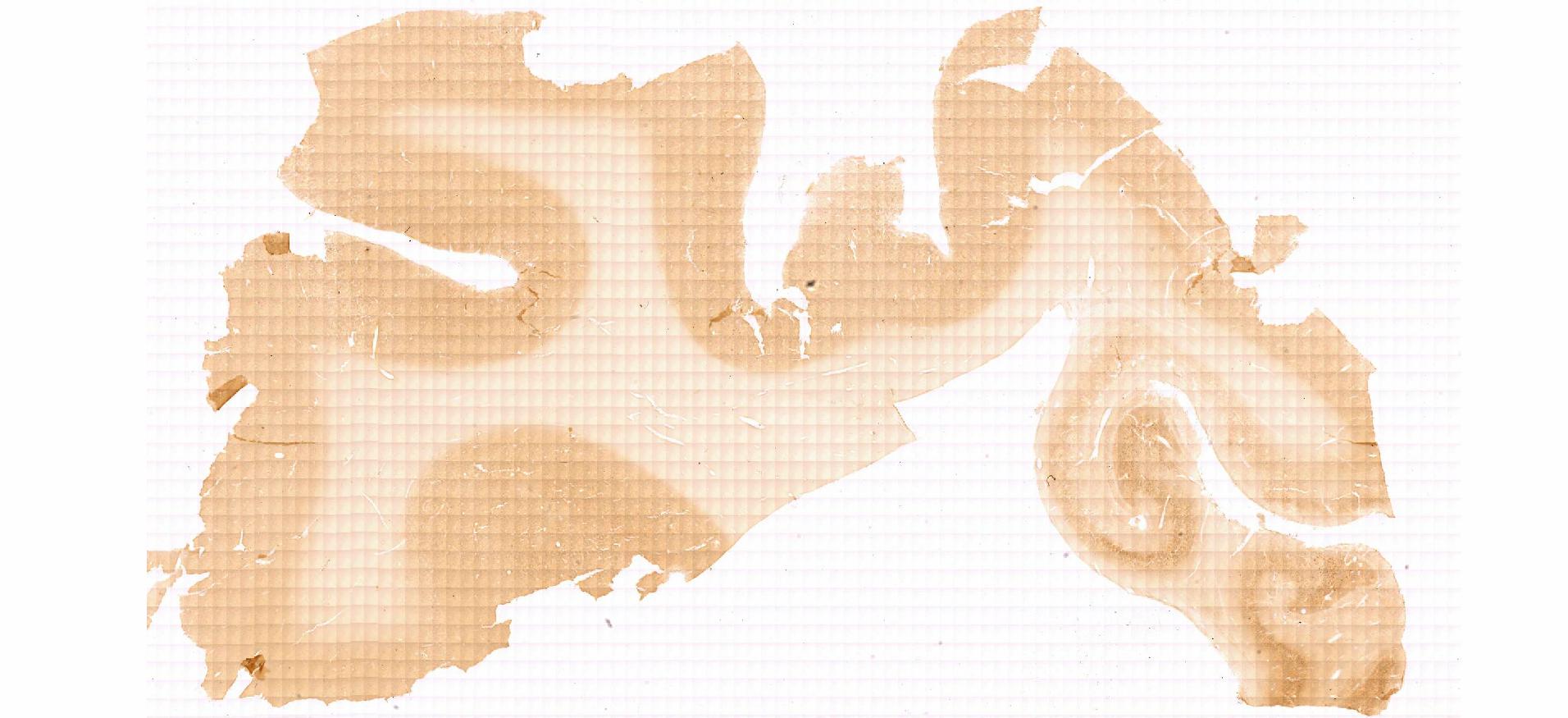

Supplement: Supplementary file 1 [file Presentation_1.ZIP › shank3-immunohistochemistry/case-27/HP-bace1.jpg]

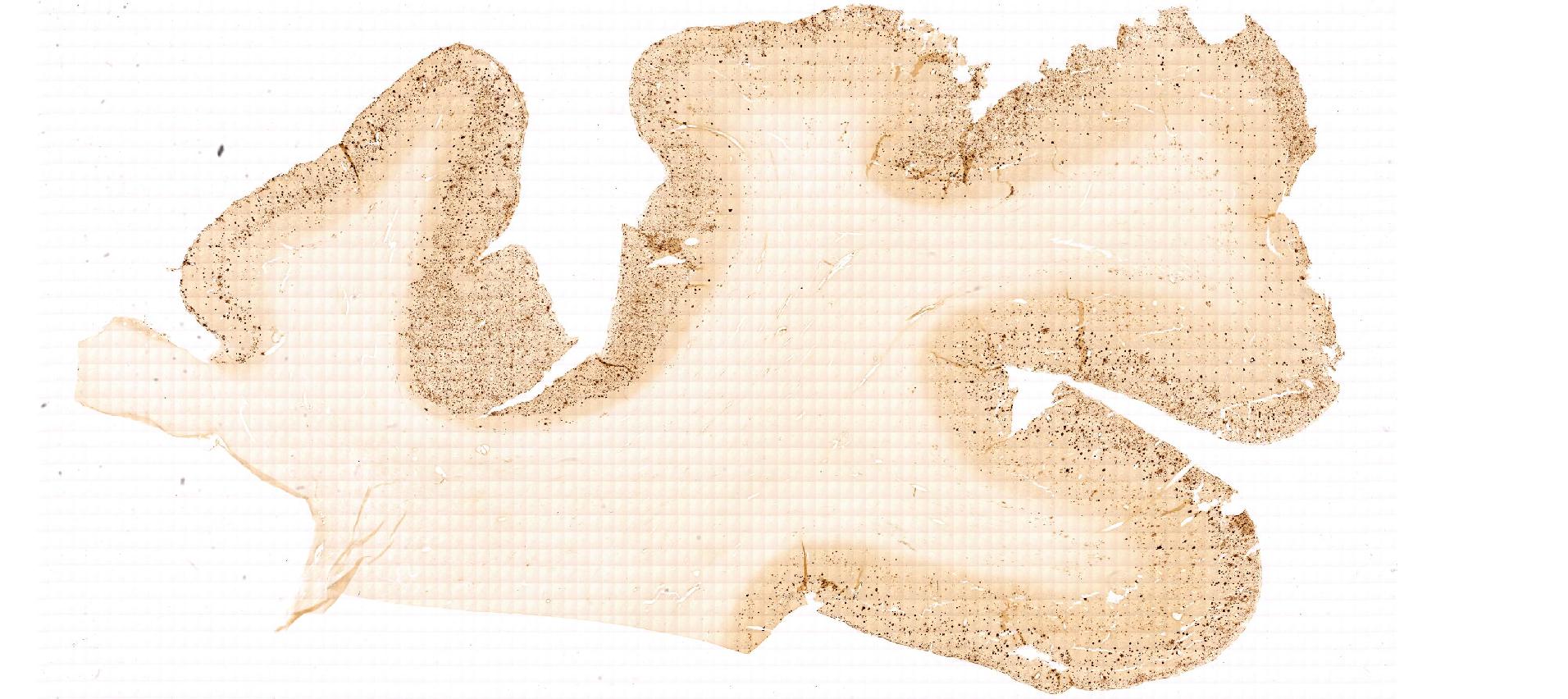

Supplement: Supplementary file 1 [file Presentation_1.ZIP › shank3-immunohistochemistry/case-27/PFC-6E10.jpg]

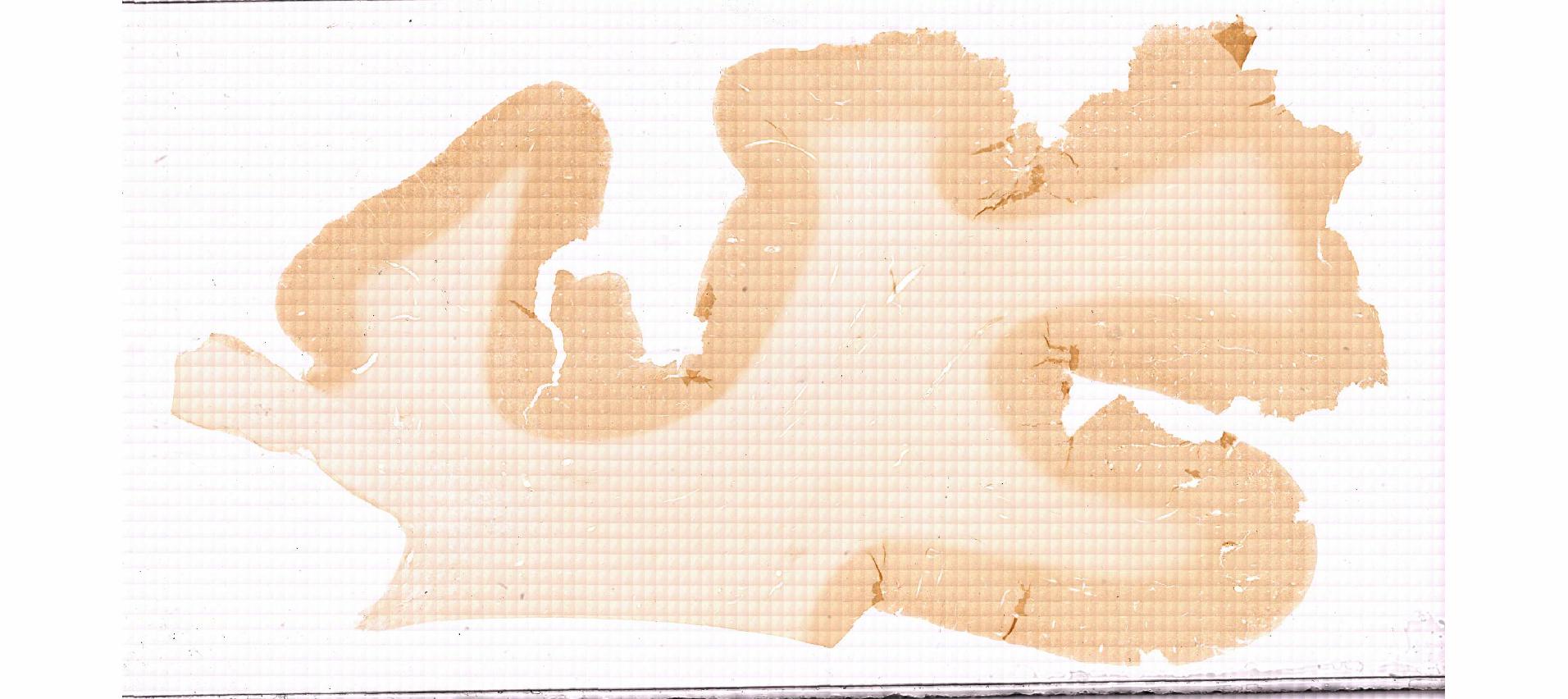

Supplement: Supplementary file 1 [file Presentation_1.ZIP › shank3-immunohistochemistry/case-27/PFC-bace1.jpg]

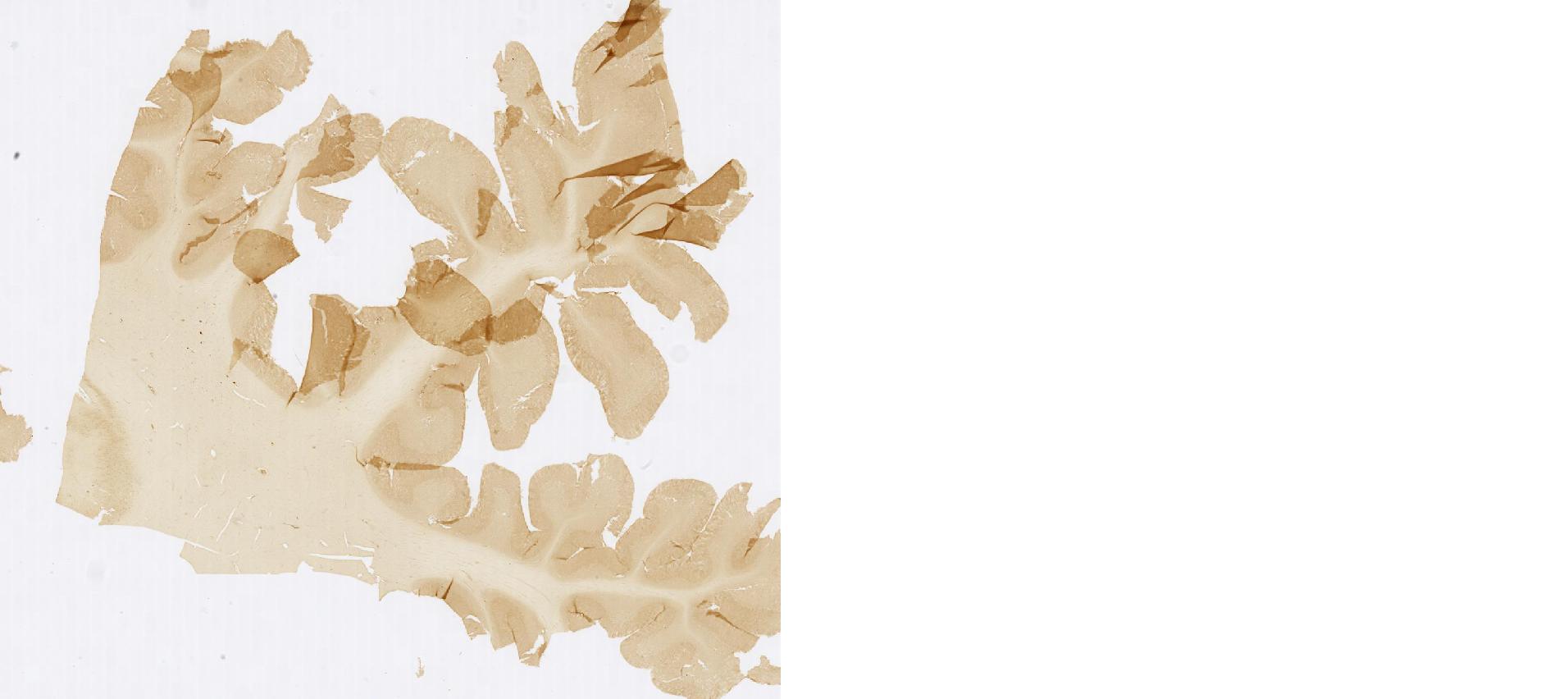

Supplement: Supplementary file 1 [file Presentation_1.ZIP › shank3-immunohistochemistry/case-27/cerebellum-shank3.jpg]

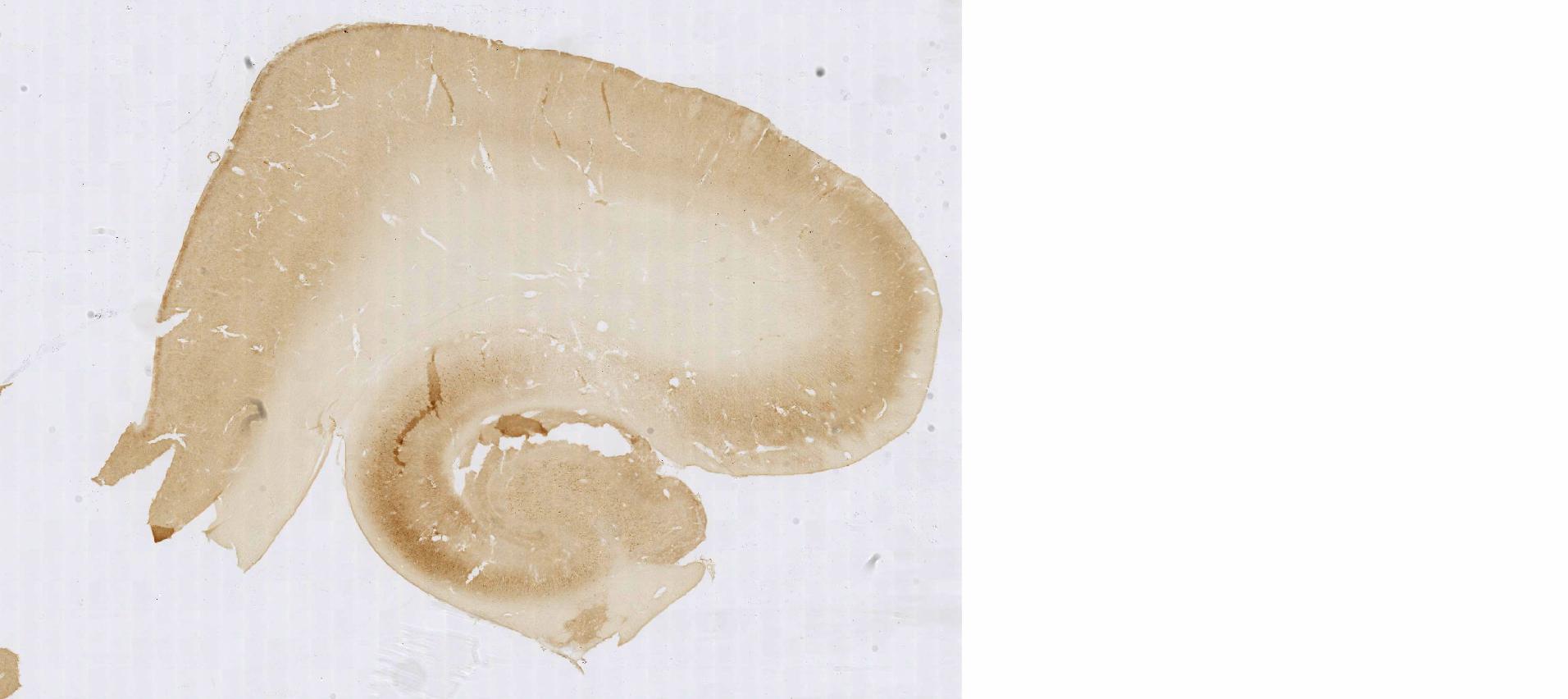

Supplement: Supplementary file 1 [file Presentation_1.ZIP › shank3-immunohistochemistry/case-27/hippocampal formation-1-shank3.jpg]

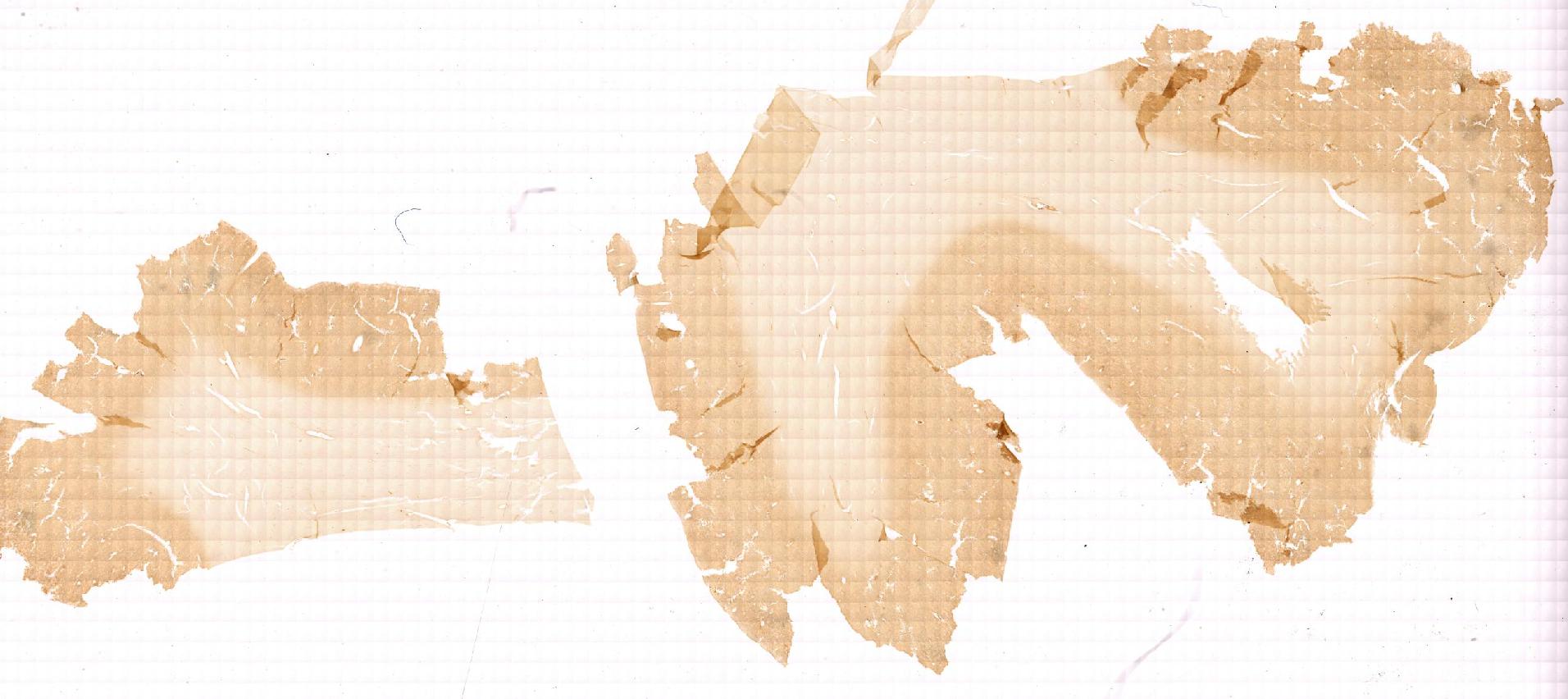

Supplement: Supplementary file 1 [file Presentation_1.ZIP › shank3-immunohistochemistry/case-27/prefrontal cortex (PFC) -shank3.jpg]

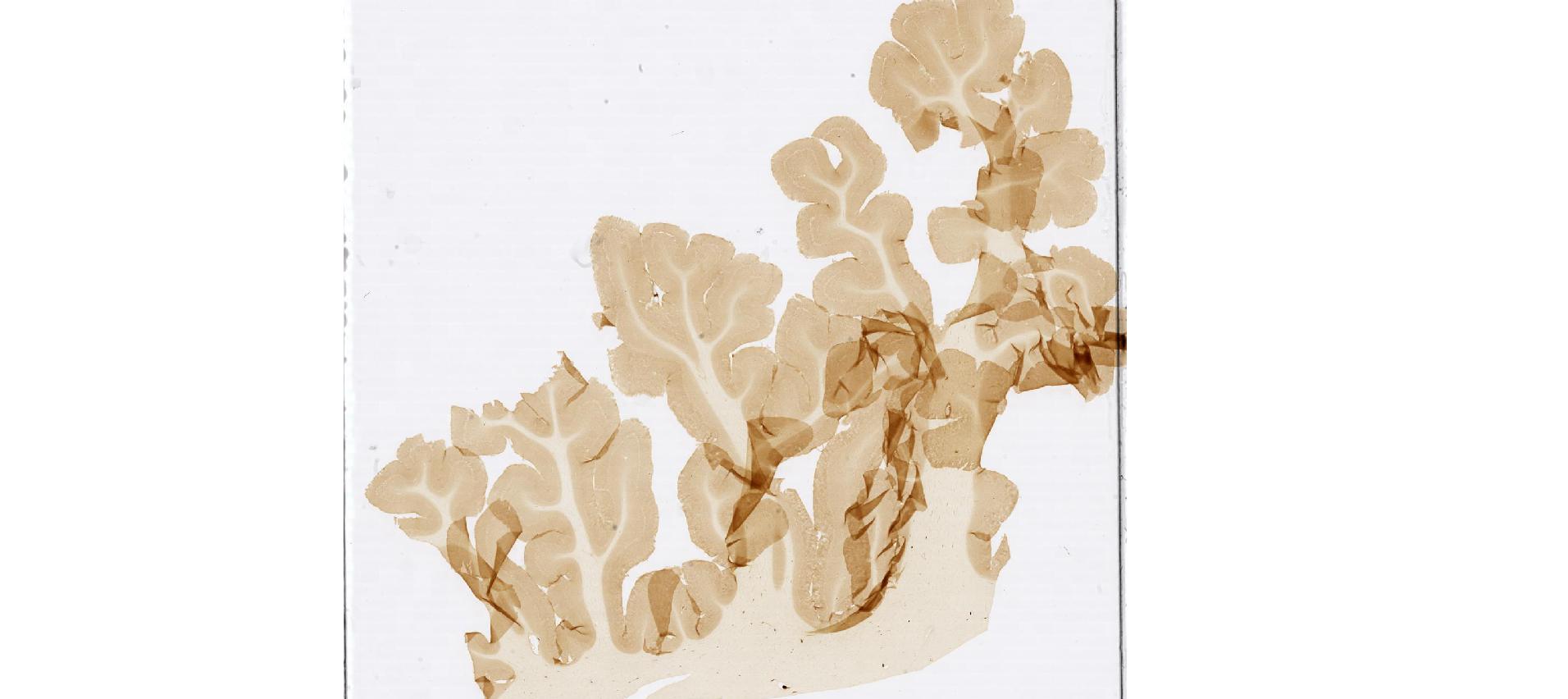

Supplement: Supplementary file 1 [file Presentation_1.ZIP › shank3-immunohistochemistry/case-28/cerebellum.jpg]

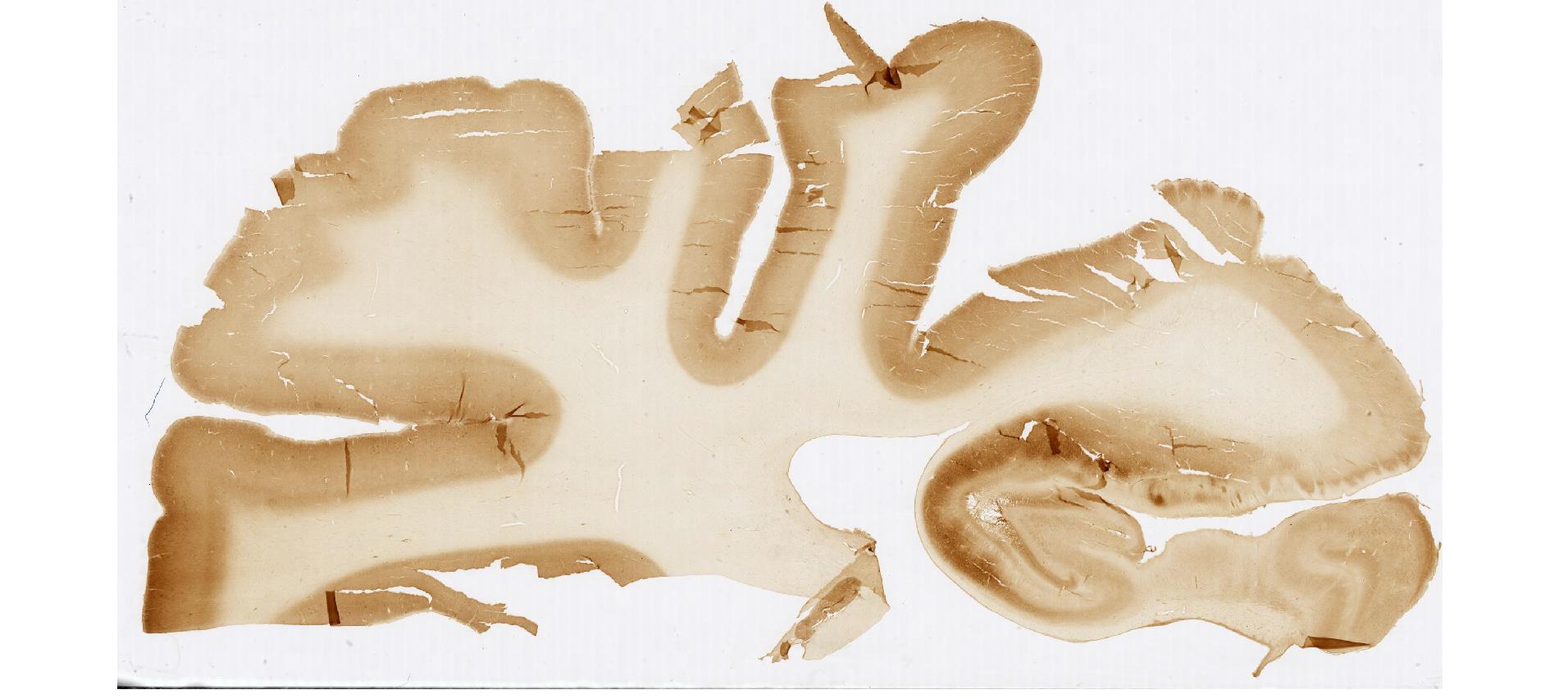

Supplement: Supplementary file 1 [file Presentation_1.ZIP › shank3-immunohistochemistry/case-28/hippocampal formation.jpg]

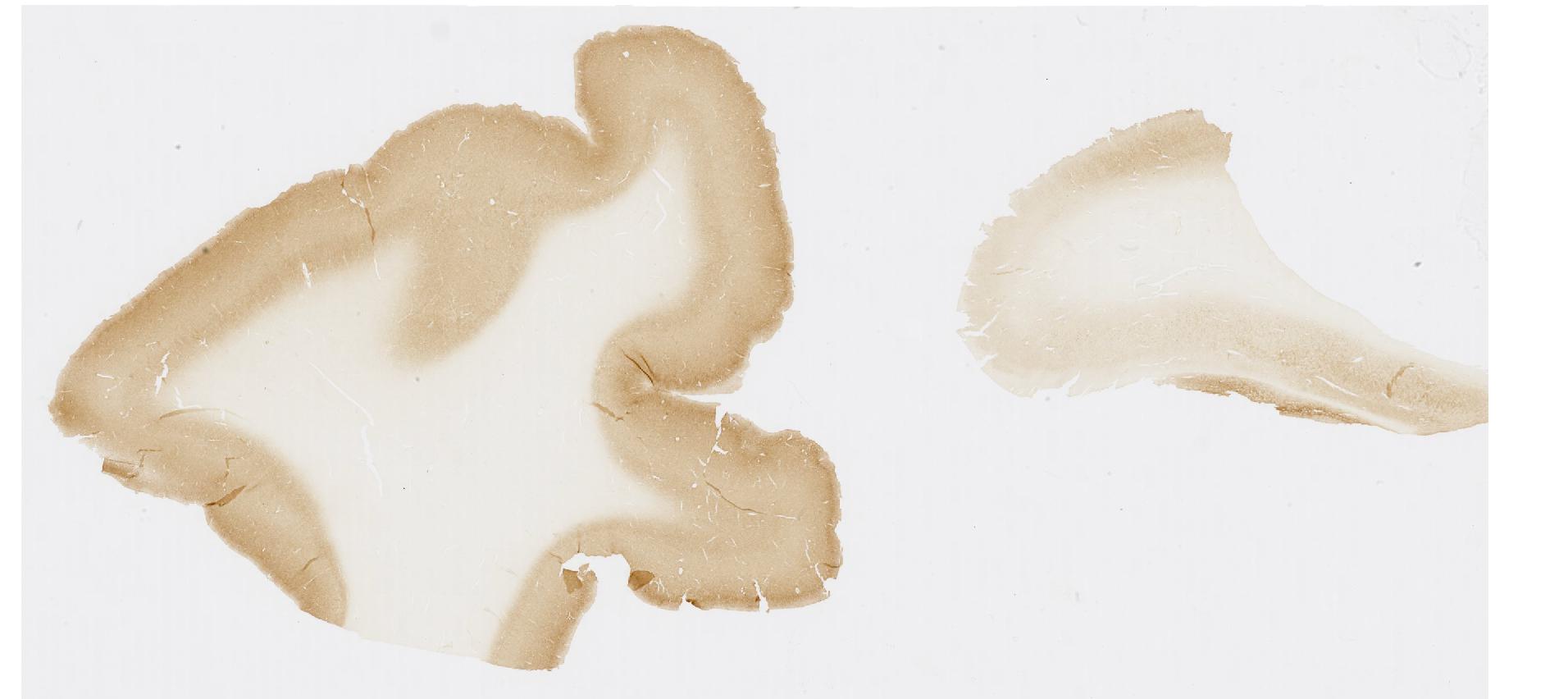

Supplement: Supplementary file 1 [file Presentation_1.ZIP › shank3-immunohistochemistry/case-28/prefrontal cortex.jpg]

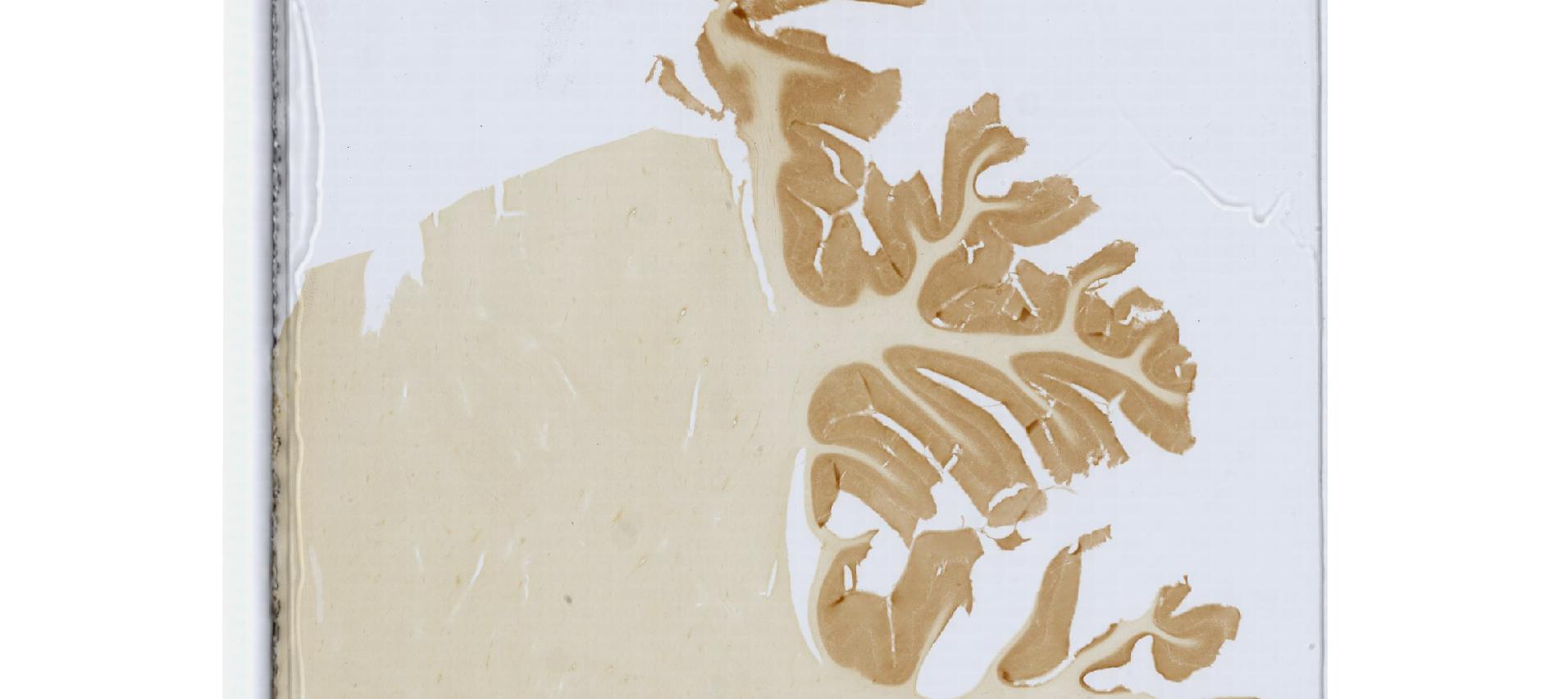

Supplement: Supplementary file 1 [file Presentation_1.ZIP › shank3-immunohistochemistry/case-3/cerebellum.jpg]

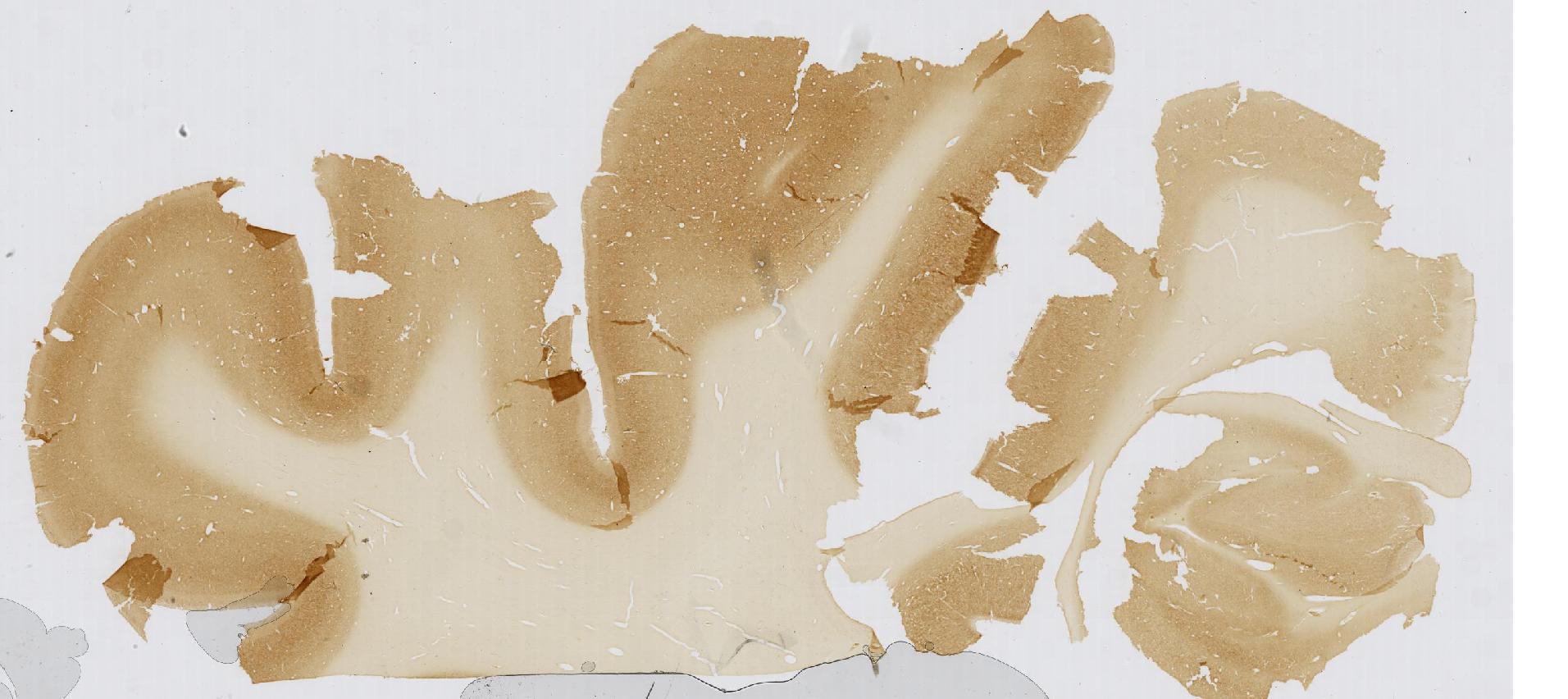

Supplement: Supplementary file 1 [file Presentation_1.ZIP › shank3-immunohistochemistry/case-3/hippocampal formation.jpg]

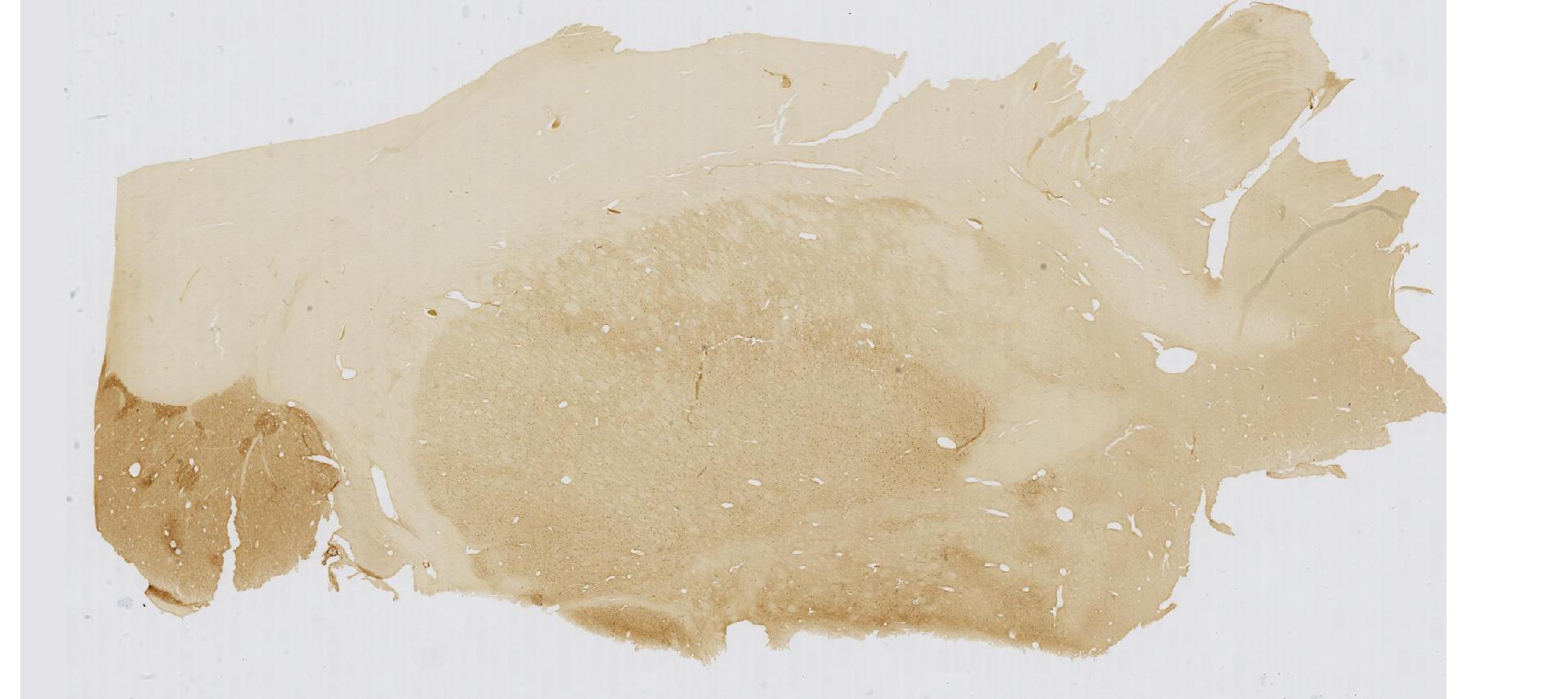

Supplement: Supplementary file 1 [file Presentation_1.ZIP › shank3-immunohistochemistry/case-3/hypothalamus.jpg]

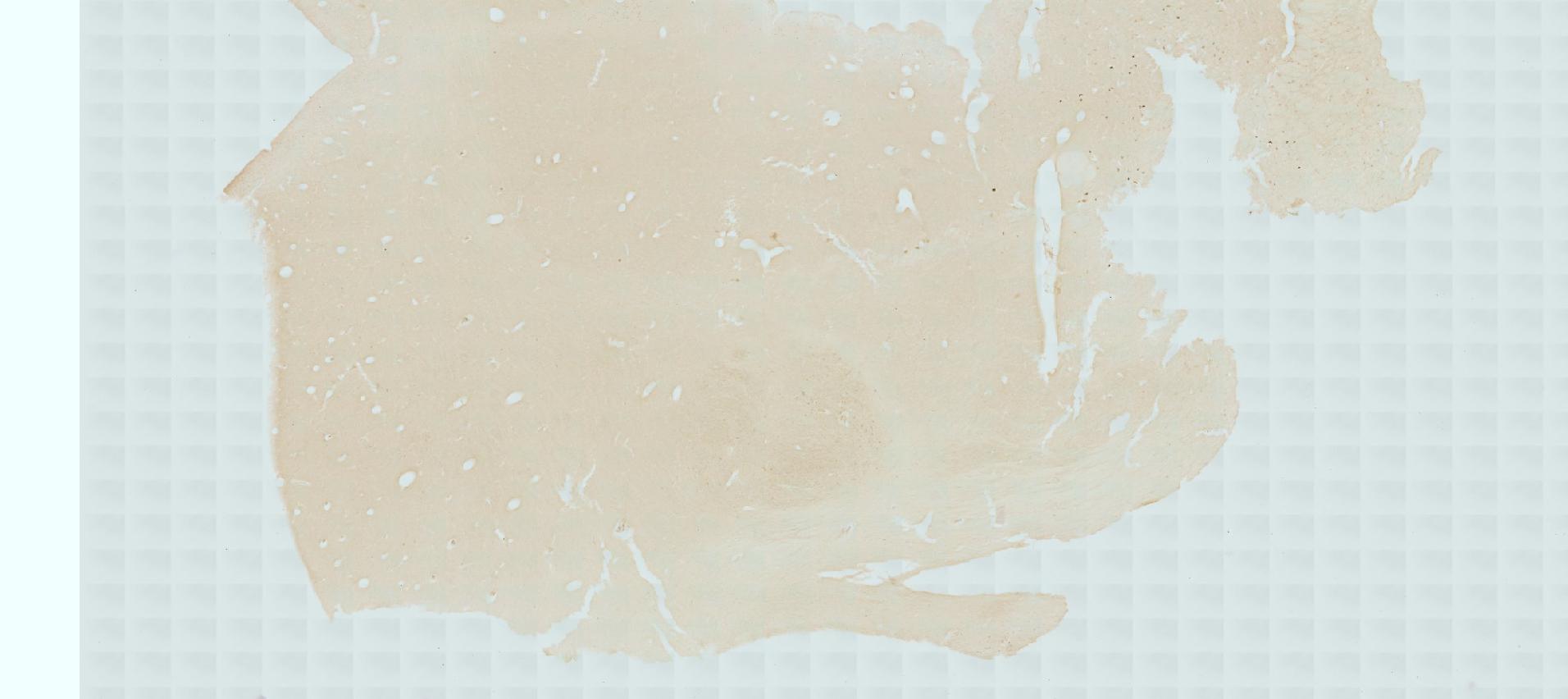

Supplement: Supplementary file 1 [file Presentation_1.ZIP › shank3-immunohistochemistry/case-3/midbrain.jpg]

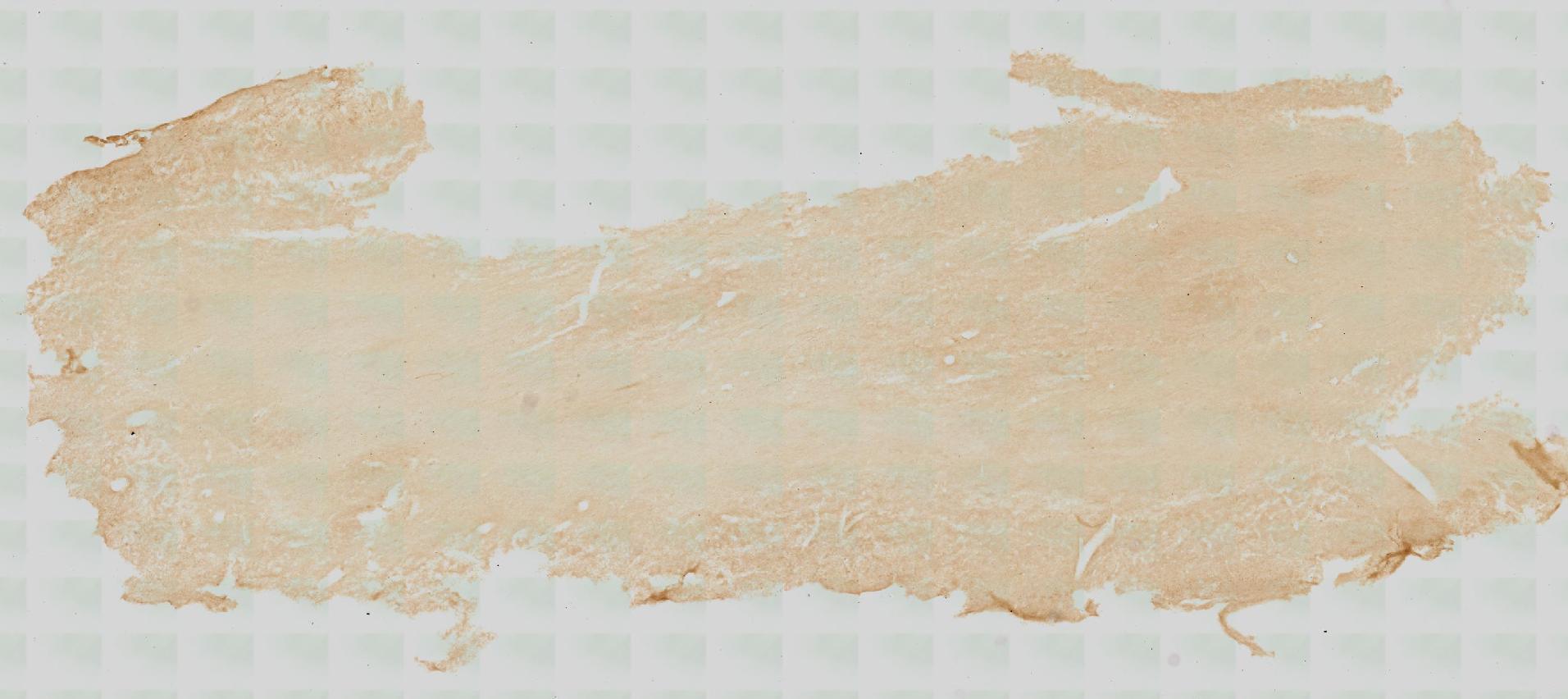

Supplement: Supplementary file 1 [file Presentation_1.ZIP › shank3-immunohistochemistry/case-3/olfactory bulb.jpg]

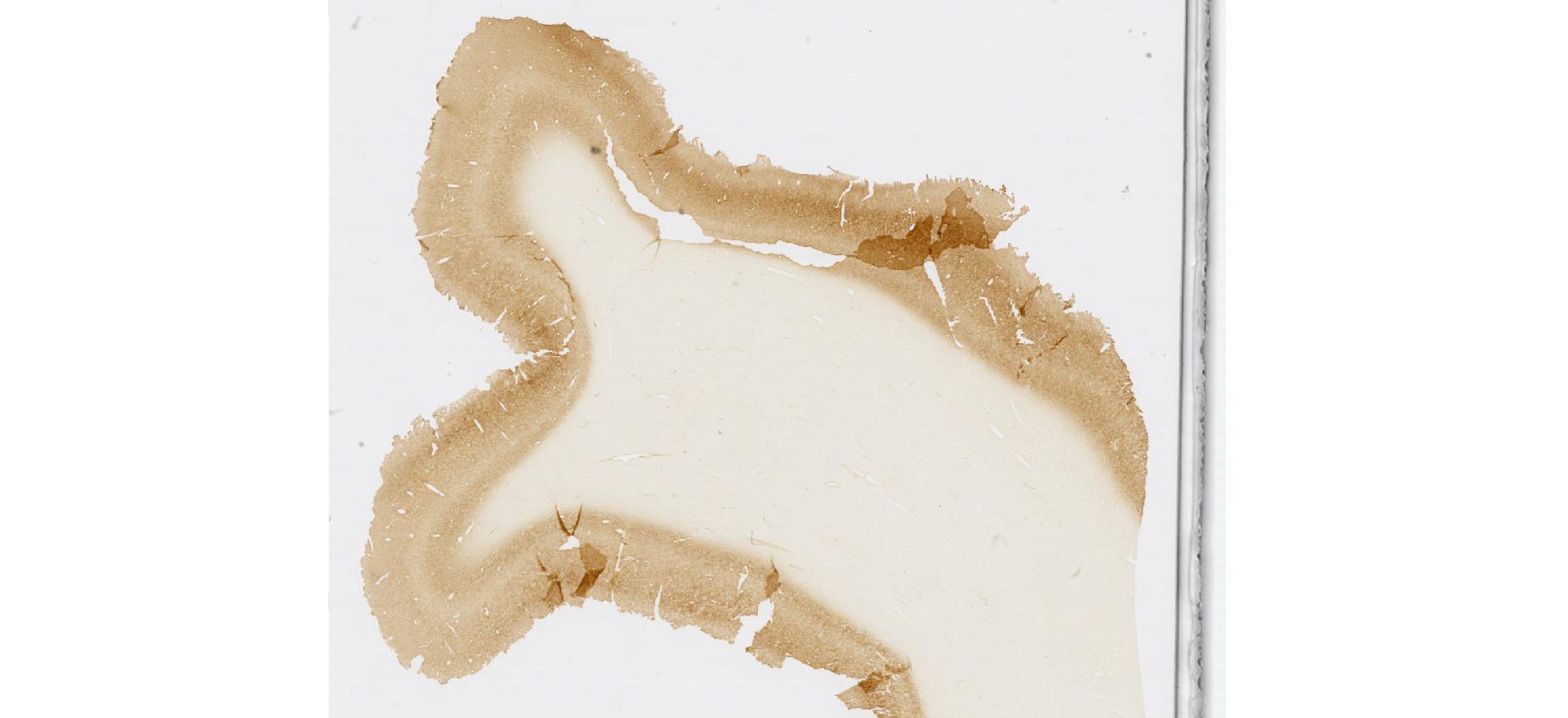

Supplement: Supplementary file 1 [file Presentation_1.ZIP › shank3-immunohistochemistry/case-3/postcentral cortex.jpg]

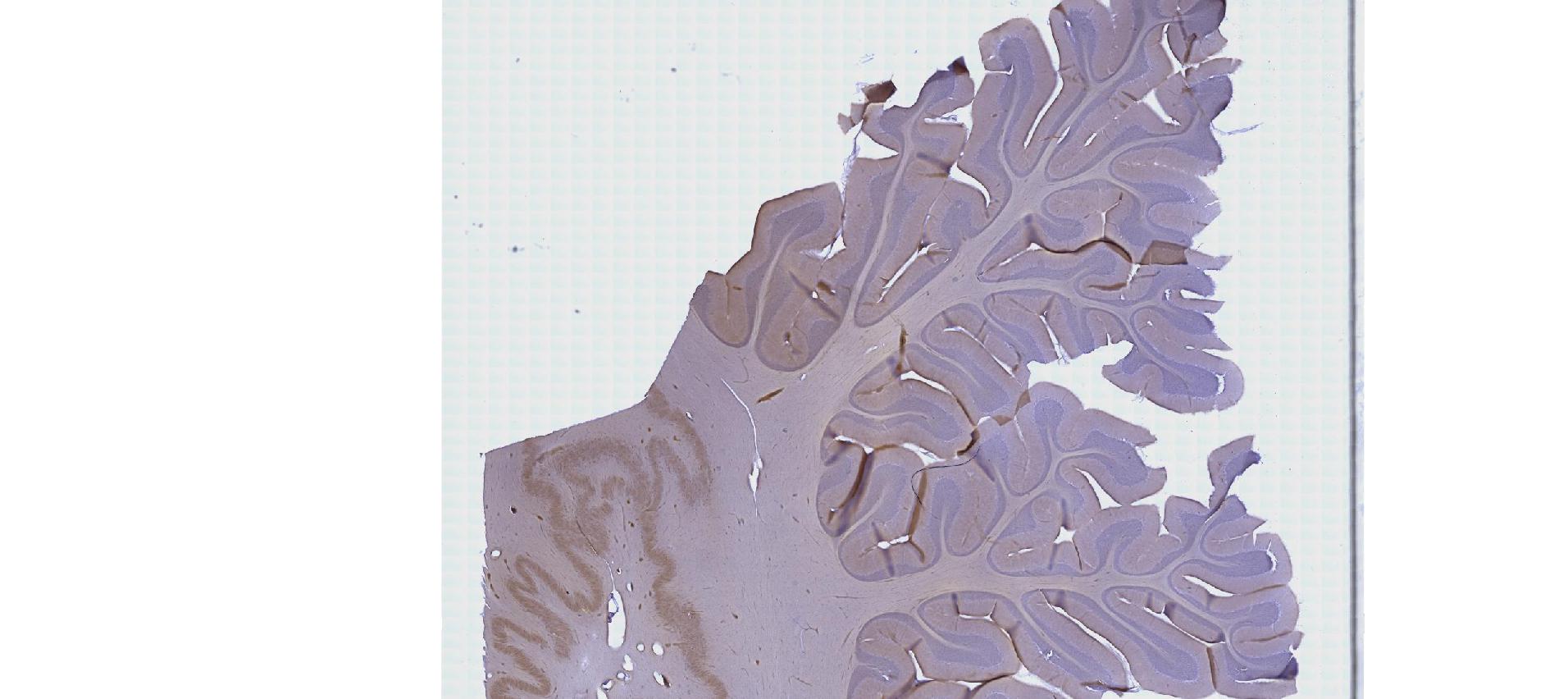

Supplement: Supplementary file 1 [file Presentation_1.ZIP › shank3-immunohistochemistry/case-4/cerebellum.jpg]

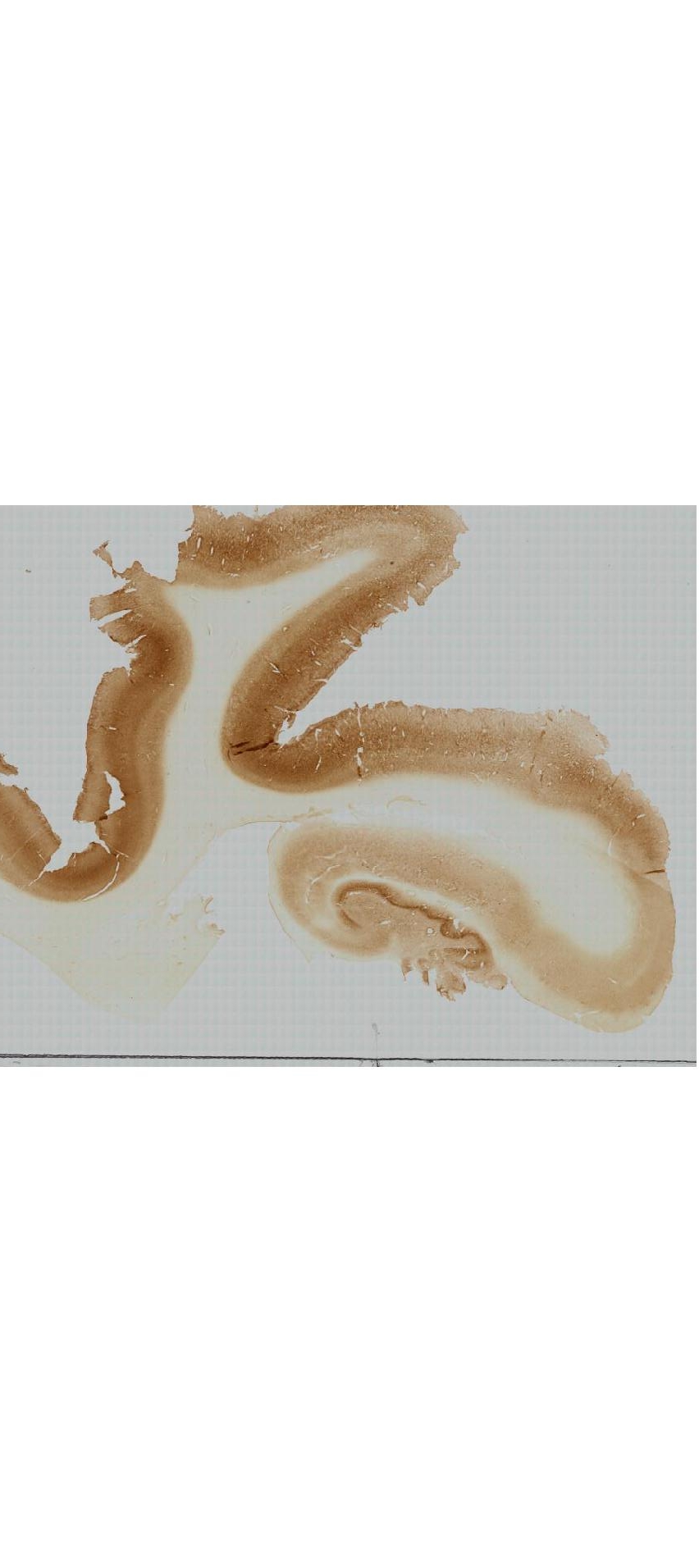

Supplement: Supplementary file 1 [file Presentation_1.ZIP › shank3-immunohistochemistry/case-4/hippocampal formation.jpg]

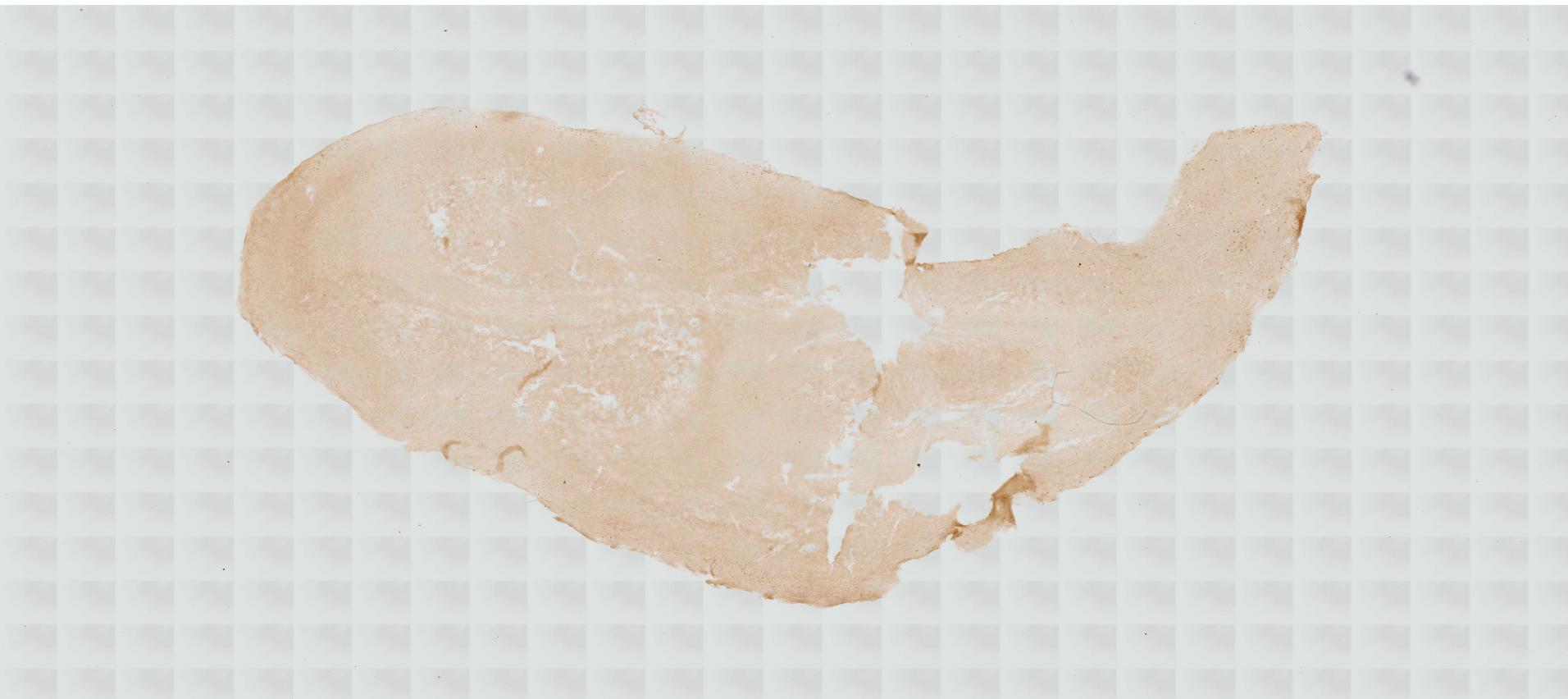

Supplement: Supplementary file 1 [file Presentation_1.ZIP › shank3-immunohistochemistry/case-4/olfactory bulb.jpg]

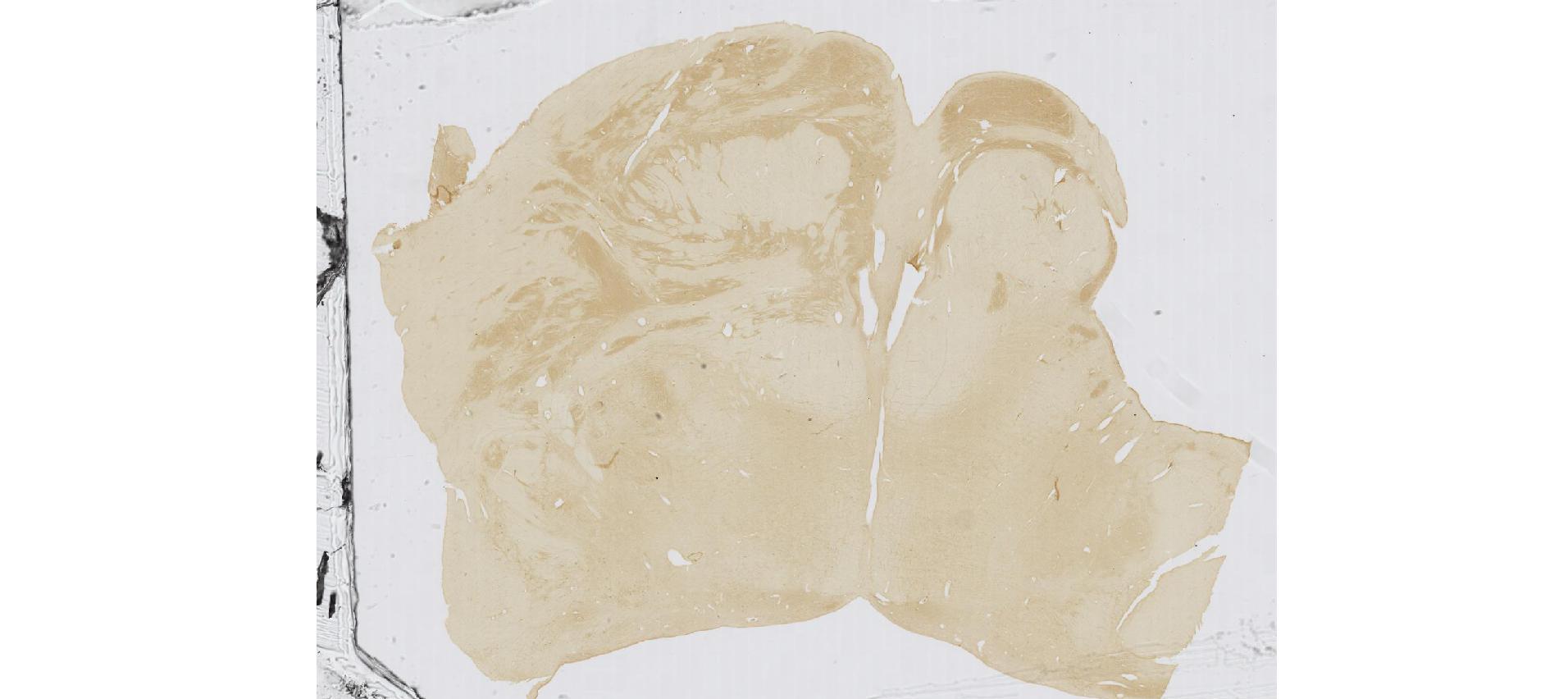

Supplement: Supplementary file 1 [file Presentation_1.ZIP › shank3-immunohistochemistry/case-4/pons.jpg]

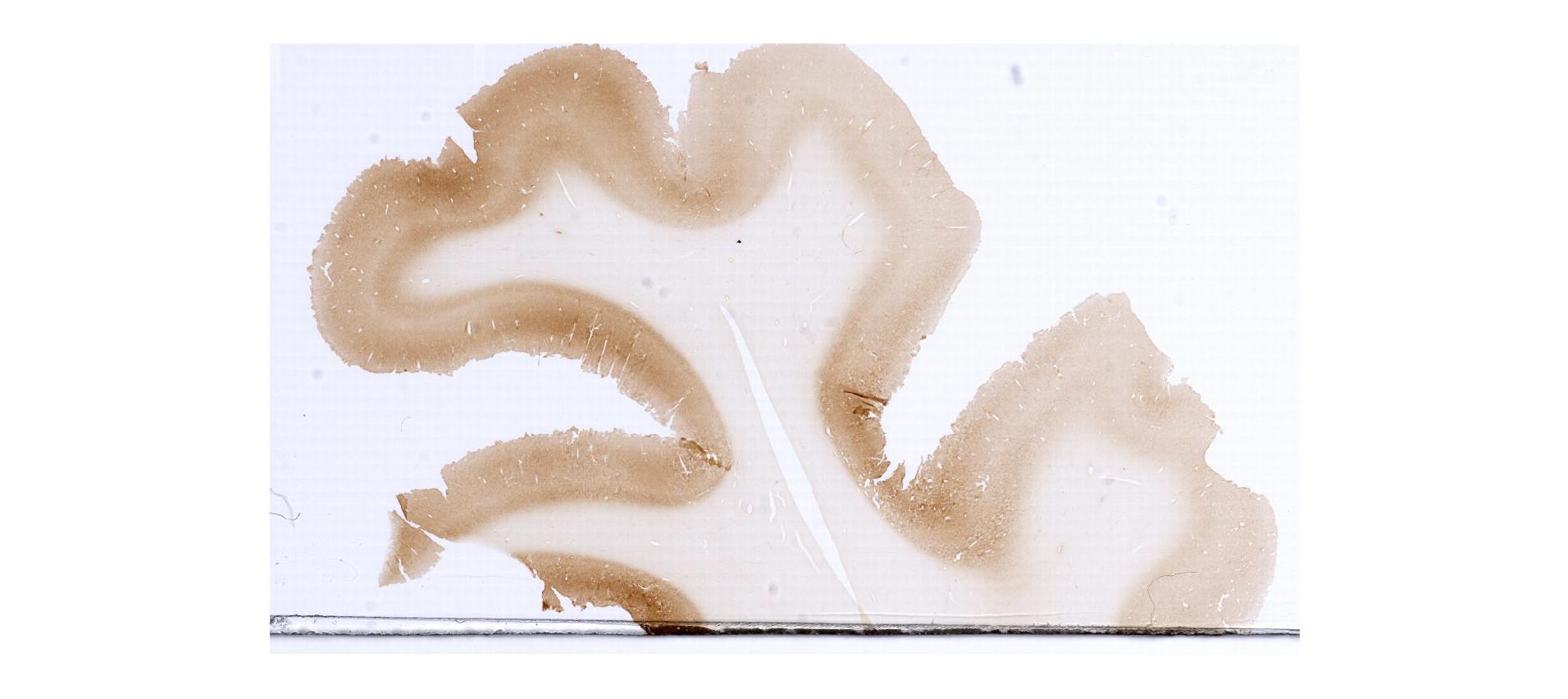

Supplement: Supplementary file 1 [file Presentation_1.ZIP › shank3-immunohistochemistry/case-4/prefrontal cortex.jpg]

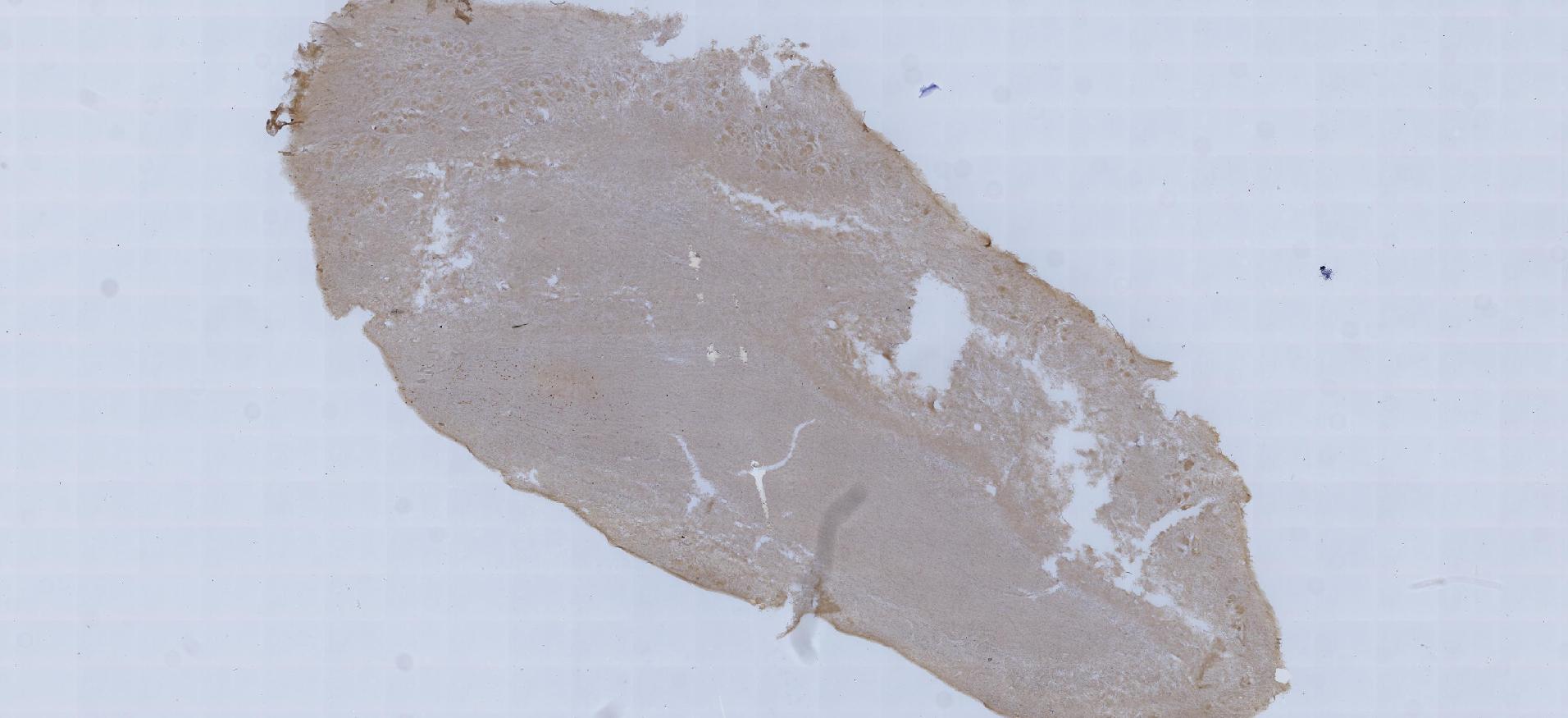

Supplement: Supplementary file 1 [file Presentation_1.ZIP › shank3-immunohistochemistry/case-5-counterstain/1-olfactory bulb-counterstain.jpg]

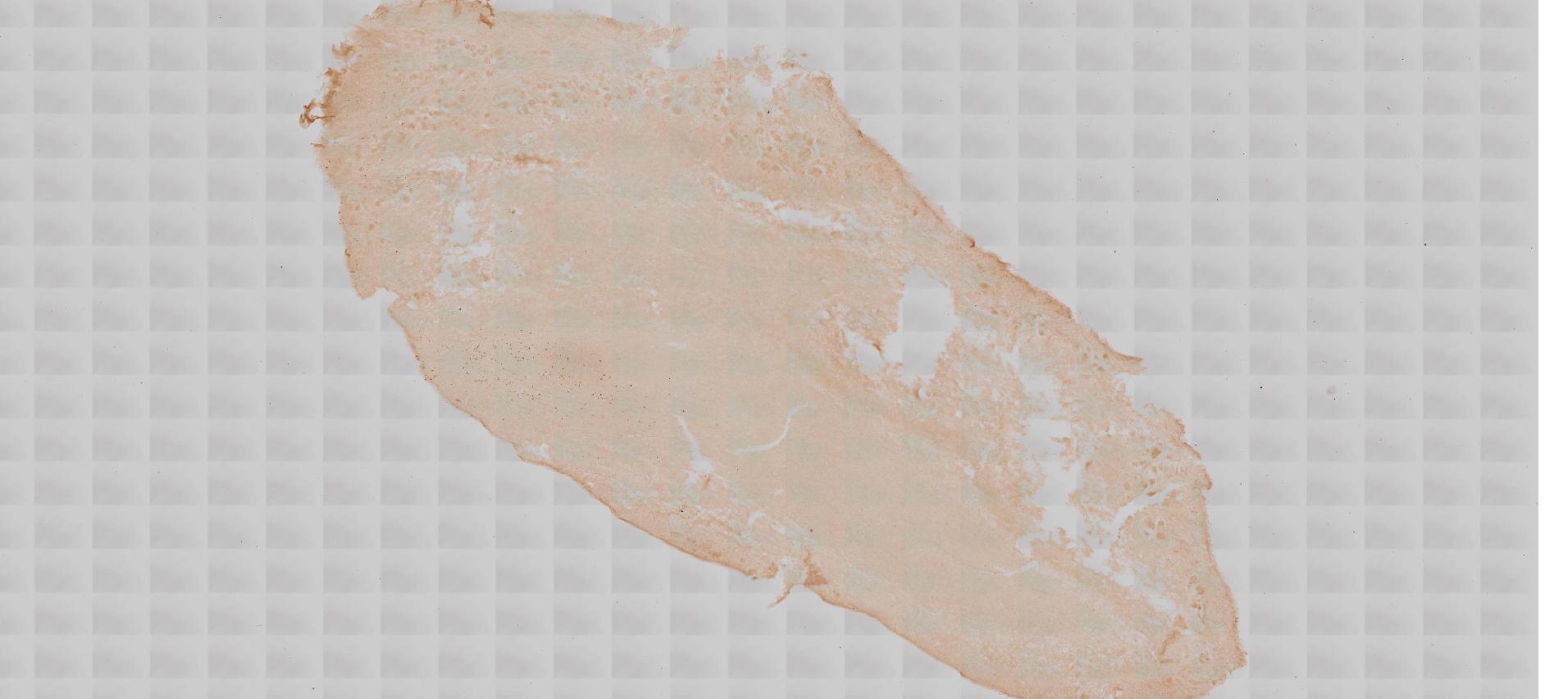

Supplement: Supplementary file 1 [file Presentation_1.ZIP › shank3-immunohistochemistry/case-5-counterstain/1-olfactory bulb.jpg]

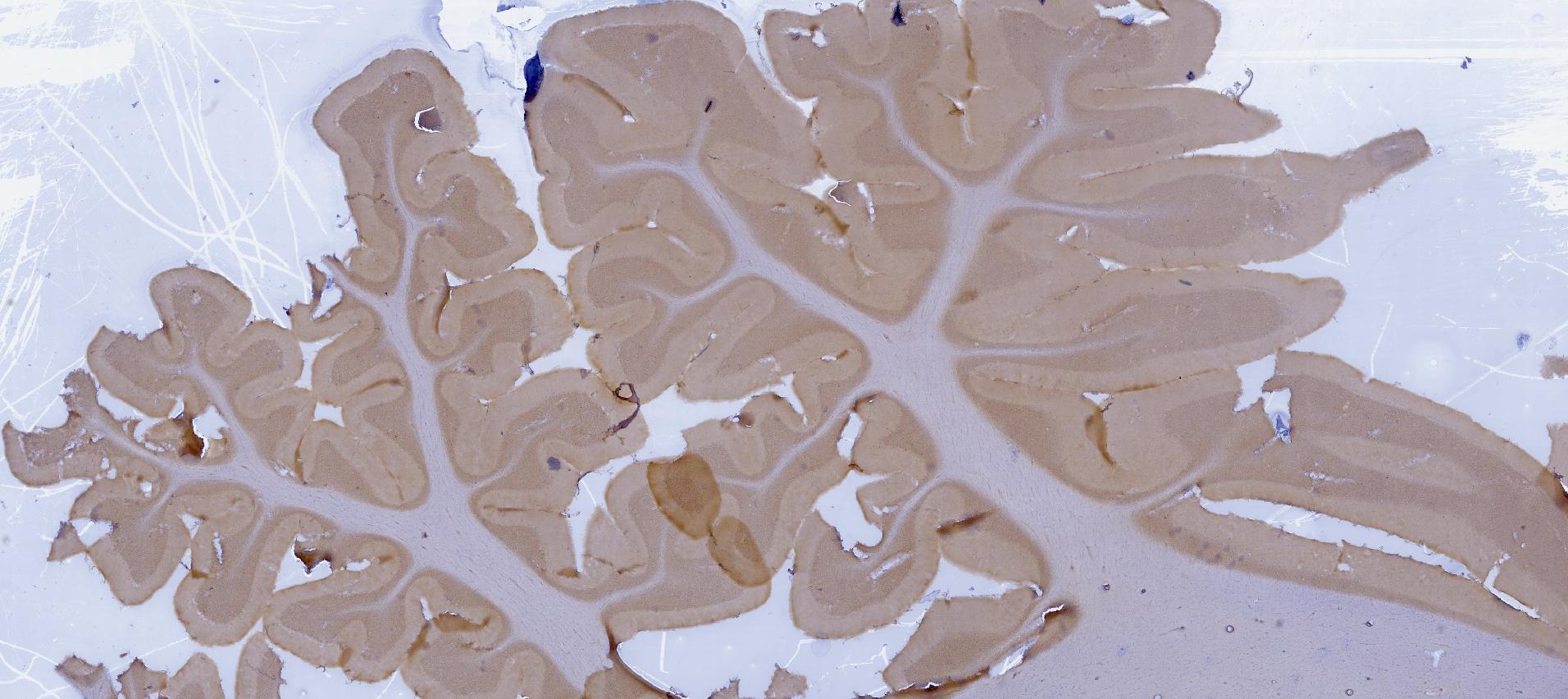

Supplement: Supplementary file 1 [file Presentation_1.ZIP › shank3-immunohistochemistry/case-5-counterstain/10-cerebellum-Counterstain.jpg]

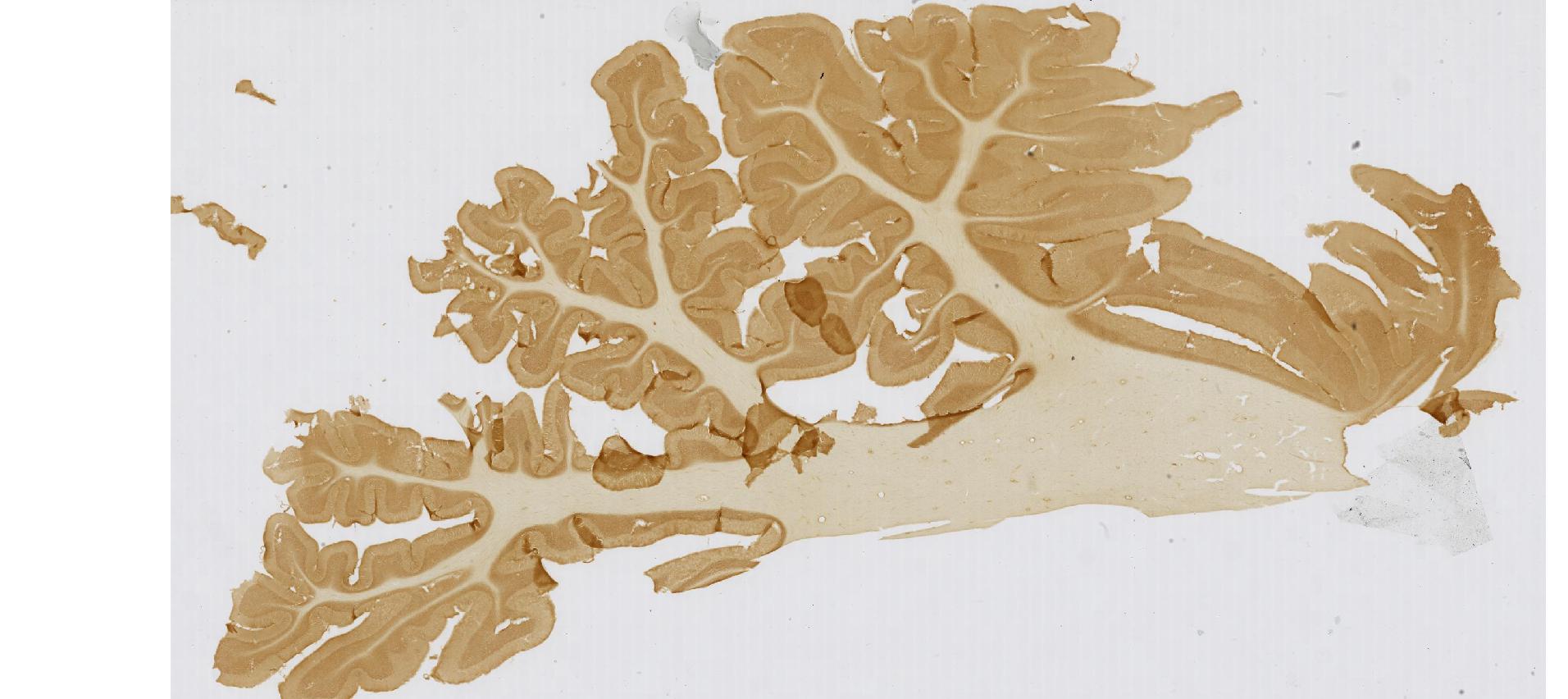

Supplement: Supplementary file 1 [file Presentation_1.ZIP › shank3-immunohistochemistry/case-5-counterstain/10-cerebellum.jpg]

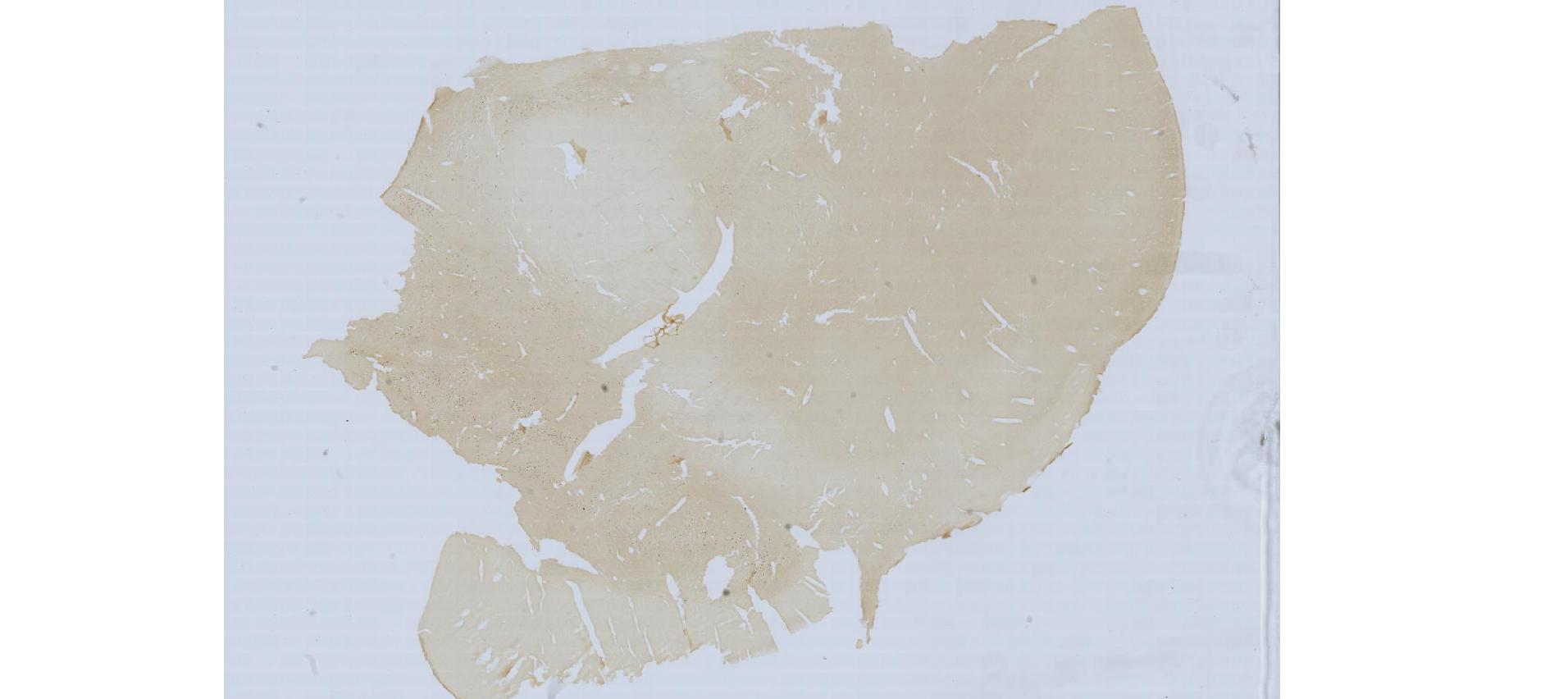

Supplement: Supplementary file 1 [file Presentation_1.ZIP › shank3-immunohistochemistry/case-5-counterstain/11-midbrain.jpg]

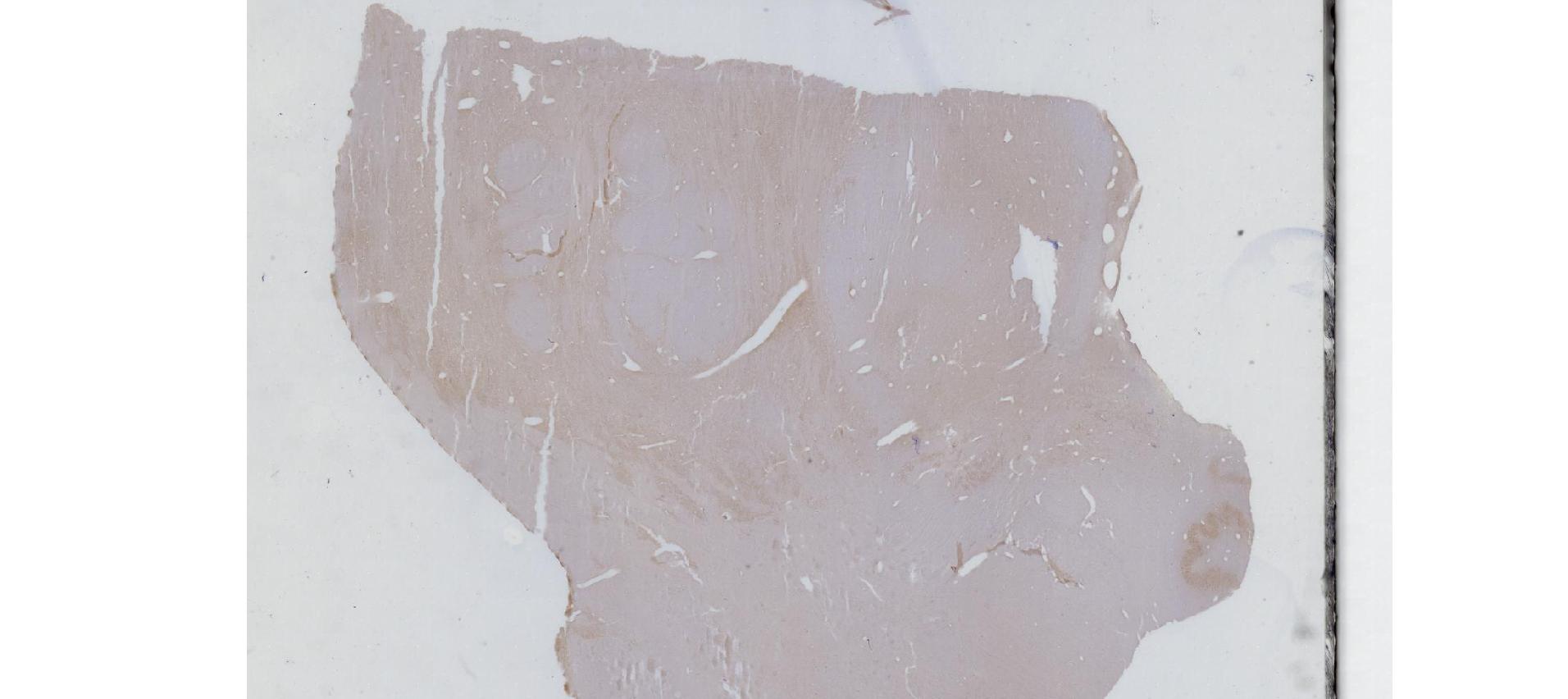

Supplement: Supplementary file 1 [file Presentation_1.ZIP › shank3-immunohistochemistry/case-5-counterstain/12-pons-counterstain.jpg]

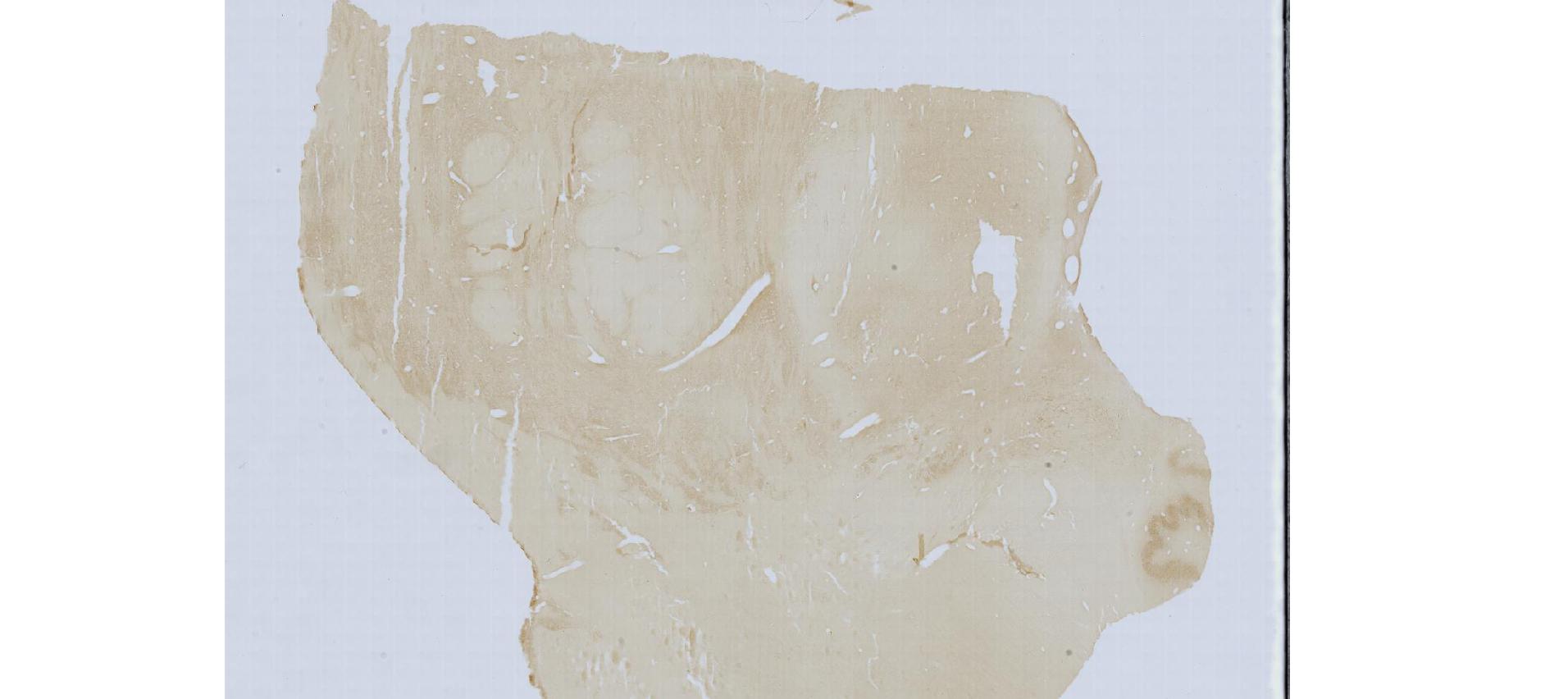

Supplement: Supplementary file 1 [file Presentation_1.ZIP › shank3-immunohistochemistry/case-5-counterstain/12-pons.jpg]

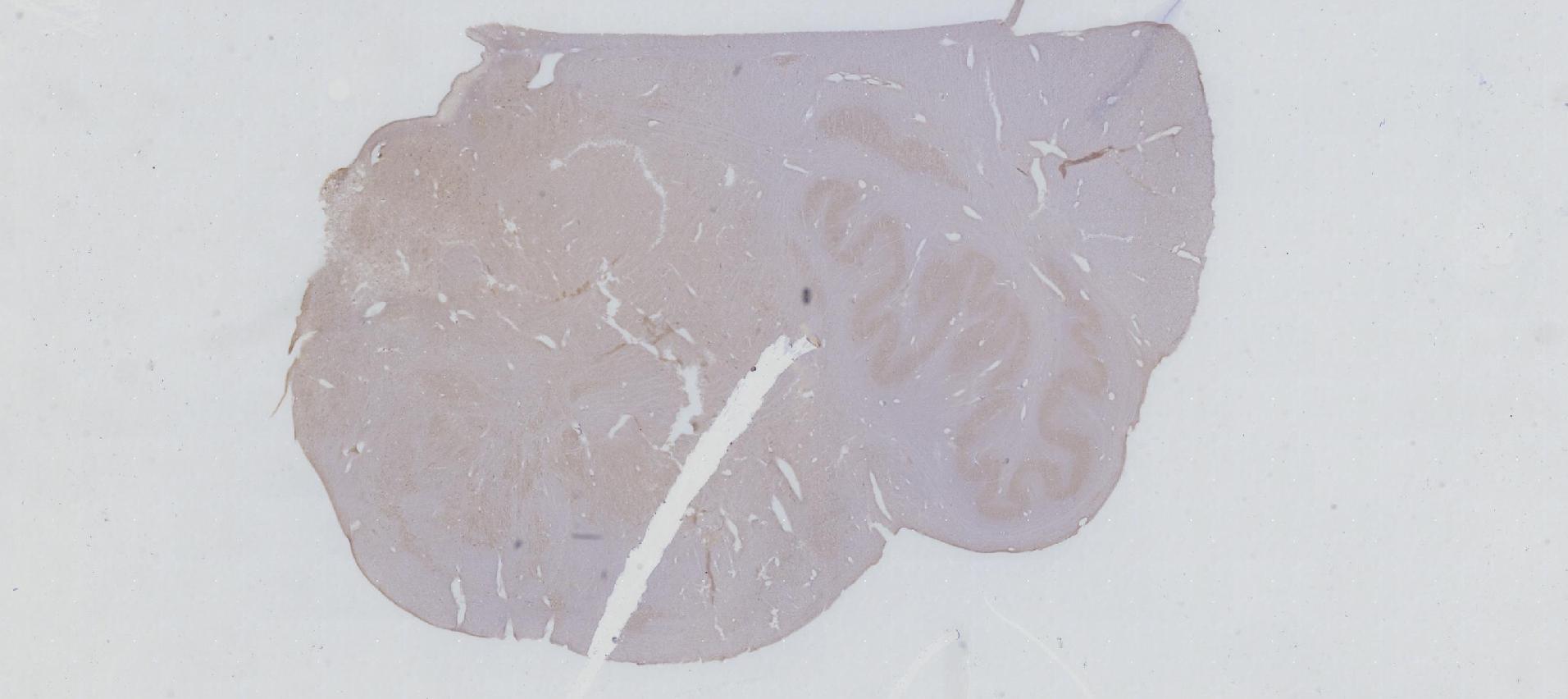

Supplement: Supplementary file 1 [file Presentation_1.ZIP › shank3-immunohistochemistry/case-5-counterstain/13-medulla oblongata-counterstain.jpg]

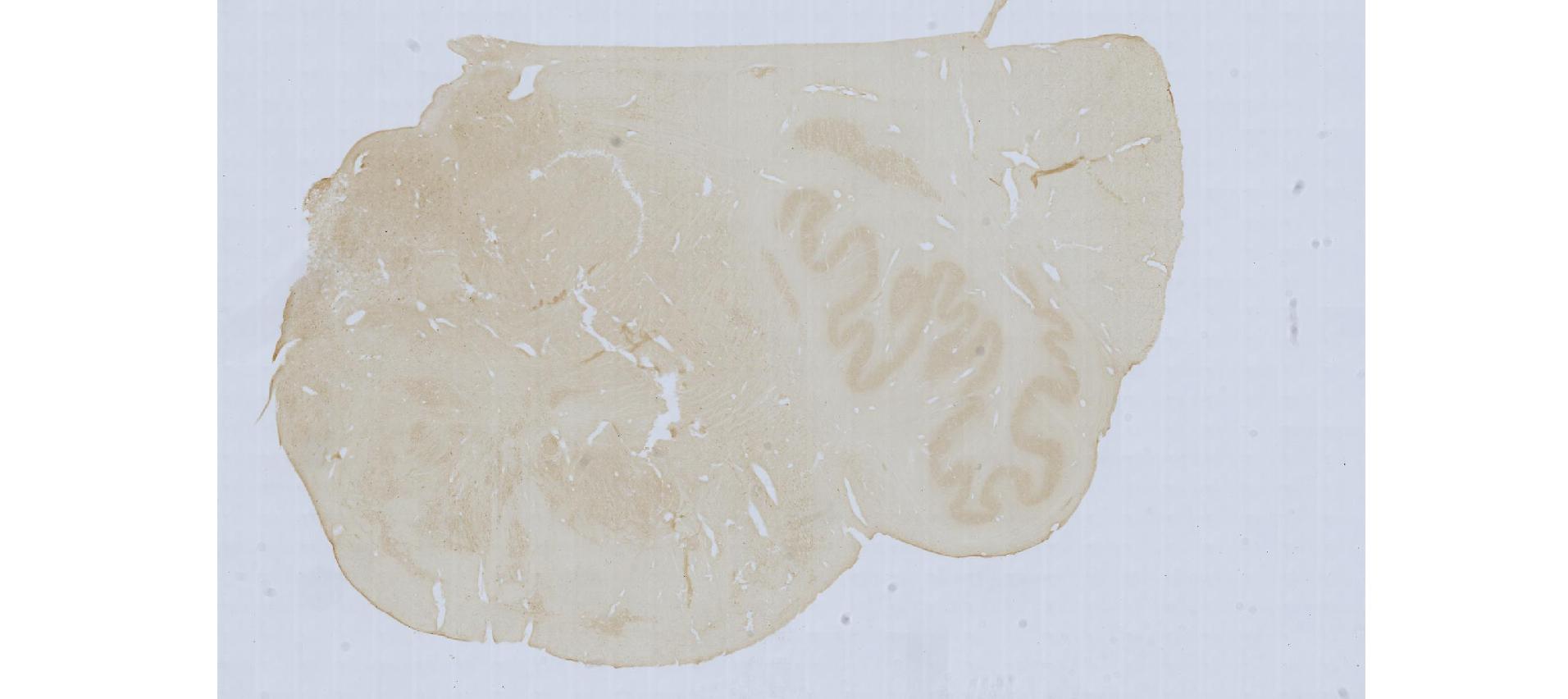

Supplement: Supplementary file 1 [file Presentation_1.ZIP › shank3-immunohistochemistry/case-5-counterstain/13-medulla oblongata.jpg]

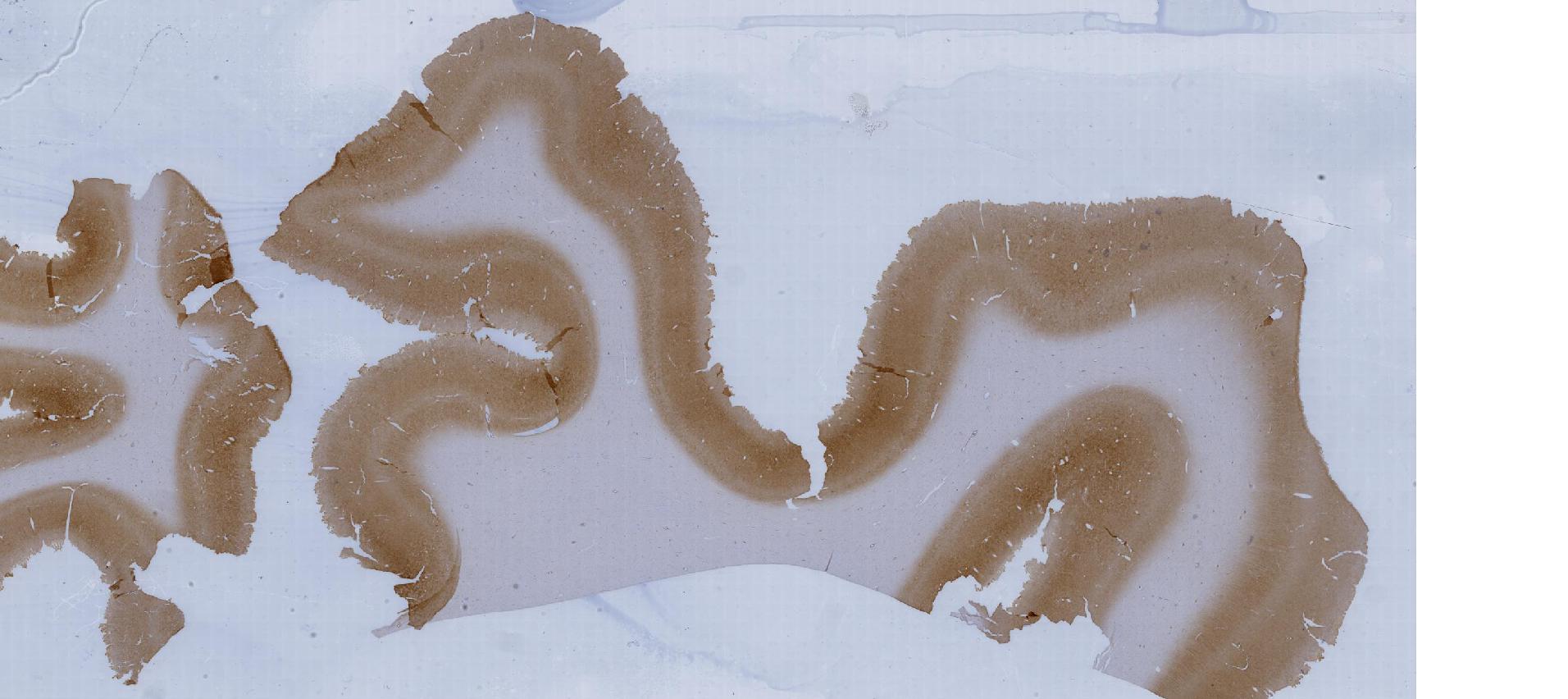

Supplement: Supplementary file 1 [file Presentation_1.ZIP › shank3-immunohistochemistry/case-5-counterstain/2-frontal cortex-counterstain.jpg]

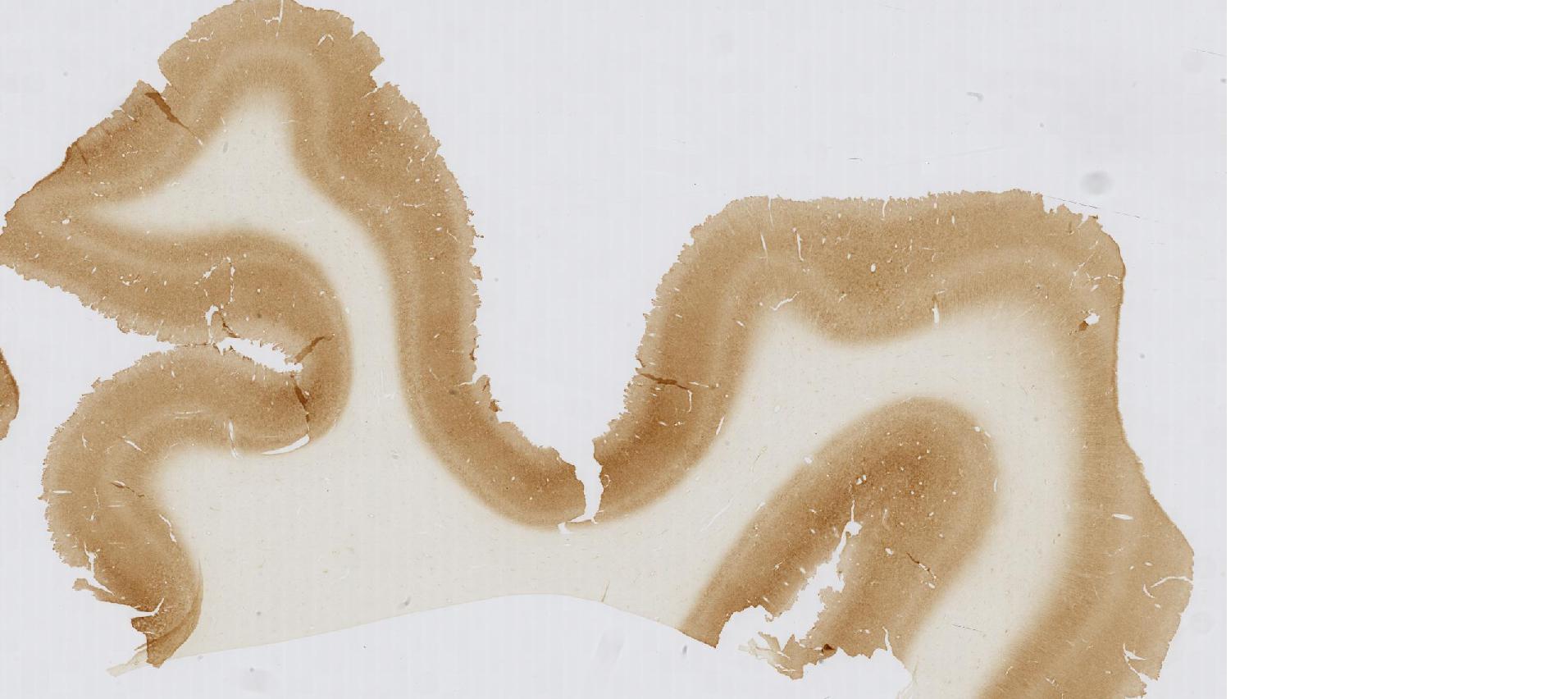

Supplement: Supplementary file 1 [file Presentation_1.ZIP › shank3-immunohistochemistry/case-5-counterstain/2-prefrontal cortex.jpg]

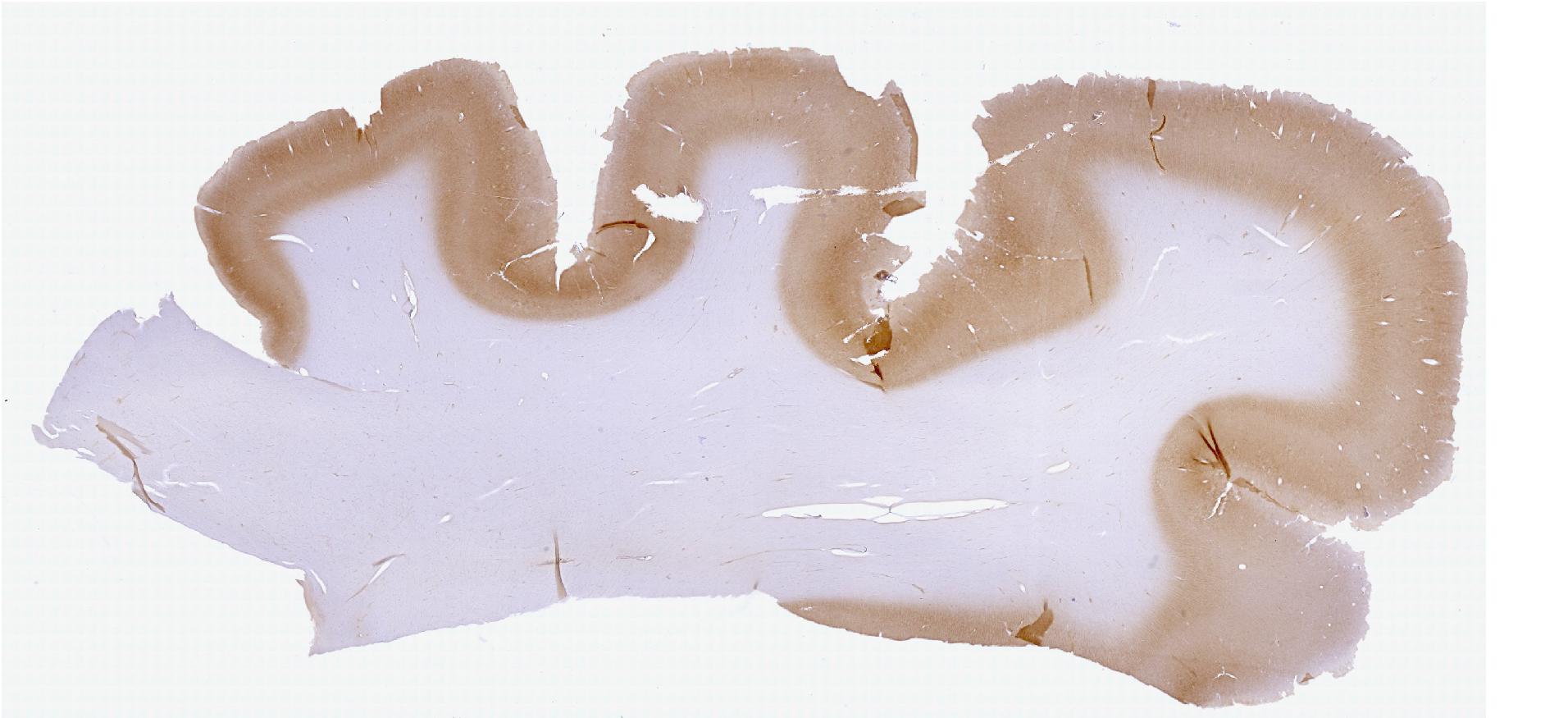

Supplement: Supplementary file 1 [file Presentation_1.ZIP › shank3-immunohistochemistry/case-5-counterstain/3-anterior cingulate neocortex-counterstain.jpg]

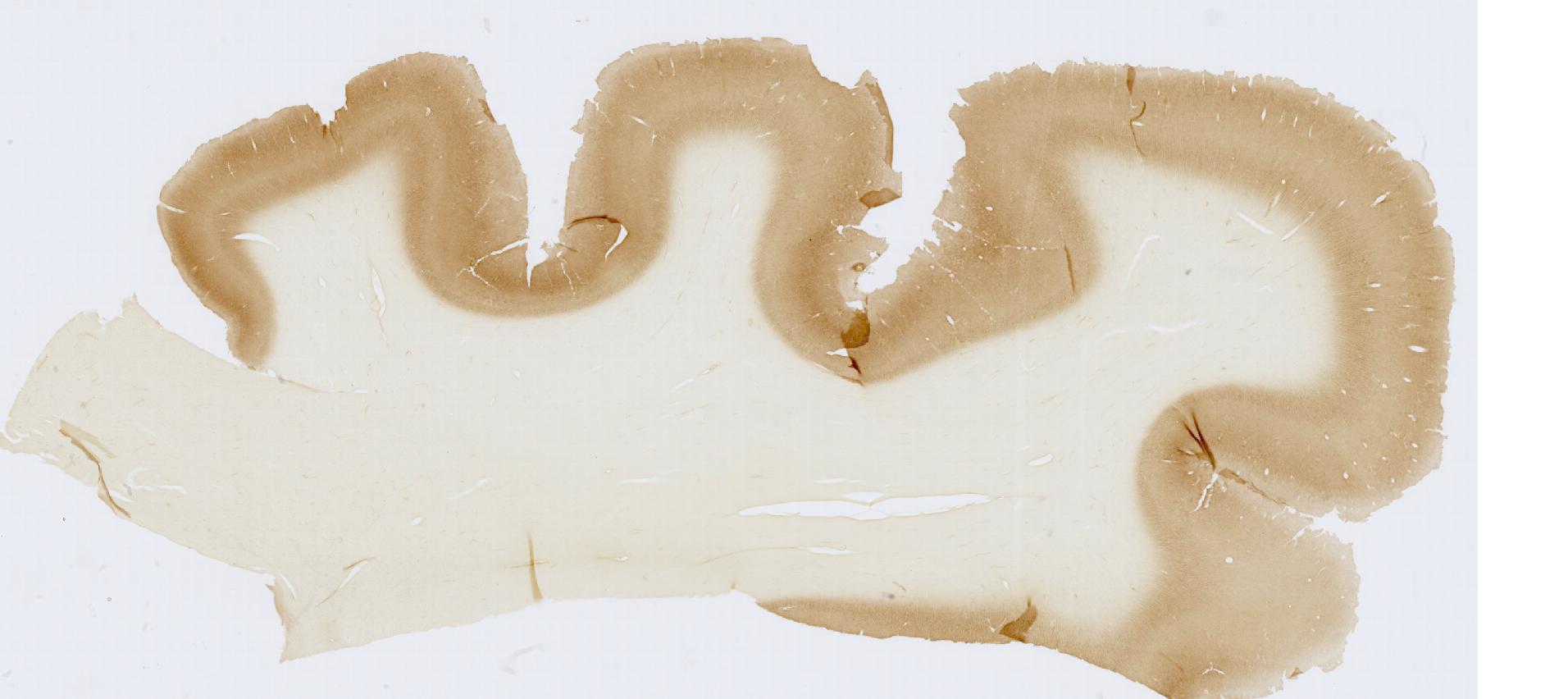

Supplement: Supplementary file 1 [file Presentation_1.ZIP › shank3-immunohistochemistry/case-5-counterstain/3-anterior cingulate neocortex.jpg]

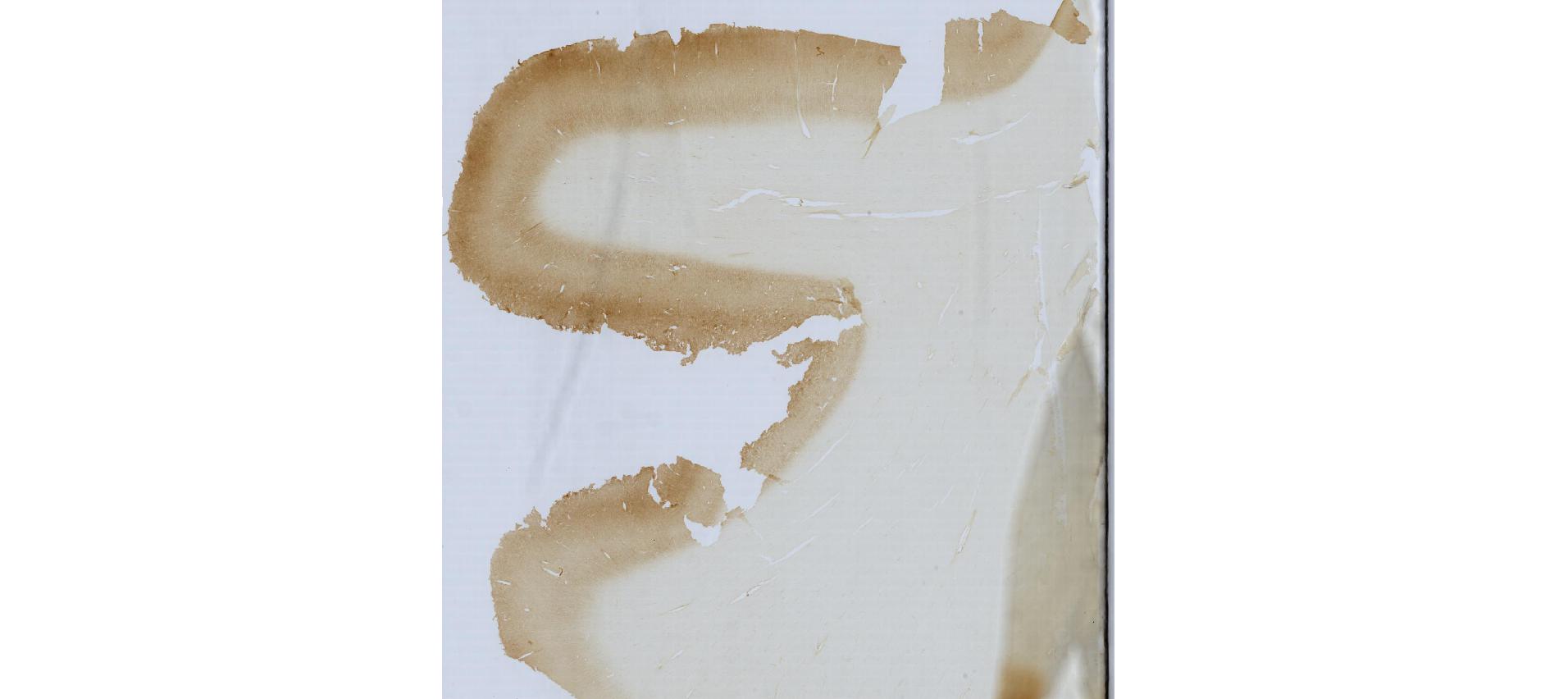

Supplement: Supplementary file 1 [file Presentation_1.ZIP › shank3-immunohistochemistry/case-5-counterstain/4- precentral cortex.jpg]

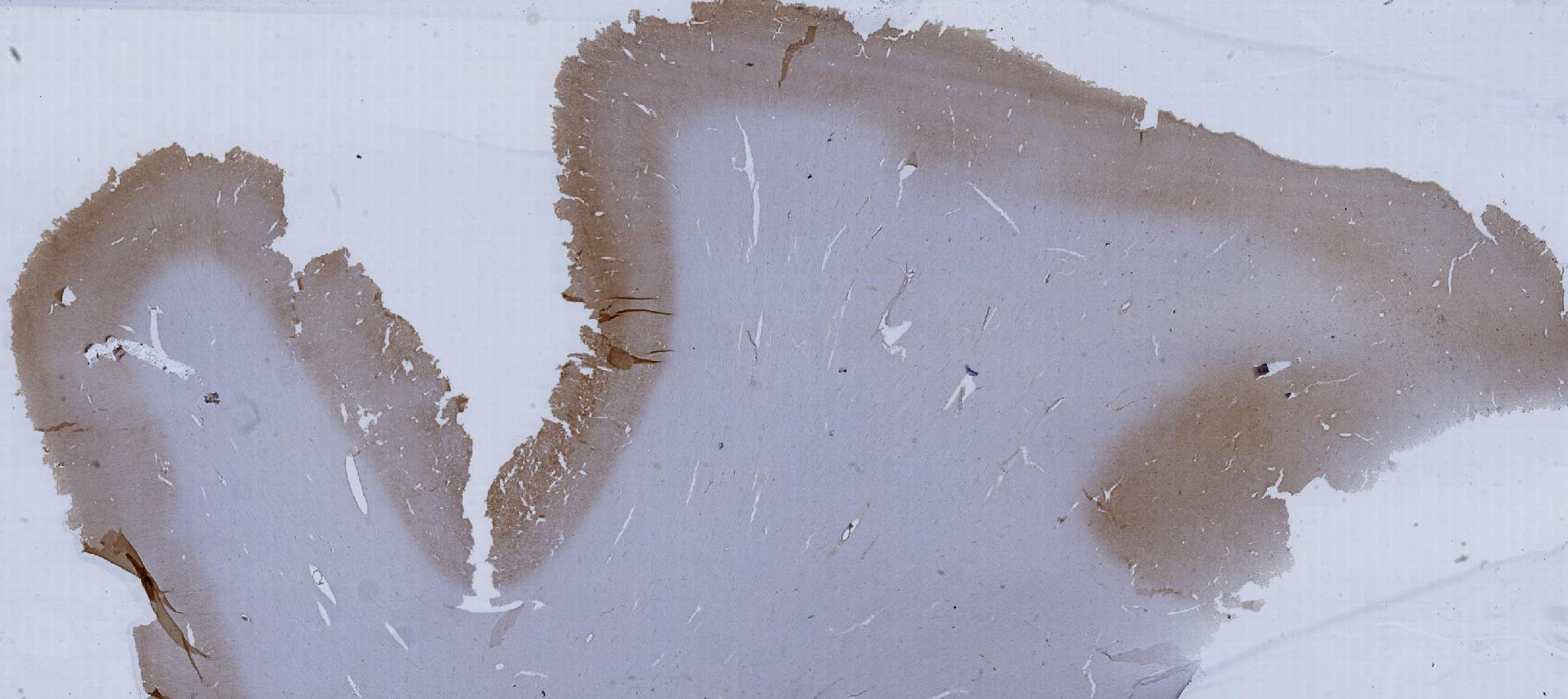

Supplement: Supplementary file 1 [file Presentation_1.ZIP › shank3-immunohistochemistry/case-5-counterstain/4-precentral cortex-counterstain.jpg]

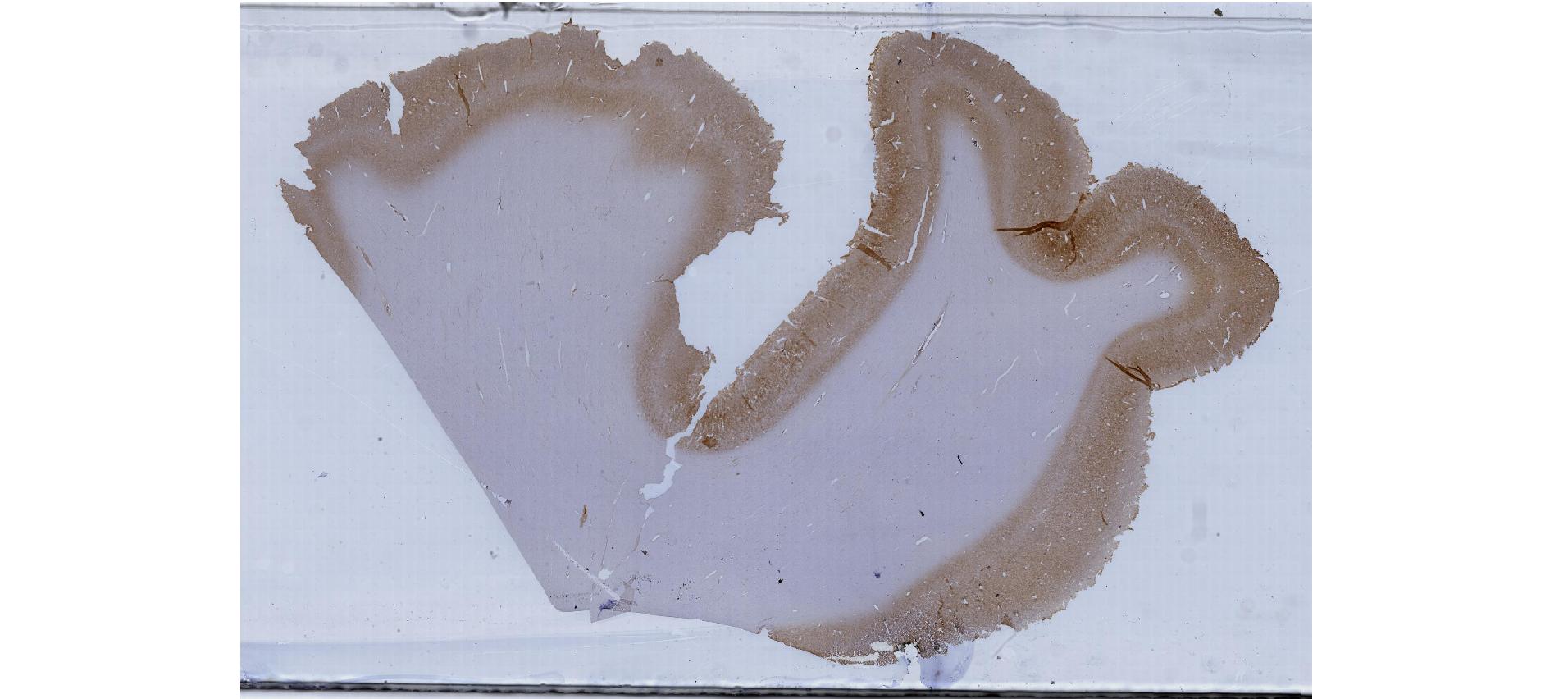

Supplement: Supplementary file 1 [file Presentation_1.ZIP › shank3-immunohistochemistry/case-5-counterstain/5-postcentral cortex-counterstain.jpg]

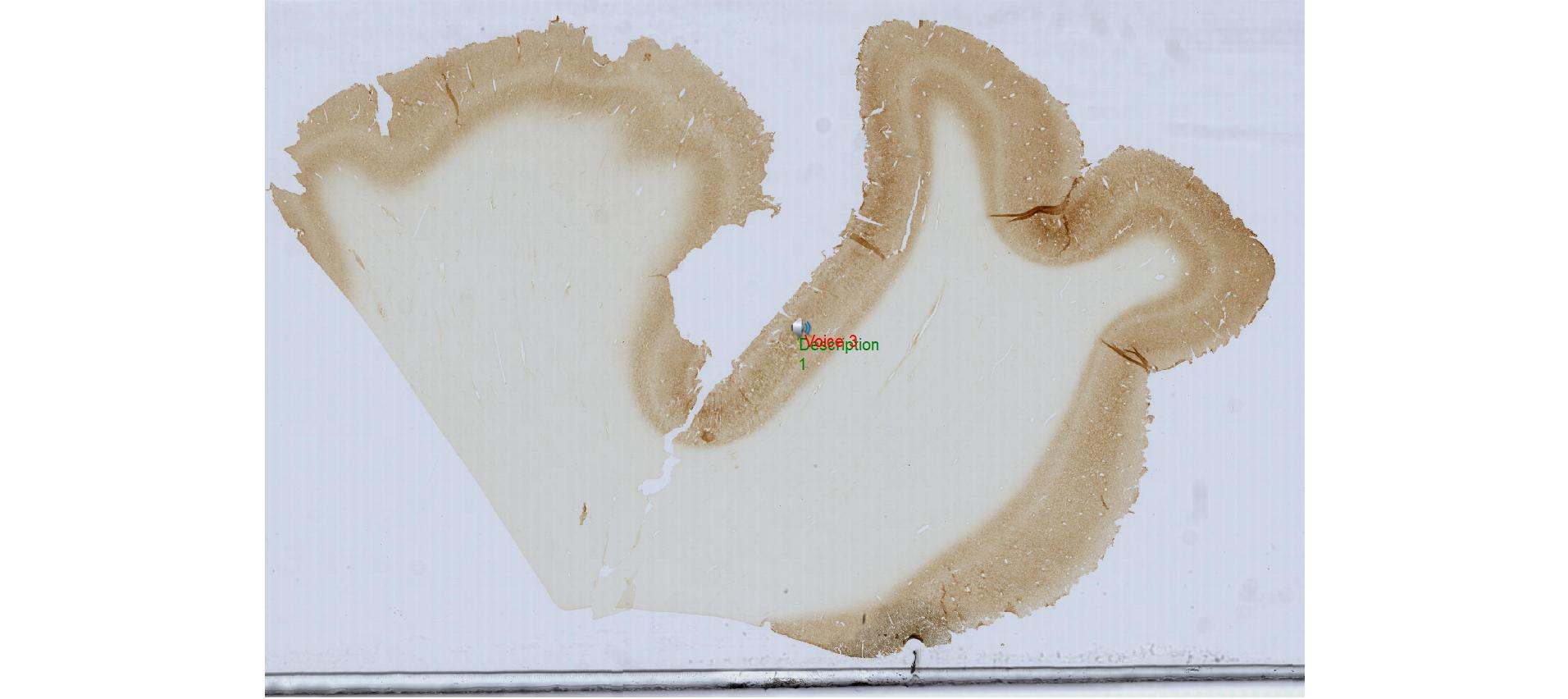

Supplement: Supplementary file 1 [file Presentation_1.ZIP › shank3-immunohistochemistry/case-5-counterstain/5-postcentral cortex.jpg]

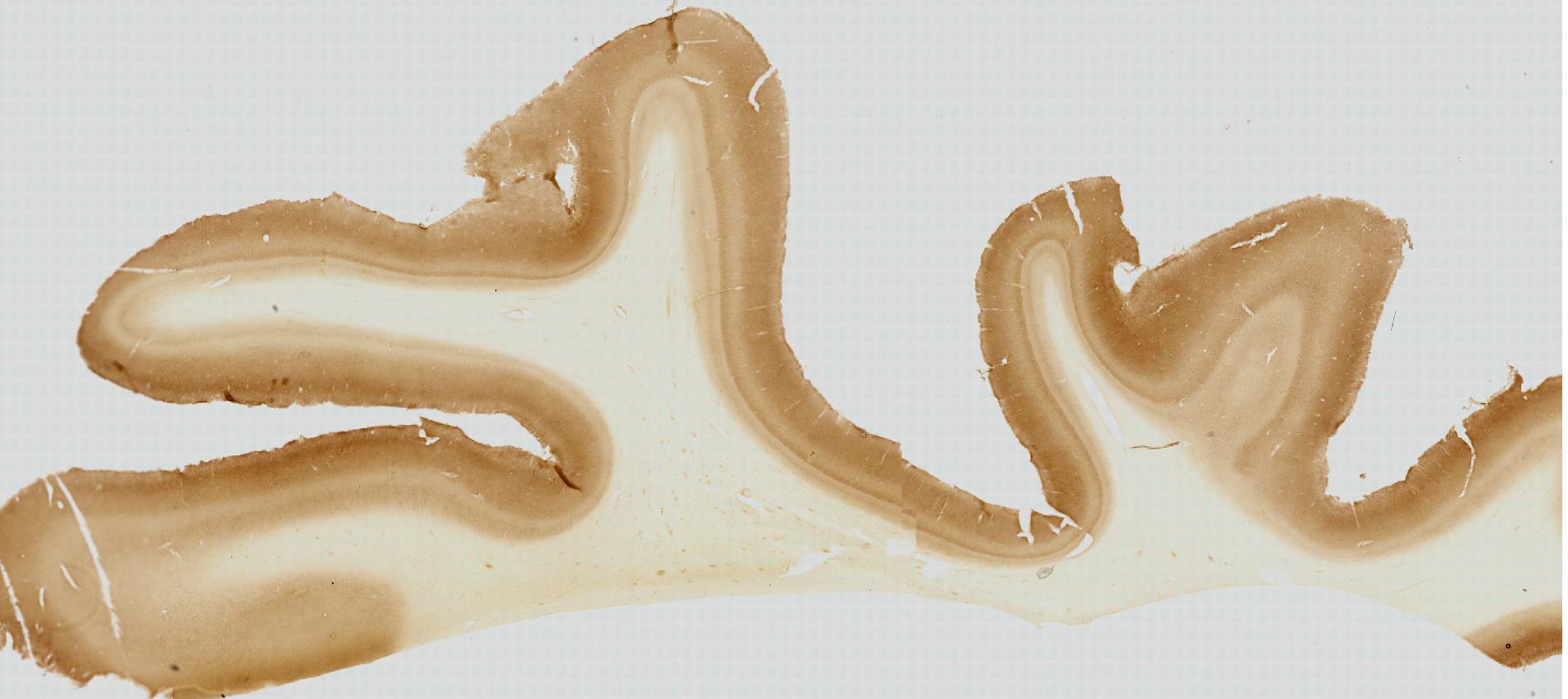

Supplement: Supplementary file 1 [file Presentation_1.ZIP › shank3-immunohistochemistry/case-5-counterstain/6- visual cortex.jpg]

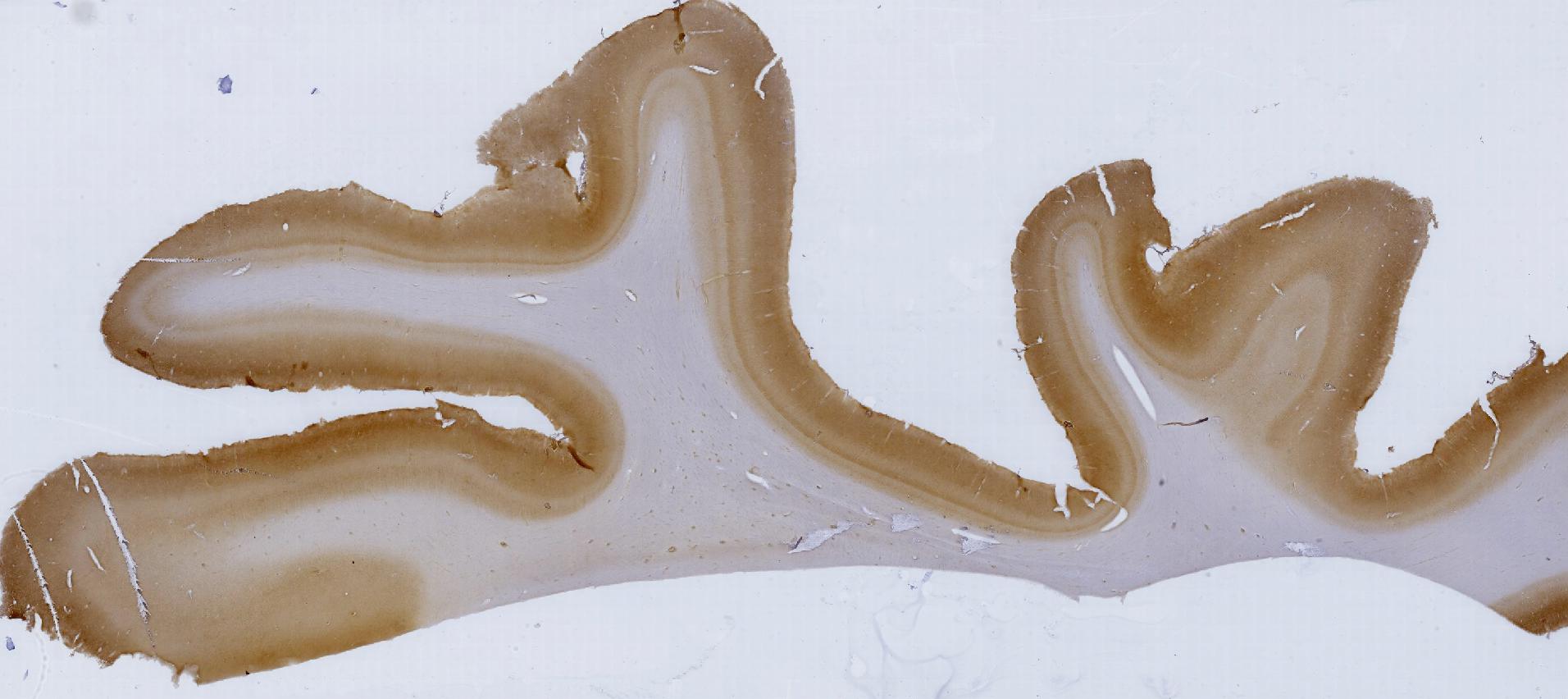

Supplement: Supplementary file 1 [file Presentation_1.ZIP › shank3-immunohistochemistry/case-5-counterstain/6-visual cortex-counterstain.jpg]

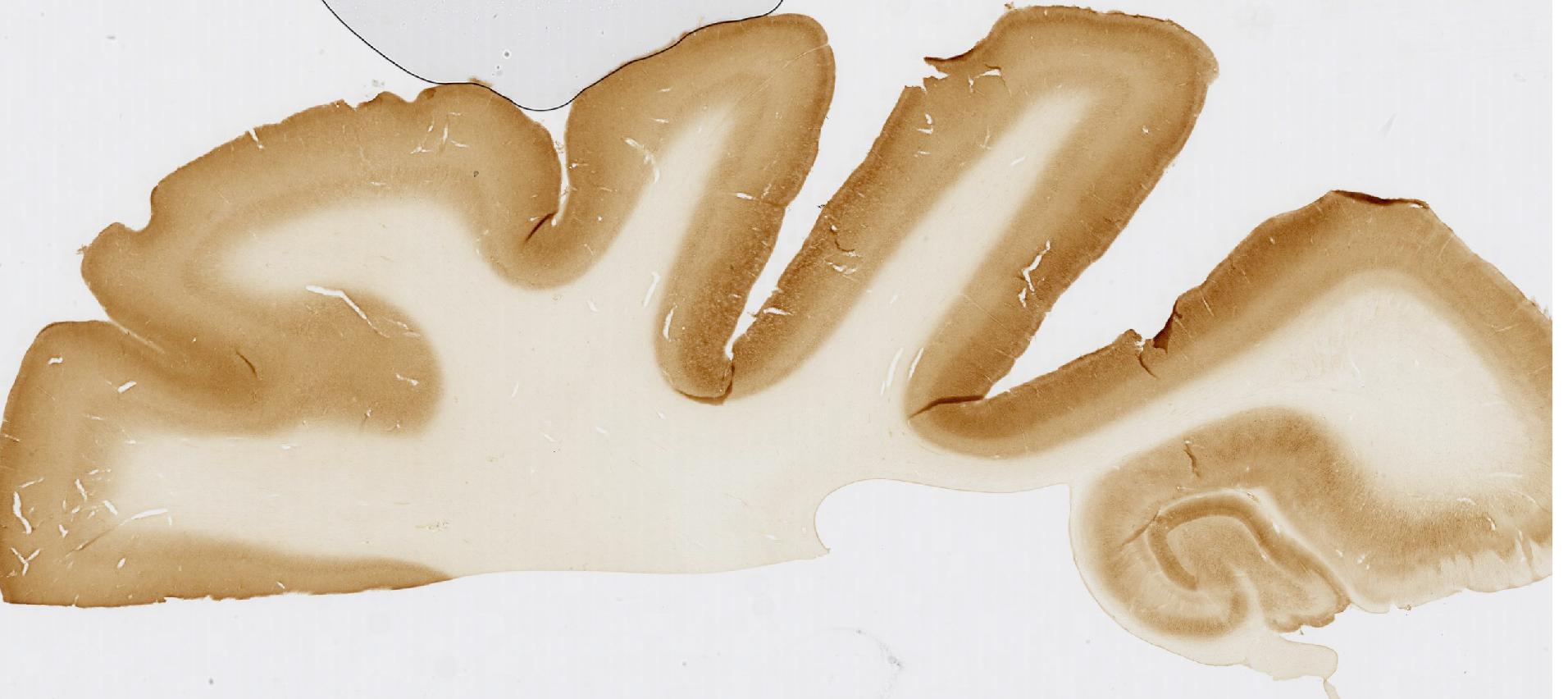

Supplement: Supplementary file 1 [file Presentation_1.ZIP › shank3-immunohistochemistry/case-5-counterstain/7- hippocampal formation.jpg]

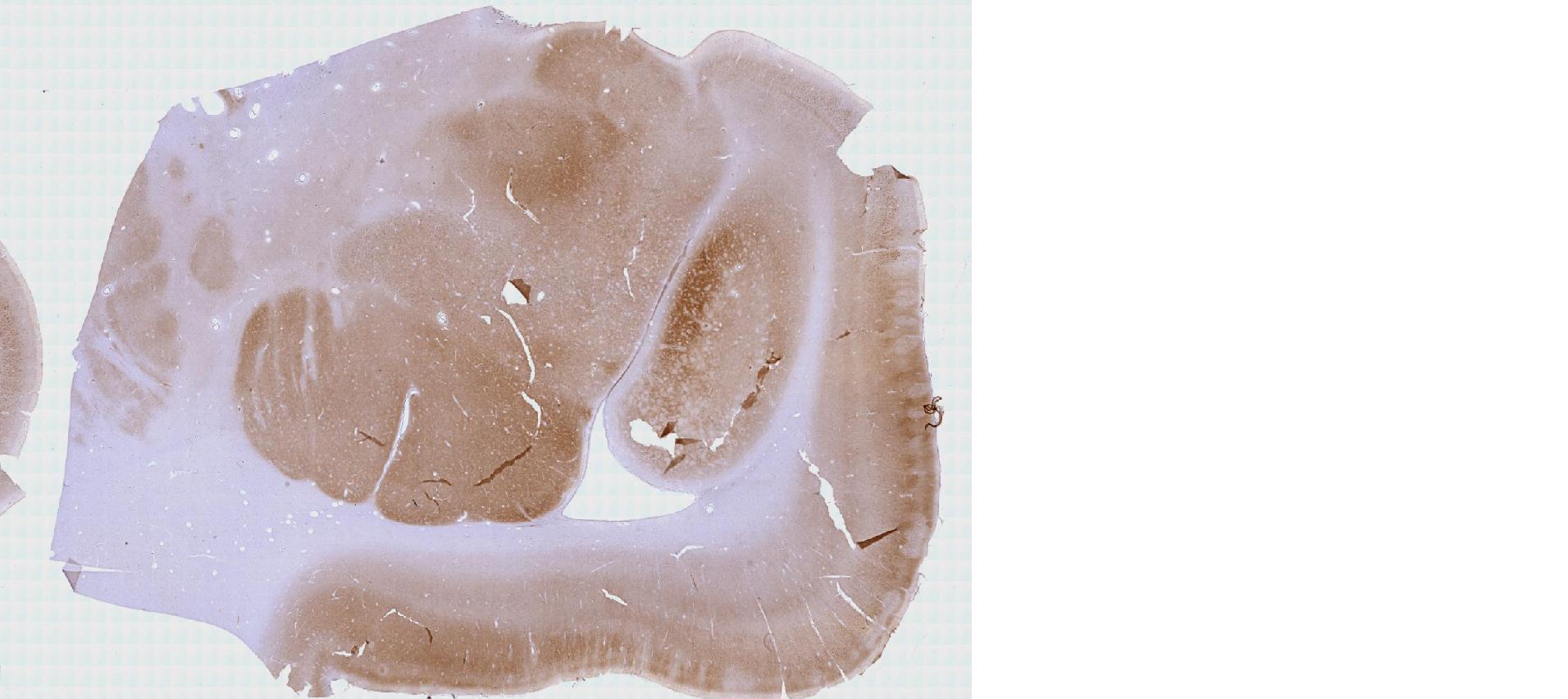

Supplement: Supplementary file 1 [file Presentation_1.ZIP › shank3-immunohistochemistry/case-5-counterstain/8-amygdalar complex-counterstain.jpg]

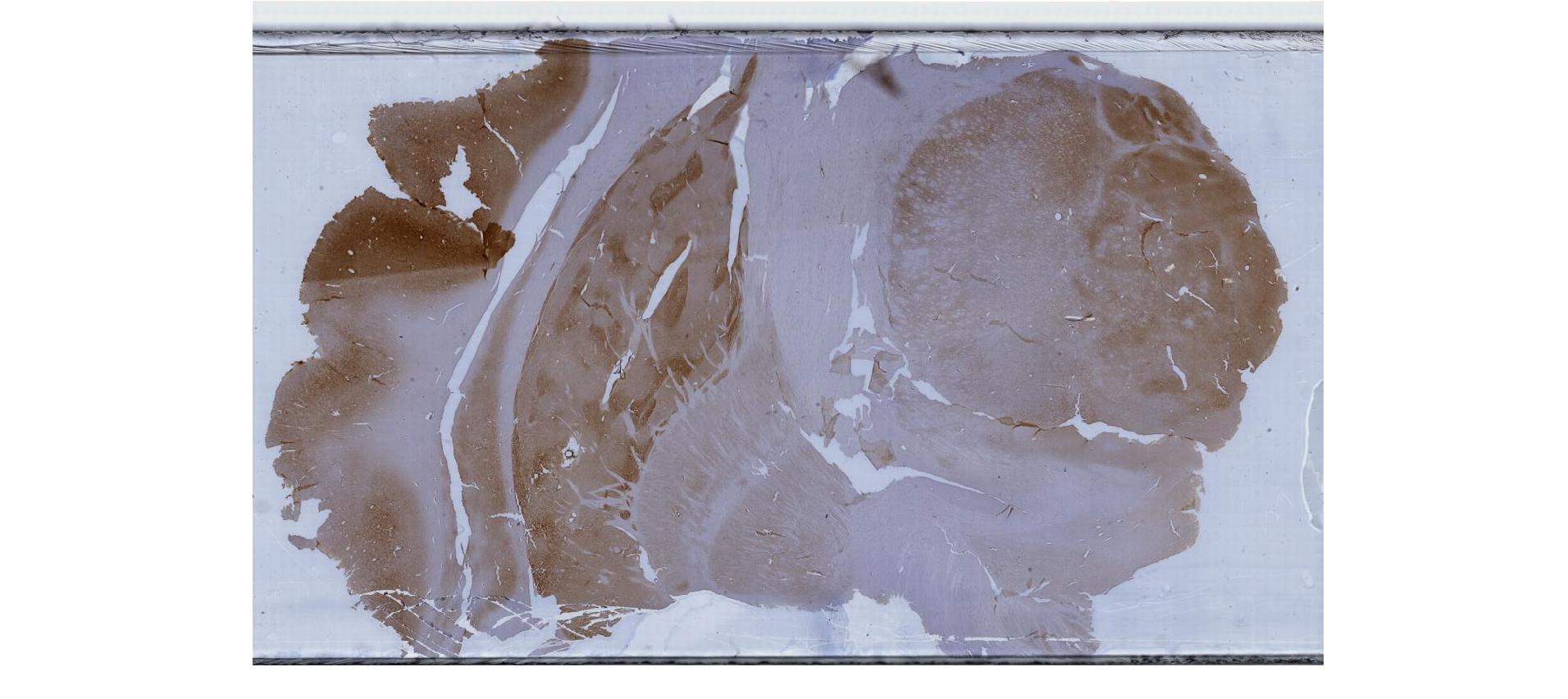

Supplement: Supplementary file 1 [file Presentation_1.ZIP › shank3-immunohistochemistry/case-5-counterstain/9-basal ganglia and diencephalon-counterstain.jpg]

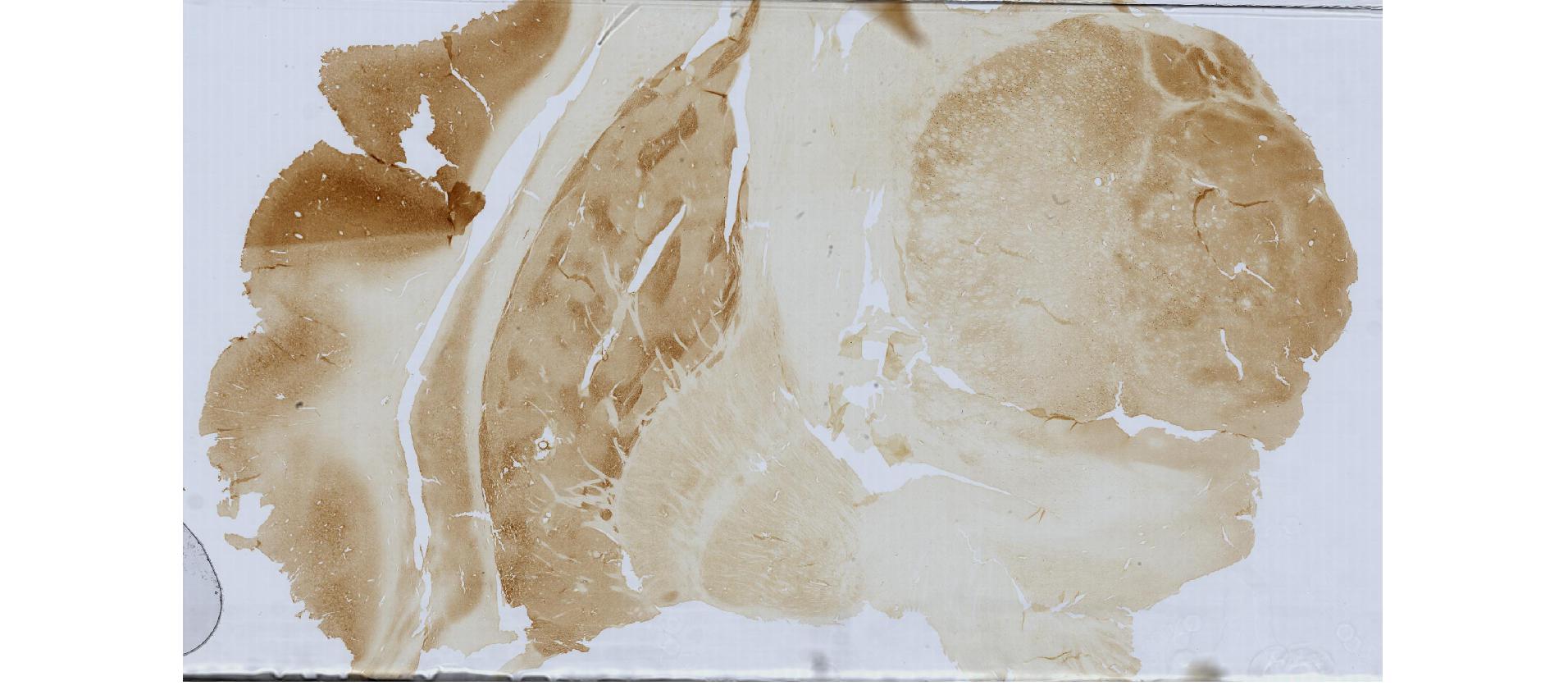

Supplement: Supplementary file 1 [file Presentation_1.ZIP › shank3-immunohistochemistry/case-5-counterstain/9-basal ganglia and diencephalon.jpg]

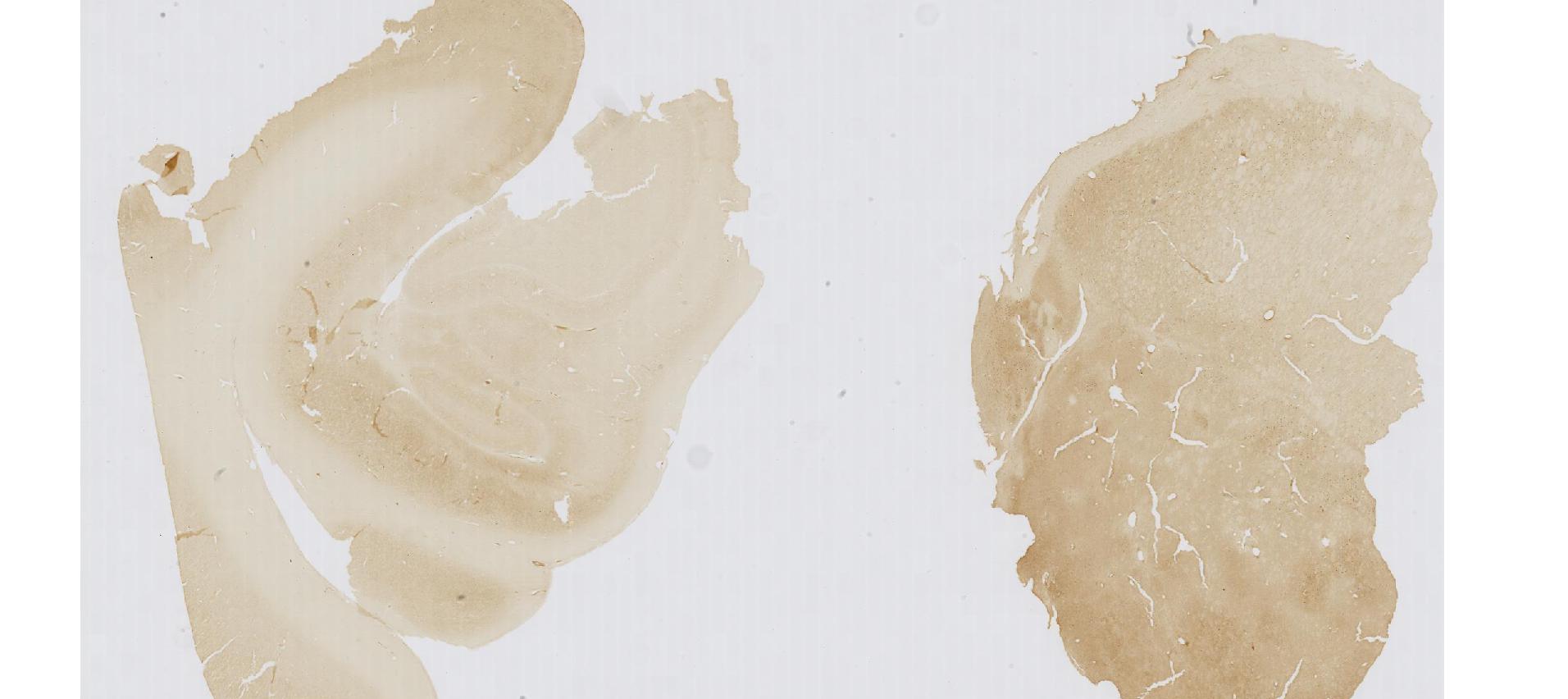

Supplement: Supplementary file 1 [file Presentation_1.ZIP › shank3-immunohistochemistry/case-6/amygdalar +basal ganglia.jpg]

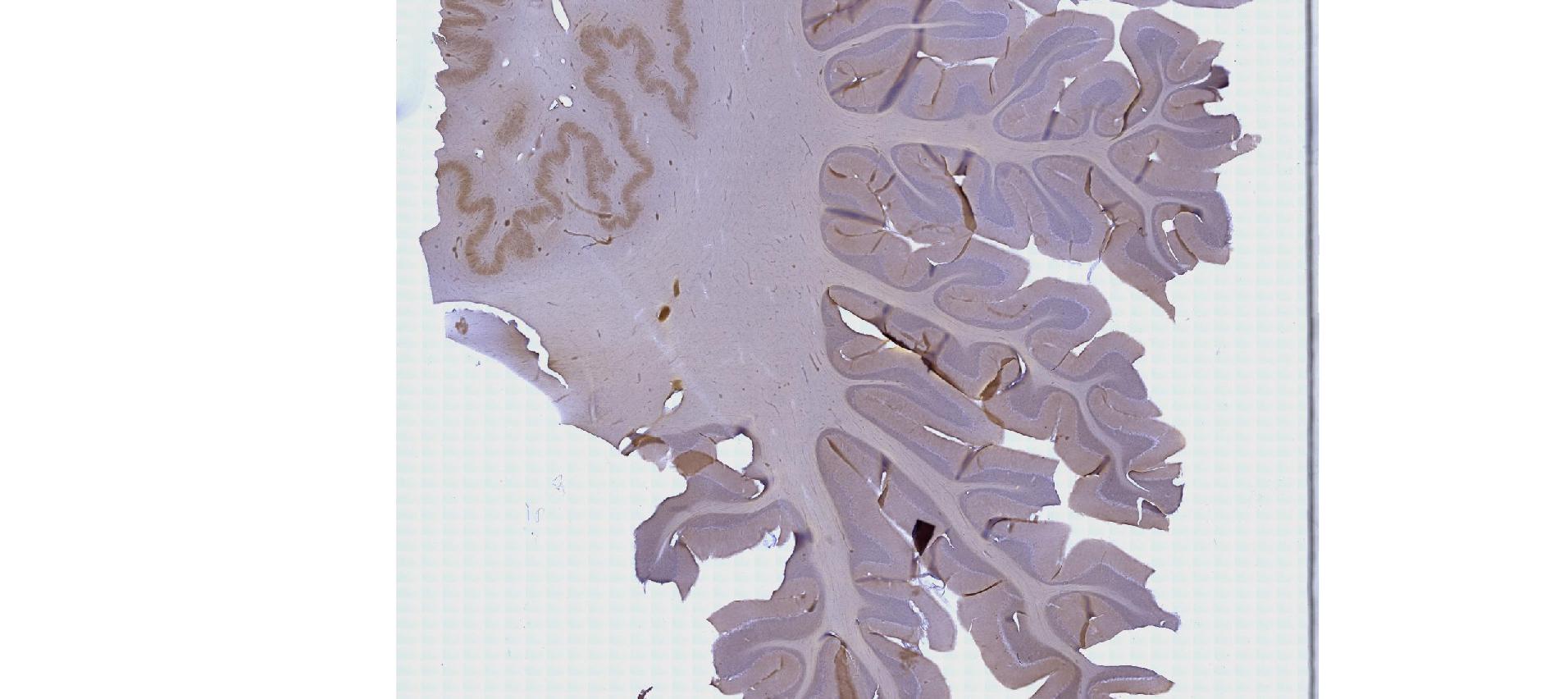

Supplement: Supplementary file 1 [file Presentation_1.ZIP › shank3-immunohistochemistry/case-6/cerebellum.jpg]

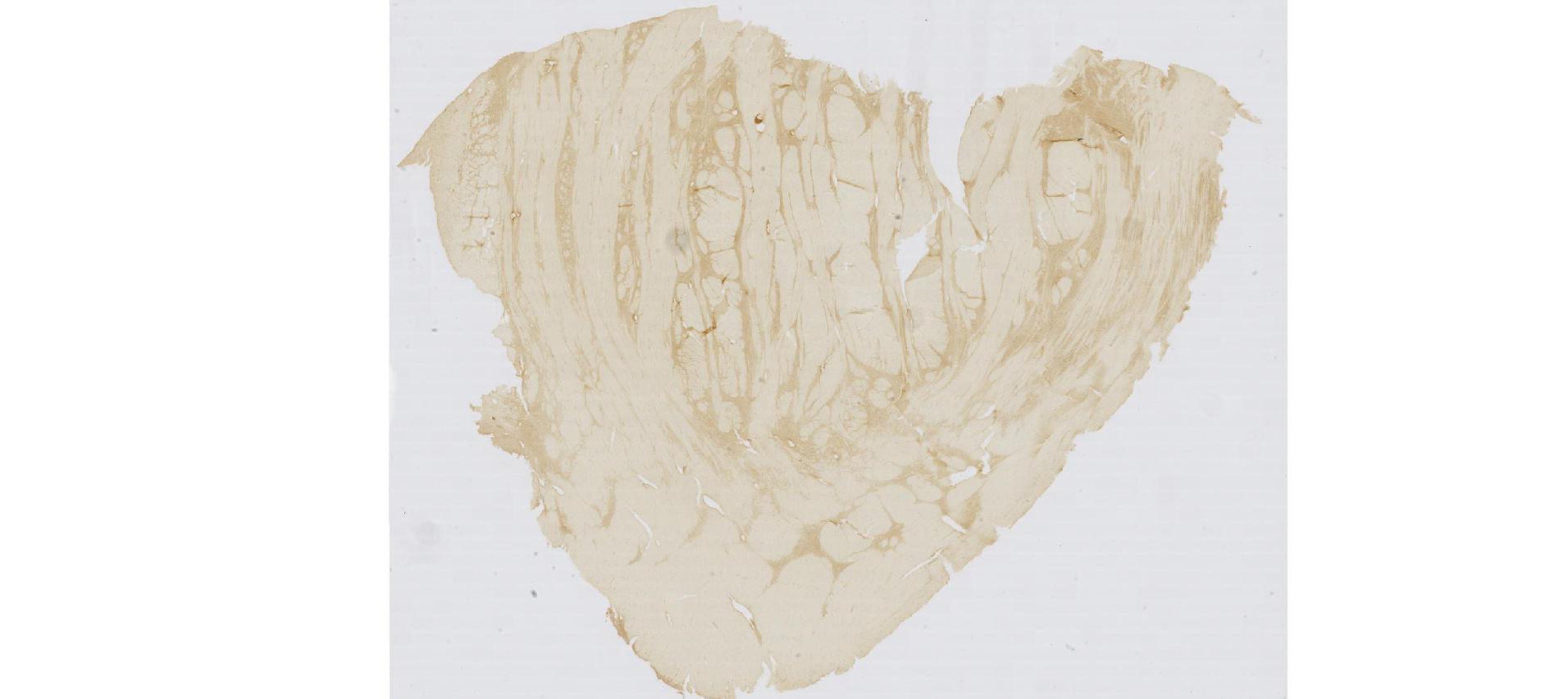

Supplement: Supplementary file 1 [file Presentation_1.ZIP › shank3-immunohistochemistry/case-6/pons.jpg]

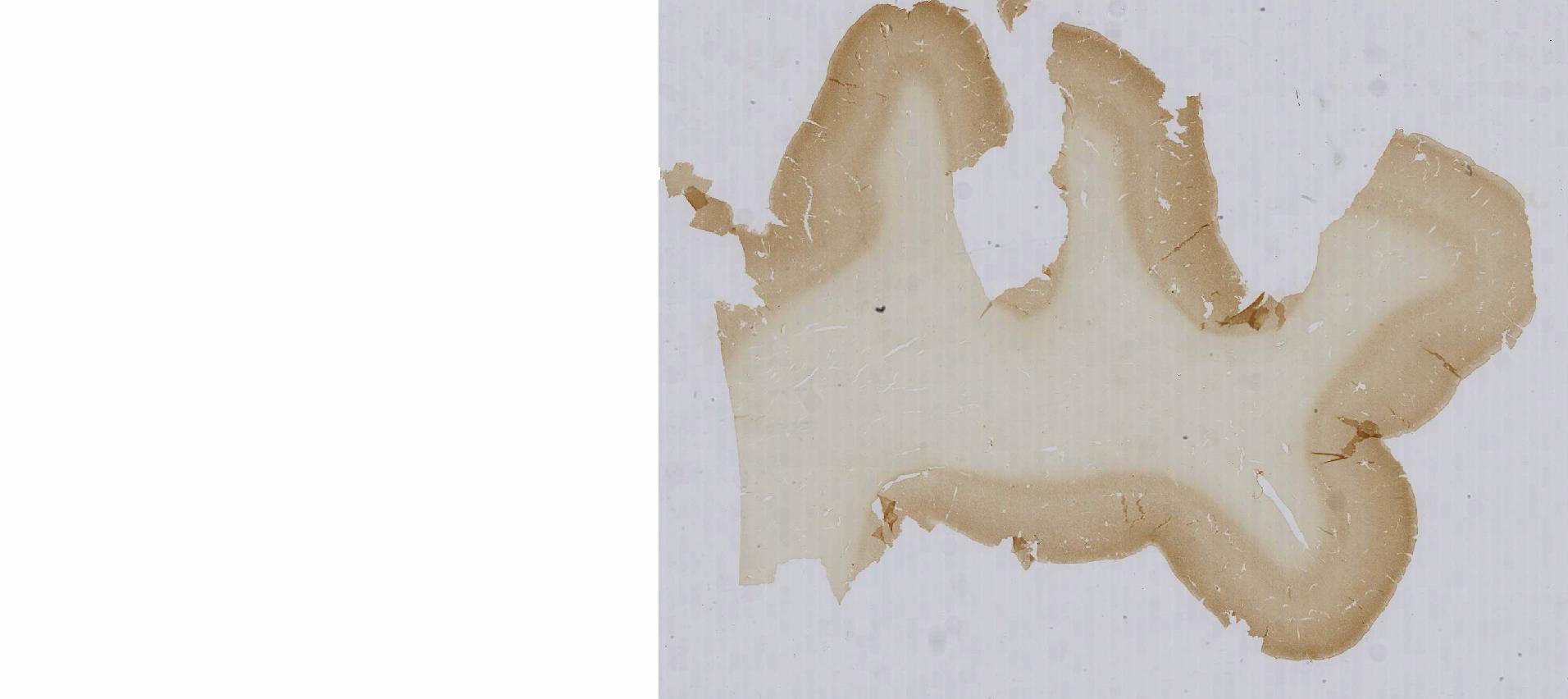

Supplement: Supplementary file 1 [file Presentation_1.ZIP › shank3-immunohistochemistry/case-6/prefrontal cortex.jpg]

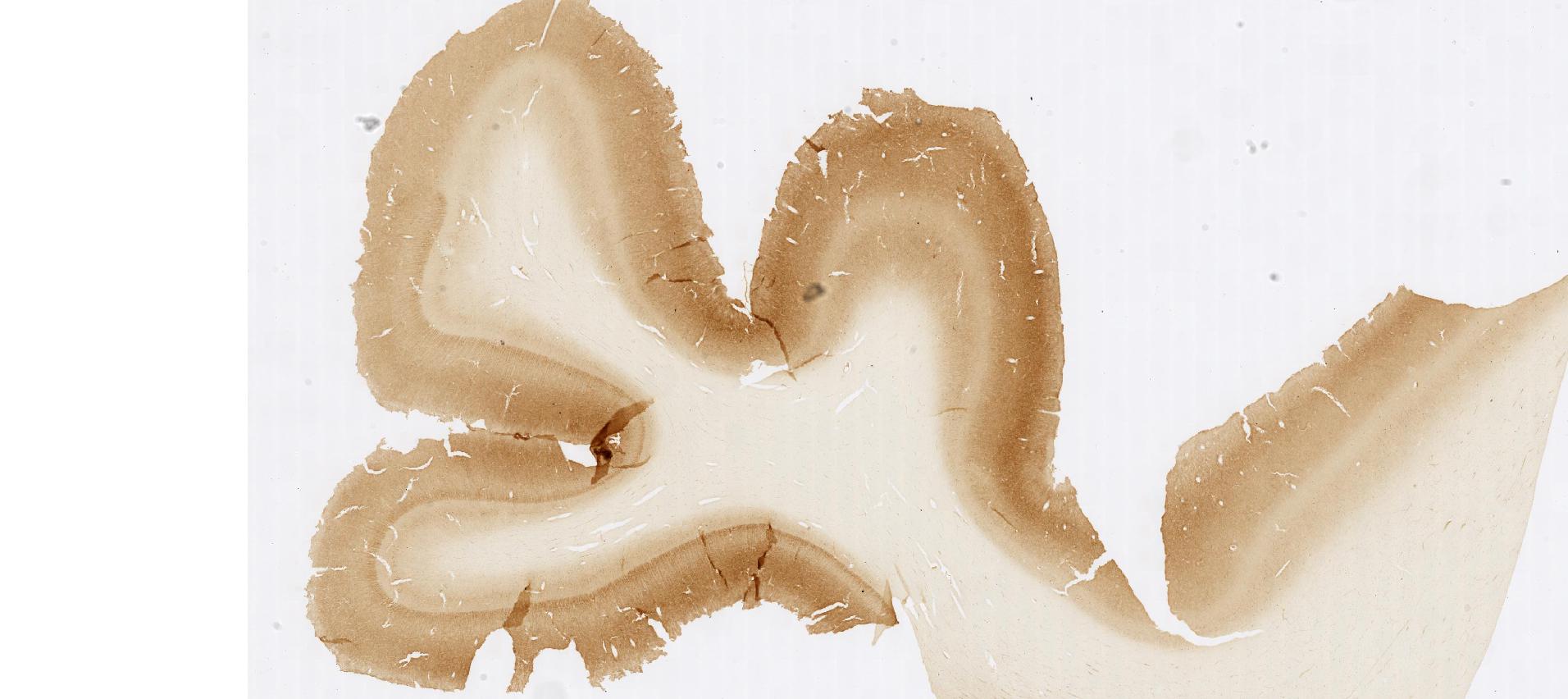

Supplement: Supplementary file 1 [file Presentation_1.ZIP › shank3-immunohistochemistry/case-6/visual cortex.jpg]

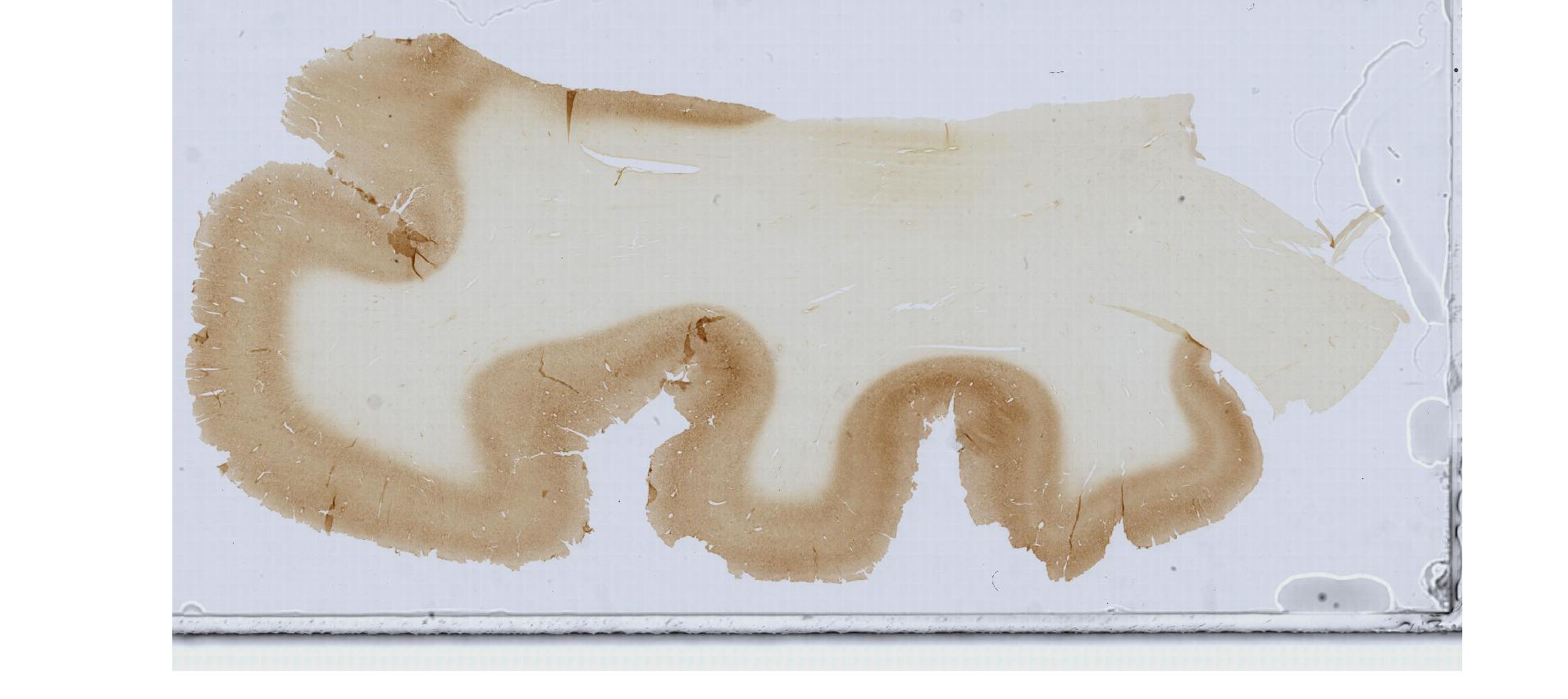

Supplement: Supplementary file 1 [file Presentation_1.ZIP › shank3-immunohistochemistry/case-7/anterior cingulate neocortex.jpg]

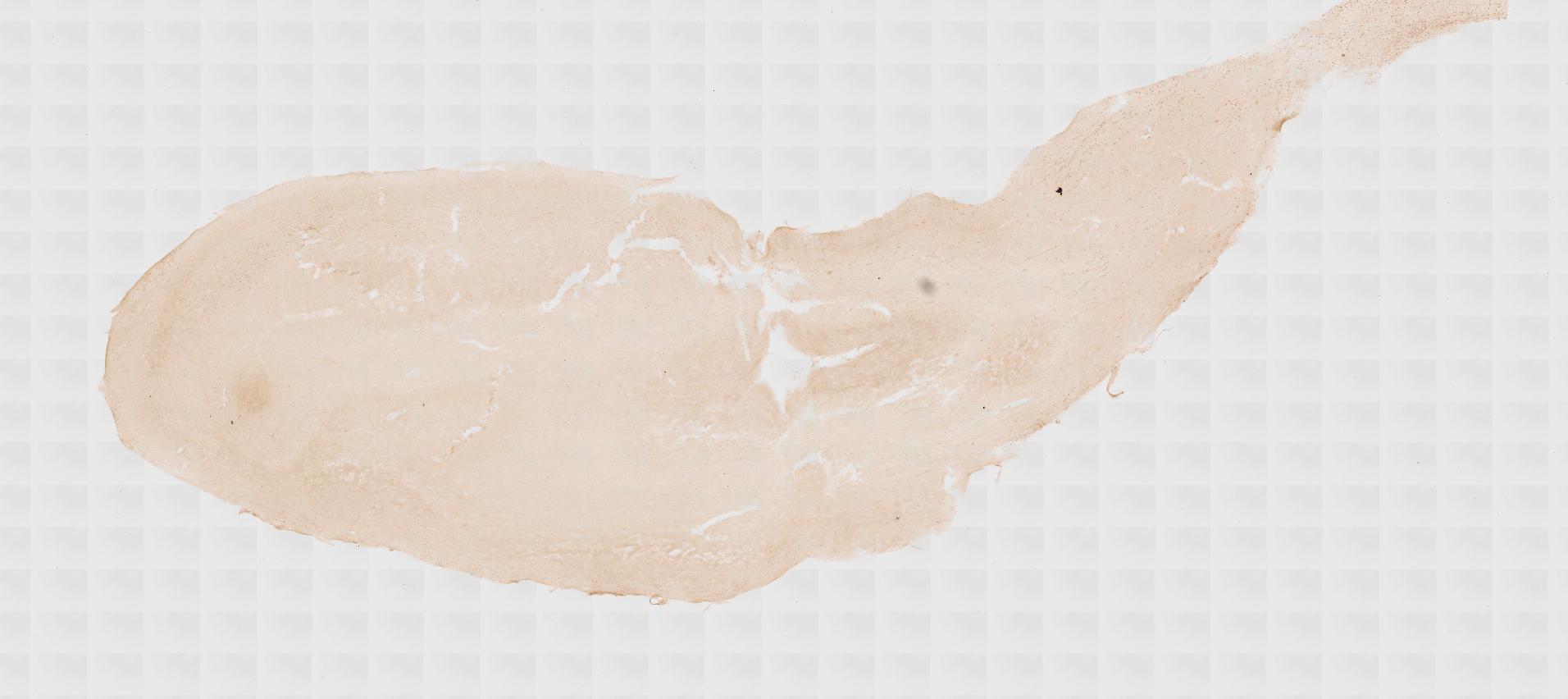

Supplement: Supplementary file 1 [file Presentation_1.ZIP › shank3-immunohistochemistry/case-7/olfactory bulb.jpg]

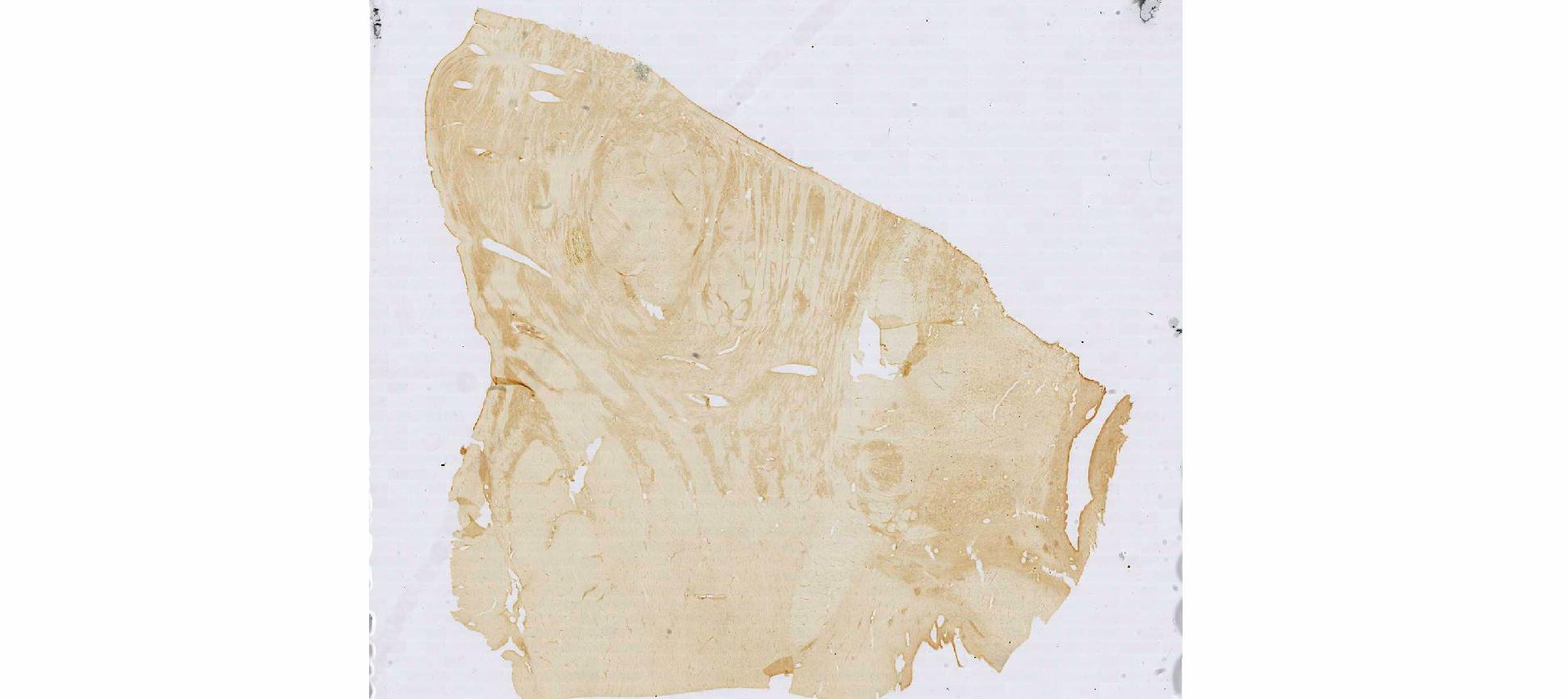

Supplement: Supplementary file 1 [file Presentation_1.ZIP › shank3-immunohistochemistry/case-7/pons.jpg]

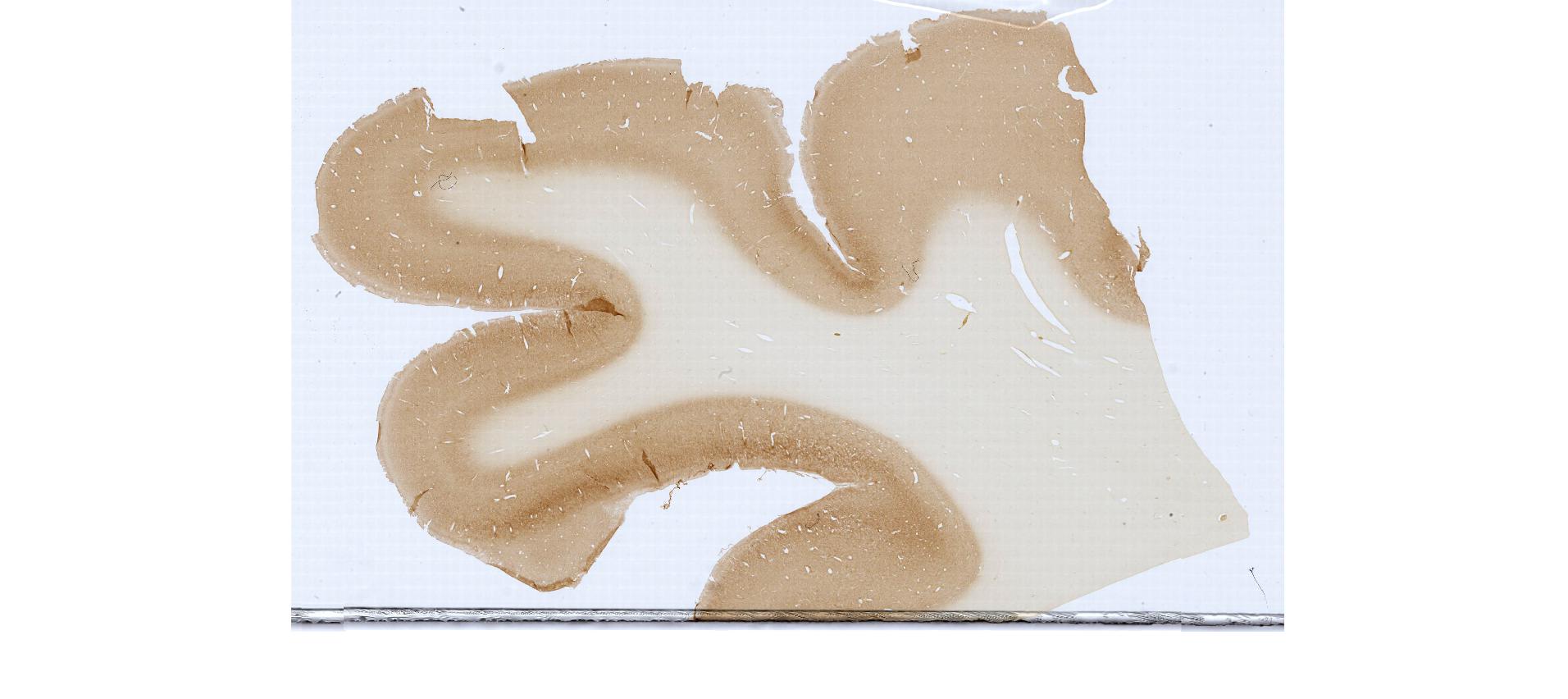

Supplement: Supplementary file 1 [file Presentation_1.ZIP › shank3-immunohistochemistry/case-7/prefrontal cortex.jpg]

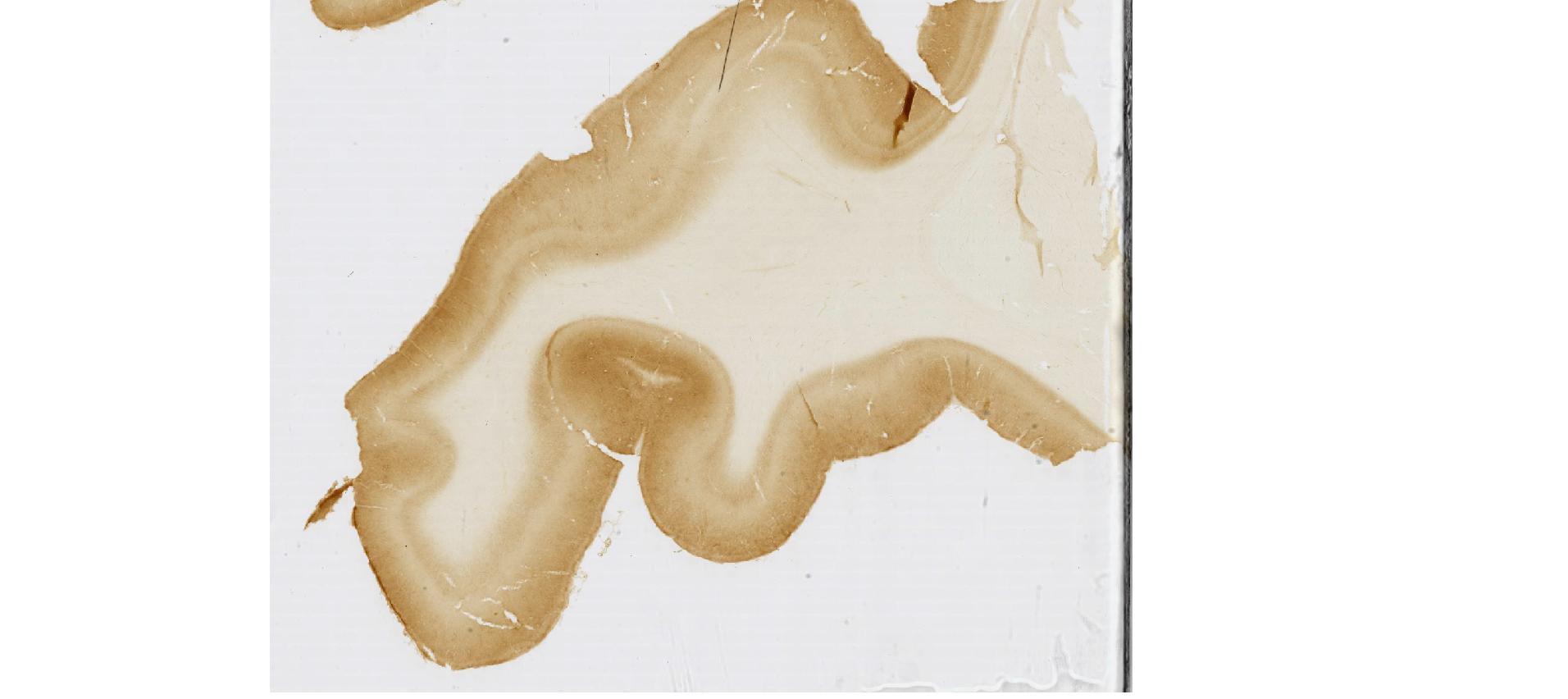

Supplement: Supplementary file 1 [file Presentation_1.ZIP › shank3-immunohistochemistry/case-7/visual cortex.jpg]

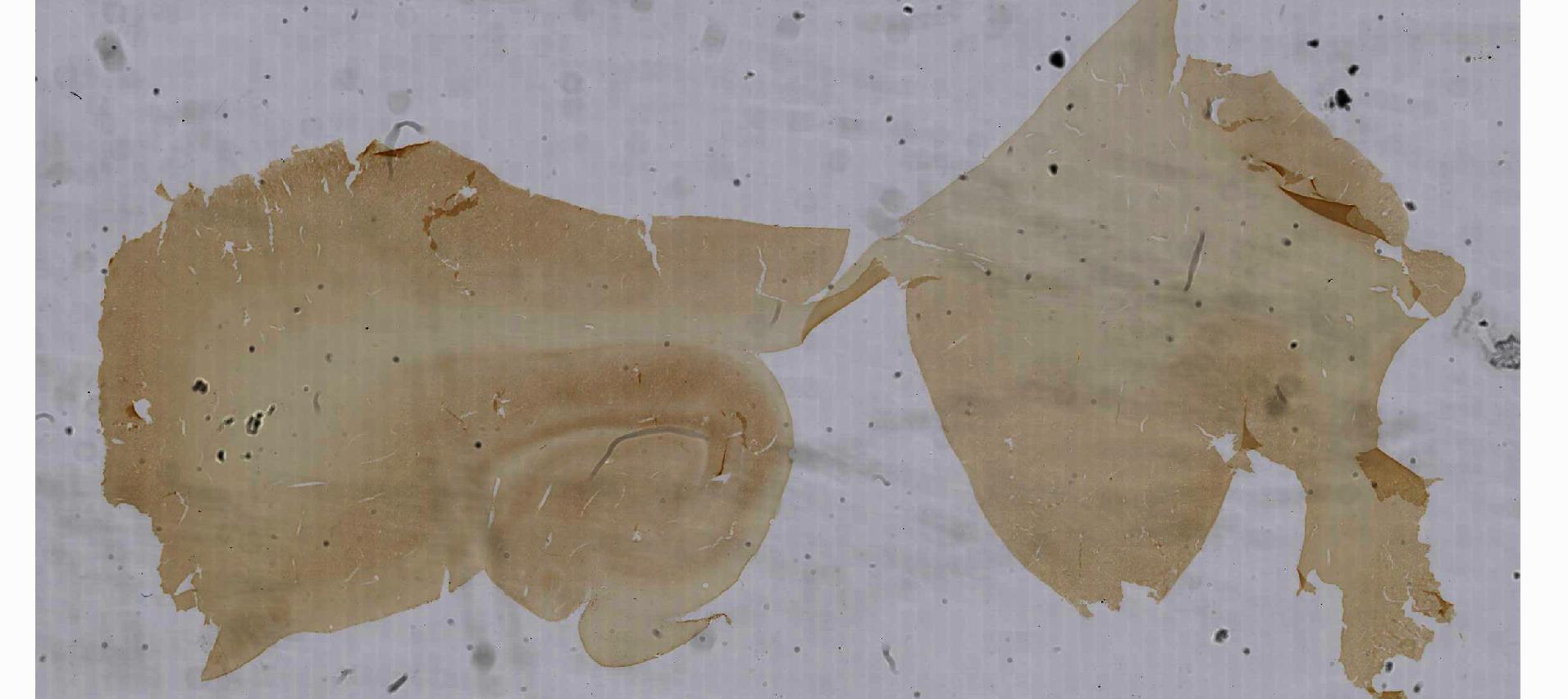

Supplement: Supplementary file 1 [file Presentation_1.ZIP › shank3-immunohistochemistry/case-9/hippocampal formation.jpg]

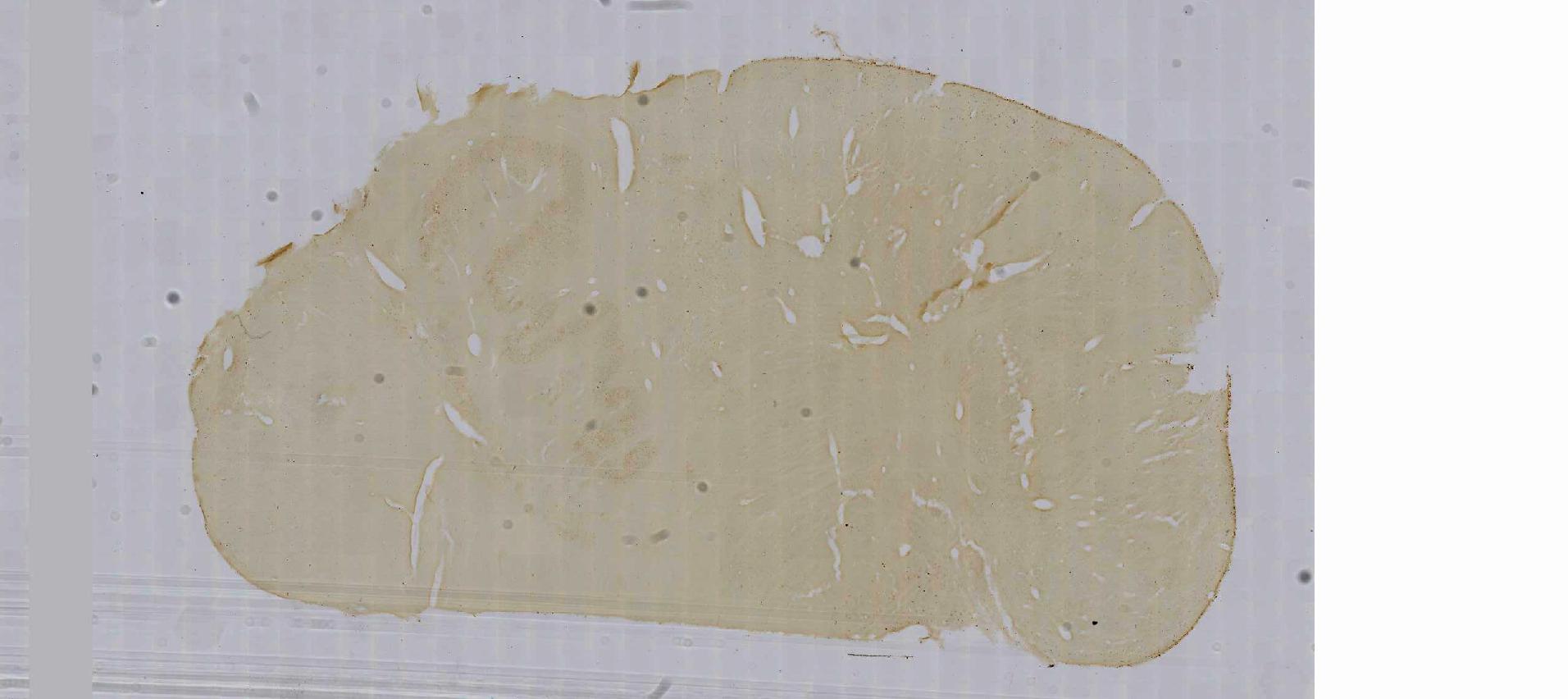

Supplement: Supplementary file 1 [file Presentation_1.ZIP › shank3-immunohistochemistry/case-9/medulla oblongata.jpg]

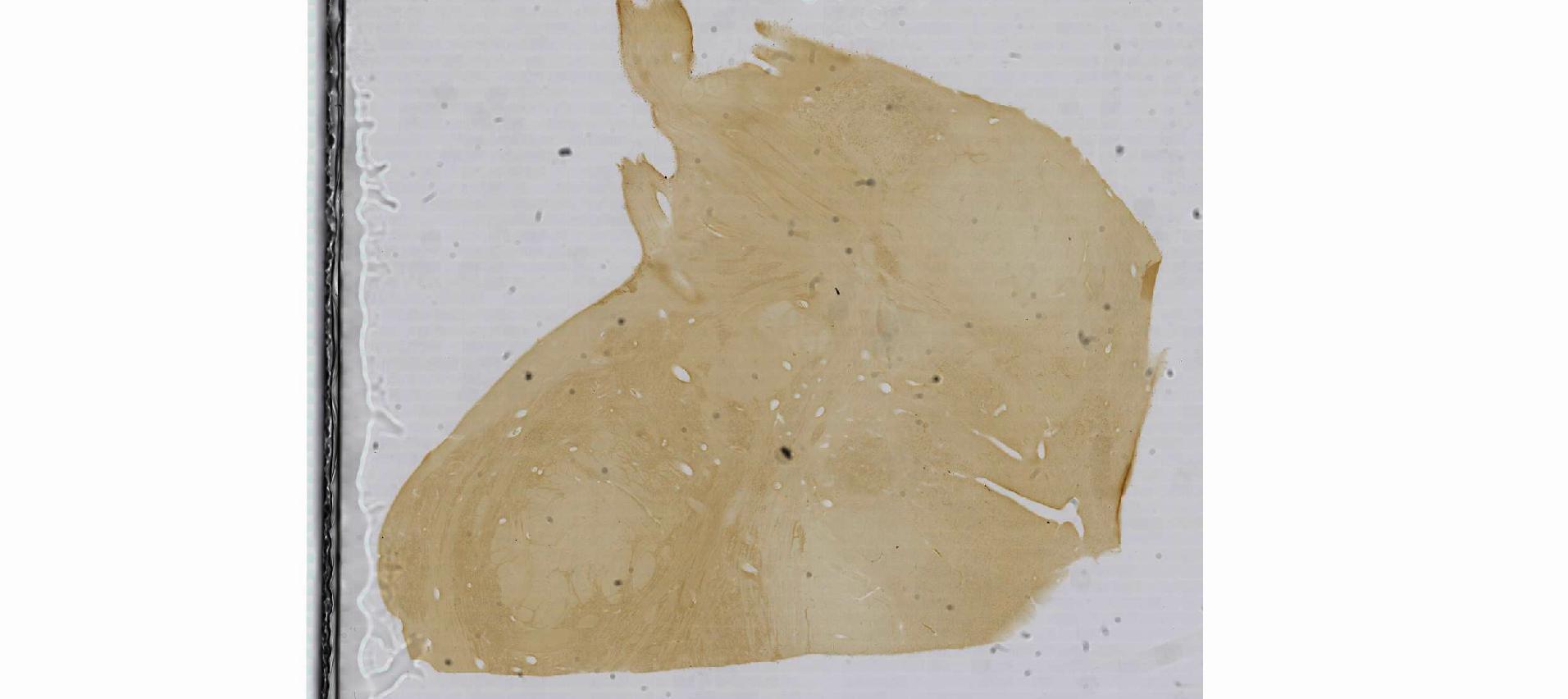

Supplement: Supplementary file 1 [file Presentation_1.ZIP › shank3-immunohistochemistry/case-9/pons.jpg]

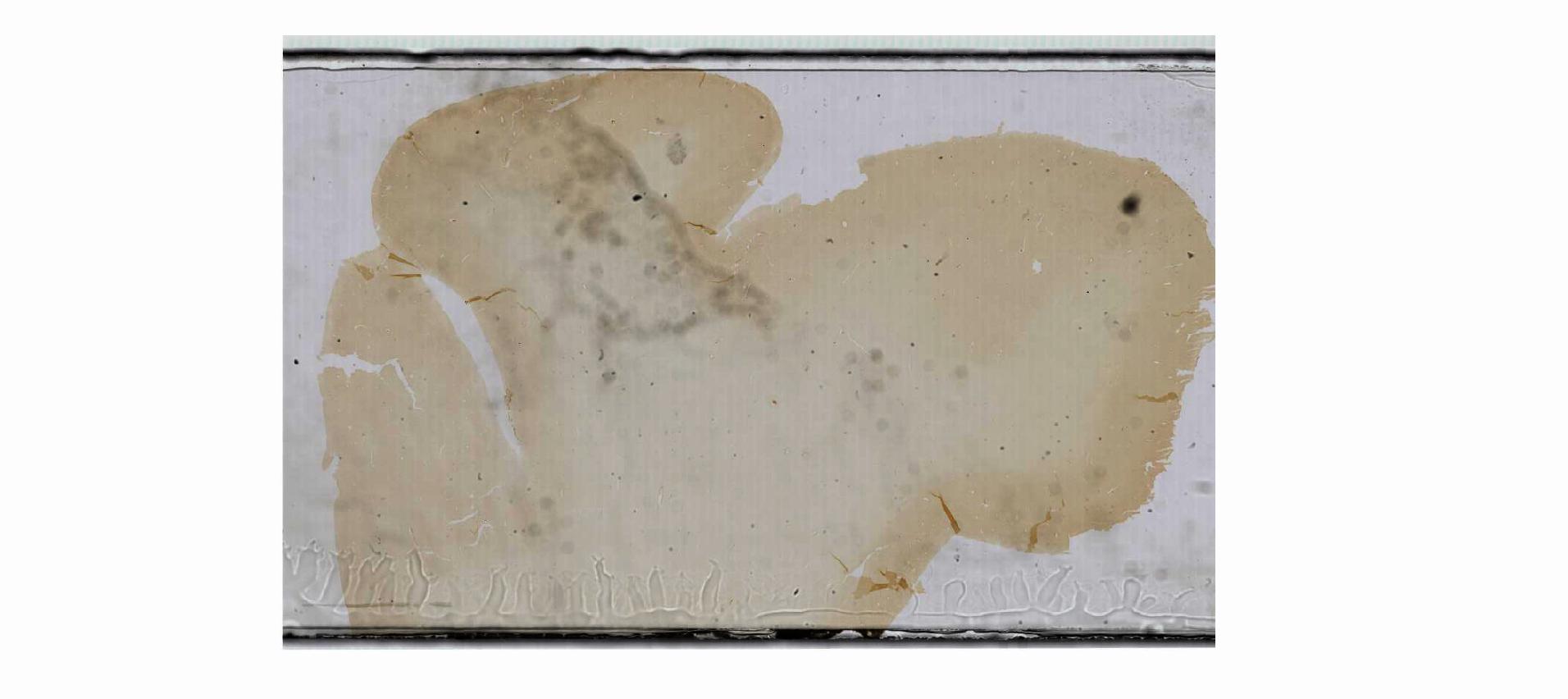

Supplement: Supplementary file 1 [file Presentation_1.ZIP › shank3-immunohistochemistry/case-9/precentral cortex.jpg]

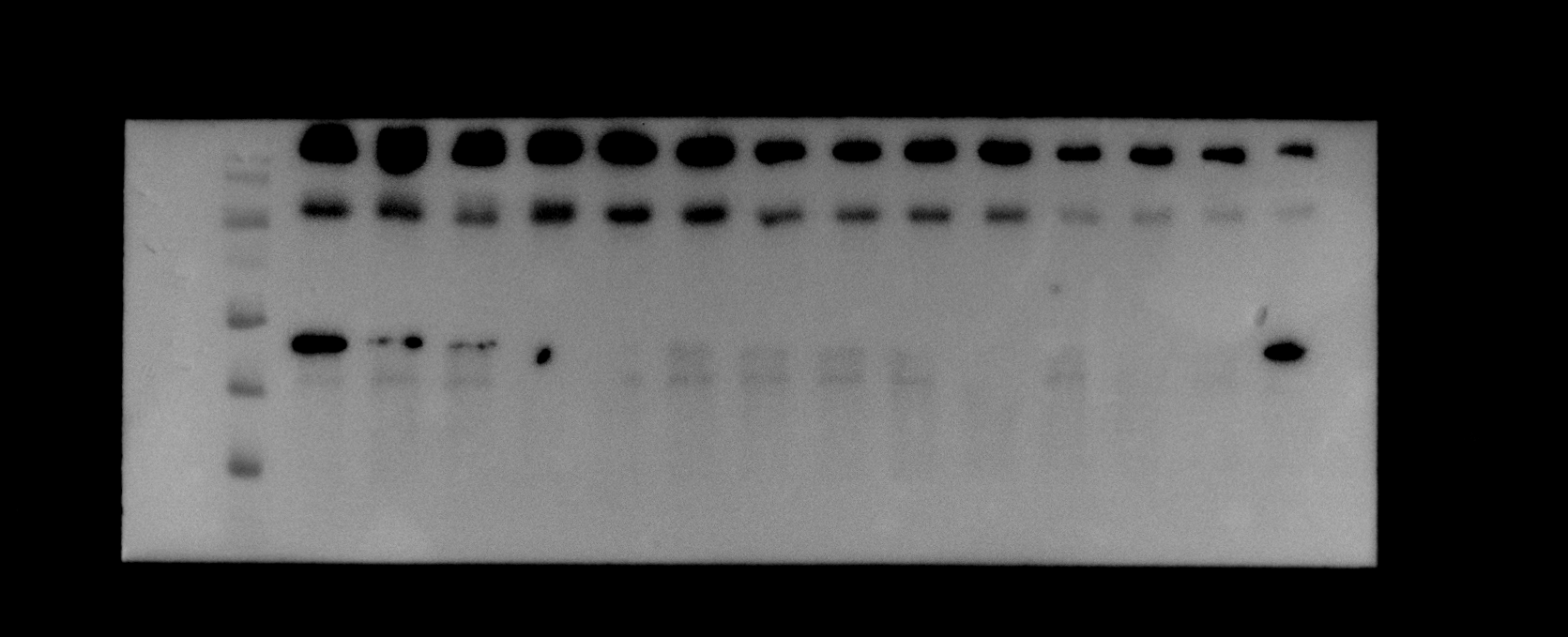

Supplement: Supplementary file 2 [file Presentation_2.ZIP › 2021-07-25-different exposure time-WB original images- full blot/Figure 14B- full wb membrane of shank3 of different exposure time/10_8bit.tif]

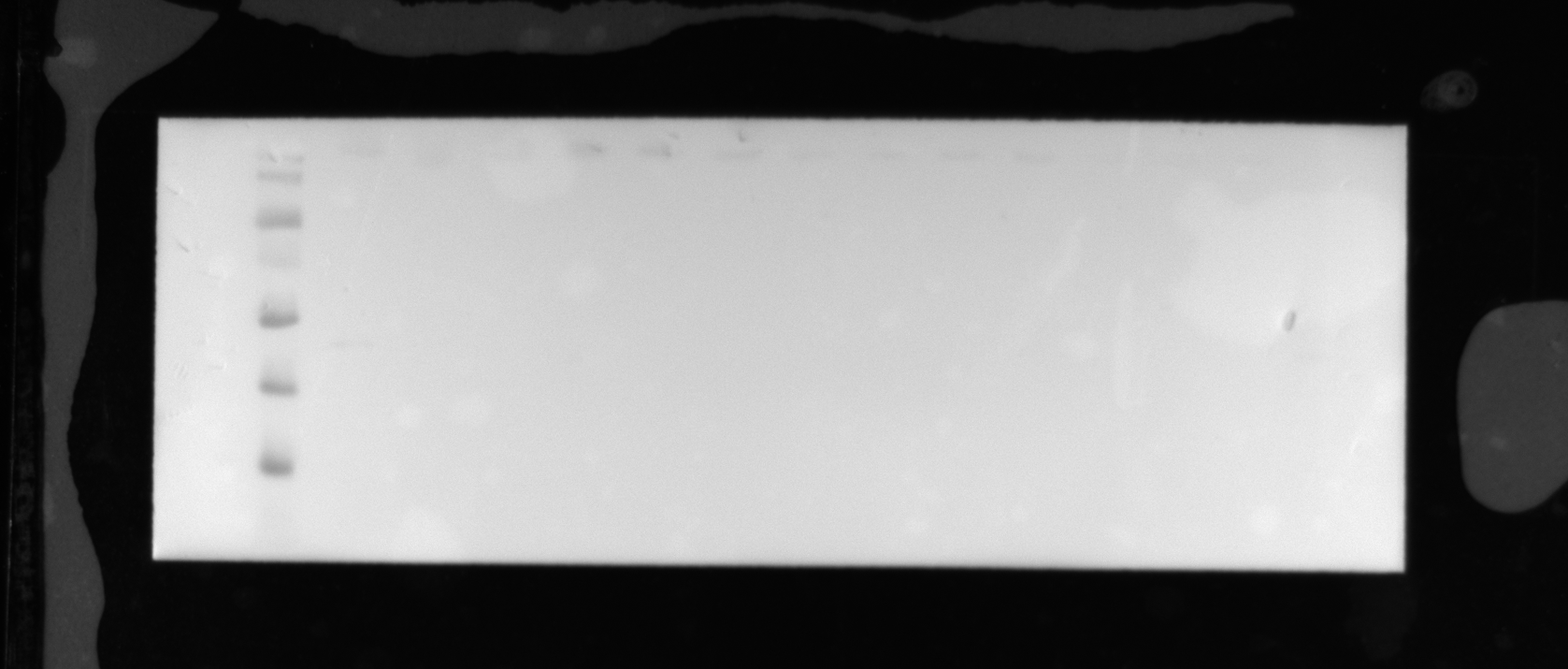

Supplement: Supplementary file 2 [file Presentation_2.ZIP › 2021-07-25-different exposure time-WB original images- full blot/Figure 14B- full wb membrane of shank3 of different exposure time/1_8bit.tif]

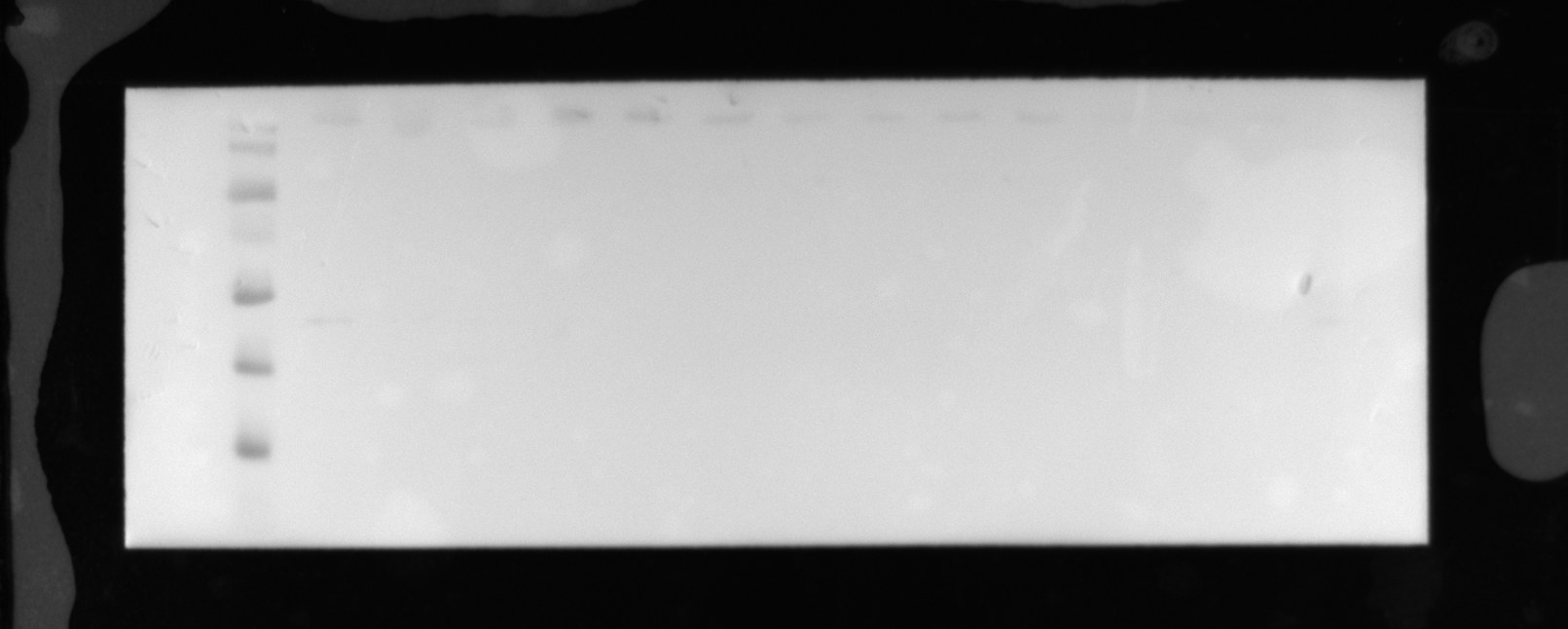

Supplement: Supplementary file 2 [file Presentation_2.ZIP › 2021-07-25-different exposure time-WB original images- full blot/Figure 14B- full wb membrane of shank3 of different exposure time/2_8bit.tif]

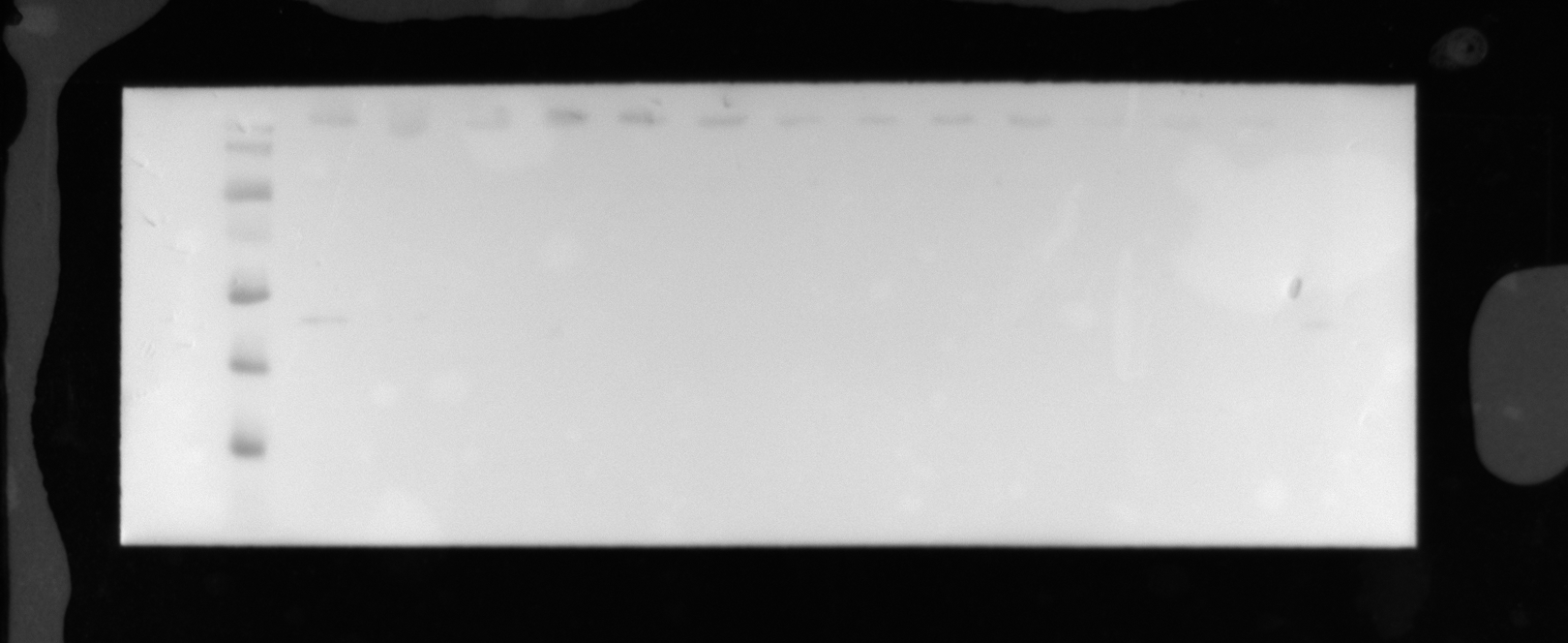

Supplement: Supplementary file 2 [file Presentation_2.ZIP › 2021-07-25-different exposure time-WB original images- full blot/Figure 14B- full wb membrane of shank3 of different exposure time/3_8bit.tif]

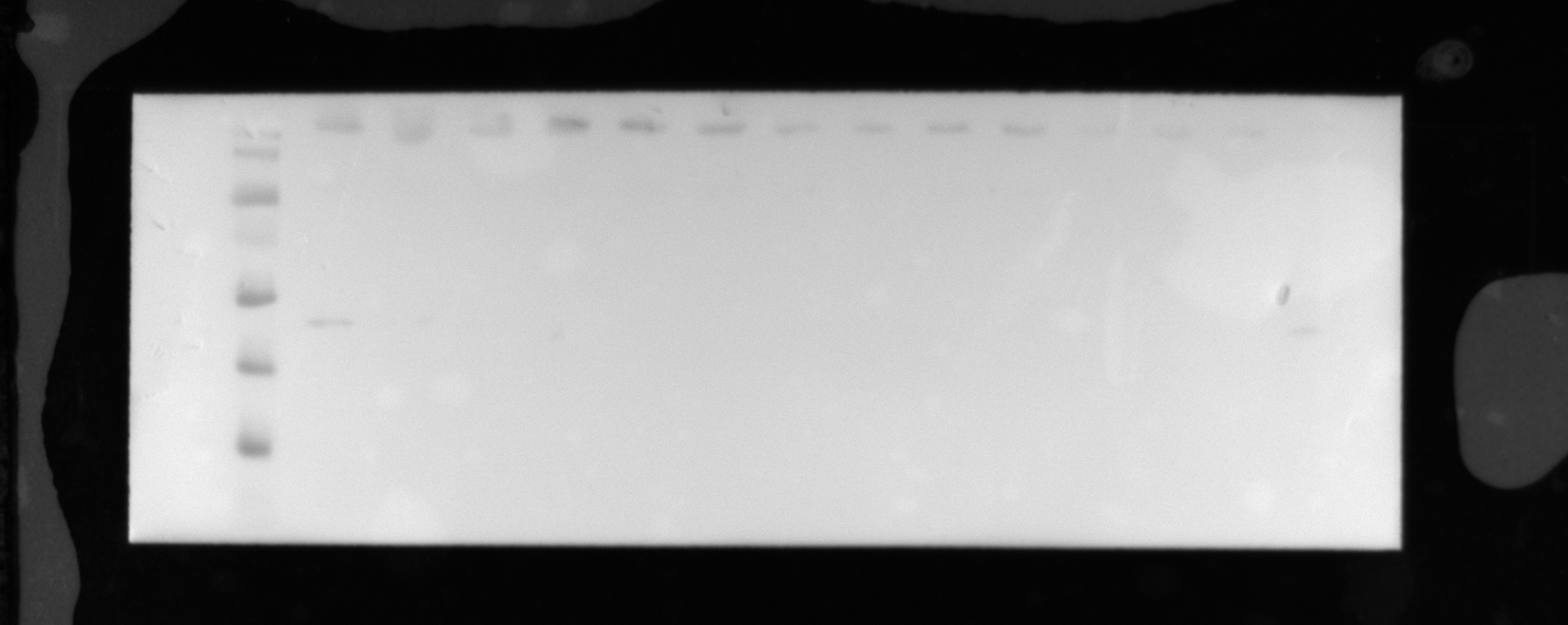

Supplement: Supplementary file 2 [file Presentation_2.ZIP › 2021-07-25-different exposure time-WB original images- full blot/Figure 14B- full wb membrane of shank3 of different exposure time/4_8bit.tif]

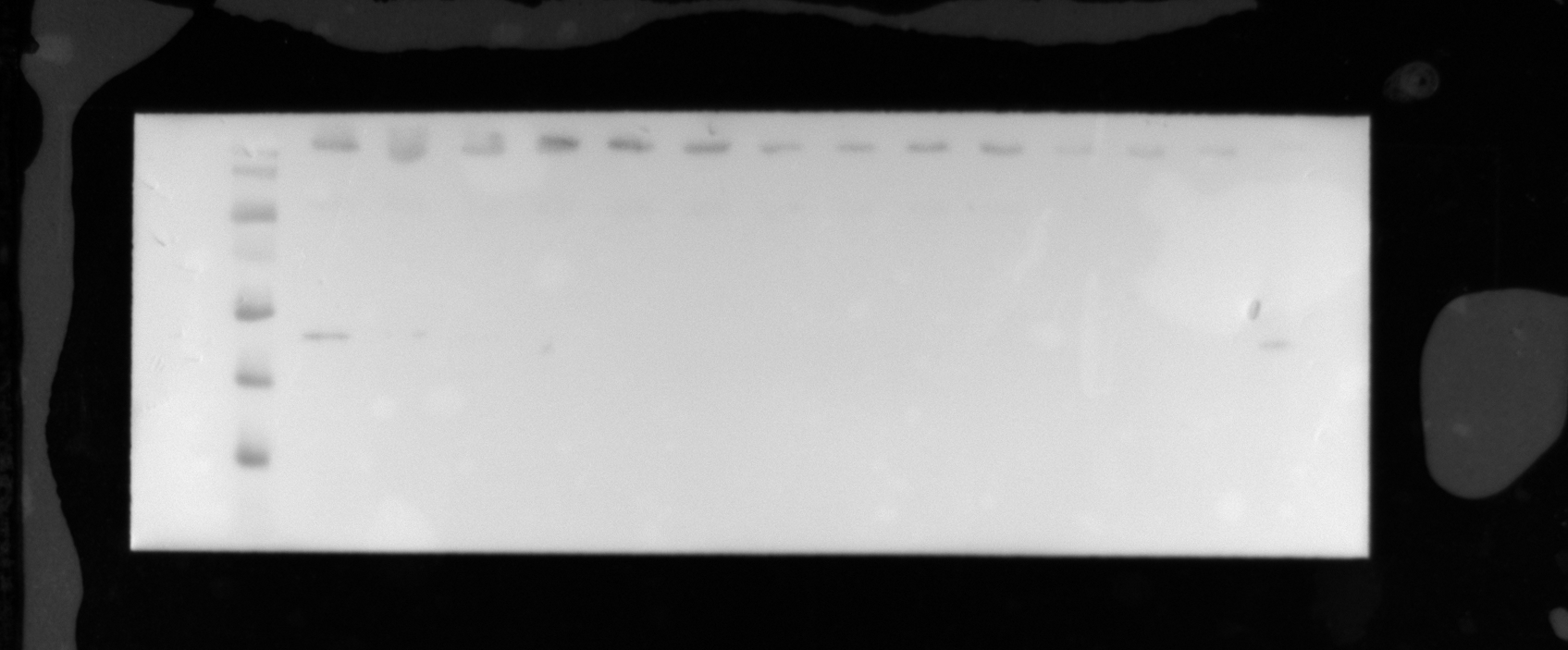

Supplement: Supplementary file 2 [file Presentation_2.ZIP › 2021-07-25-different exposure time-WB original images- full blot/Figure 14B- full wb membrane of shank3 of different exposure time/5_8bit.tif]

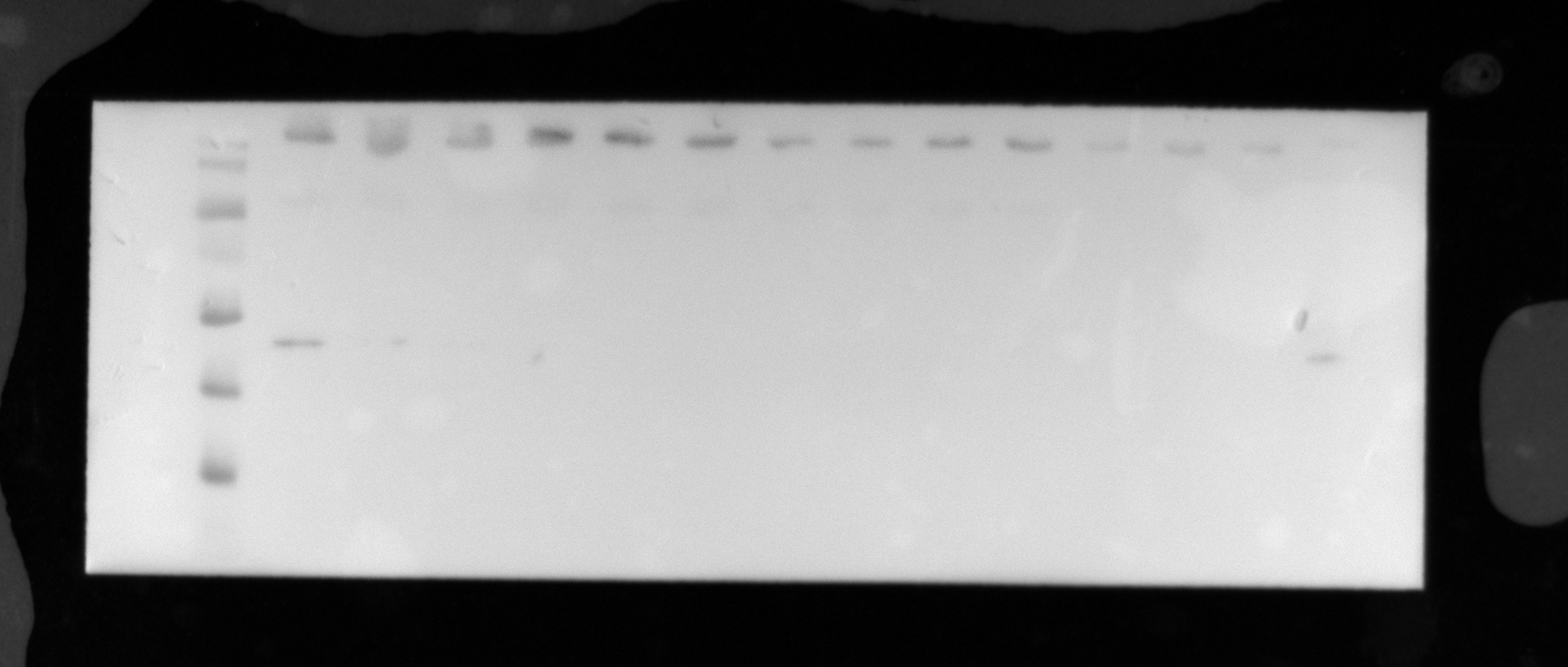

Supplement: Supplementary file 2 [file Presentation_2.ZIP › 2021-07-25-different exposure time-WB original images- full blot/Figure 14B- full wb membrane of shank3 of different exposure time/6_8bit.tif]

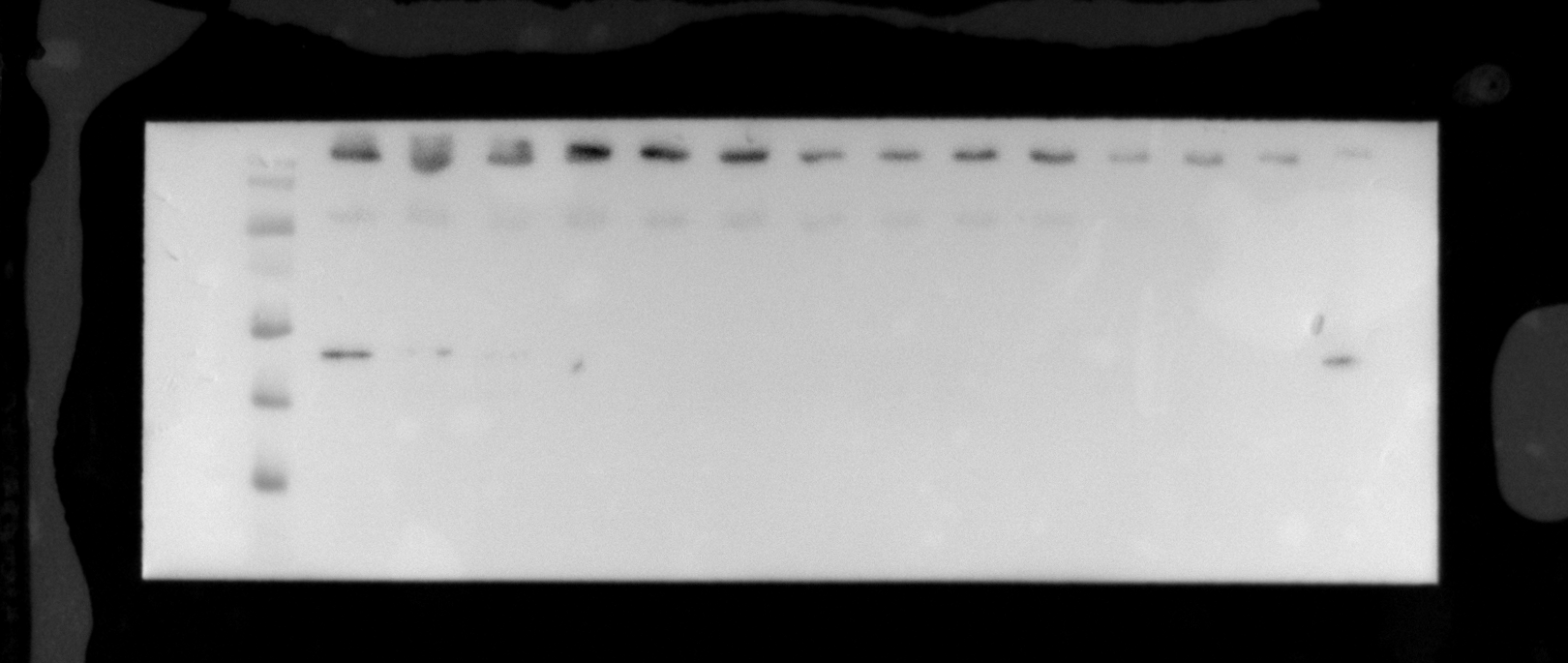

Supplement: Supplementary file 2 [file Presentation_2.ZIP › 2021-07-25-different exposure time-WB original images- full blot/Figure 14B- full wb membrane of shank3 of different exposure time/7_8bit.tif]

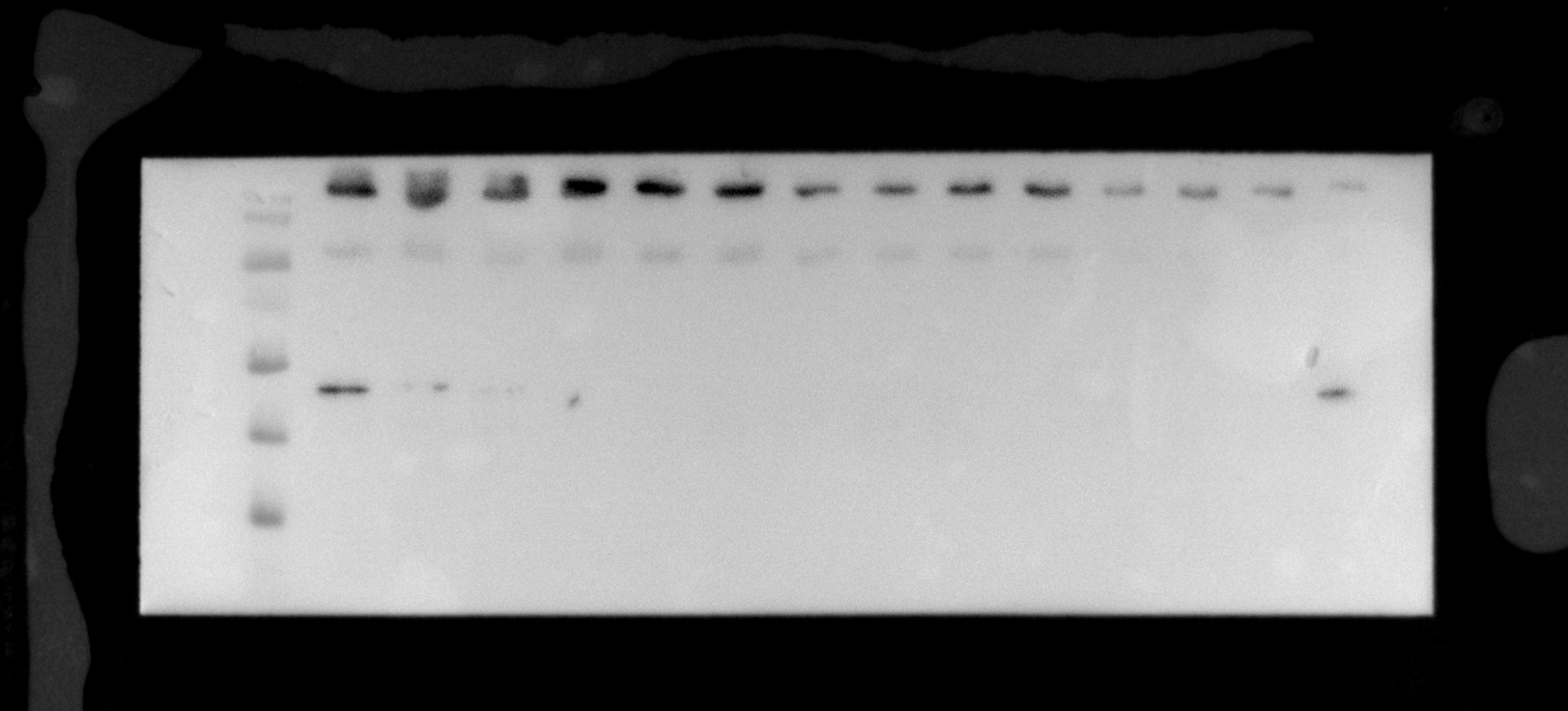

Supplement: Supplementary file 2 [file Presentation_2.ZIP › 2021-07-25-different exposure time-WB original images- full blot/Figure 14B- full wb membrane of shank3 of different exposure time/8_8bit.tif]

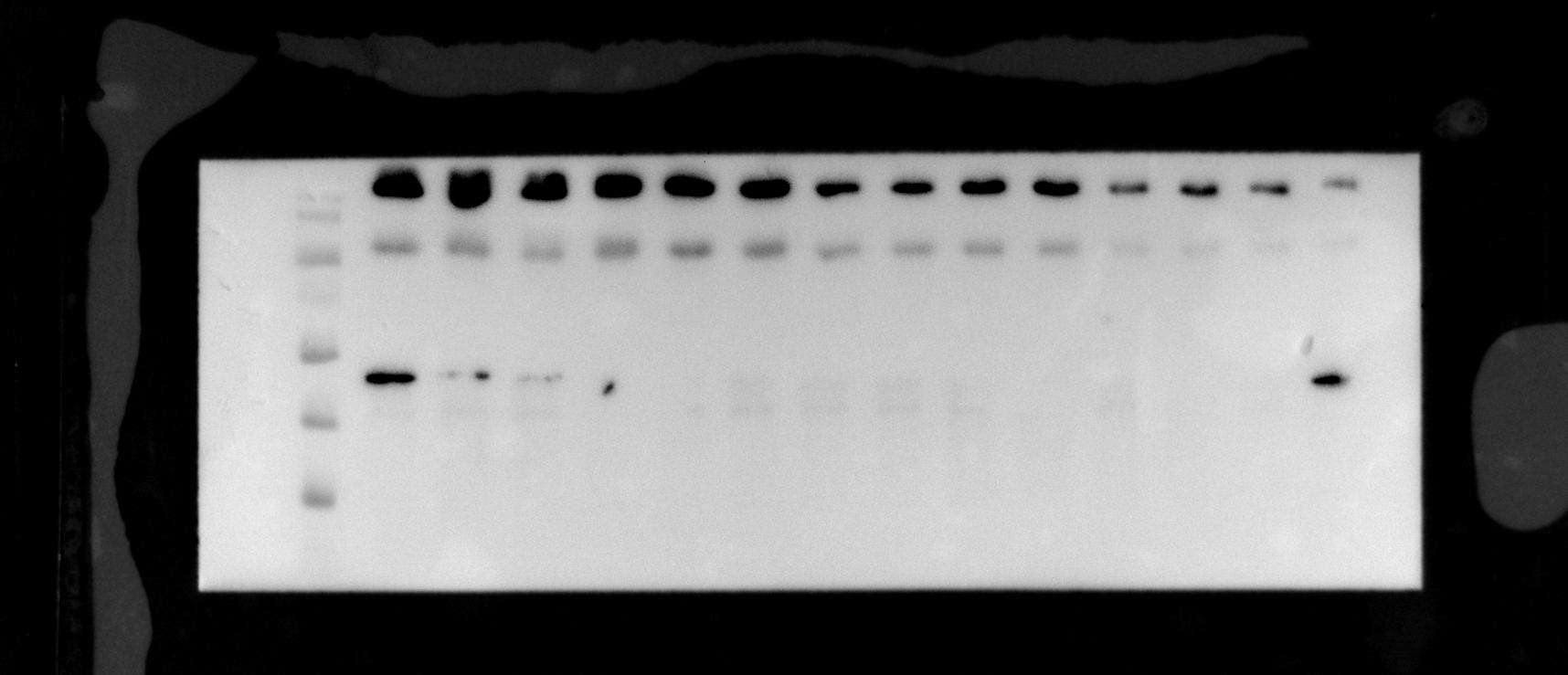

Supplement: Supplementary file 2 [file Presentation_2.ZIP › 2021-07-25-different exposure time-WB original images- full blot/Figure 14B- full wb membrane of shank3 of different exposure time/9_8bit.tif]

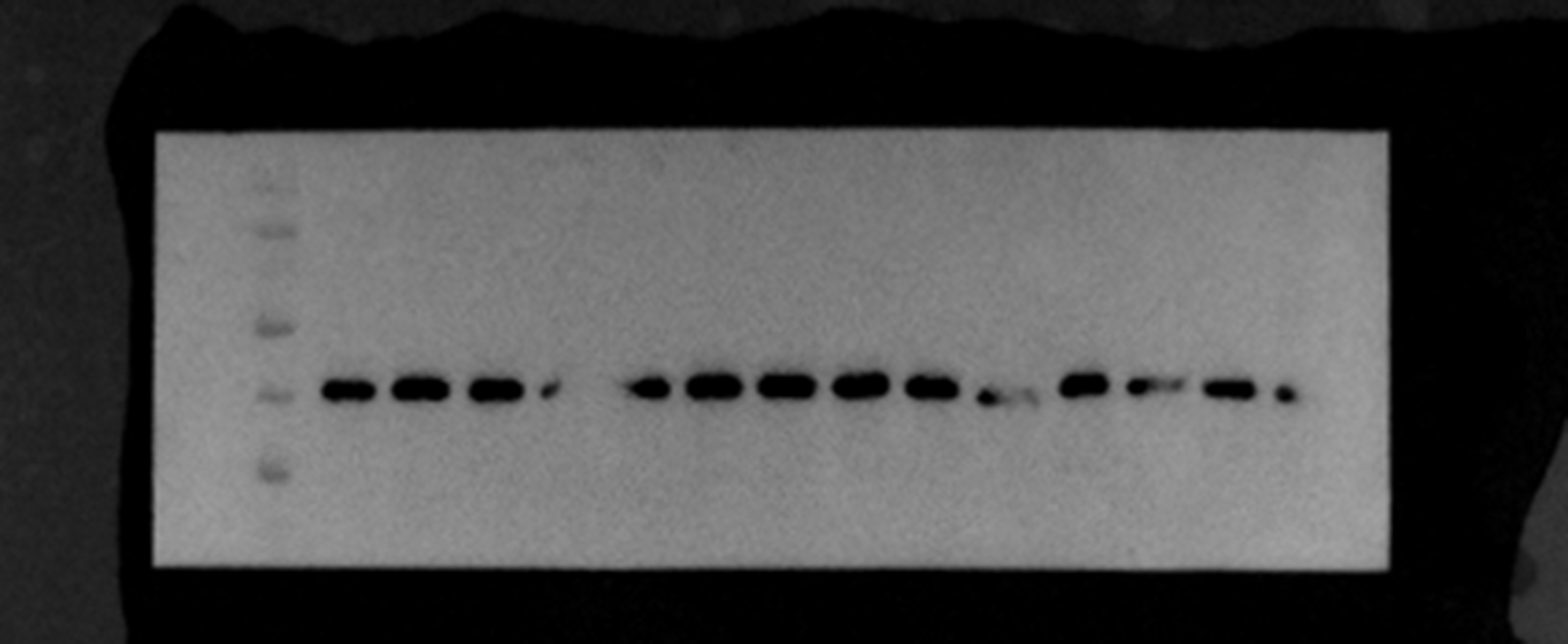

Supplement: Supplementary file 2 [file Presentation_2.ZIP › 2021-07-25-different exposure time-WB original images- full blot/Figure 14B- full wb membrane of shank3 of different exposure time/actin_8bit.tif]

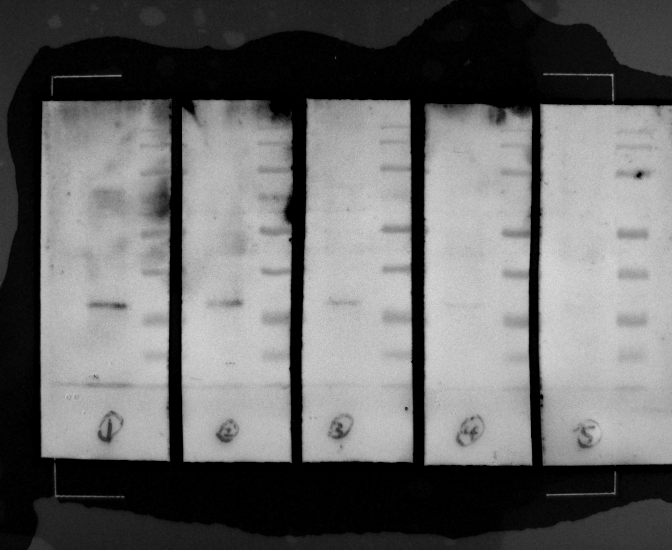

Supplement: Supplementary file 2 [file Presentation_2.ZIP › 2021-07-25-different exposure time-WB original images- full blot/Full wb membrane of syn/1-syn- from the label 1 to 5,the antibody concentration decreased gradually.tif]

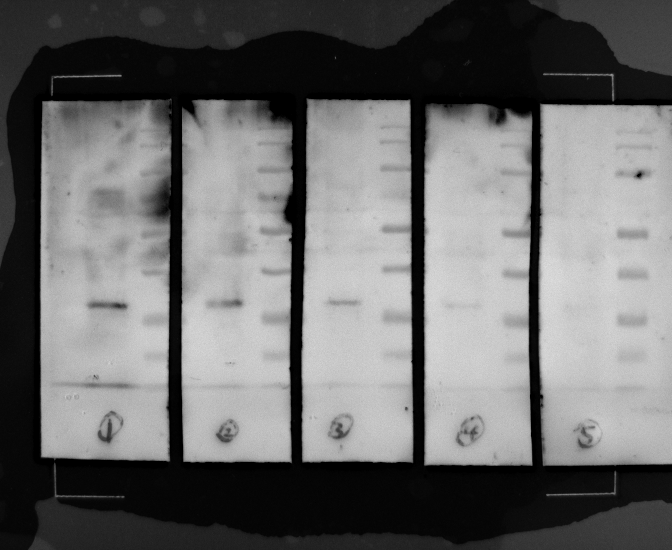

Supplement: Supplementary file 2 [file Presentation_2.ZIP › 2021-07-25-different exposure time-WB original images- full blot/Full wb membrane of syn/2-syn.tif]

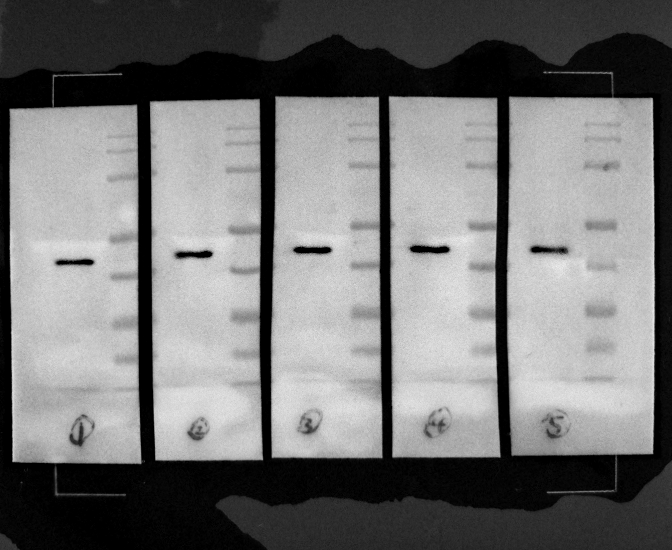

Supplement: Supplementary file 2 [file Presentation_2.ZIP › 2021-07-25-different exposure time-WB original images- full blot/Full wb membrane of syn/actin.tif]

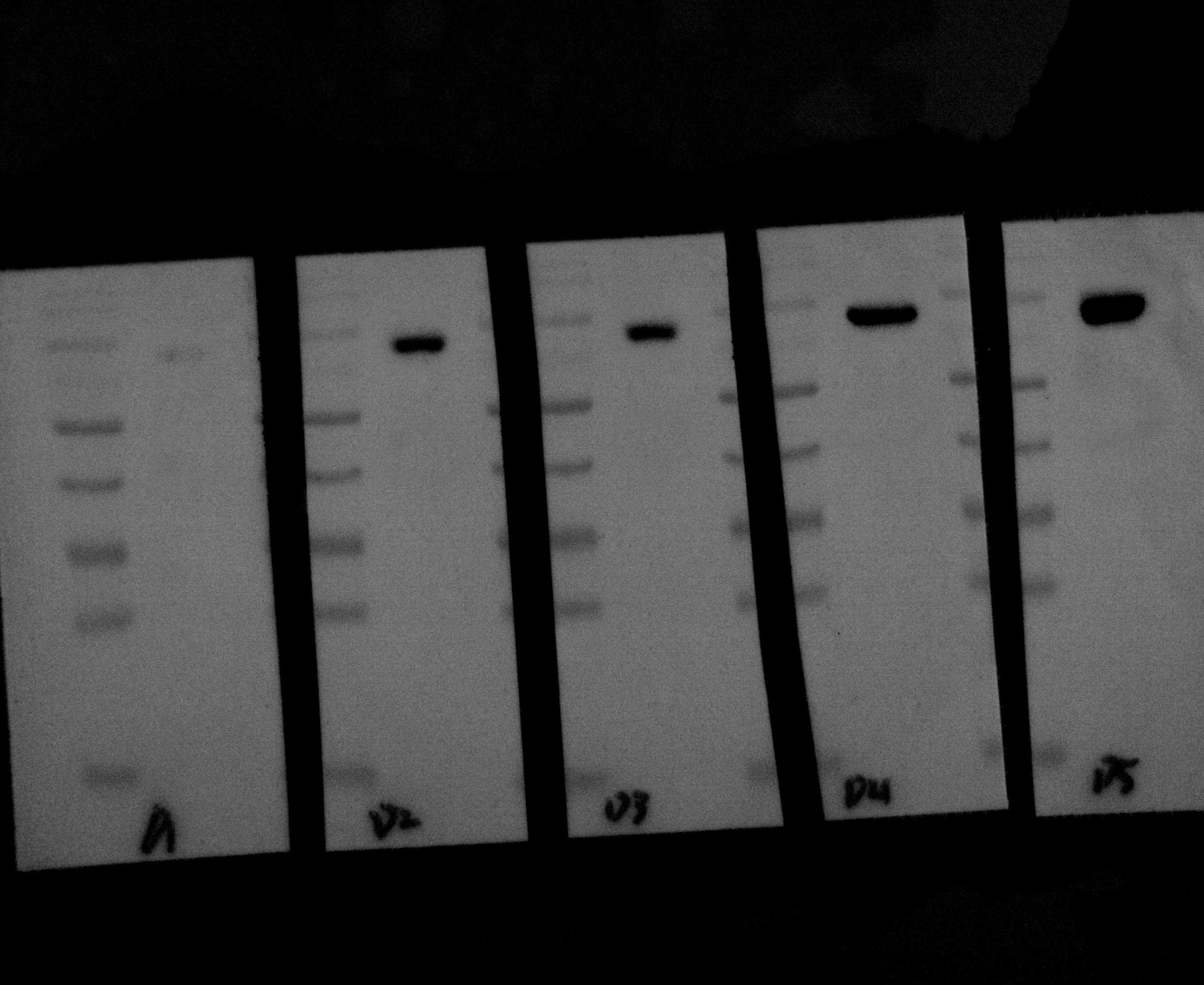

Supplement: Supplementary file 2 [file Presentation_2.ZIP › 2021-07-25-different exposure time-WB original images- full blot/Full wb membrane of psd95/1-PSD95-from the label D1 to D5,the antibody concentration increased gradually.tif]

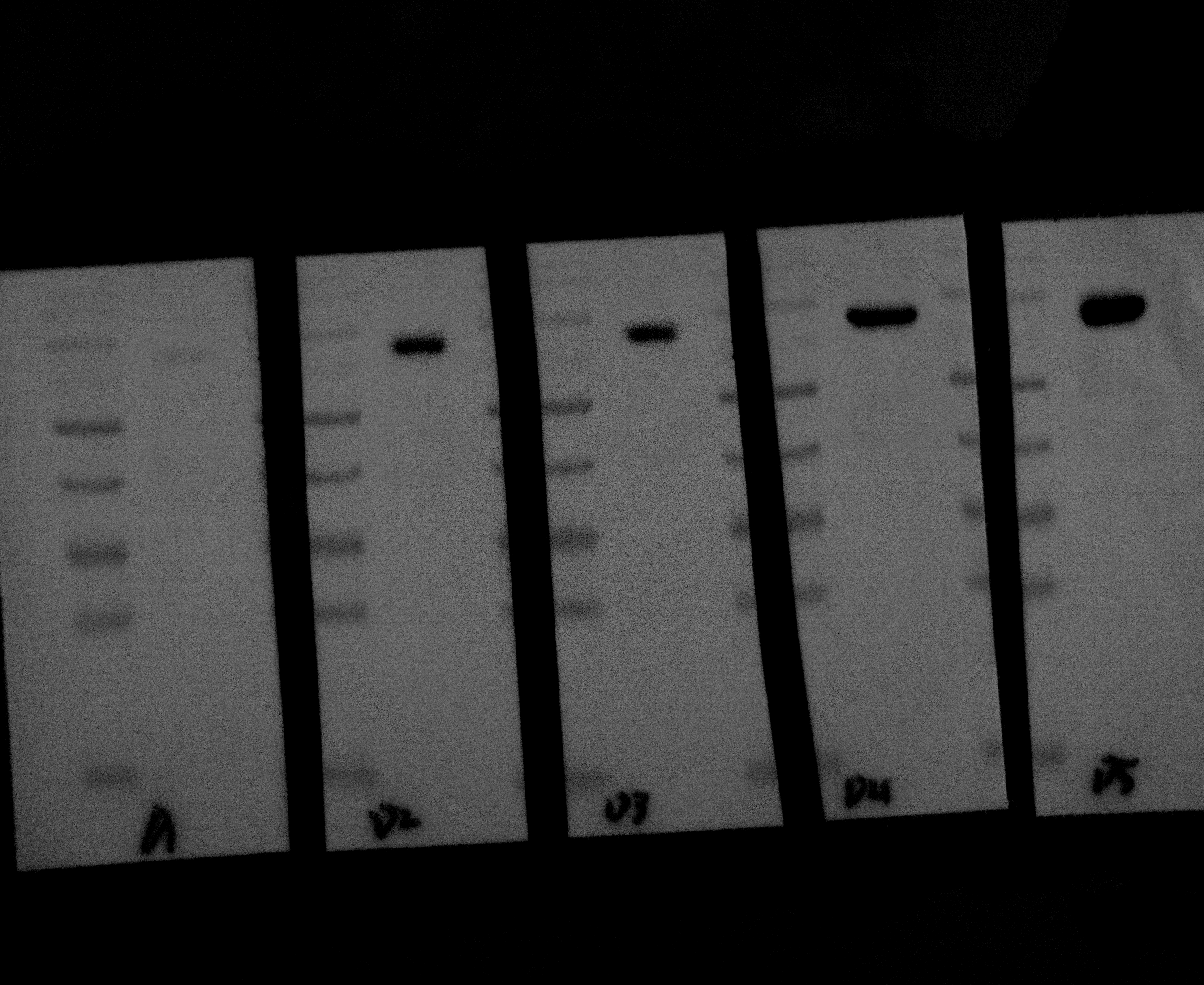

Supplement: Supplementary file 2 [file Presentation_2.ZIP › 2021-07-25-different exposure time-WB original images- full blot/Full wb membrane of psd95/2-PSD95.tif]

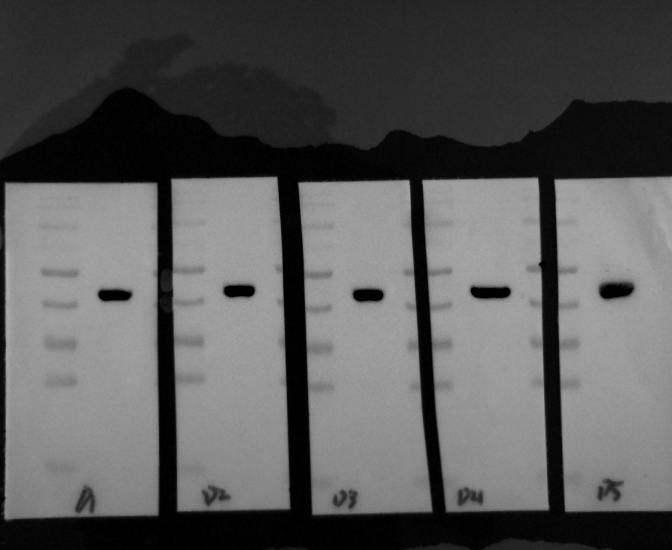

Supplement: Supplementary file 2 [file Presentation_2.ZIP › 2021-07-25-different exposure time-WB original images- full blot/Full wb membrane of psd95/actin.tif]

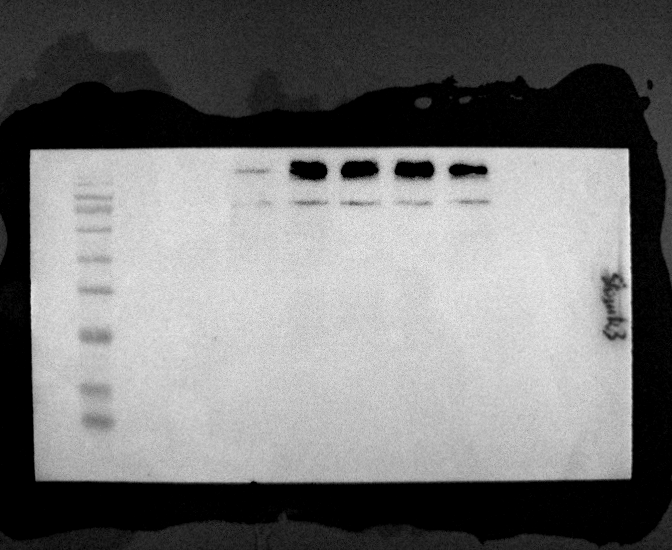

Supplement: Supplementary file 2 [file Presentation_2.ZIP › 2021-07-25-different exposure time-WB original images- full blot/Full wb membrane of shank3/1-Full blot of shank3 in different region of human brain from the third lane to the eigth lane.tif]

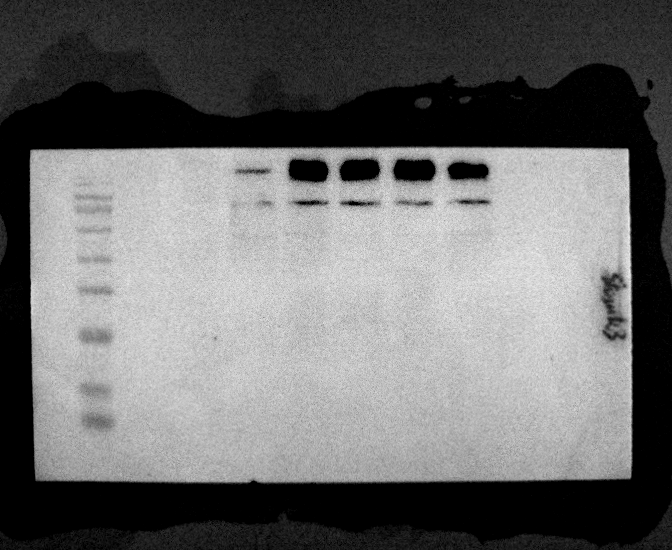

Supplement: Supplementary file 2 [file Presentation_2.ZIP › 2021-07-25-different exposure time-WB original images- full blot/Full wb membrane of shank3/2-Full blot of shank3.tif]

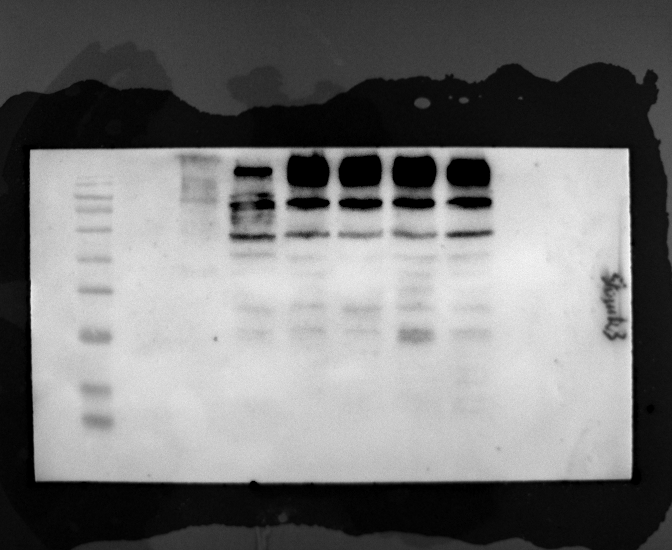

Supplement: Supplementary file 2 [file Presentation_2.ZIP › 2021-07-25-different exposure time-WB original images- full blot/Full wb membrane of shank3/3-Full blot of shank3.tif]

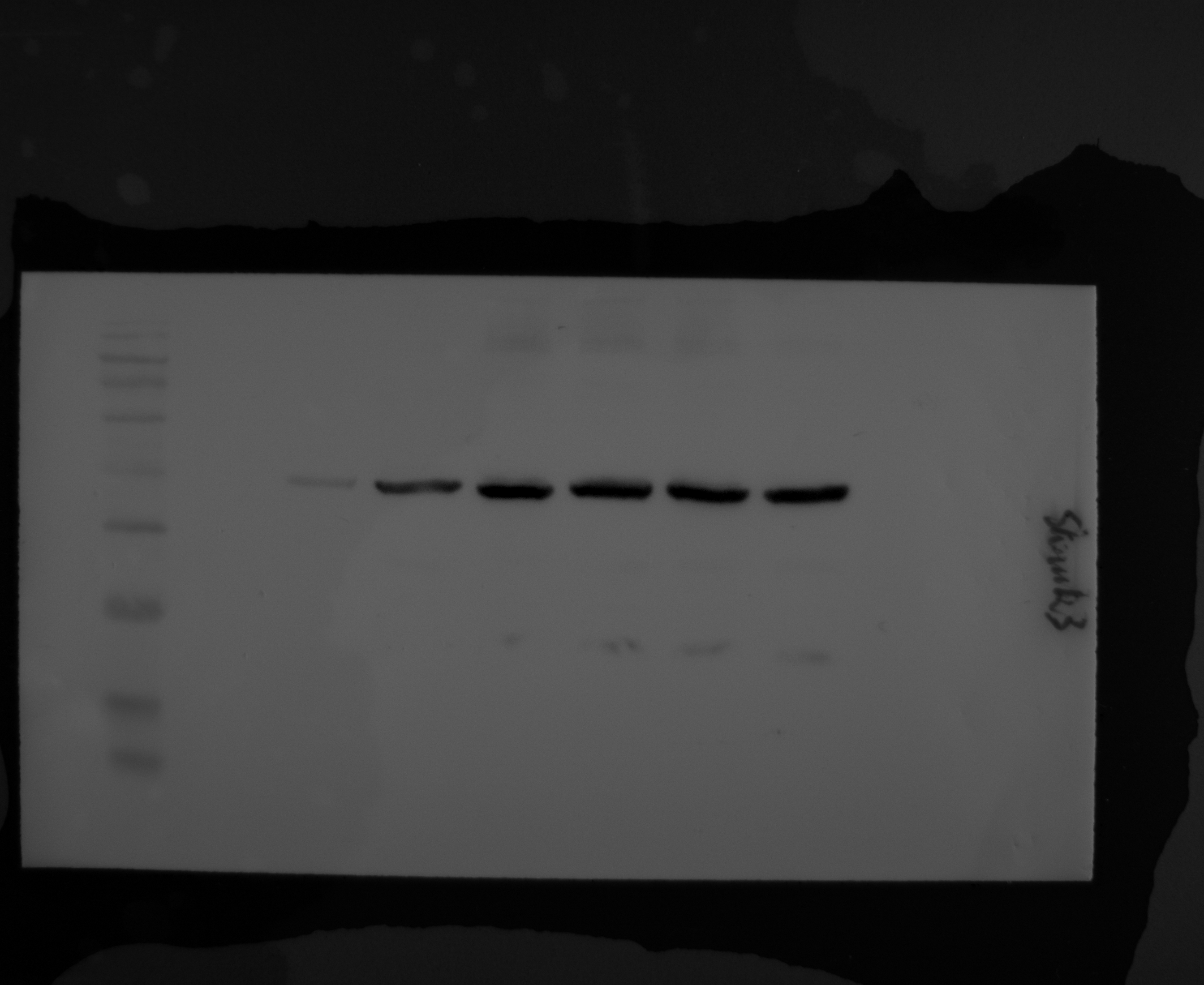

Supplement: Supplementary file 2 [file Presentation_2.ZIP › 2021-07-25-different exposure time-WB original images- full blot/Full wb membrane of shank3/actin.tif]

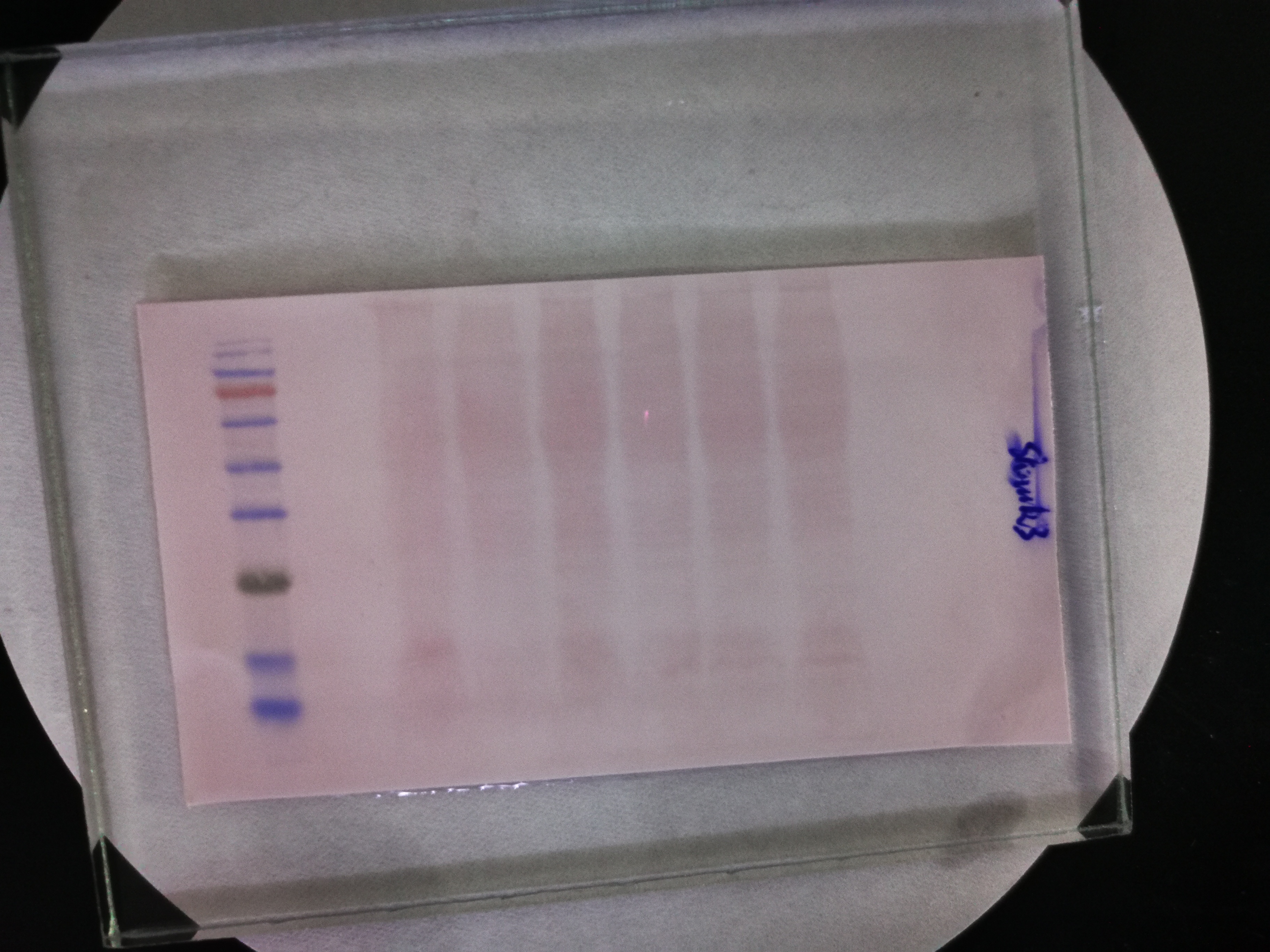

Supplement: Supplementary file 2 [file Presentation_2.ZIP › 2021-07-25-different exposure time-WB original images- full blot/Full wb membrane of shank3/ponceau-1.jpg]

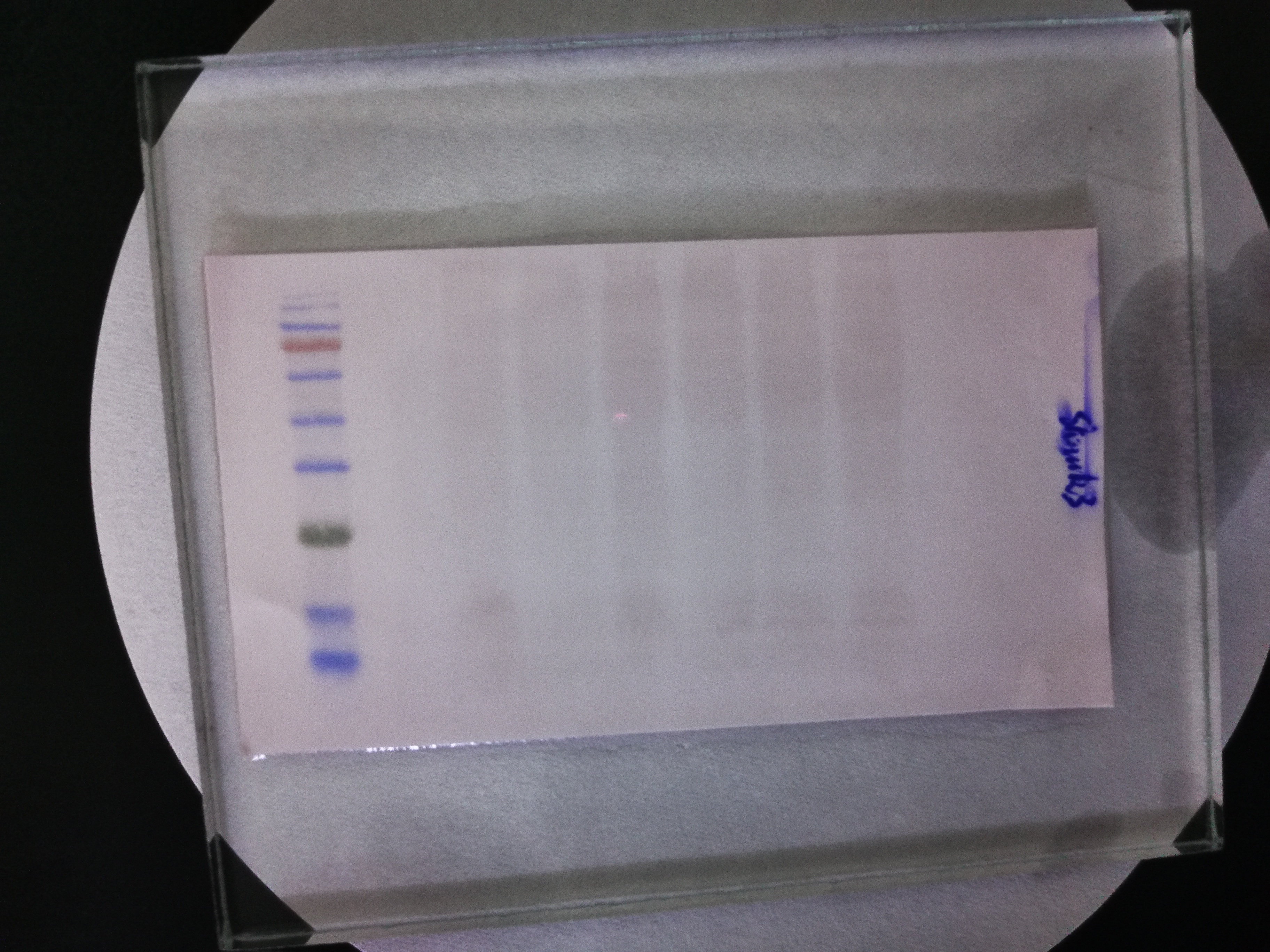

Supplement: Supplementary file 2 [file Presentation_2.ZIP › 2021-07-25-different exposure time-WB original images- full blot/Full wb membrane of shank3/ponceau-2.jpg]

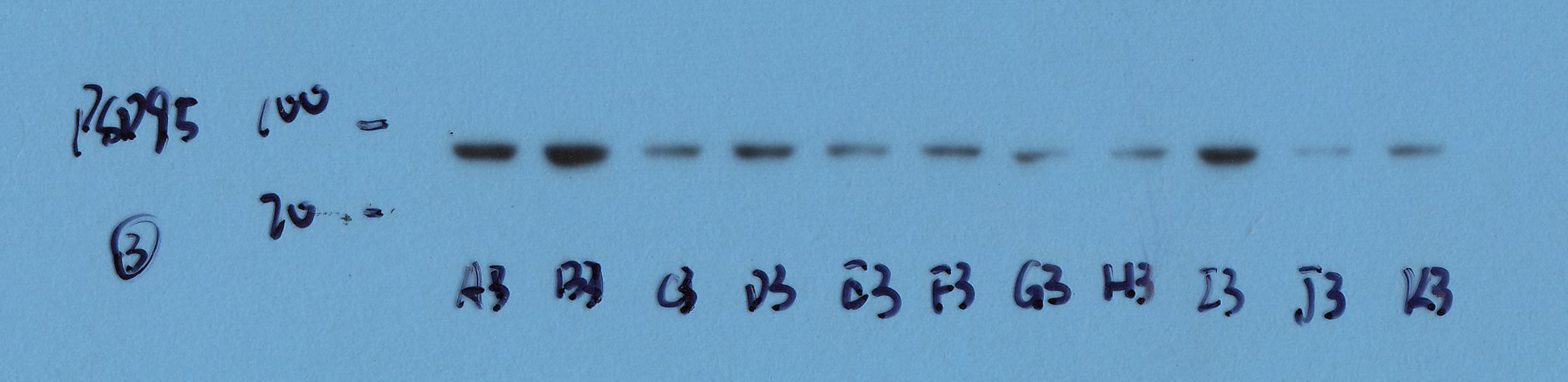

Supplement: Supplementary file 3 [file Presentation_3.ZIP › Figure 10D-Imaging with film exposure/Figure 10D-PSD95-case#14.tif]

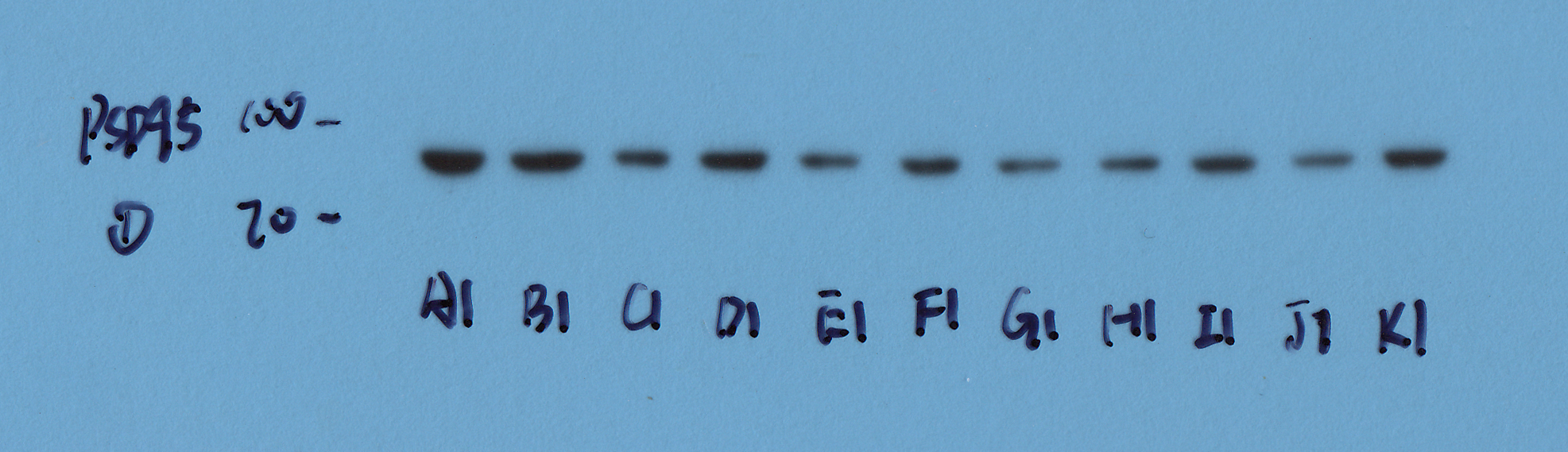

Supplement: Supplementary file 3 [file Presentation_3.ZIP › Figure 10D-Imaging with film exposure/Figure 10D-PSD95-case#6.tif]

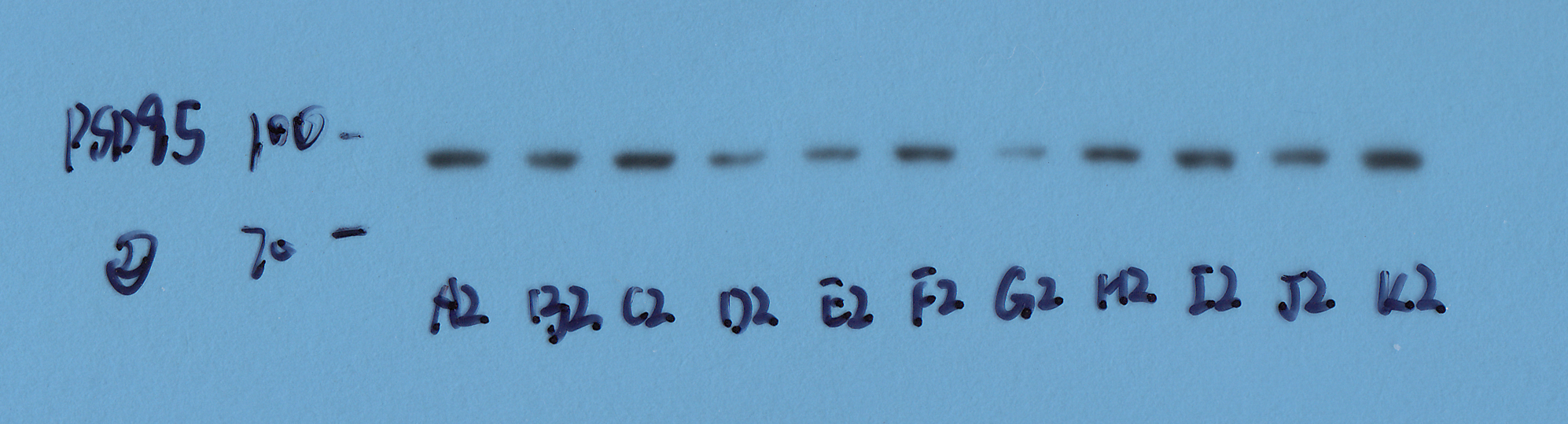

Supplement: Supplementary file 3 [file Presentation_3.ZIP › Figure 10D-Imaging with film exposure/Figure 10D-PSD95-case#9.tif]

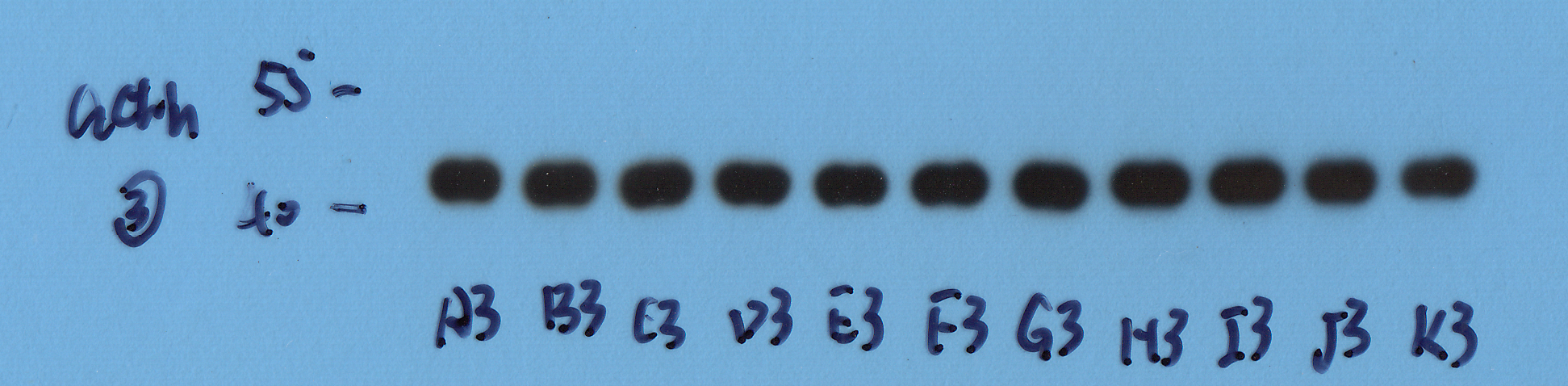

Supplement: Supplementary file 3 [file Presentation_3.ZIP › Figure 10D-Imaging with film exposure/Figure 10D-actin-case#14.tif]
