# Supplementary figures and images for: Expression of the Excitatory Postsynaptic Scaffolding Protein, Shank3, in Human Brain: Effect of Age and Alzheimer’s Disease (part 1 of 3)
Source: Front Aging Neurosci. 2021 Aug 24;13:717263. doi: 10.3389/fnagi.2021.717263 (PMC8421777; doi:10.3389/fnagi.2021.717263)

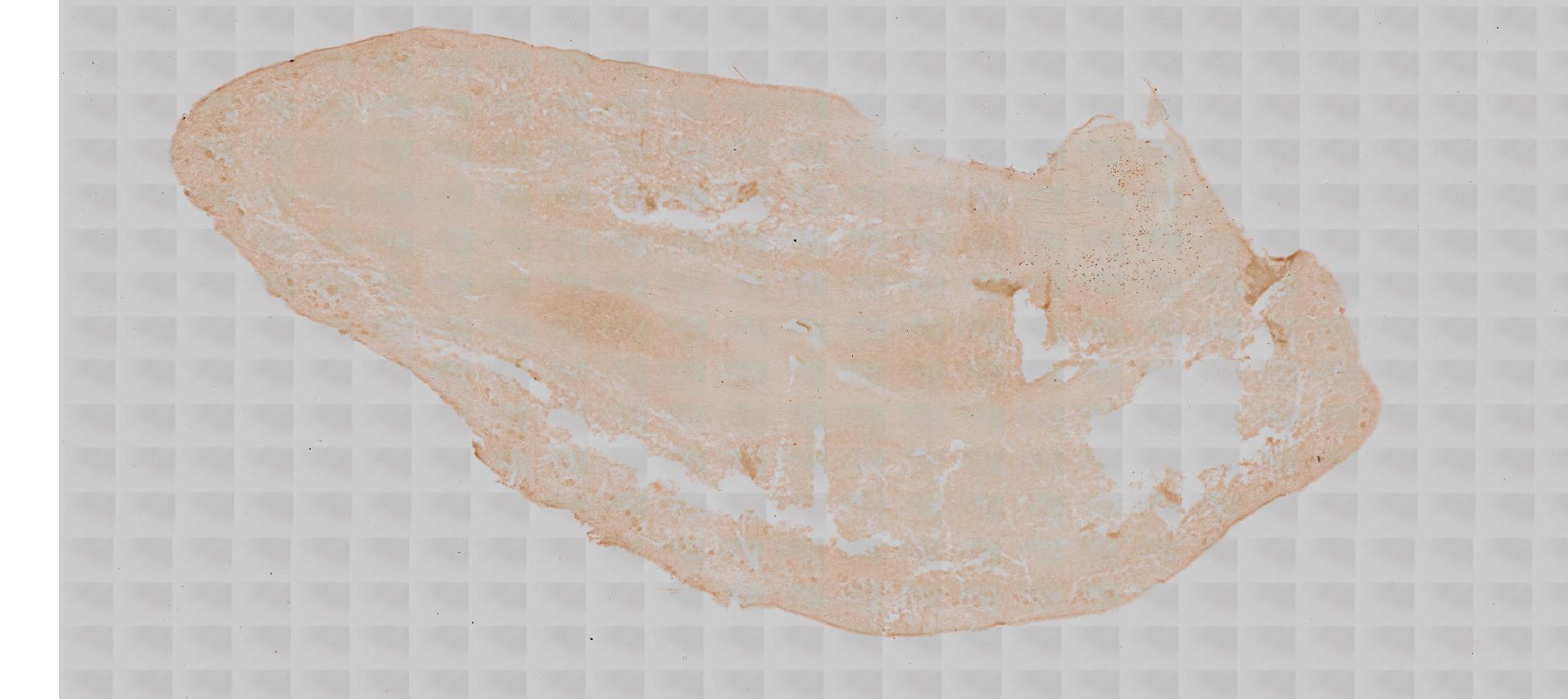

Supplement: Supplementary file 1 [file Presentation_1.ZIP › shank3-immunohistochemistry/Case-8/1-olfactory bulb.jpg]

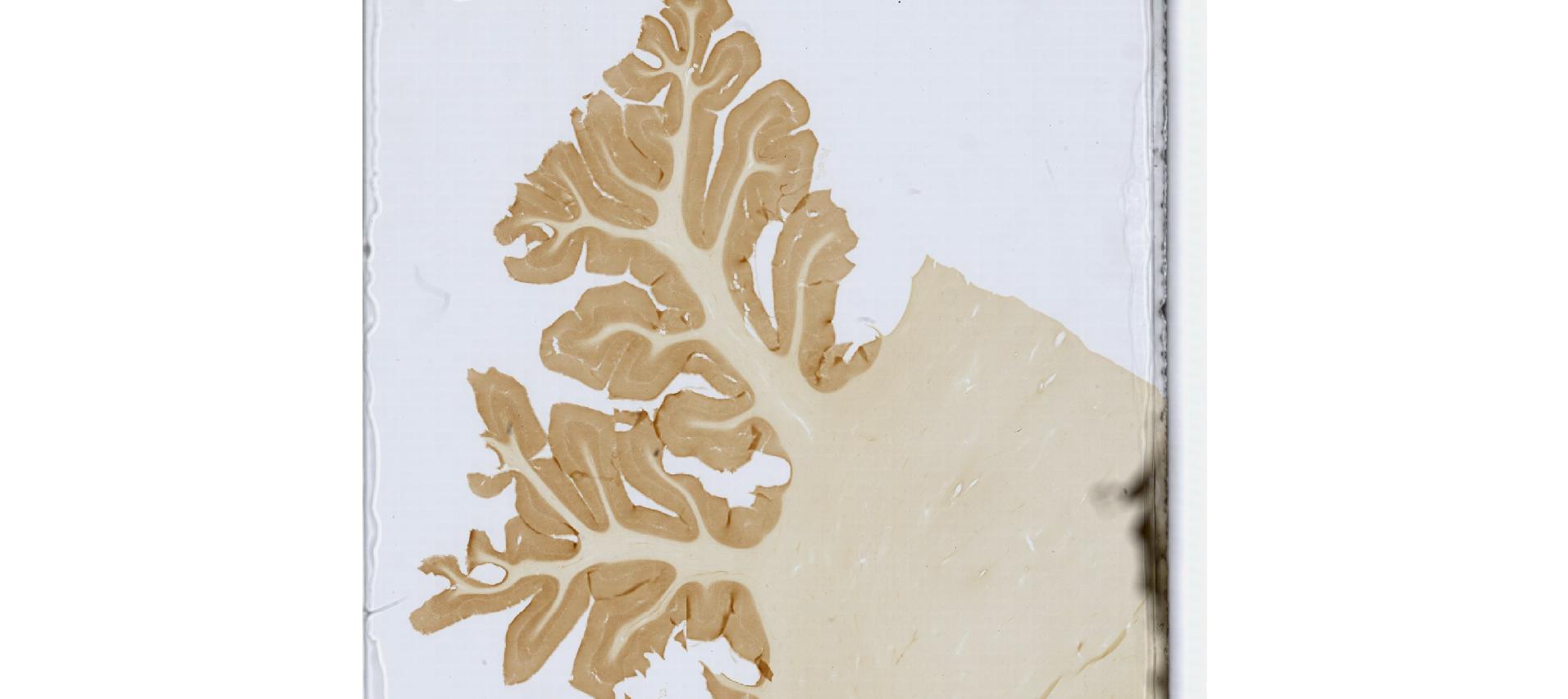

Supplement: Supplementary file 1 [file Presentation_1.ZIP › shank3-immunohistochemistry/Case-8/10-cerebellum.jpg]

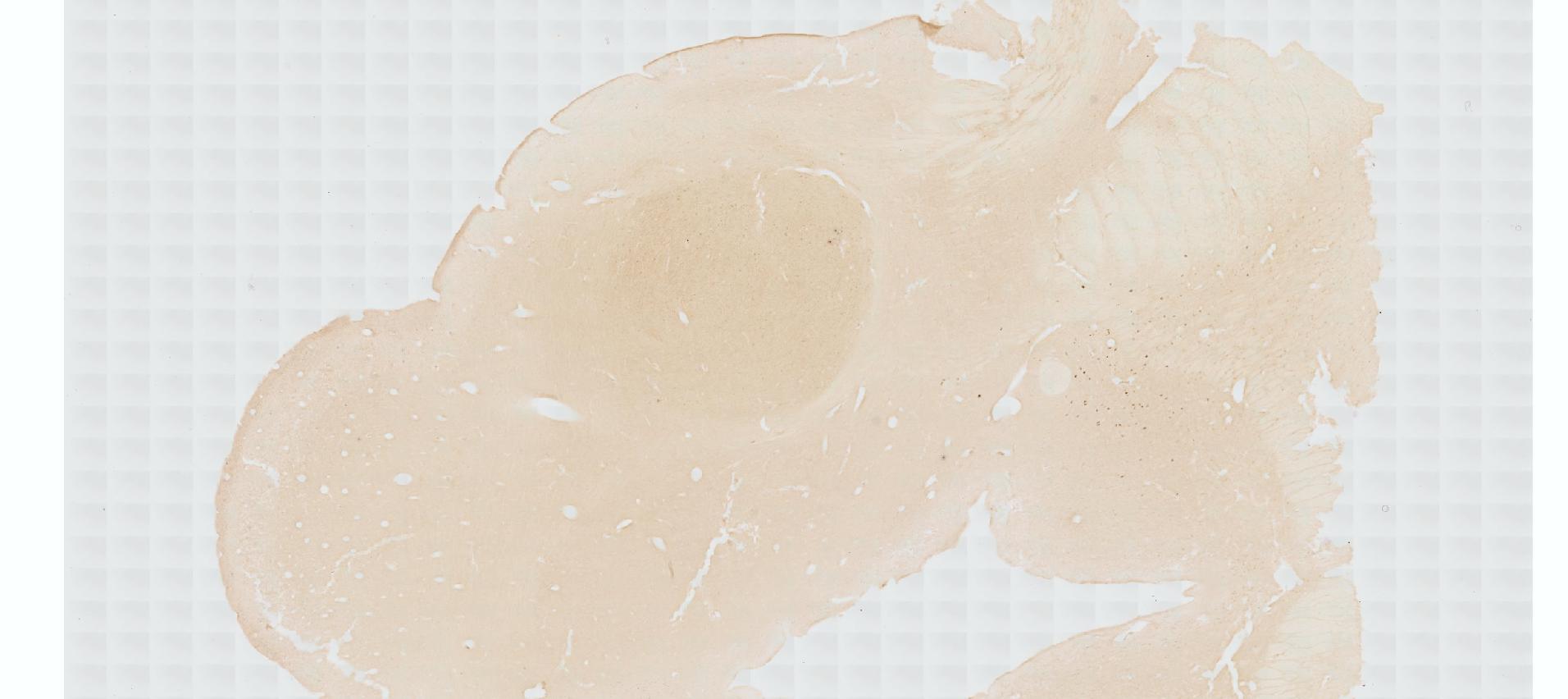

Supplement: Supplementary file 1 [file Presentation_1.ZIP › shank3-immunohistochemistry/Case-8/11-midbrain.jpg]

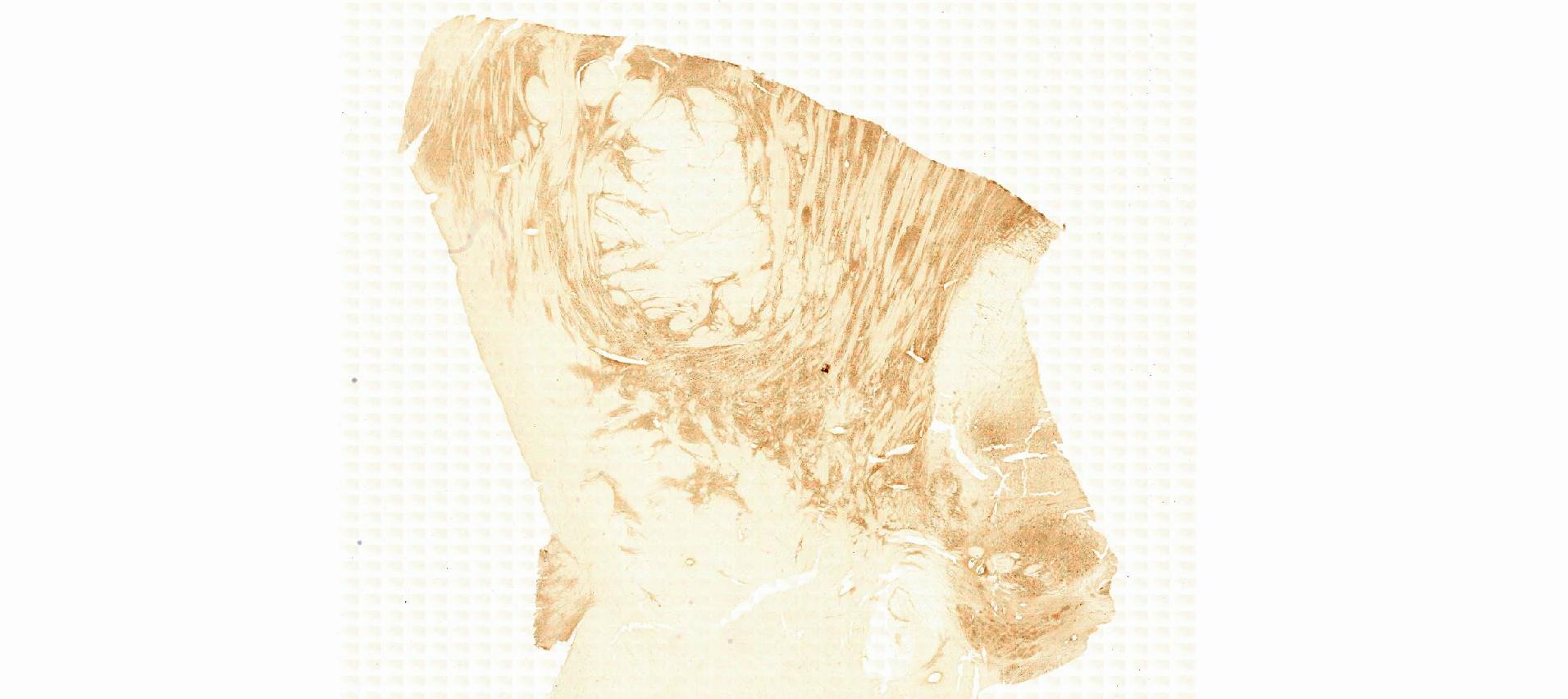

Supplement: Supplementary file 1 [file Presentation_1.ZIP › shank3-immunohistochemistry/Case-8/12-pons.jpg]

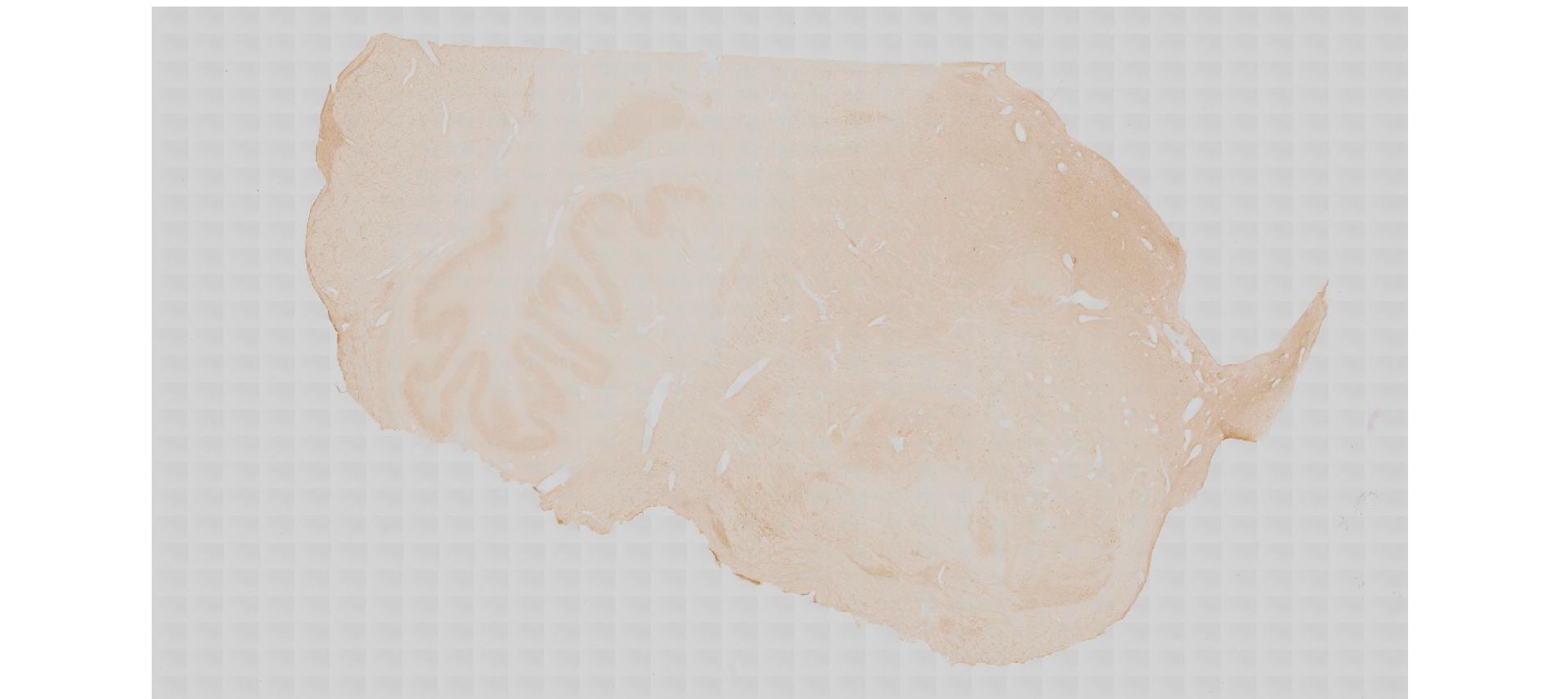

Supplement: Supplementary file 1 [file Presentation_1.ZIP › shank3-immunohistochemistry/Case-8/13-medulla oblongata.jpg]

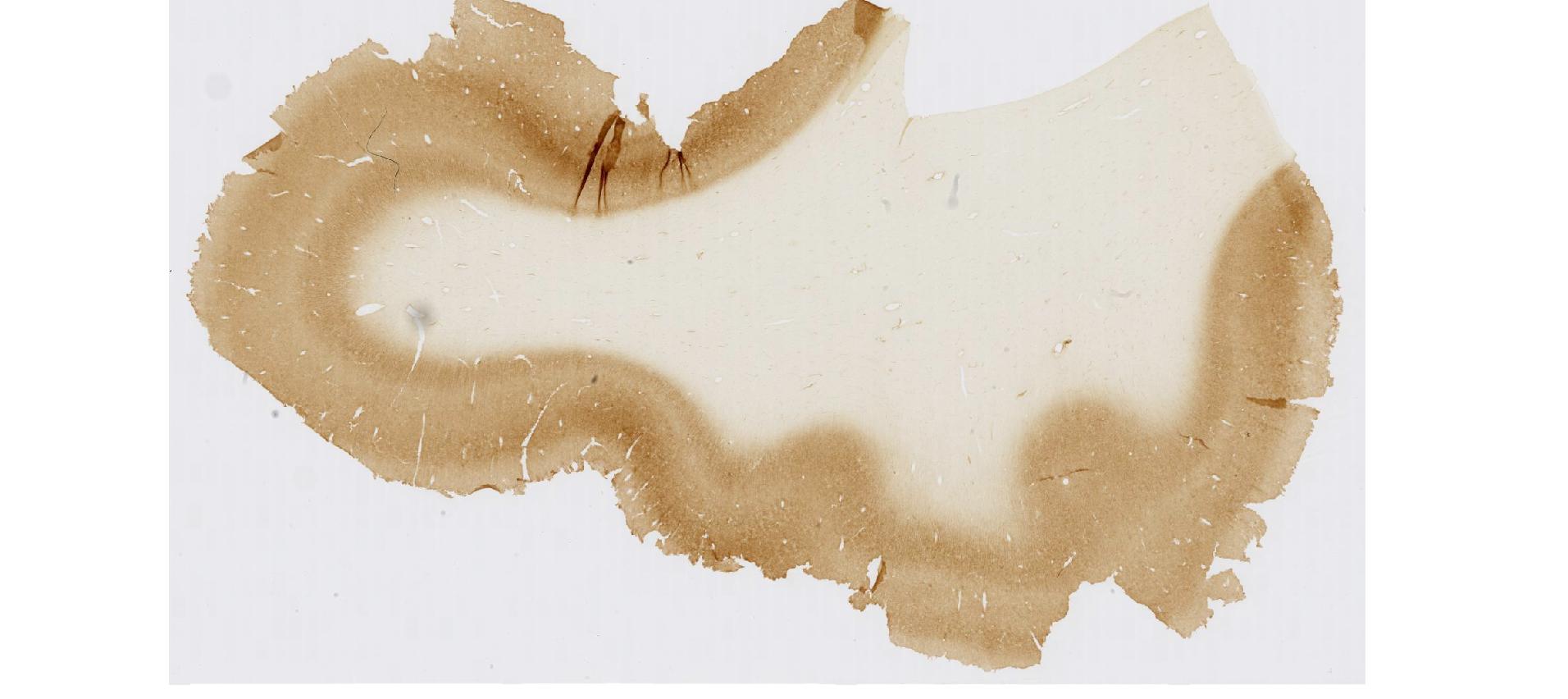

Supplement: Supplementary file 1 [file Presentation_1.ZIP › shank3-immunohistochemistry/Case-8/2-prefrontal cortex.jpg]

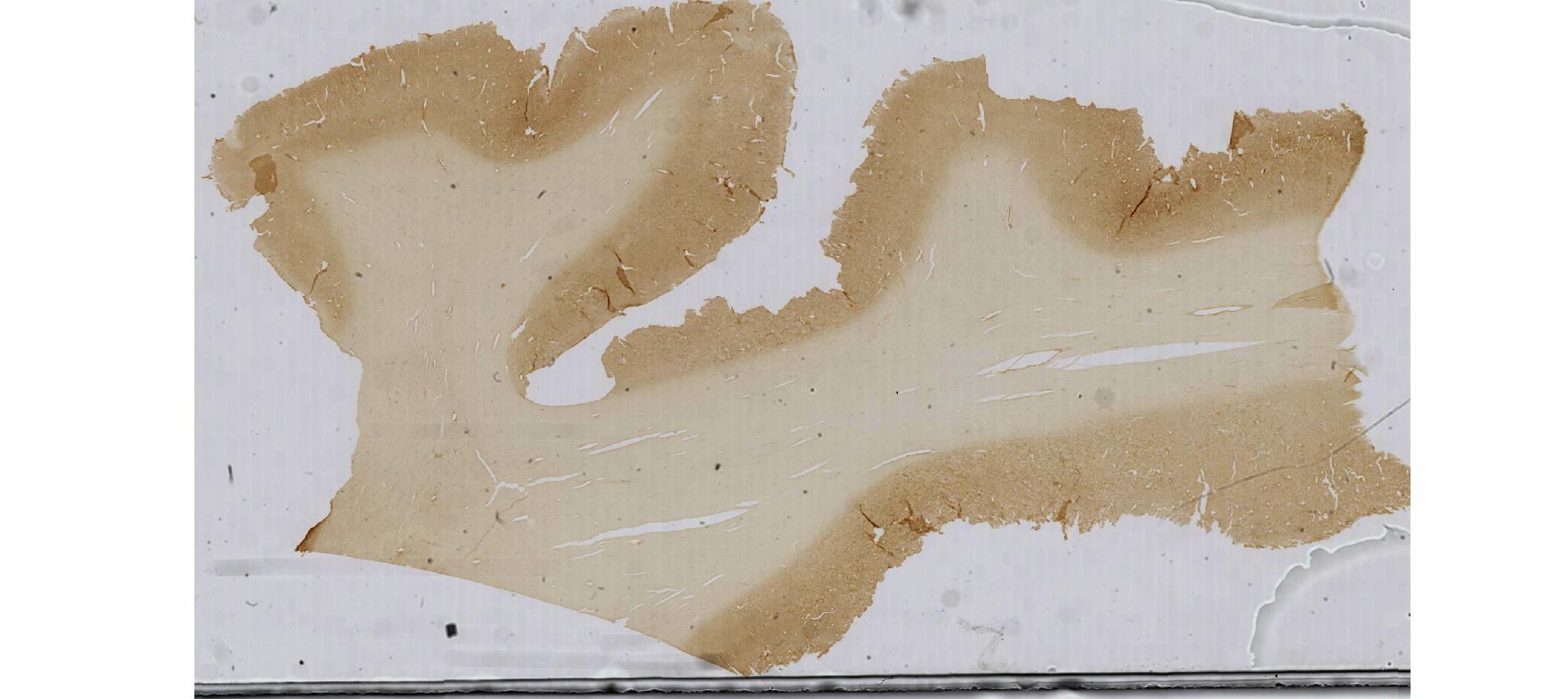

Supplement: Supplementary file 1 [file Presentation_1.ZIP › shank3-immunohistochemistry/Case-8/3-anterior cingulate neocortex.jpg]

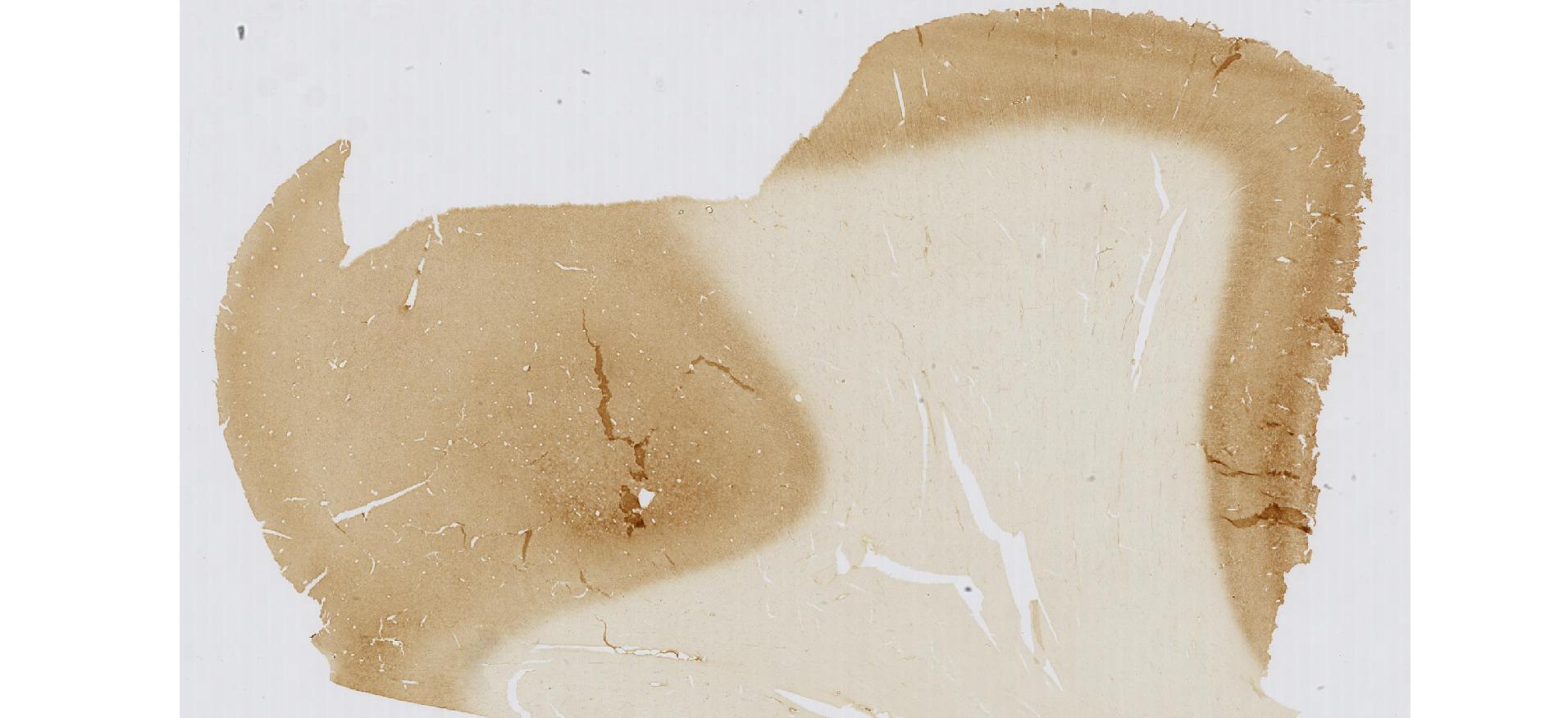

Supplement: Supplementary file 1 [file Presentation_1.ZIP › shank3-immunohistochemistry/Case-8/4- precentral cortex.jpg]

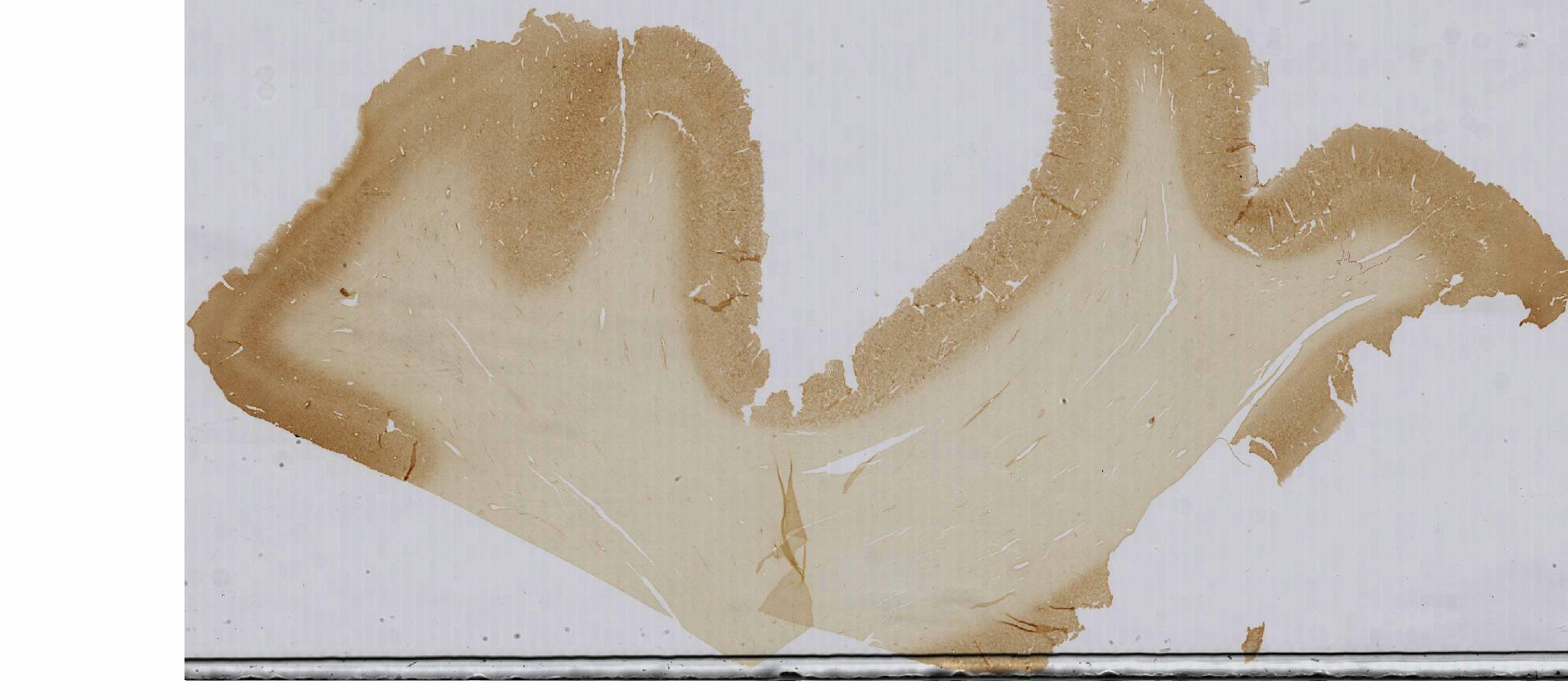

Supplement: Supplementary file 1 [file Presentation_1.ZIP › shank3-immunohistochemistry/Case-8/5-postcentral cortex.jpg]

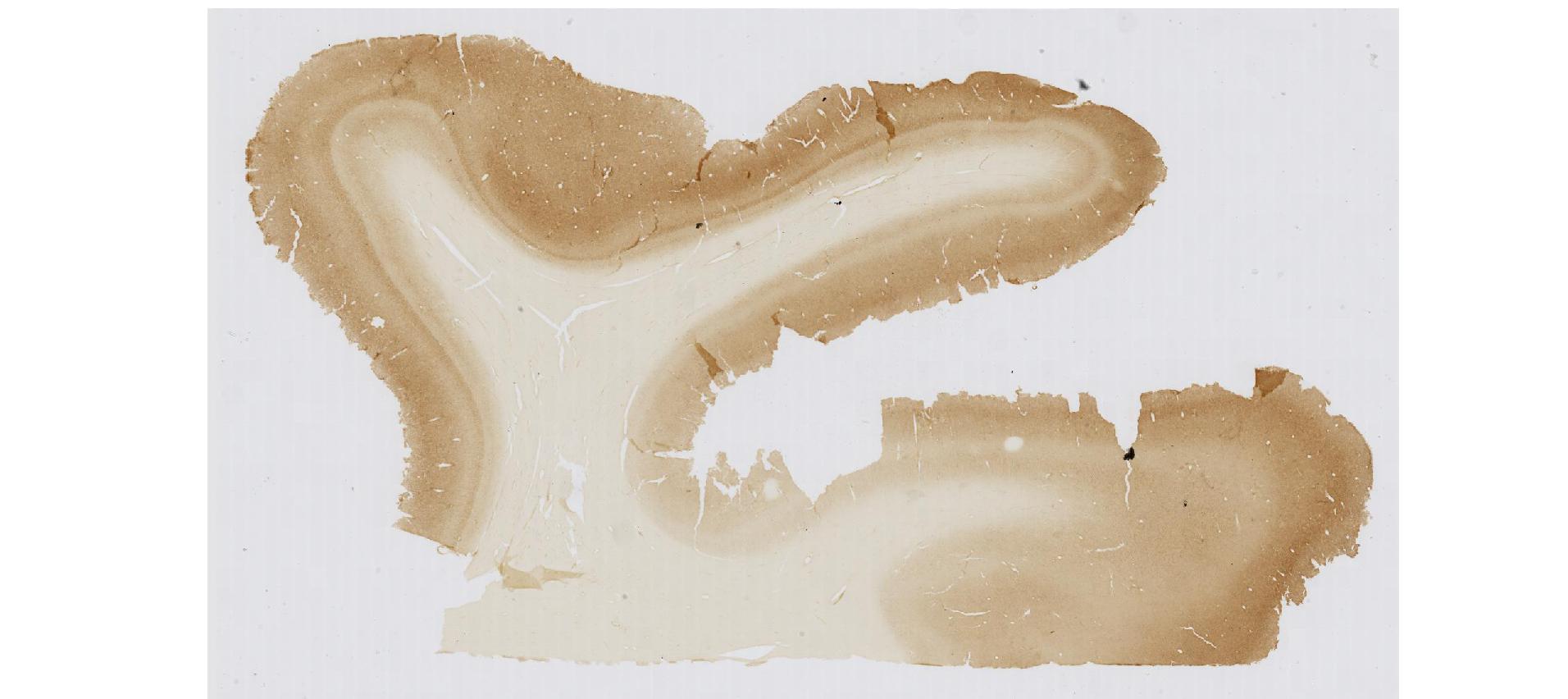

Supplement: Supplementary file 1 [file Presentation_1.ZIP › shank3-immunohistochemistry/Case-8/6- visual cortex.jpg]

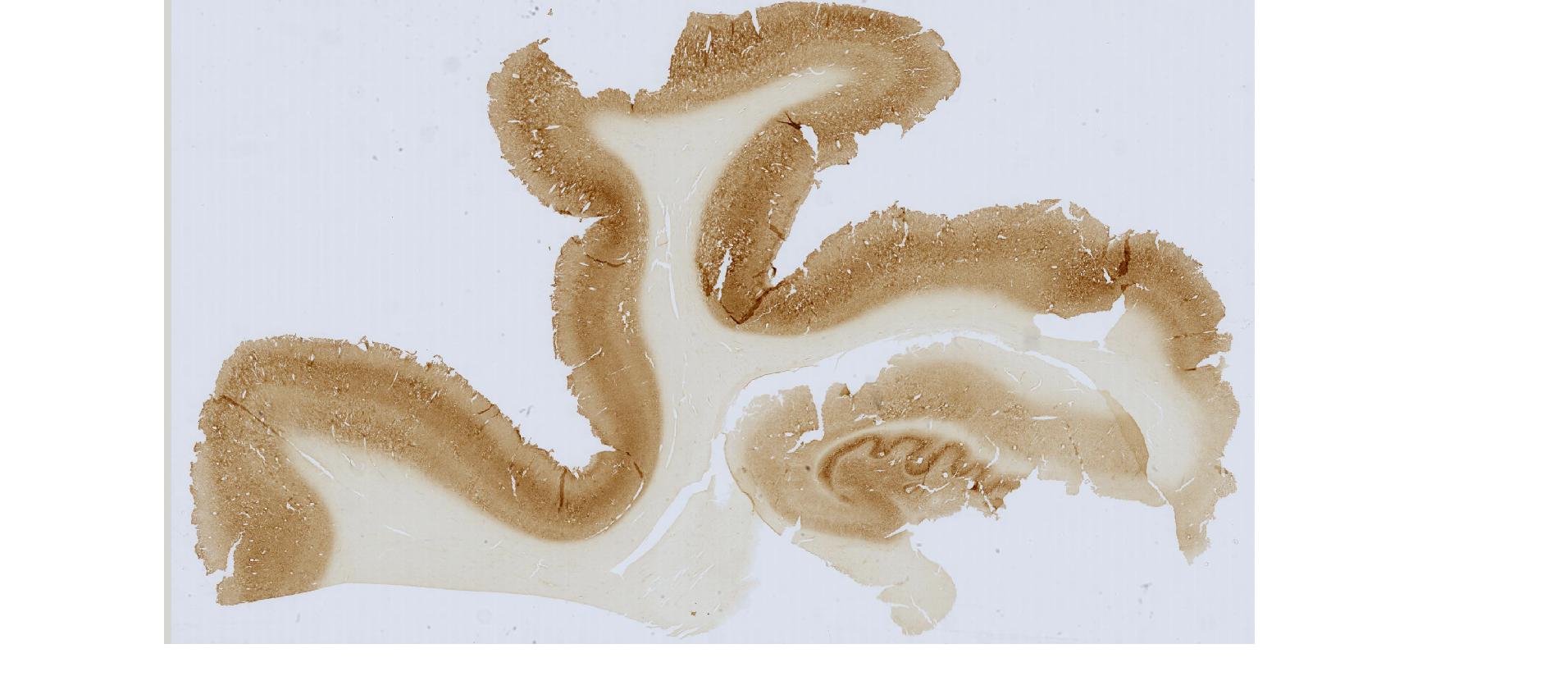

Supplement: Supplementary file 1 [file Presentation_1.ZIP › shank3-immunohistochemistry/Case-8/7-hippocampal formation.jpg]

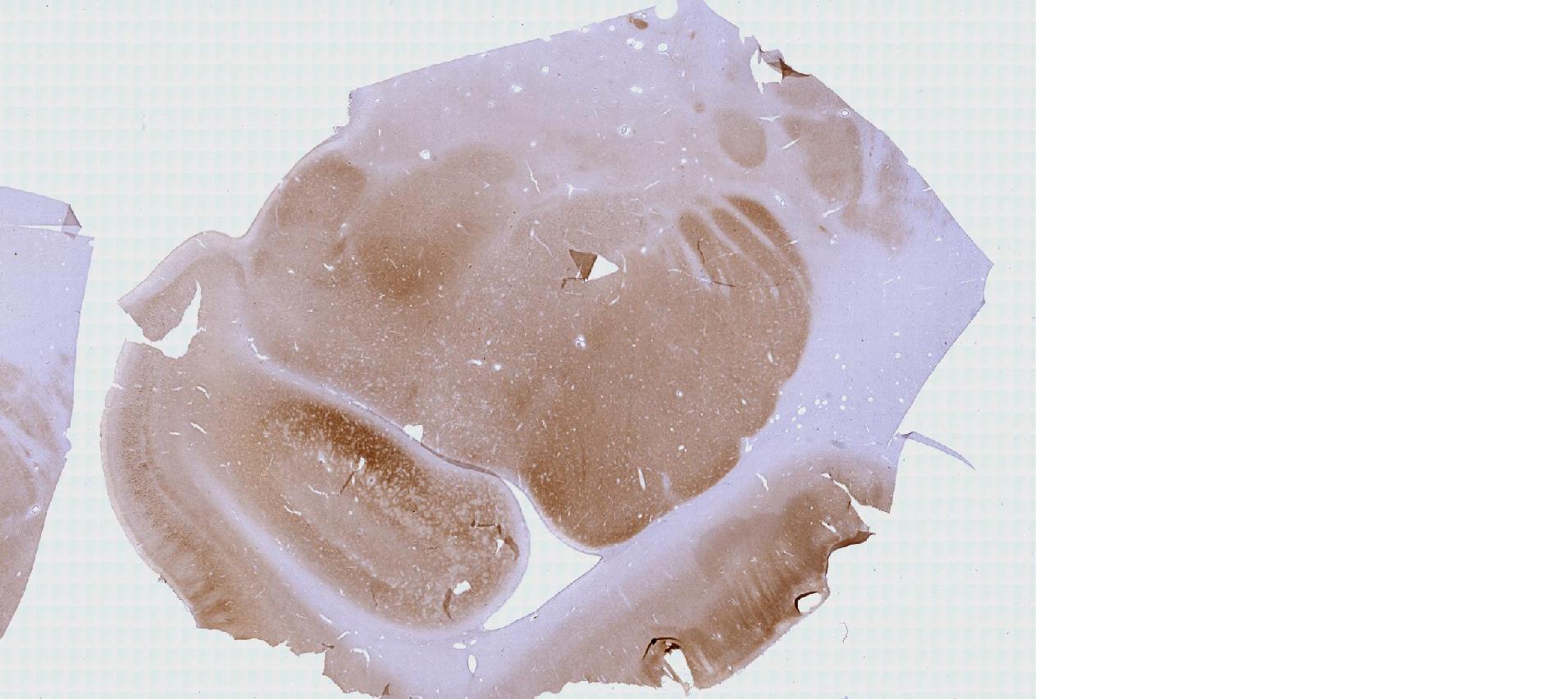

Supplement: Supplementary file 1 [file Presentation_1.ZIP › shank3-immunohistochemistry/Case-8/8-amygdalar complex.jpg]

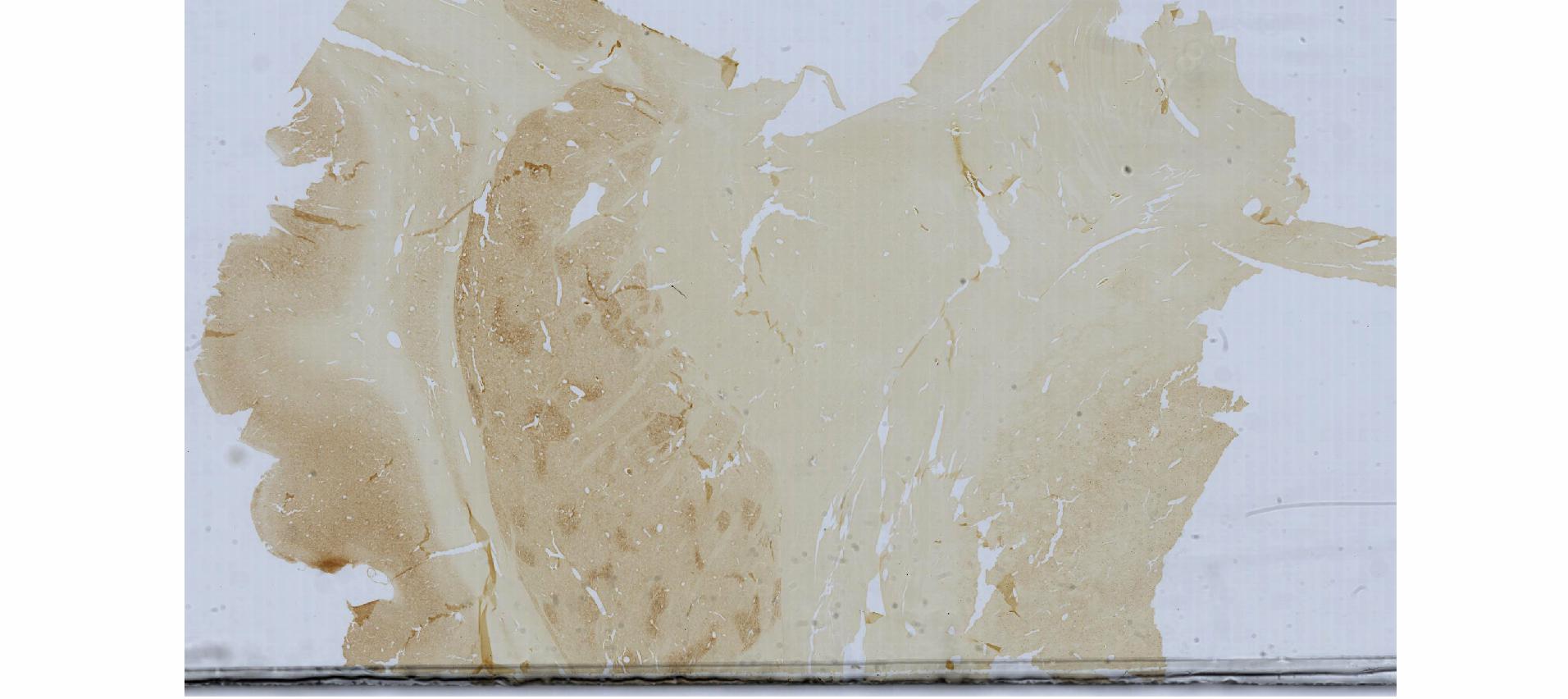

Supplement: Supplementary file 1 [file Presentation_1.ZIP › shank3-immunohistochemistry/Case-8/9-basal ganglia and diencephalon.jpg]

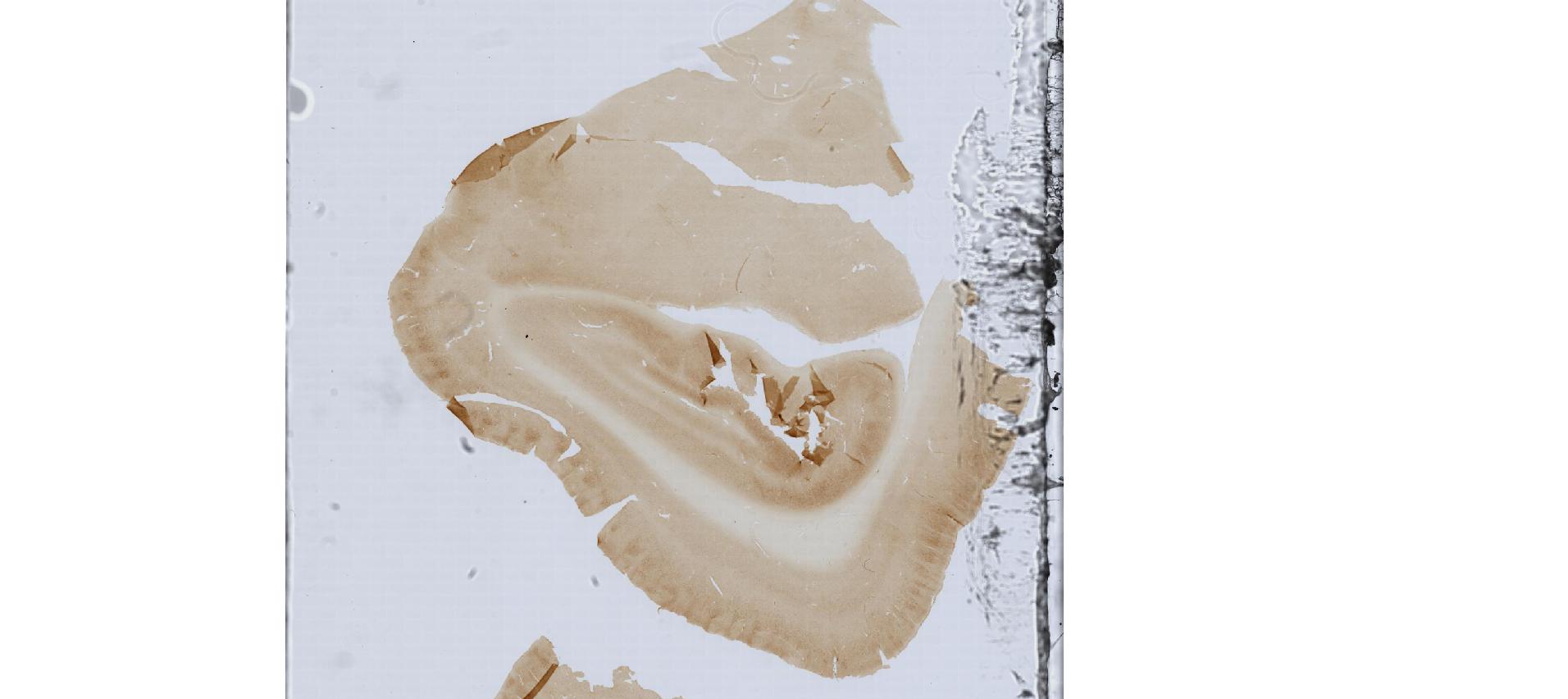

Supplement: Supplementary file 1 [file Presentation_1.ZIP › shank3-immunohistochemistry/case-1/amygdalar complex.jpg]

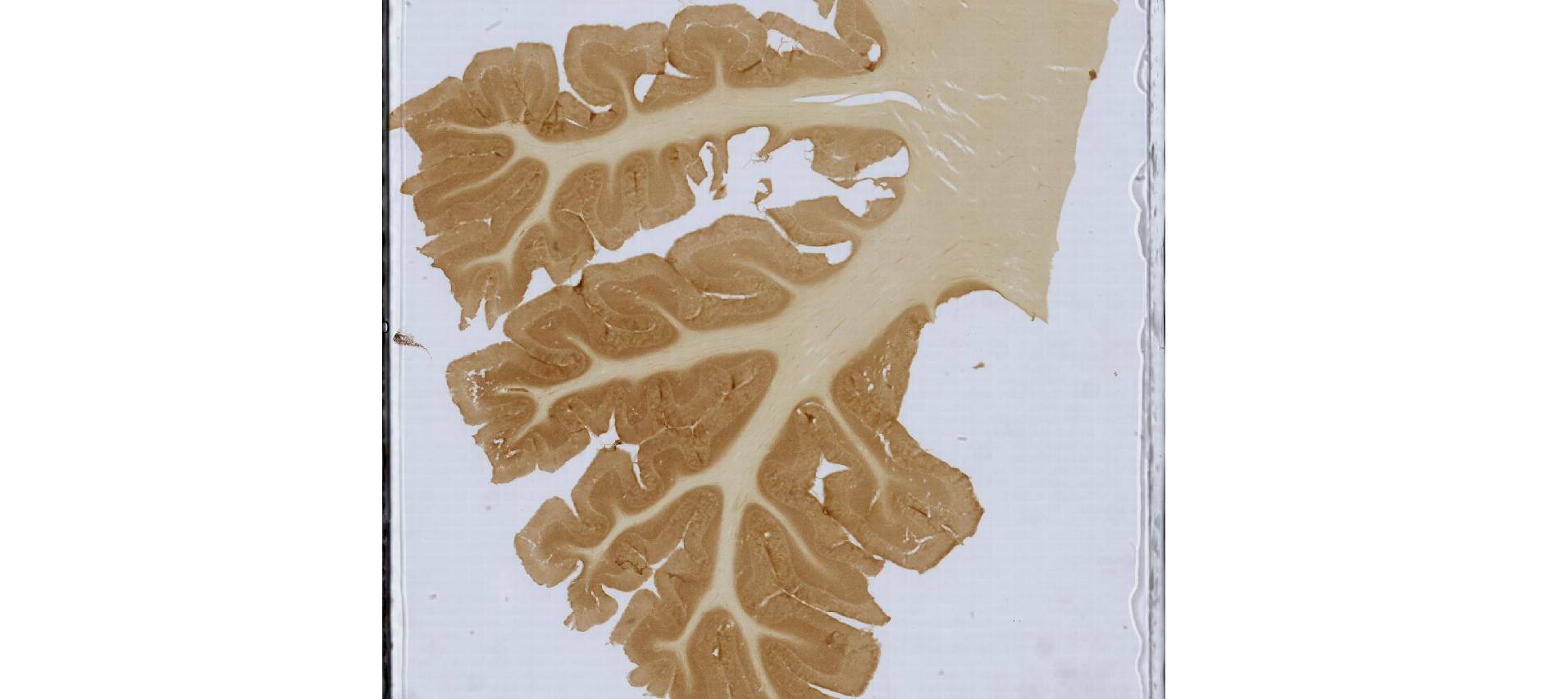

Supplement: Supplementary file 1 [file Presentation_1.ZIP › shank3-immunohistochemistry/case-1/cerebellum.jpg]

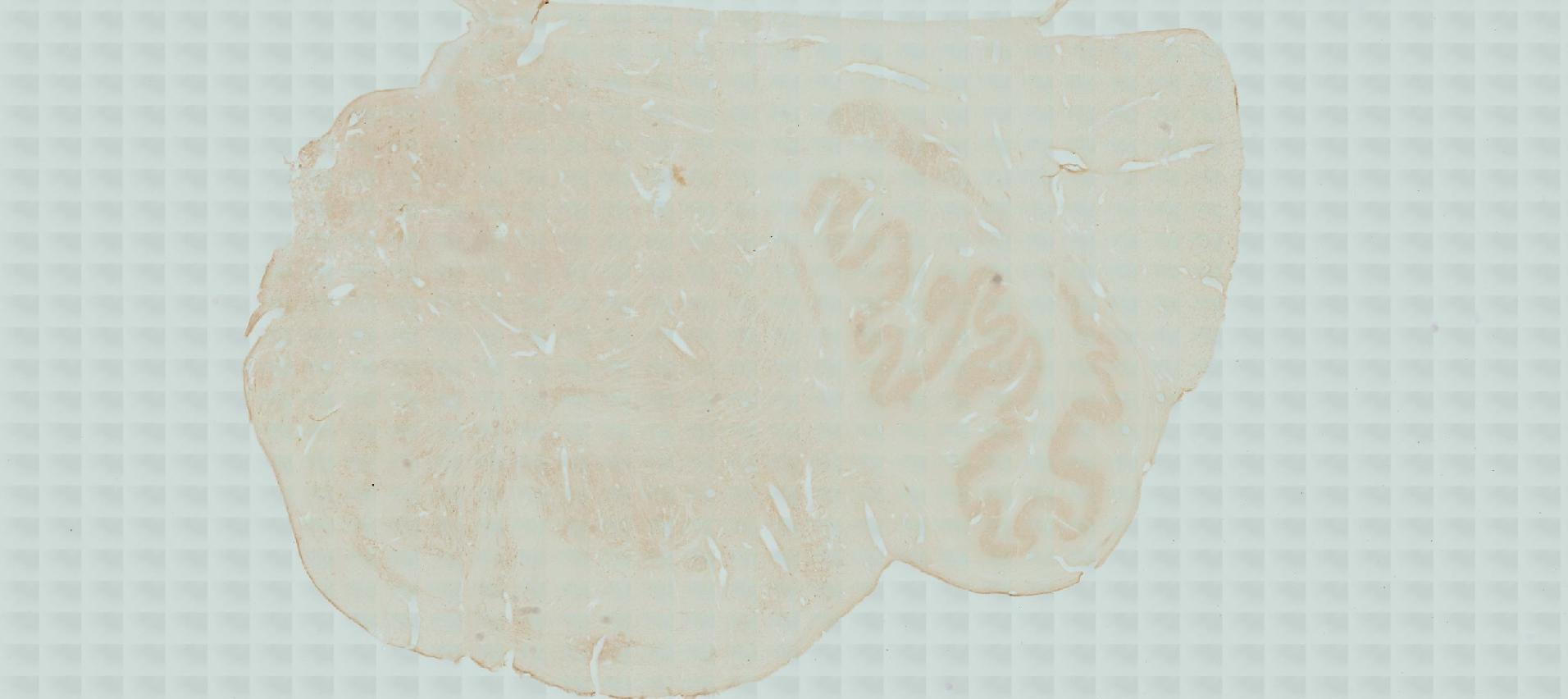

Supplement: Supplementary file 1 [file Presentation_1.ZIP › shank3-immunohistochemistry/case-1/medulla oblongata.jpg]

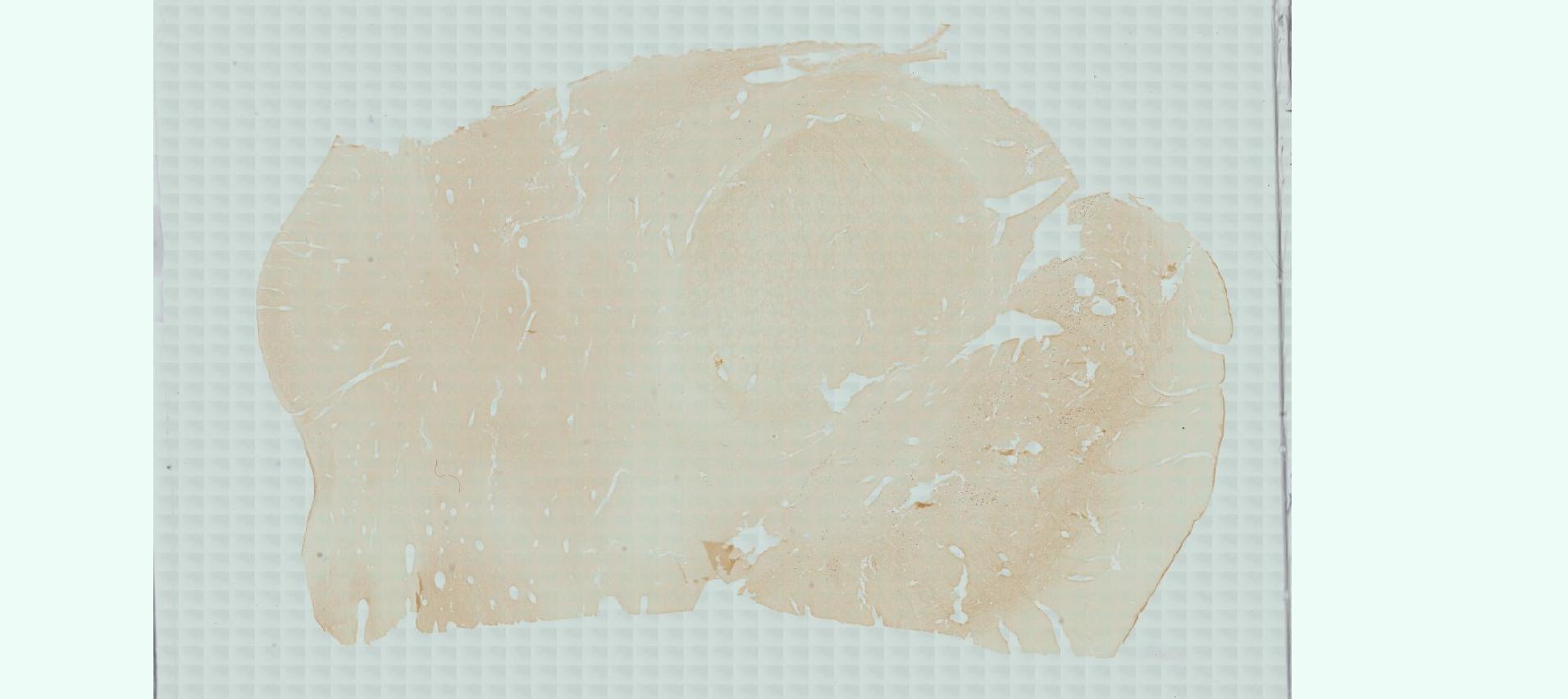

Supplement: Supplementary file 1 [file Presentation_1.ZIP › shank3-immunohistochemistry/case-1/midbrain.jpg]

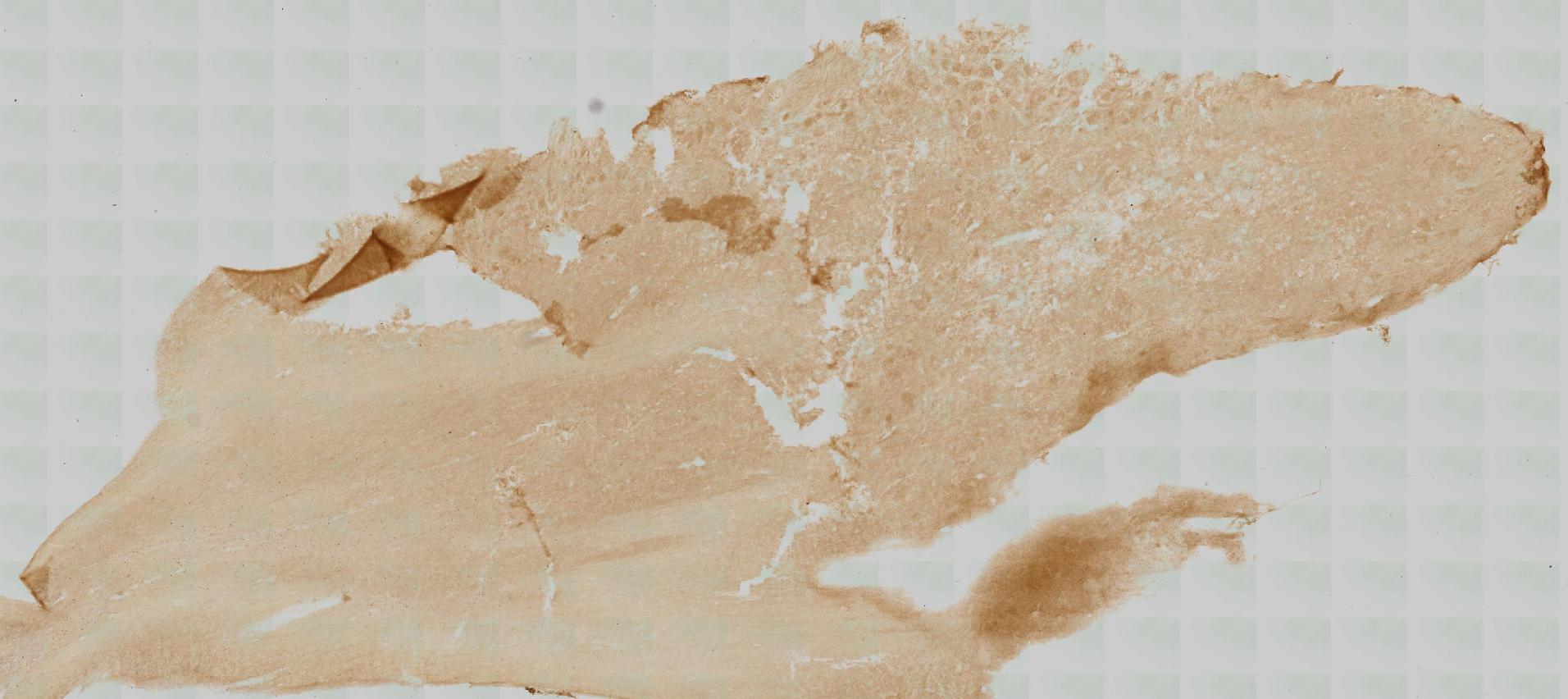

Supplement: Supplementary file 1 [file Presentation_1.ZIP › shank3-immunohistochemistry/case-1/olfactory bulb.jpg]

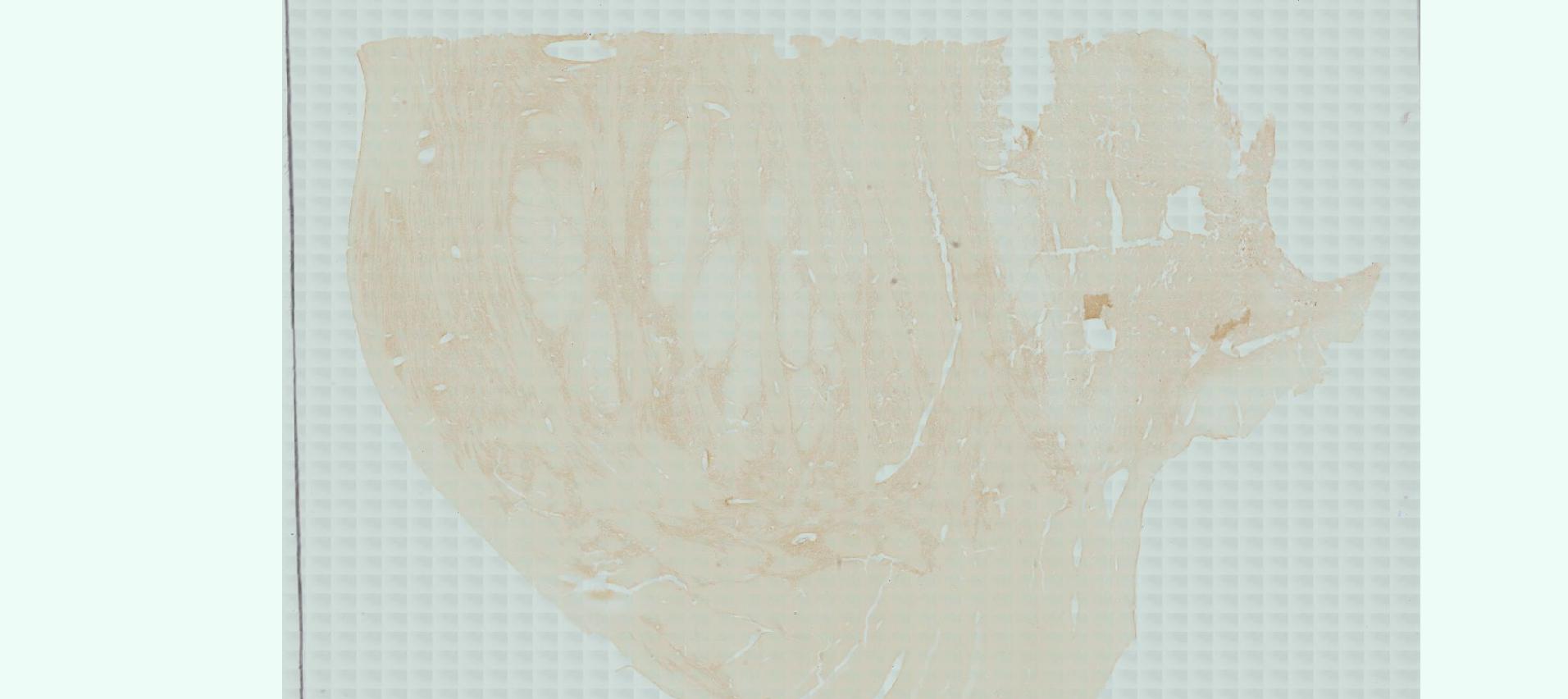

Supplement: Supplementary file 1 [file Presentation_1.ZIP › shank3-immunohistochemistry/case-1/pons.jpg]

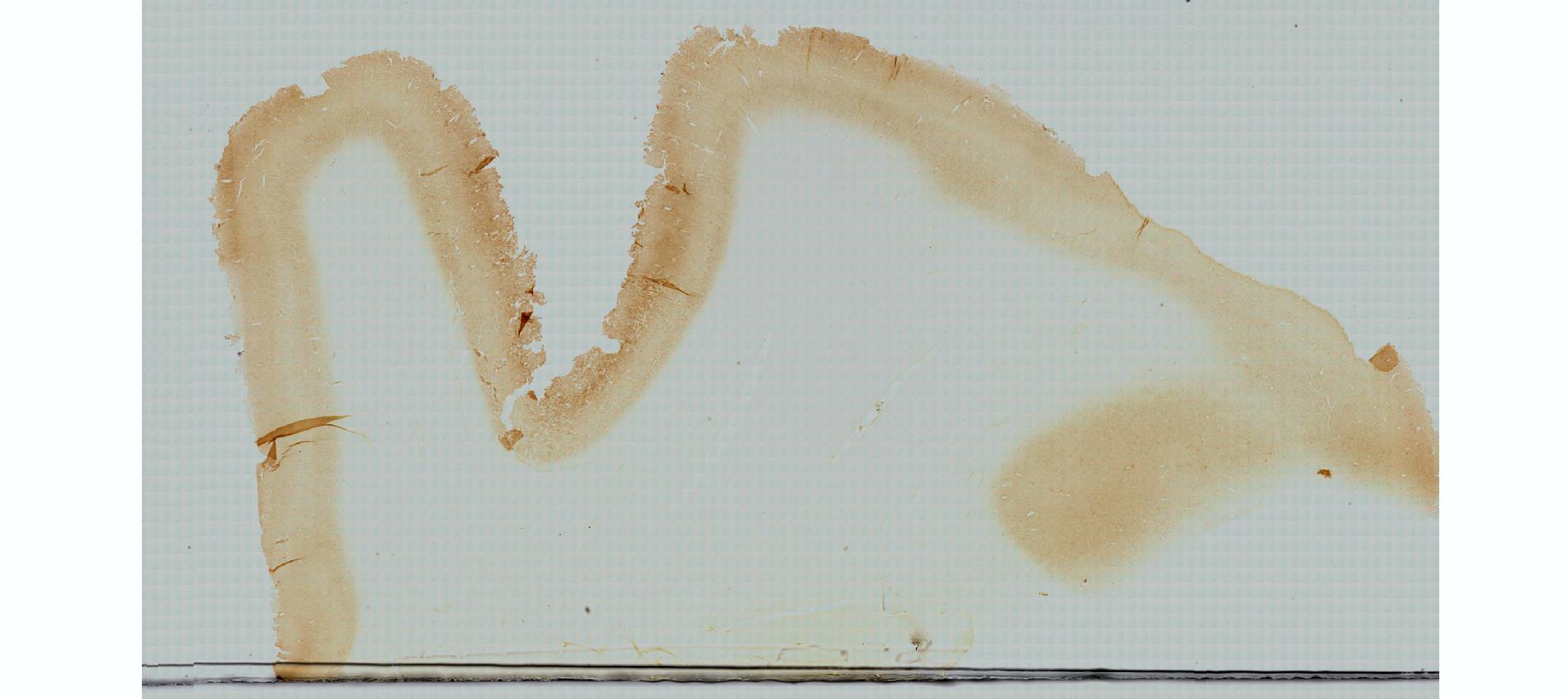

Supplement: Supplementary file 1 [file Presentation_1.ZIP › shank3-immunohistochemistry/case-1/precentral cortex.jpg]

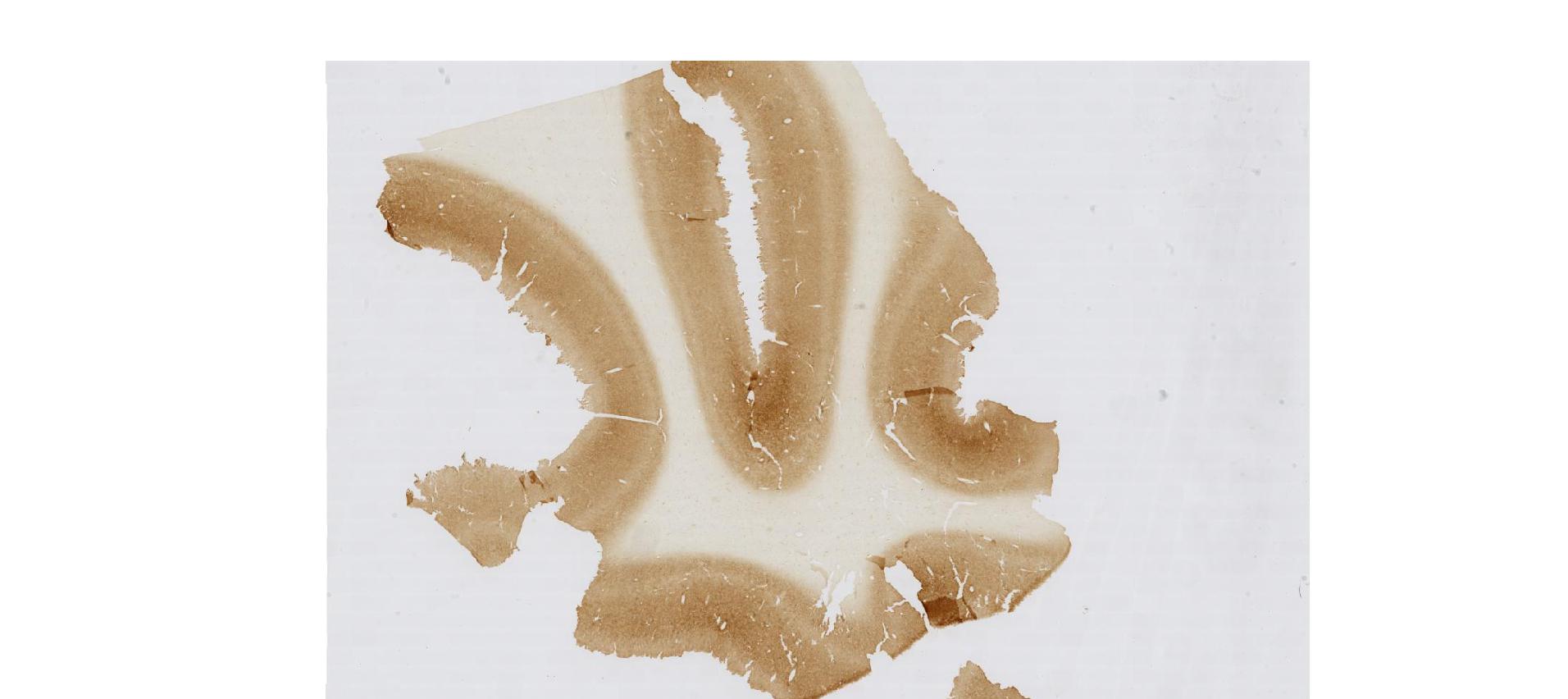

Supplement: Supplementary file 1 [file Presentation_1.ZIP › shank3-immunohistochemistry/case-1/prefrontal cortex.jpg]

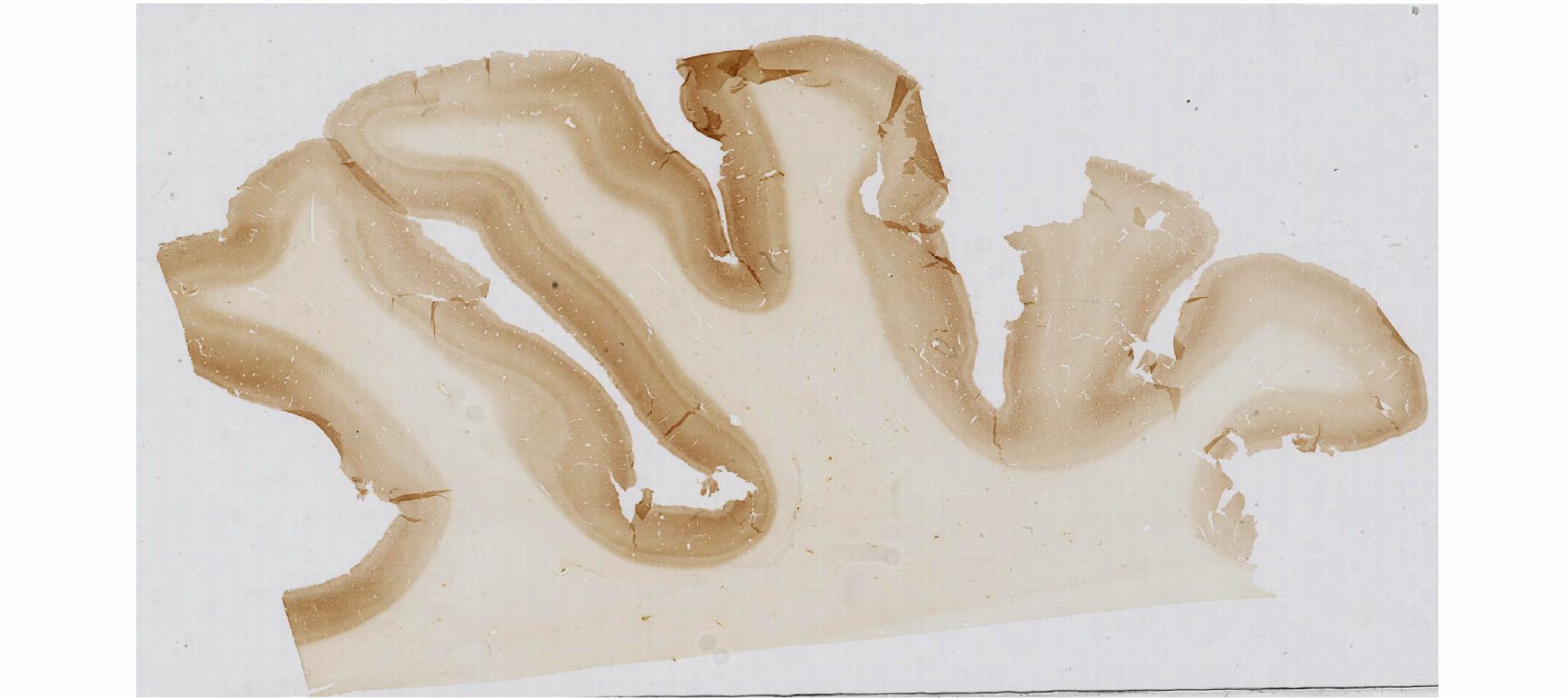

Supplement: Supplementary file 1 [file Presentation_1.ZIP › shank3-immunohistochemistry/case-1/visual cortex.jpg]

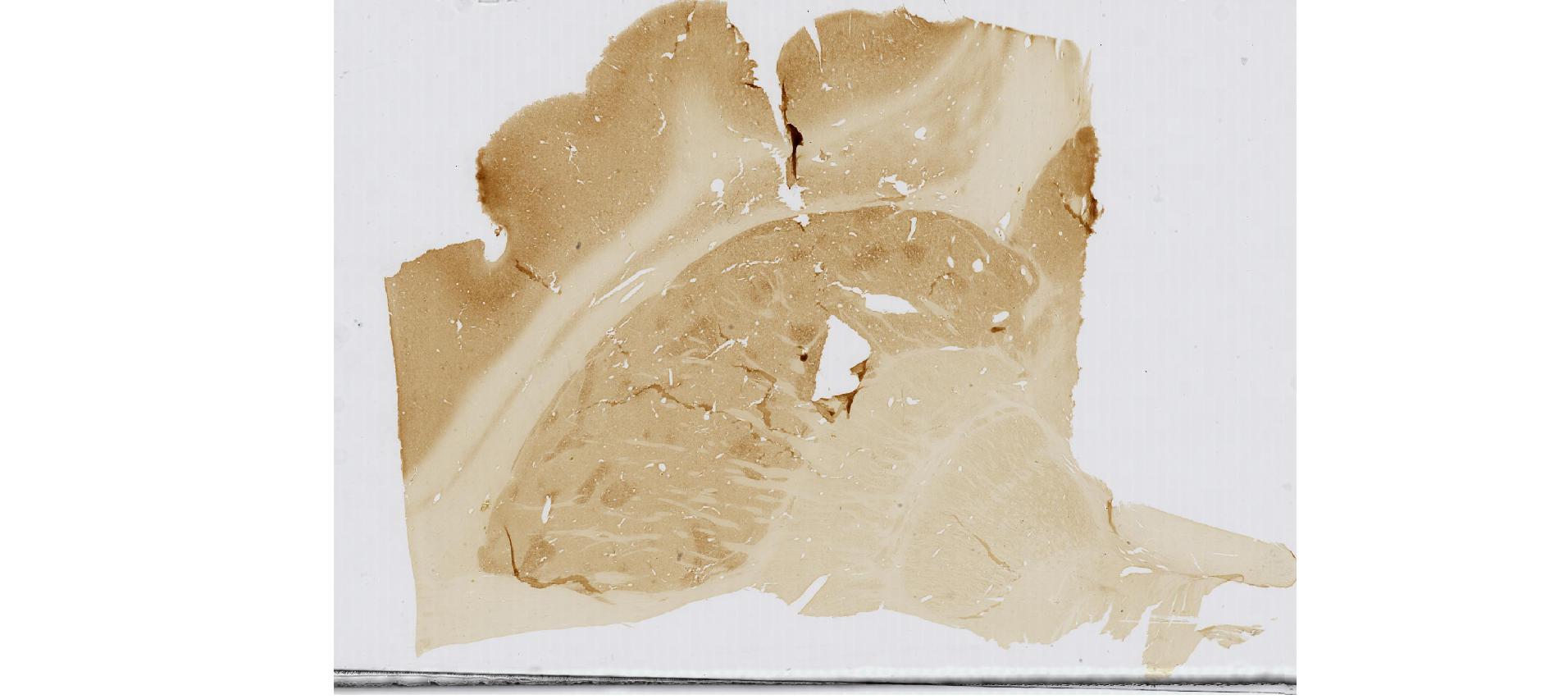

Supplement: Supplementary file 1 [file Presentation_1.ZIP › shank3-immunohistochemistry/case-10/basal ganglia.jpg]

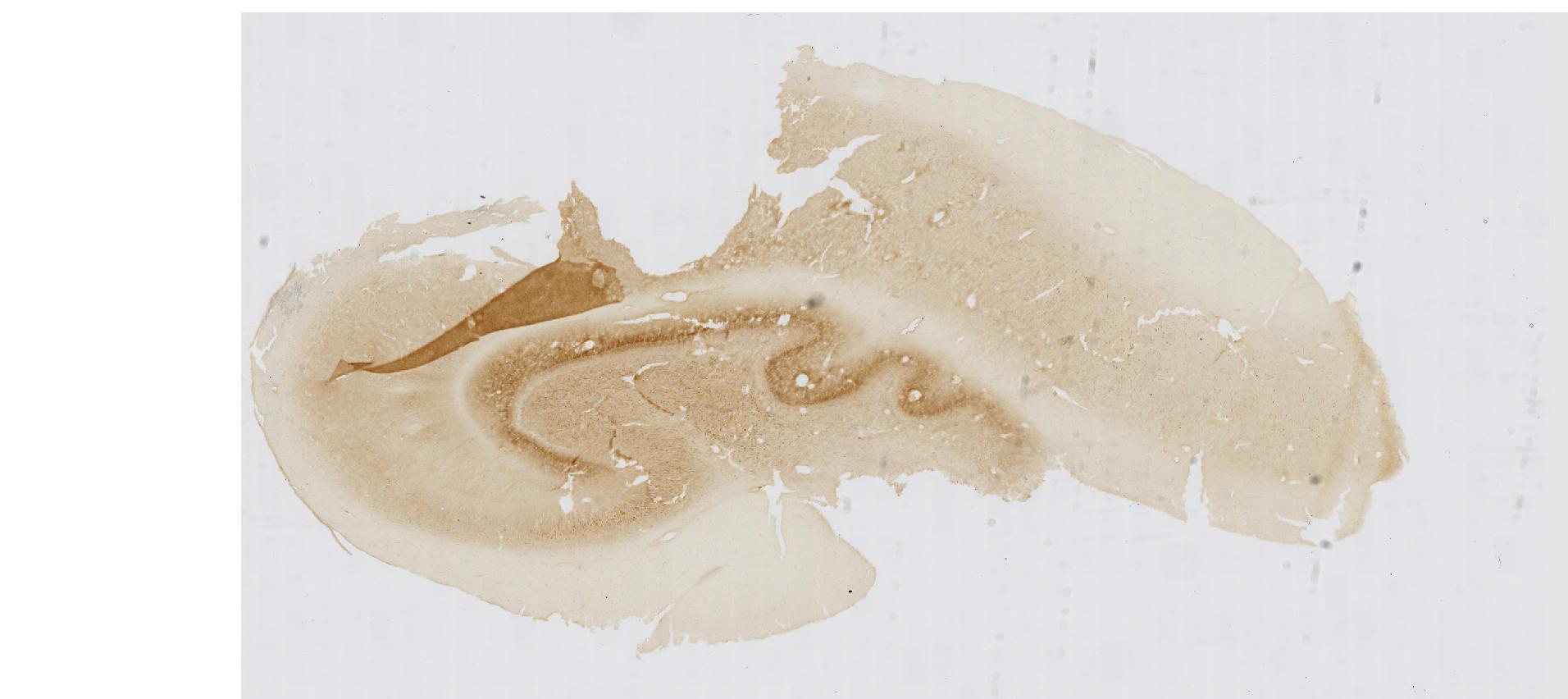

Supplement: Supplementary file 1 [file Presentation_1.ZIP › shank3-immunohistochemistry/case-10/hippocampal formation.jpg]

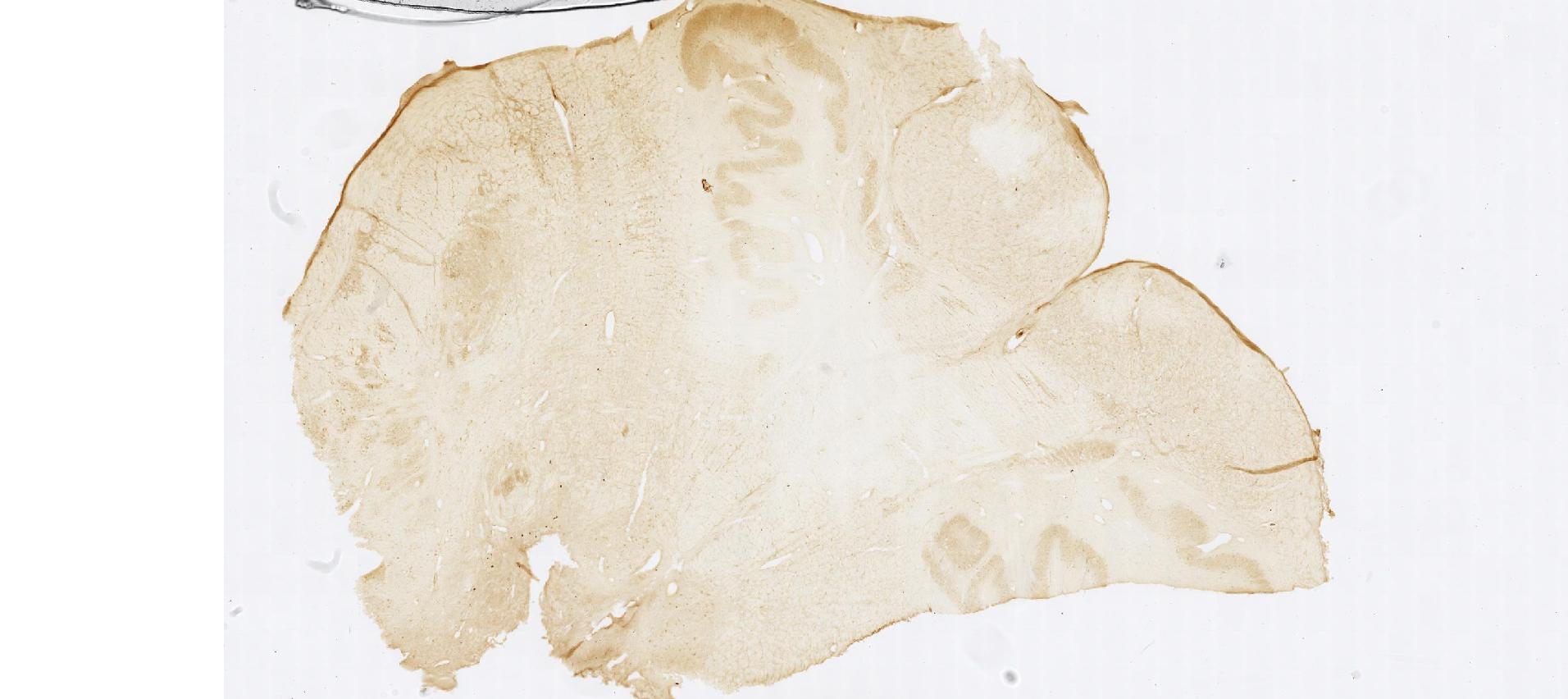

Supplement: Supplementary file 1 [file Presentation_1.ZIP › shank3-immunohistochemistry/case-10/medulla oblongata.jpg]

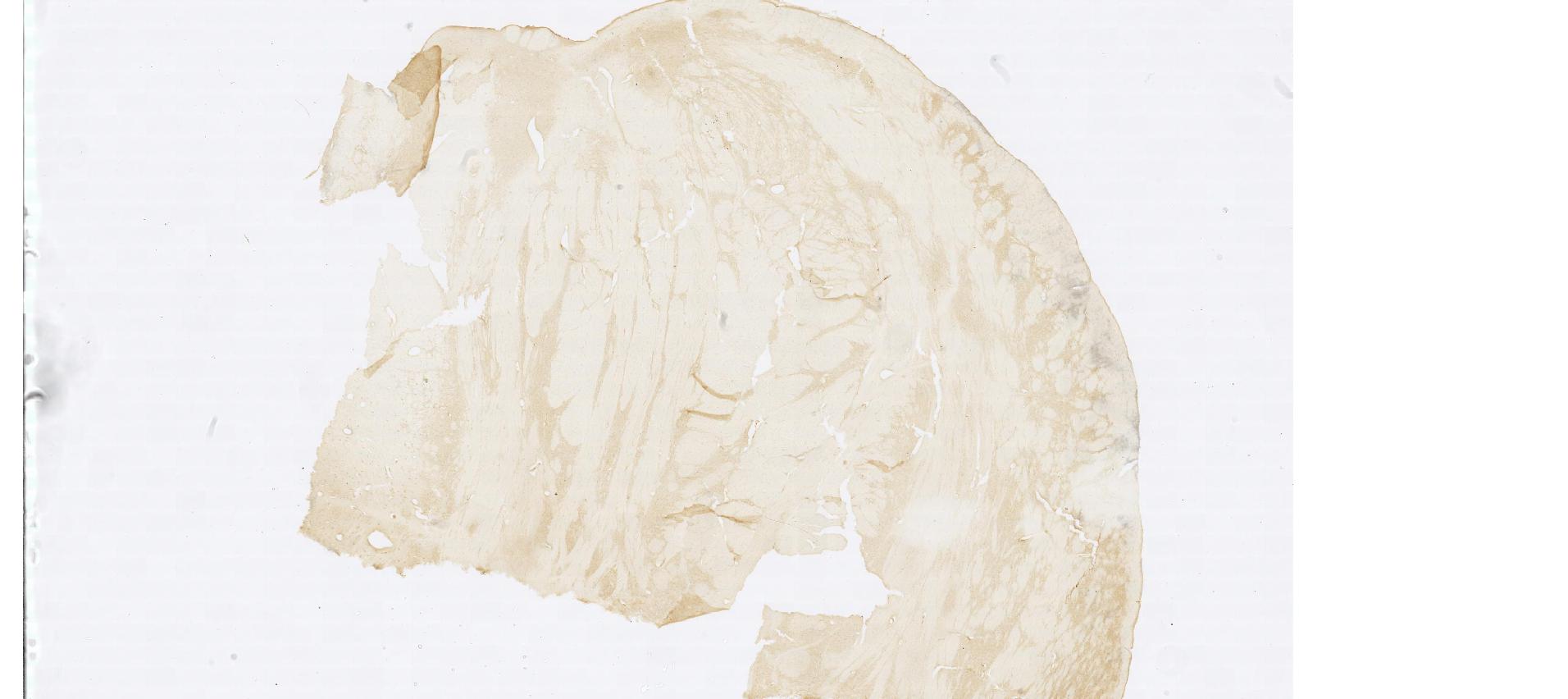

Supplement: Supplementary file 1 [file Presentation_1.ZIP › shank3-immunohistochemistry/case-10/pons.jpg]

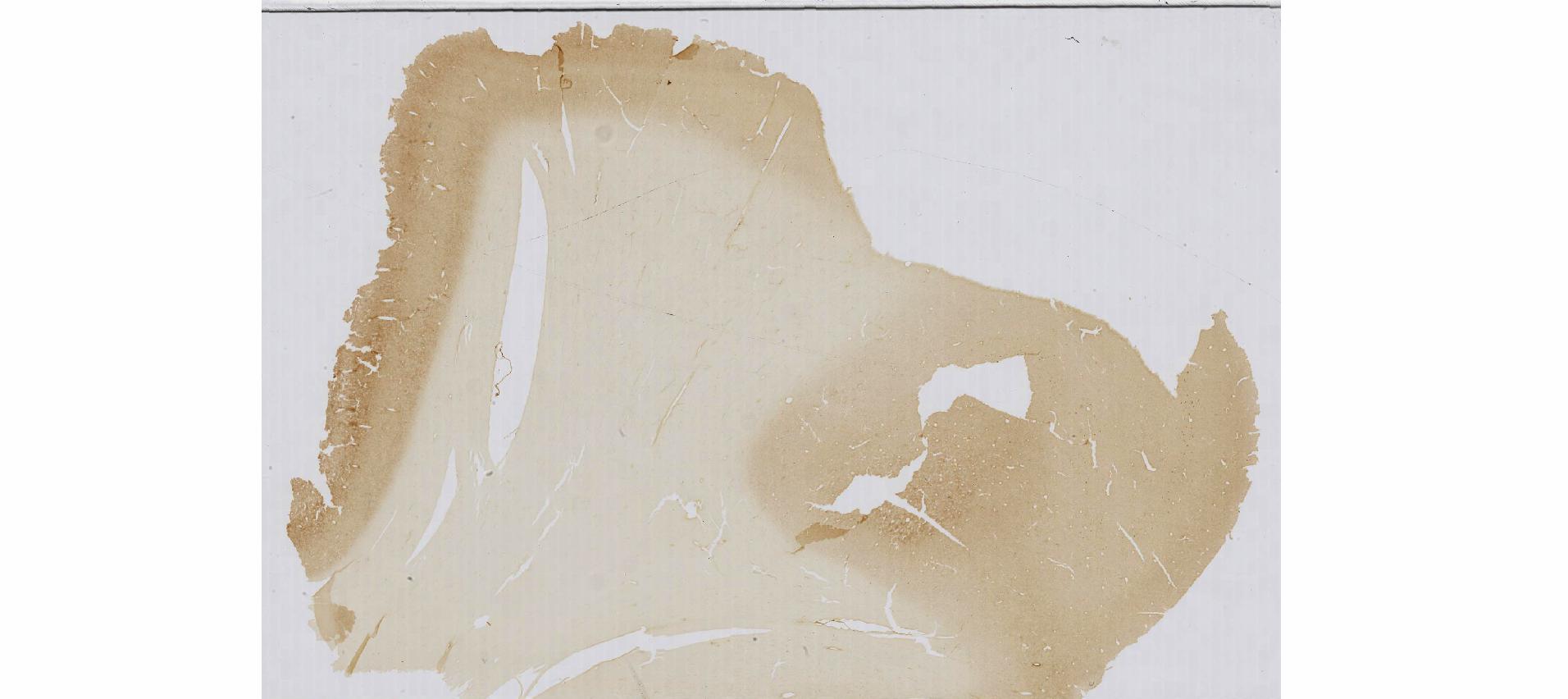

Supplement: Supplementary file 1 [file Presentation_1.ZIP › shank3-immunohistochemistry/case-10/precentral cortex.jpg]

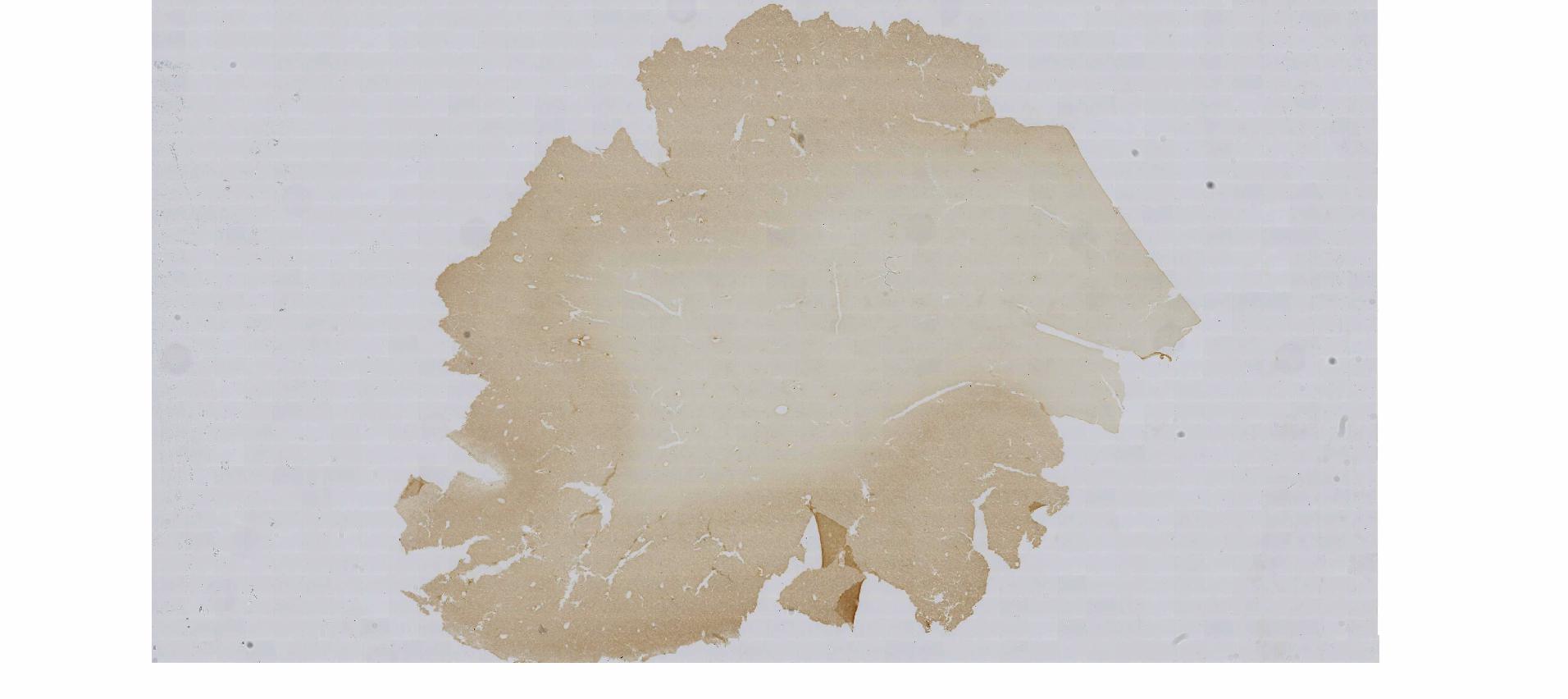

Supplement: Supplementary file 1 [file Presentation_1.ZIP › shank3-immunohistochemistry/case-10/prefrontal cortex.jpg]

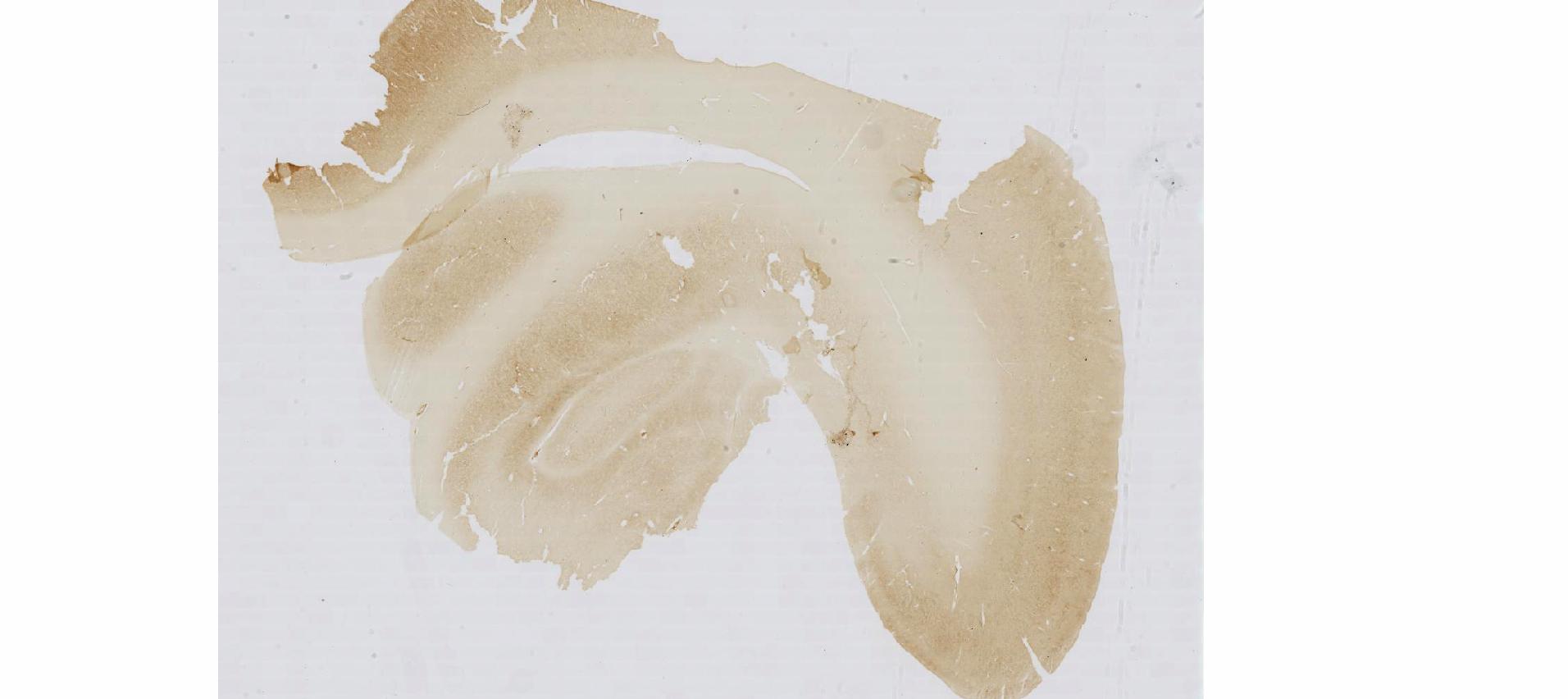

Supplement: Supplementary file 1 [file Presentation_1.ZIP › shank3-immunohistochemistry/case-11/amygdalar.jpg]

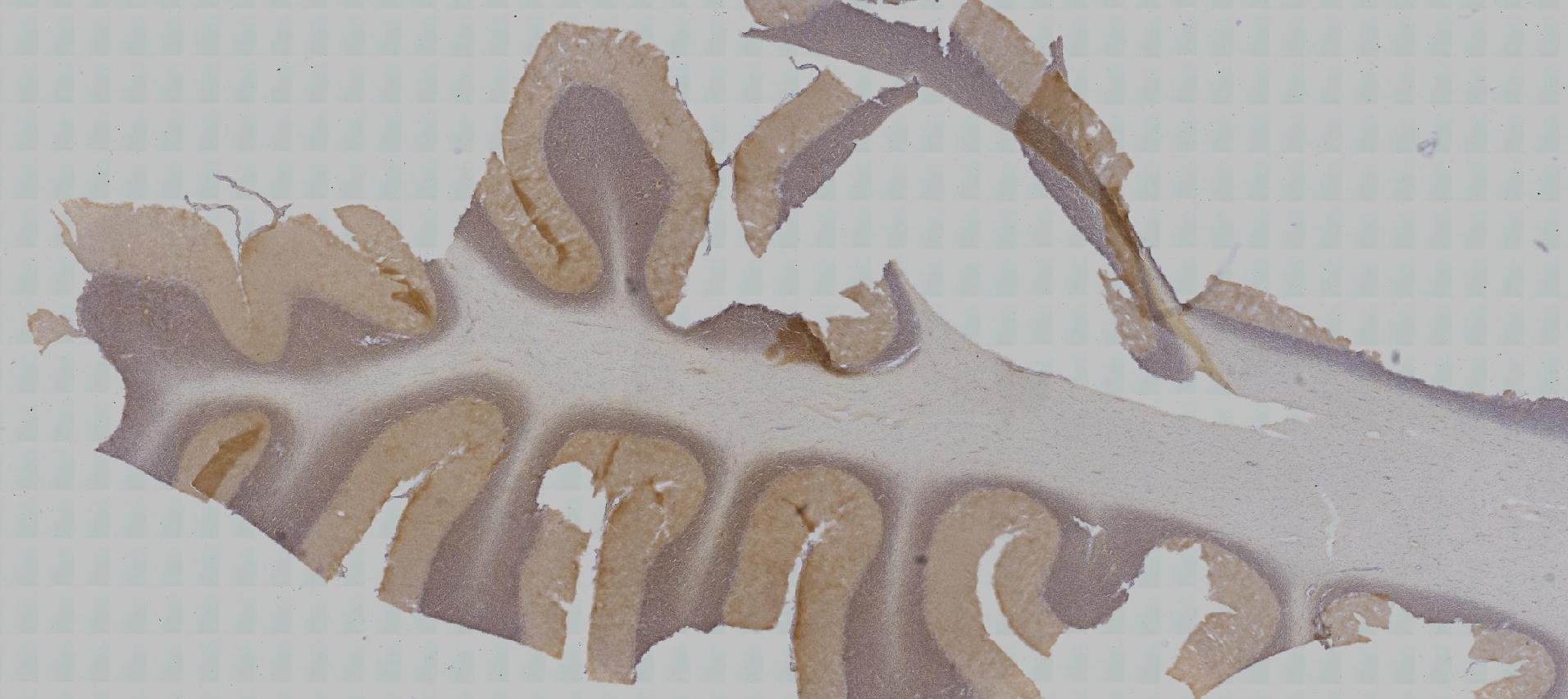

Supplement: Supplementary file 1 [file Presentation_1.ZIP › shank3-immunohistochemistry/case-11/cerebellum.jpg]

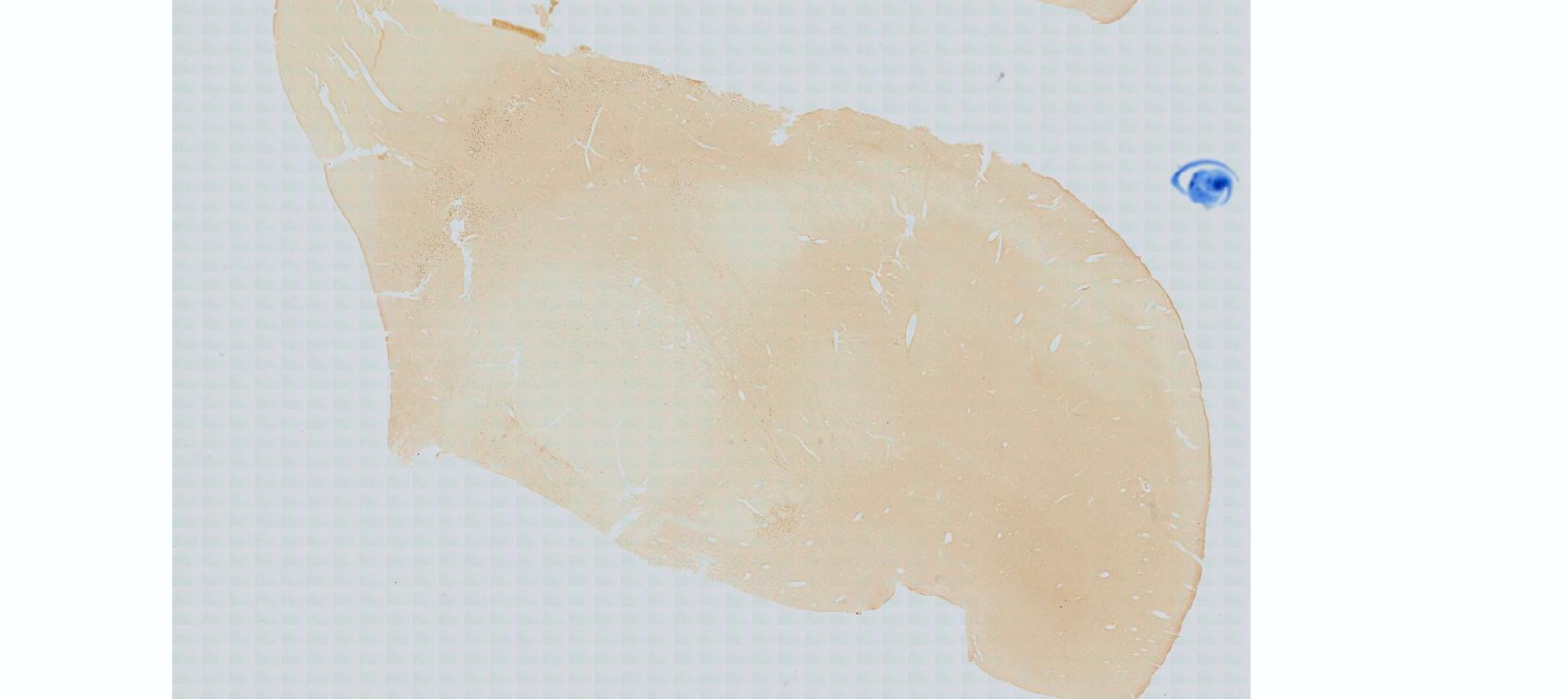

Supplement: Supplementary file 1 [file Presentation_1.ZIP › shank3-immunohistochemistry/case-11/midbrain.jpg]

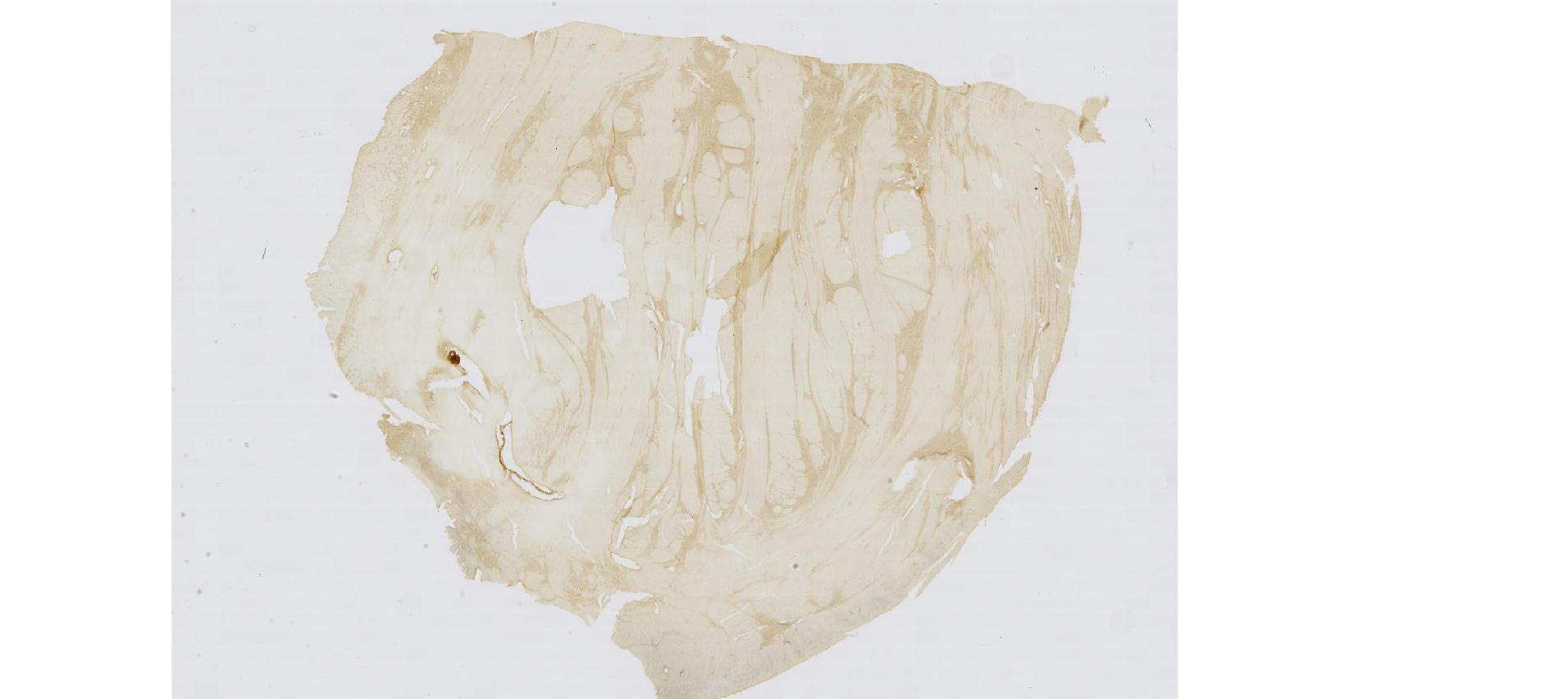

Supplement: Supplementary file 1 [file Presentation_1.ZIP › shank3-immunohistochemistry/case-11/pons.jpg]

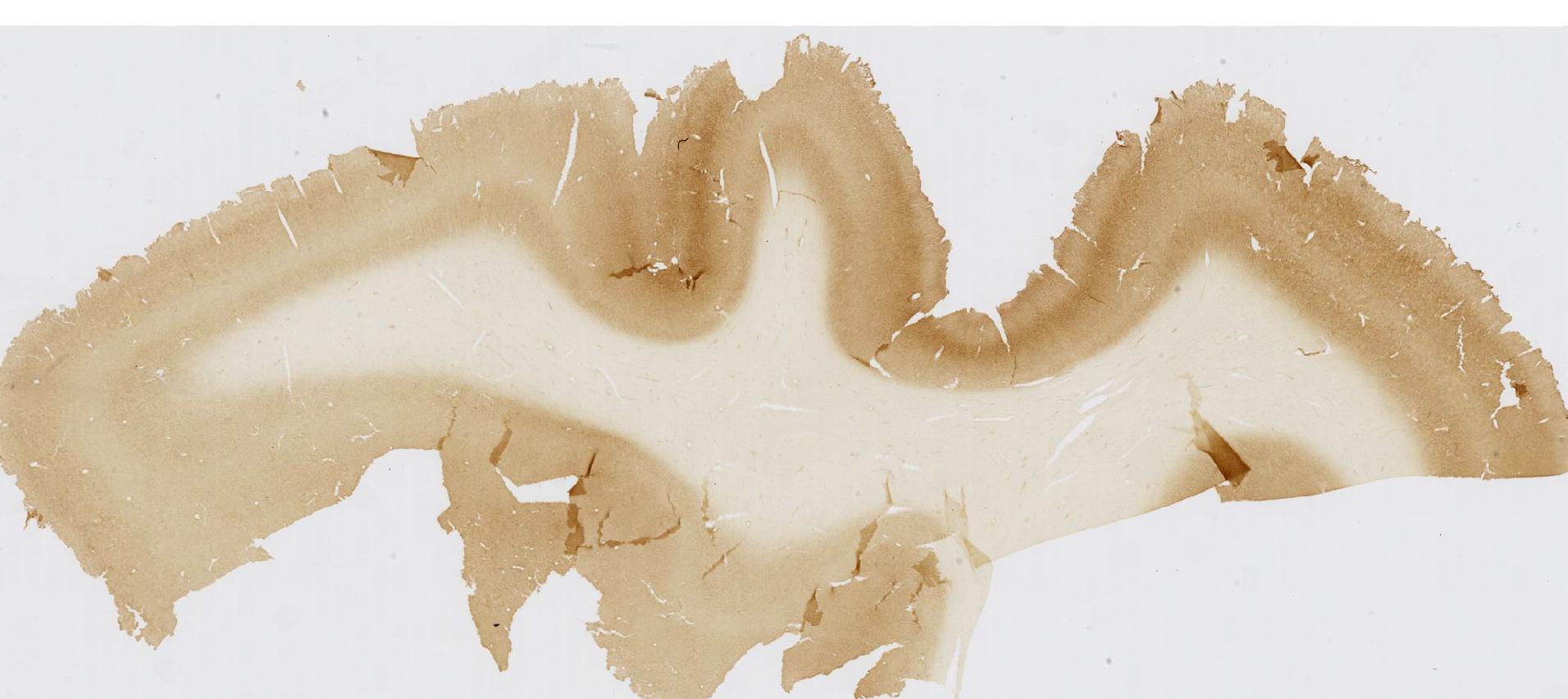

Supplement: Supplementary file 1 [file Presentation_1.ZIP › shank3-immunohistochemistry/case-11/precentral cortex.jpg]

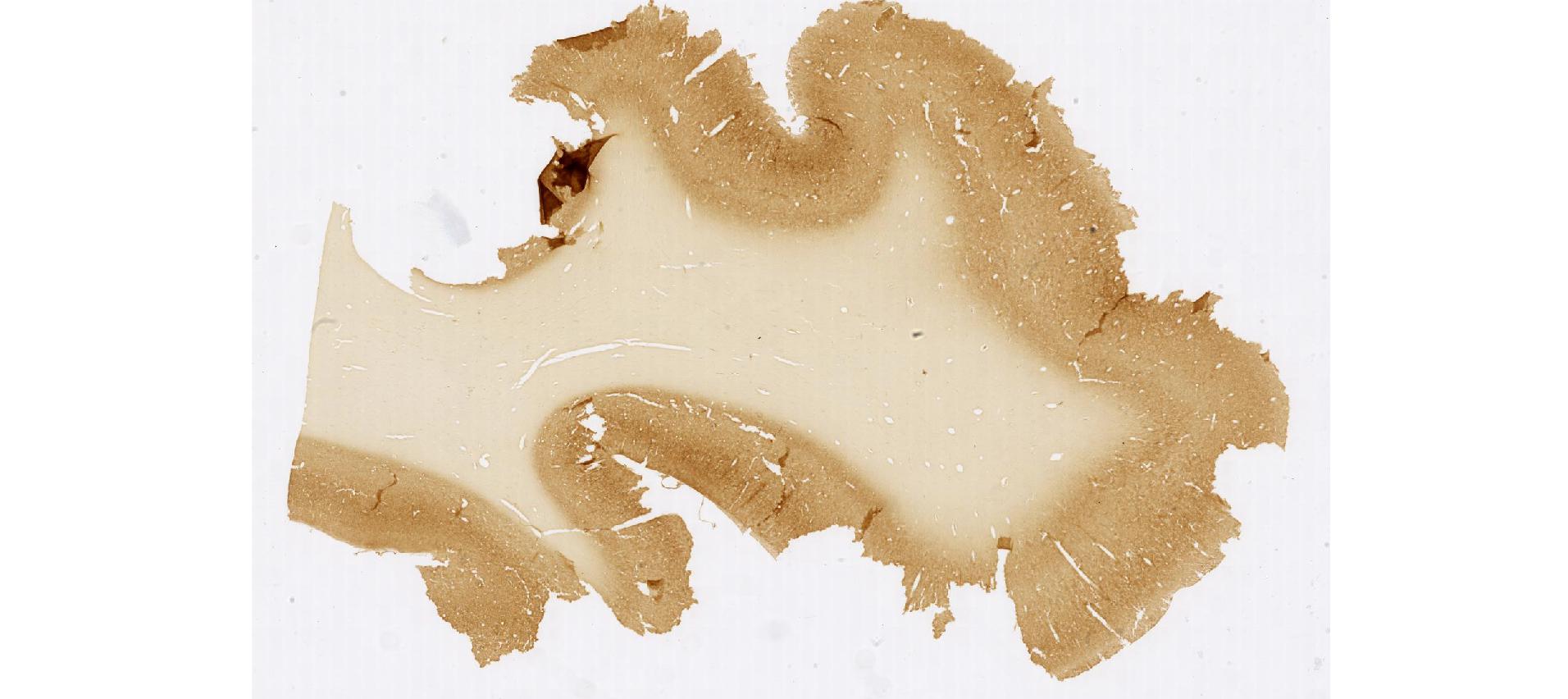

Supplement: Supplementary file 1 [file Presentation_1.ZIP › shank3-immunohistochemistry/case-11/prefrontal cortex.jpg]

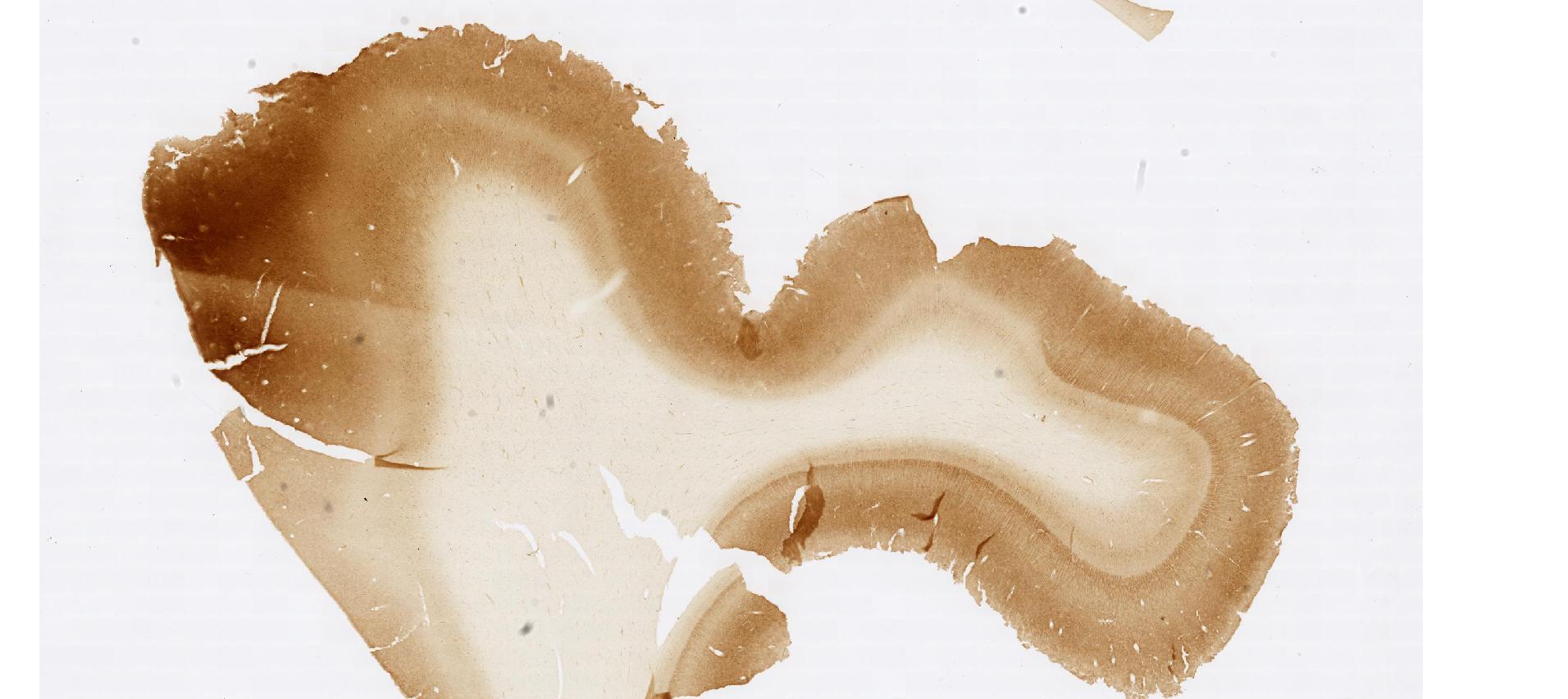

Supplement: Supplementary file 1 [file Presentation_1.ZIP › shank3-immunohistochemistry/case-11/visual cortex.jpg]

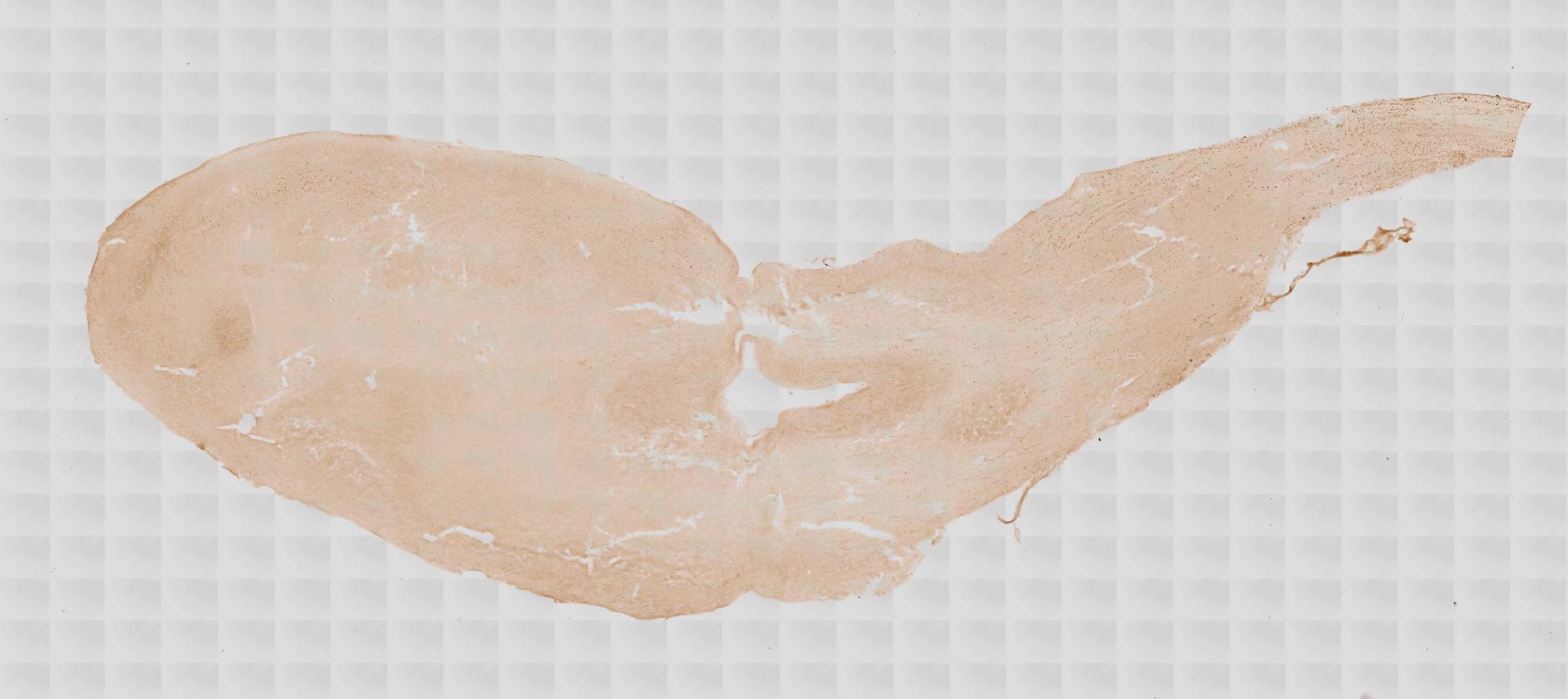

Supplement: Supplementary file 1 [file Presentation_1.ZIP › shank3-immunohistochemistry/case-12/1-olfactory bulb.jpg]

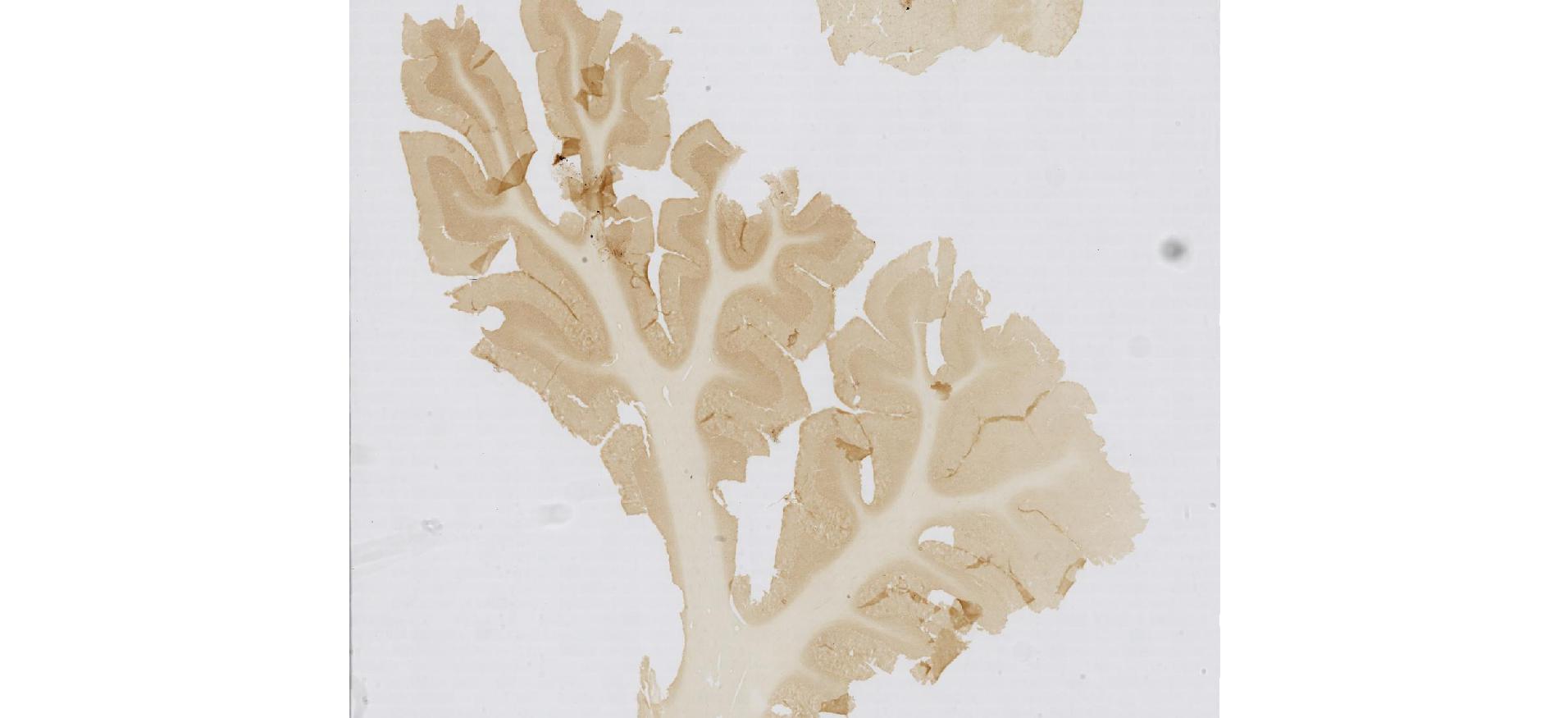

Supplement: Supplementary file 1 [file Presentation_1.ZIP › shank3-immunohistochemistry/case-12/cerebellum.jpg]

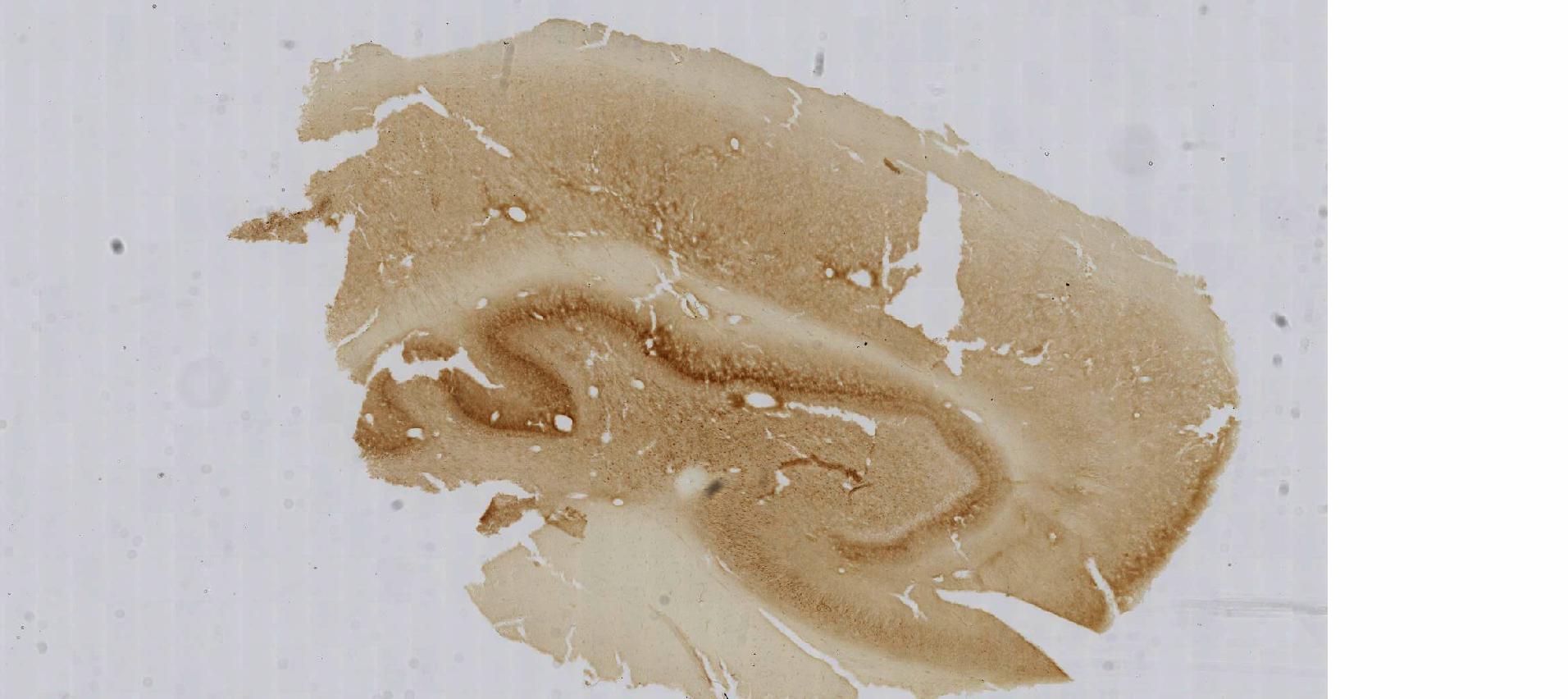

Supplement: Supplementary file 1 [file Presentation_1.ZIP › shank3-immunohistochemistry/case-12/hippocampal formation.jpg]

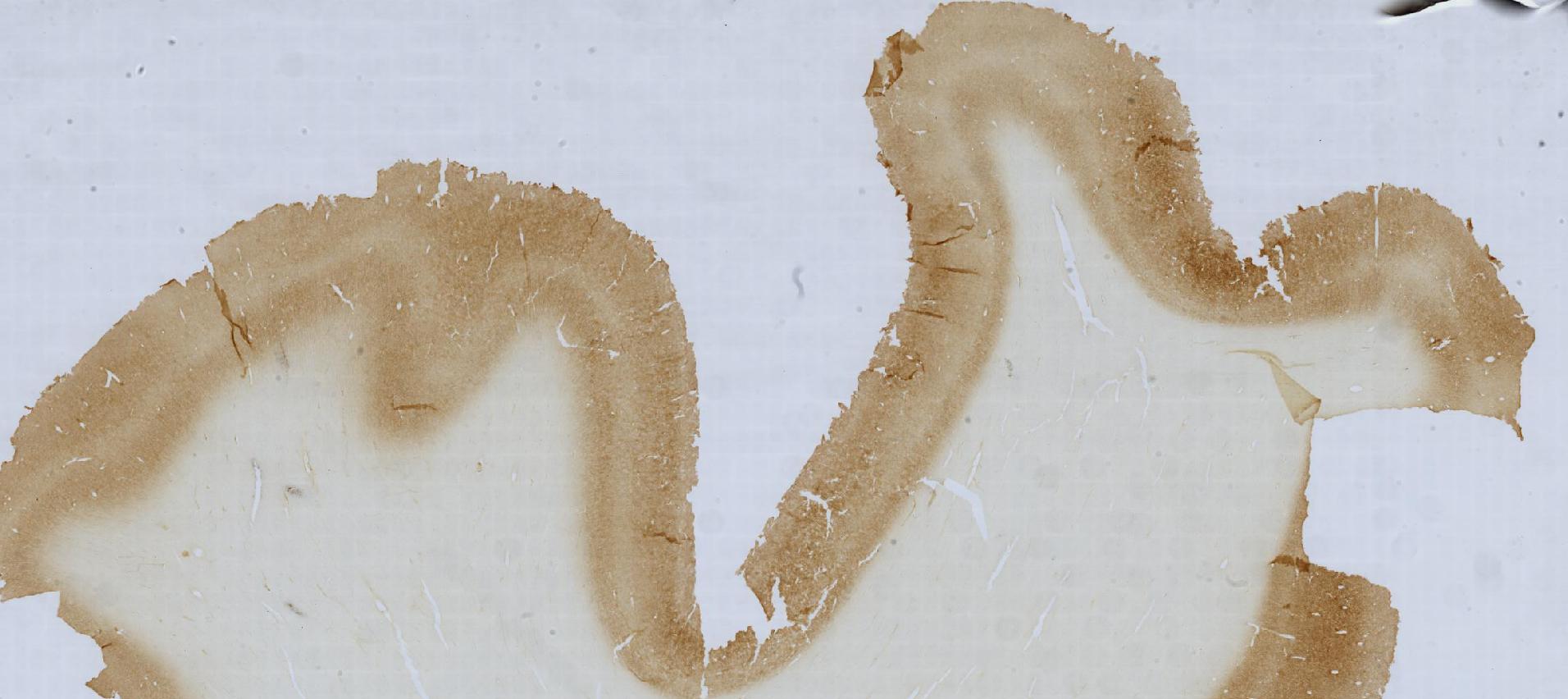

Supplement: Supplementary file 1 [file Presentation_1.ZIP › shank3-immunohistochemistry/case-12/postcentral cortex.jpg]

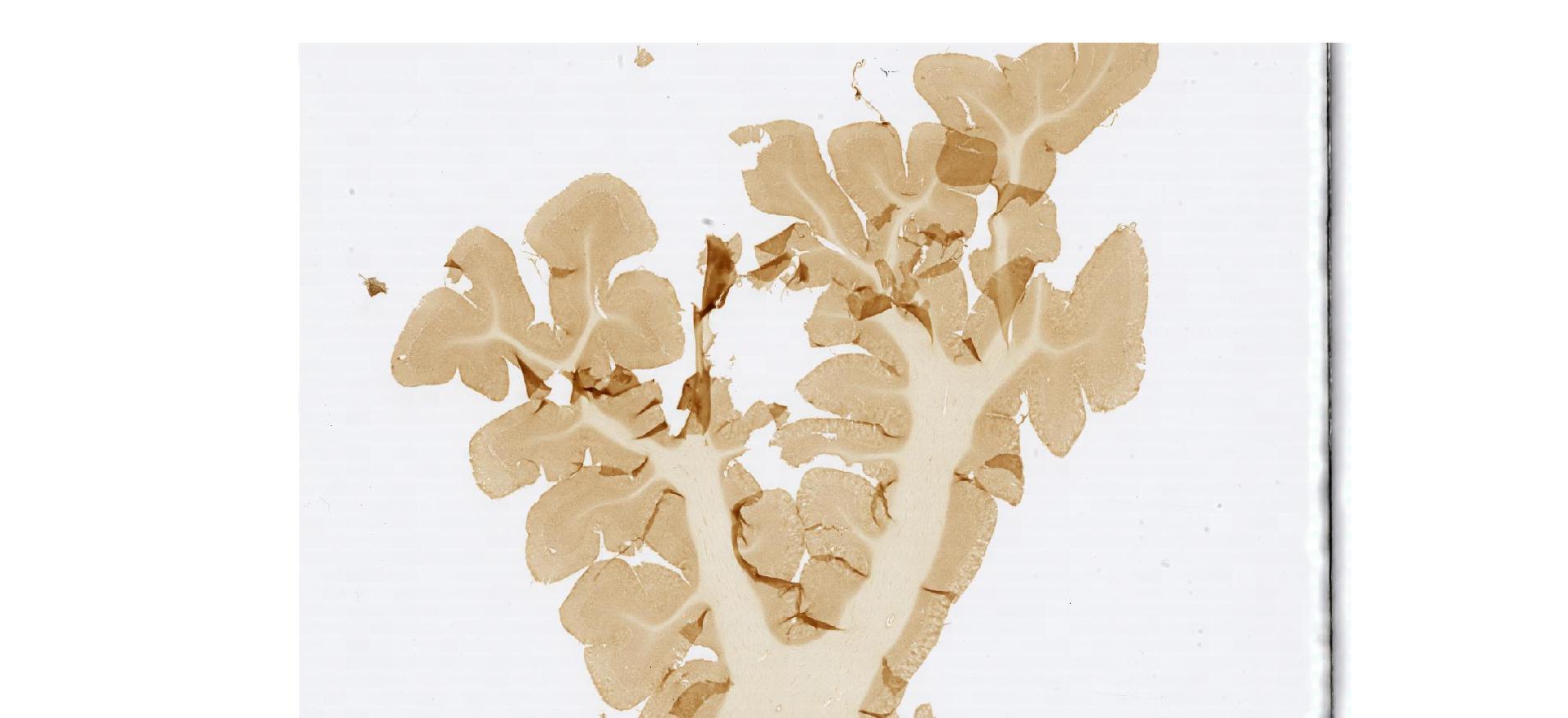

Supplement: Supplementary file 1 [file Presentation_1.ZIP › shank3-immunohistochemistry/case-13/cerebellum.jpg]

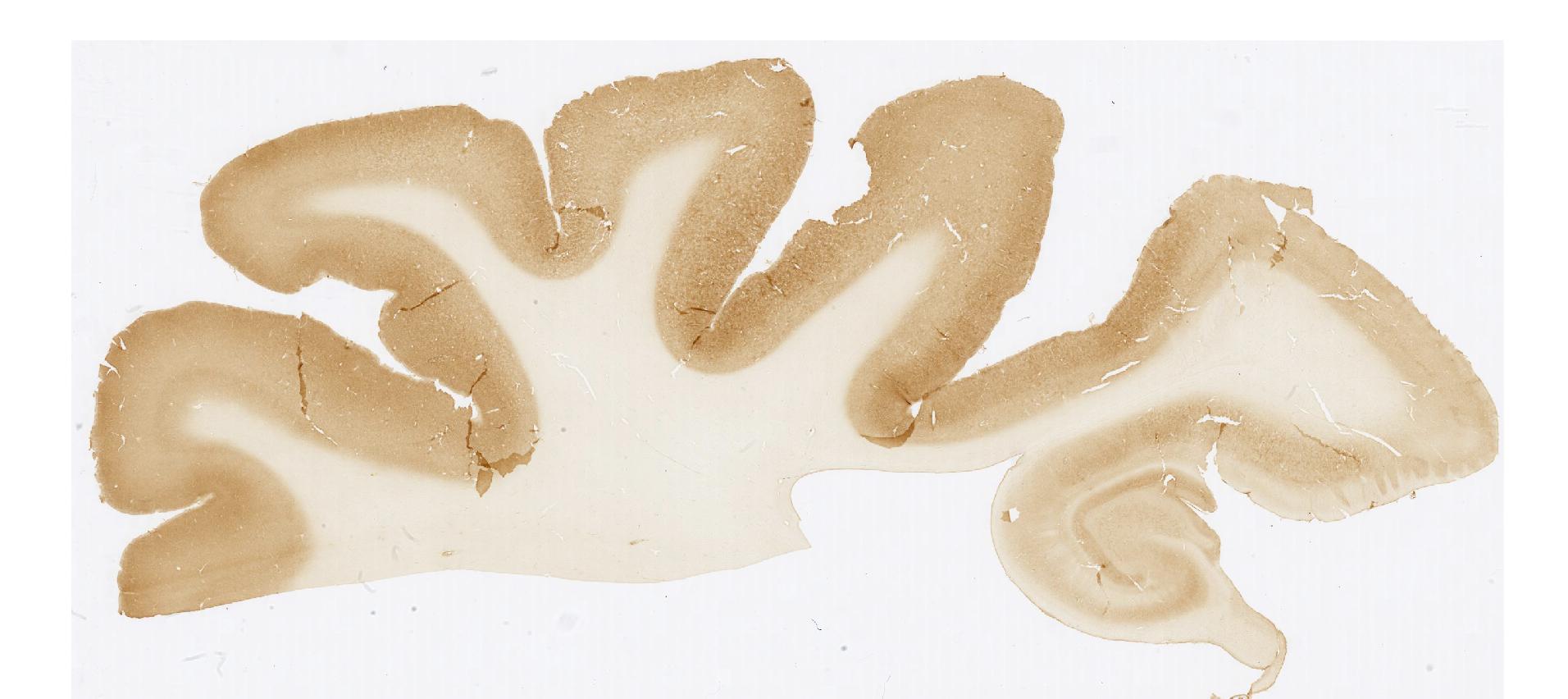

Supplement: Supplementary file 1 [file Presentation_1.ZIP › shank3-immunohistochemistry/case-13/hippocampal formation.jpg]

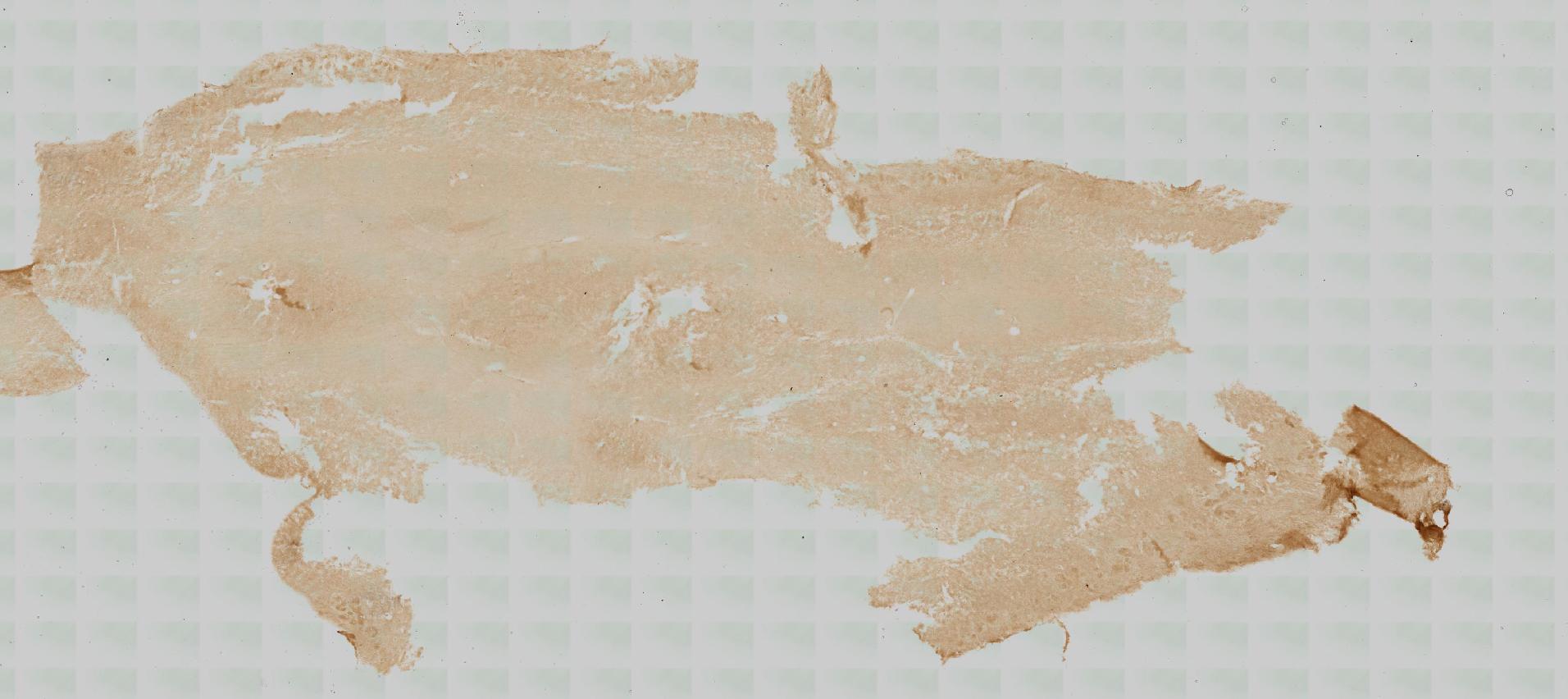

Supplement: Supplementary file 1 [file Presentation_1.ZIP › shank3-immunohistochemistry/case-13/olfactory bulb.jpg]

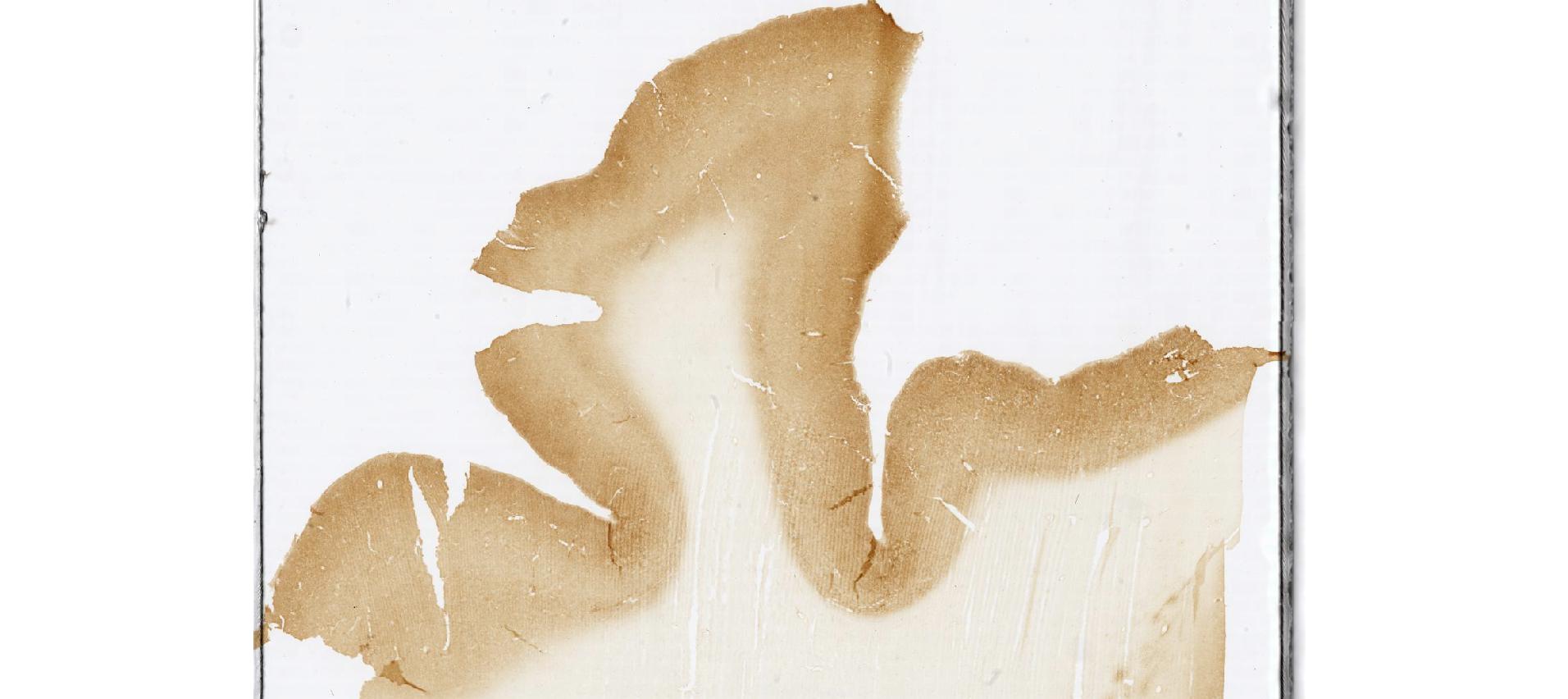

Supplement: Supplementary file 1 [file Presentation_1.ZIP › shank3-immunohistochemistry/case-13/postcentral cortex.jpg]

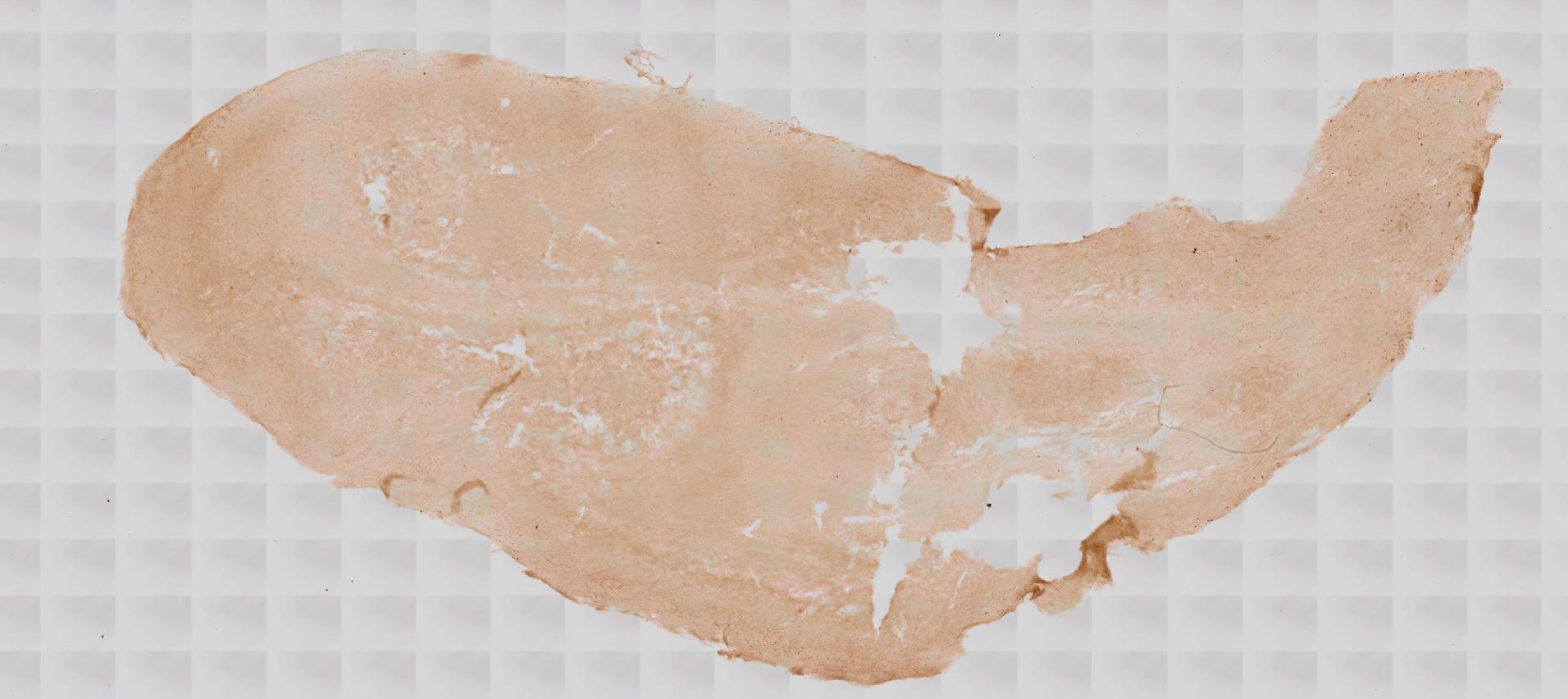

Supplement: Supplementary file 1 [file Presentation_1.ZIP › shank3-immunohistochemistry/case-14/1-olfactory bulb.jpg]

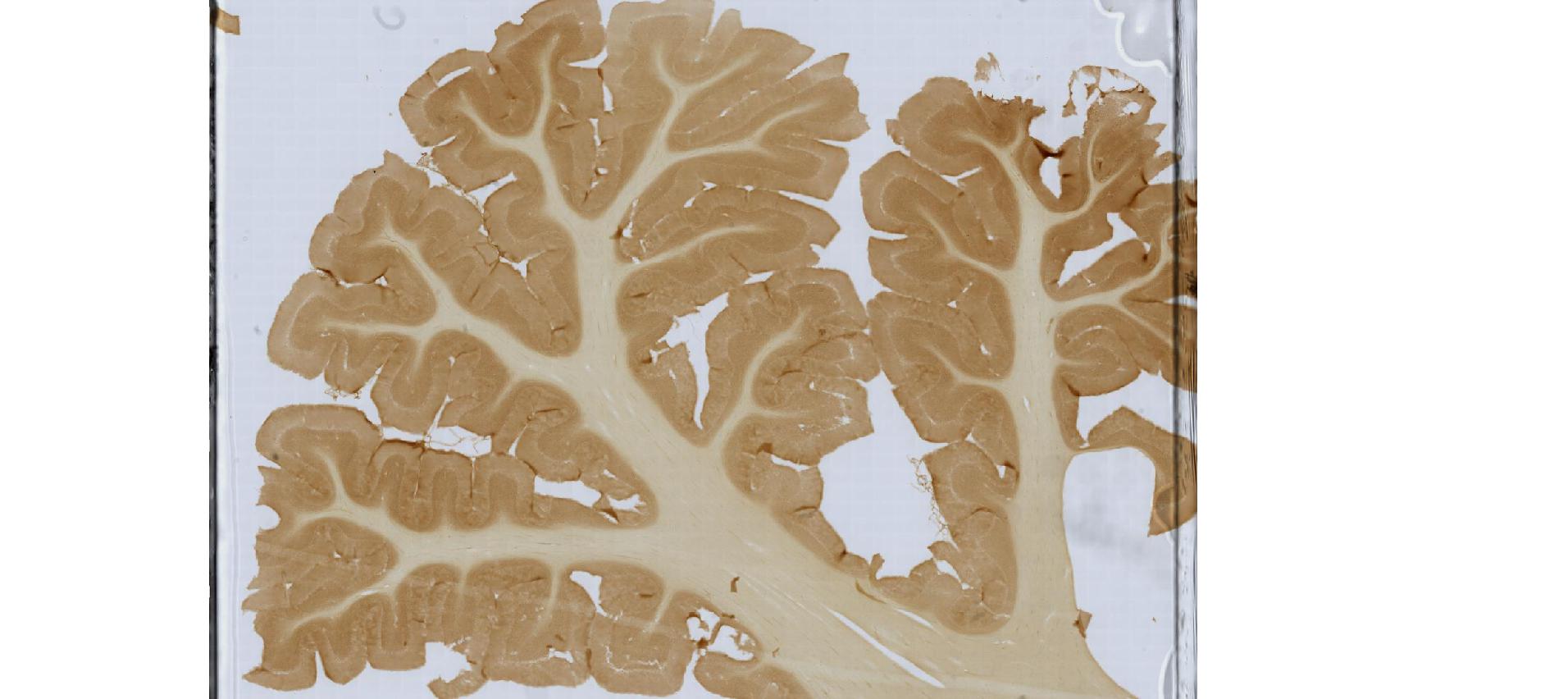

Supplement: Supplementary file 1 [file Presentation_1.ZIP › shank3-immunohistochemistry/case-14/10-cerebellum.jpg]

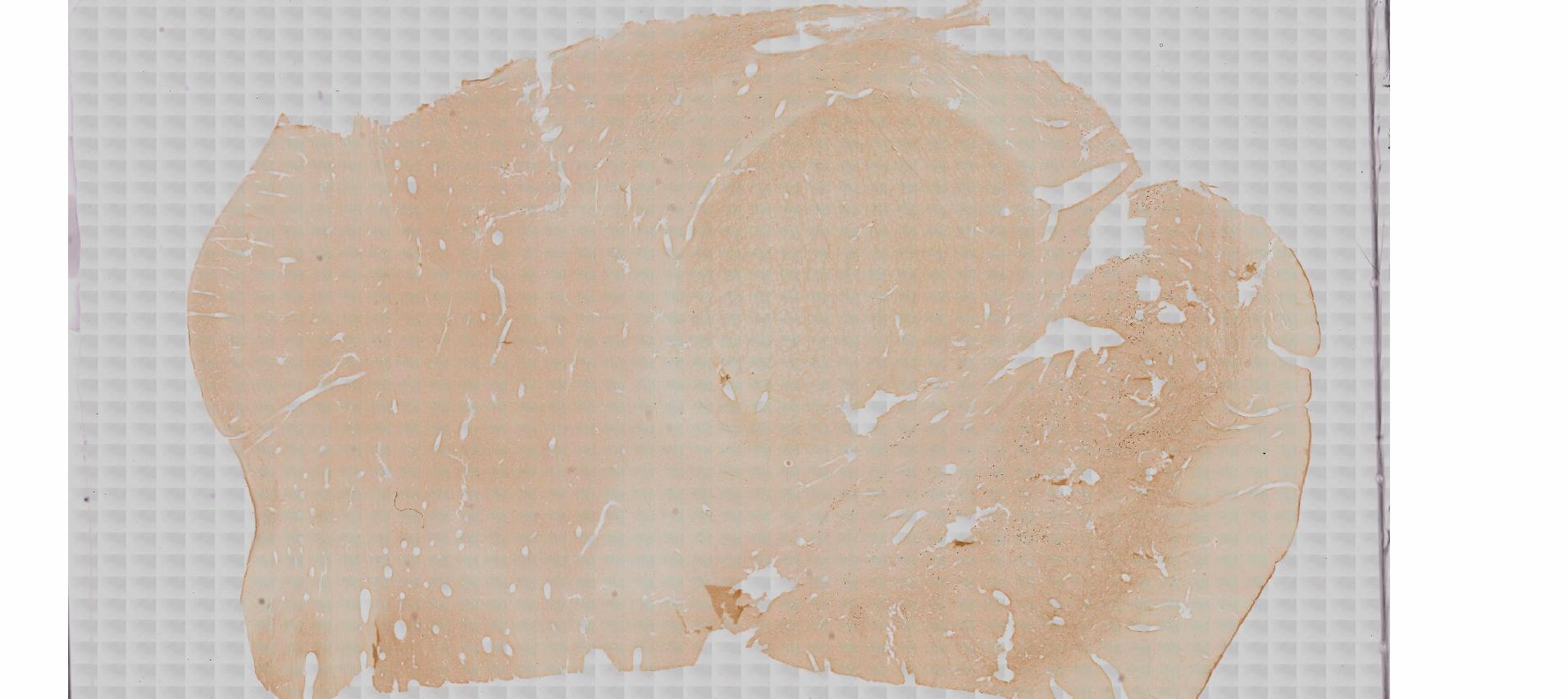

Supplement: Supplementary file 1 [file Presentation_1.ZIP › shank3-immunohistochemistry/case-14/11-midbrain.jpg]

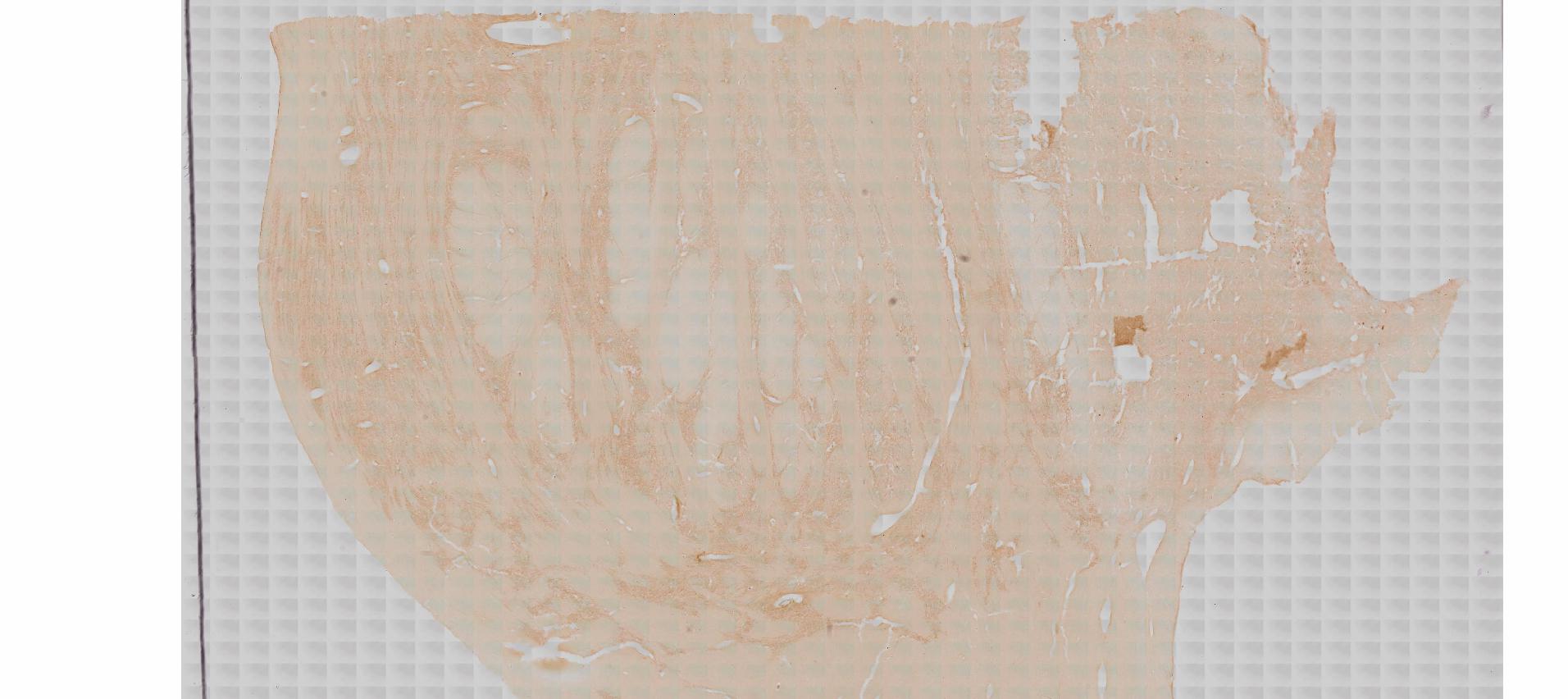

Supplement: Supplementary file 1 [file Presentation_1.ZIP › shank3-immunohistochemistry/case-14/12-pons.jpg]

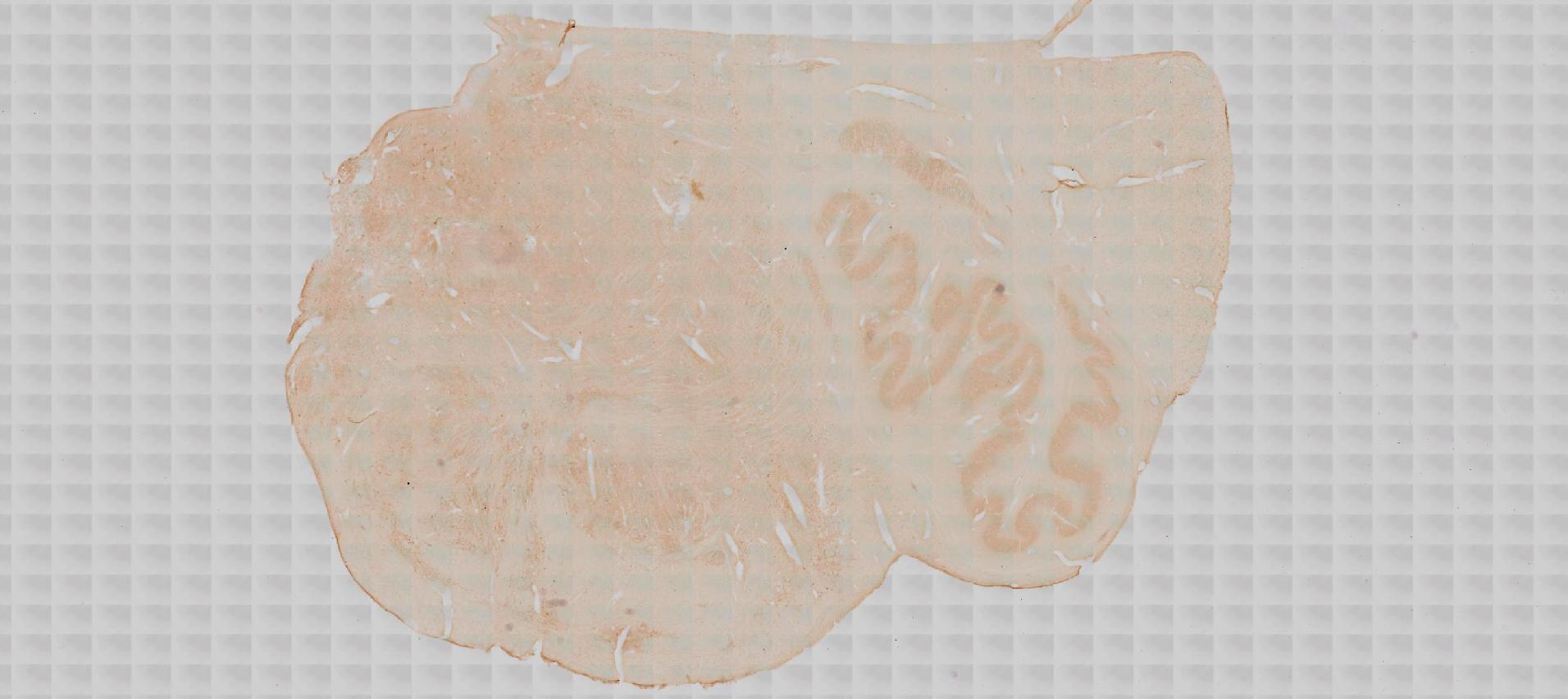

Supplement: Supplementary file 1 [file Presentation_1.ZIP › shank3-immunohistochemistry/case-14/13-medulla oblongata.jpg]

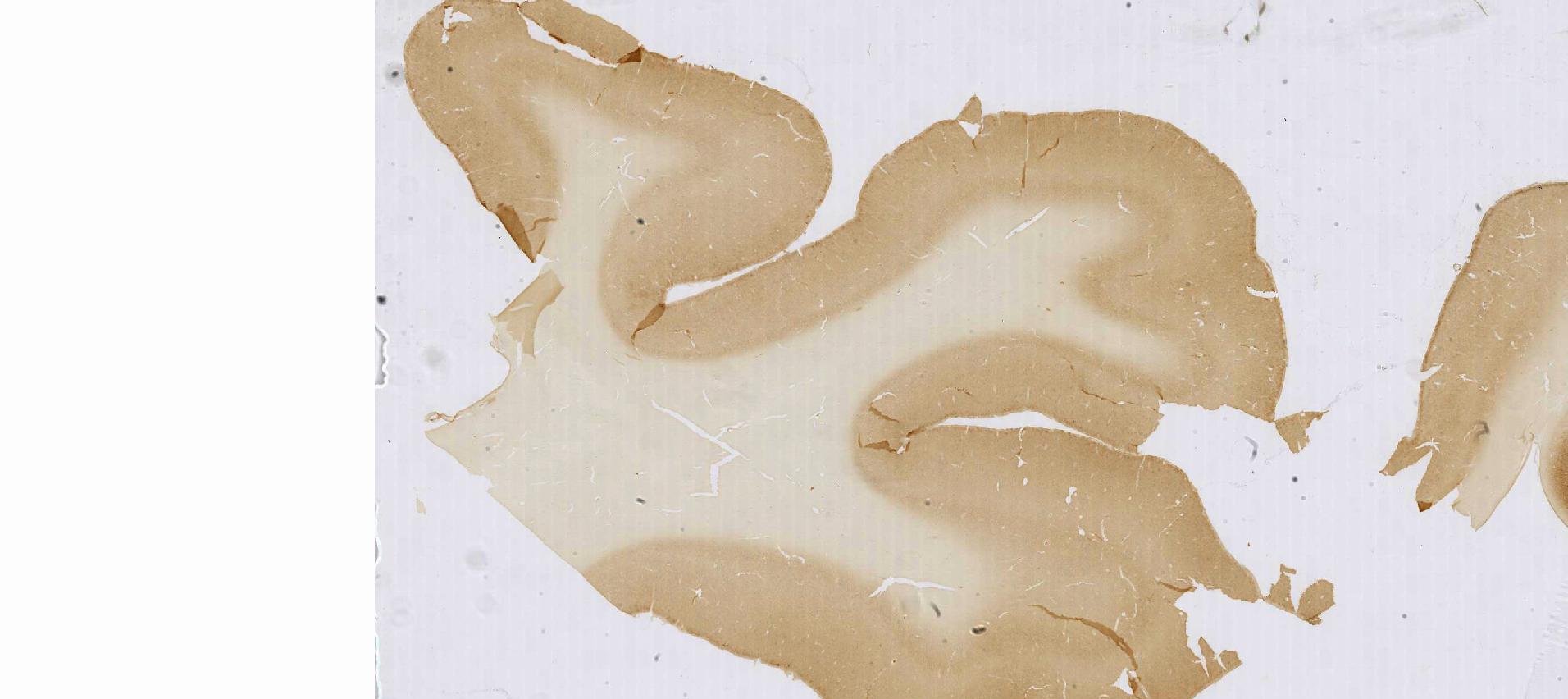

Supplement: Supplementary file 1 [file Presentation_1.ZIP › shank3-immunohistochemistry/case-14/2-prefrontal cortex.jpg]

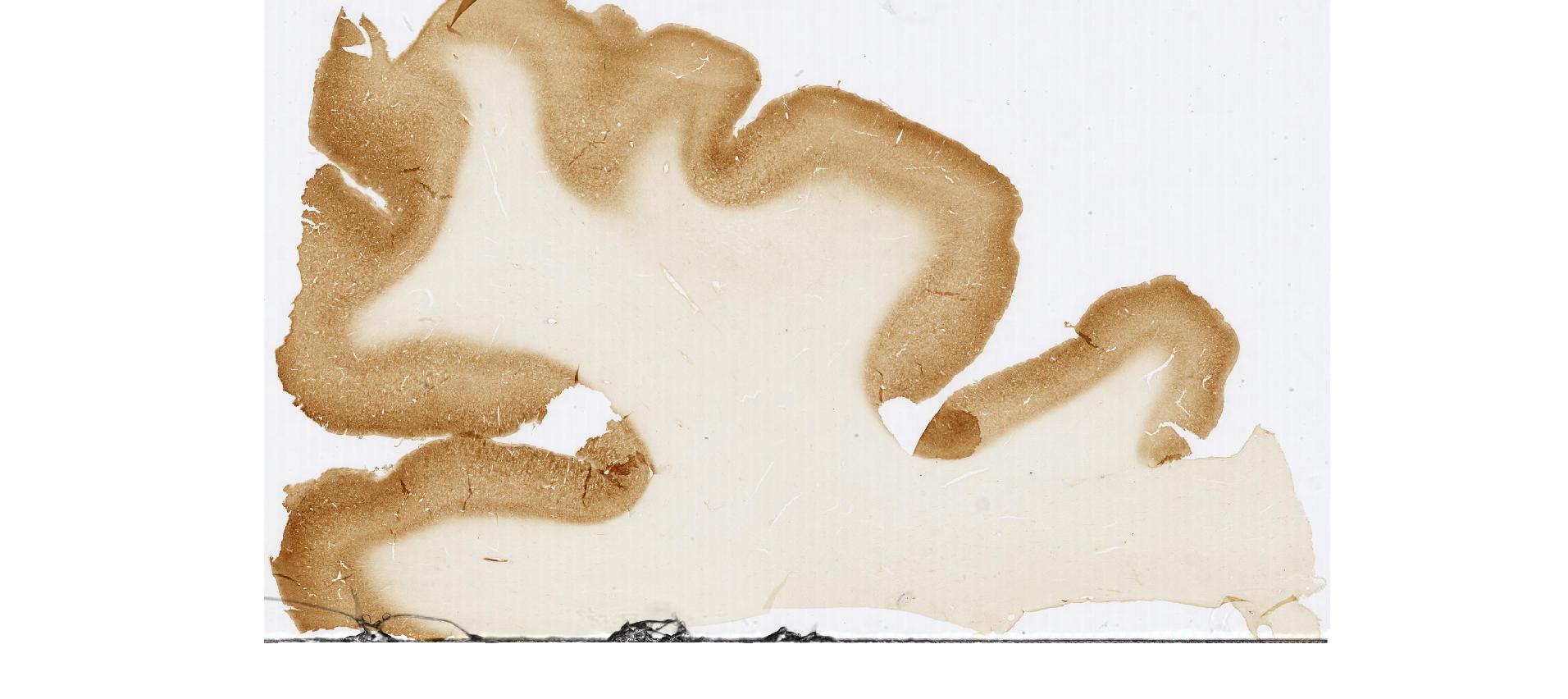

Supplement: Supplementary file 1 [file Presentation_1.ZIP › shank3-immunohistochemistry/case-14/3-anterior cingulate neocortex.jpg]

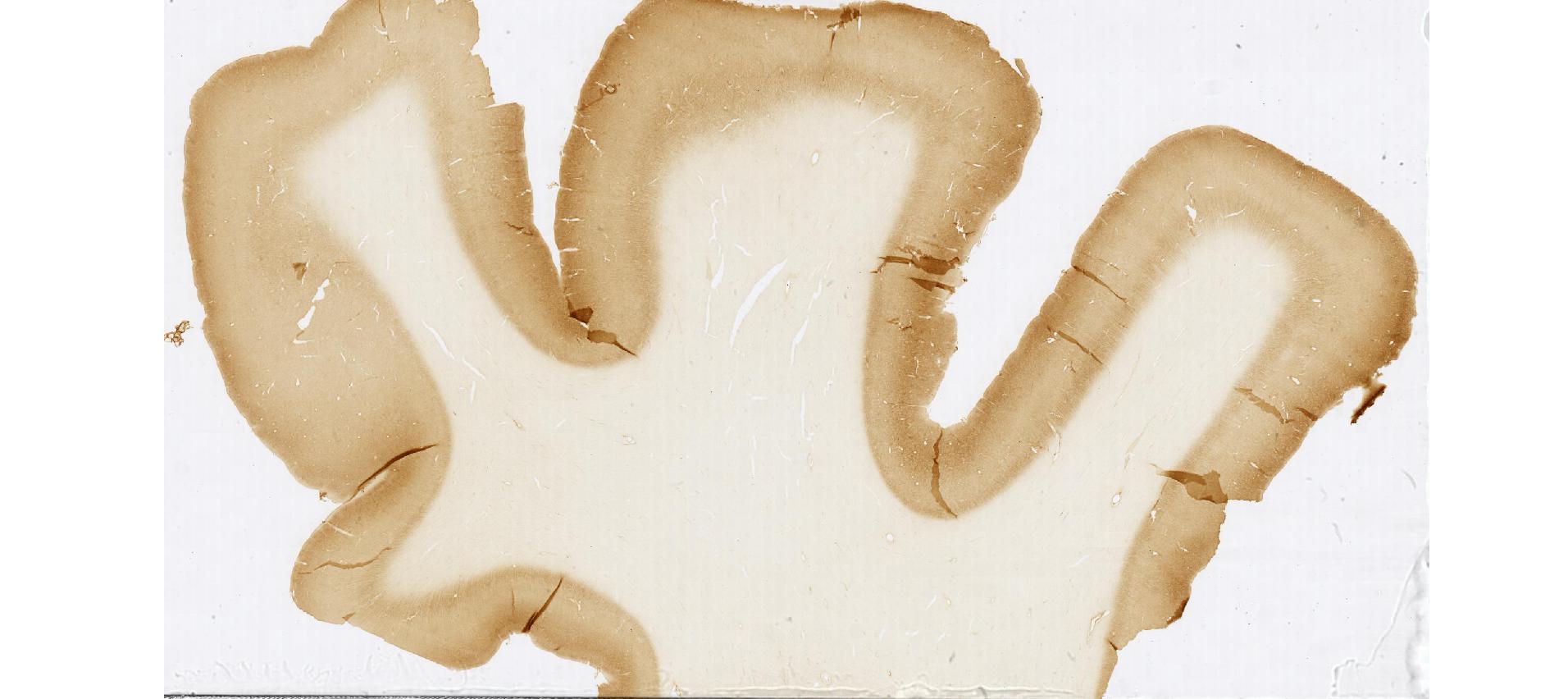

Supplement: Supplementary file 1 [file Presentation_1.ZIP › shank3-immunohistochemistry/case-14/4- precentral cortex.jpg]

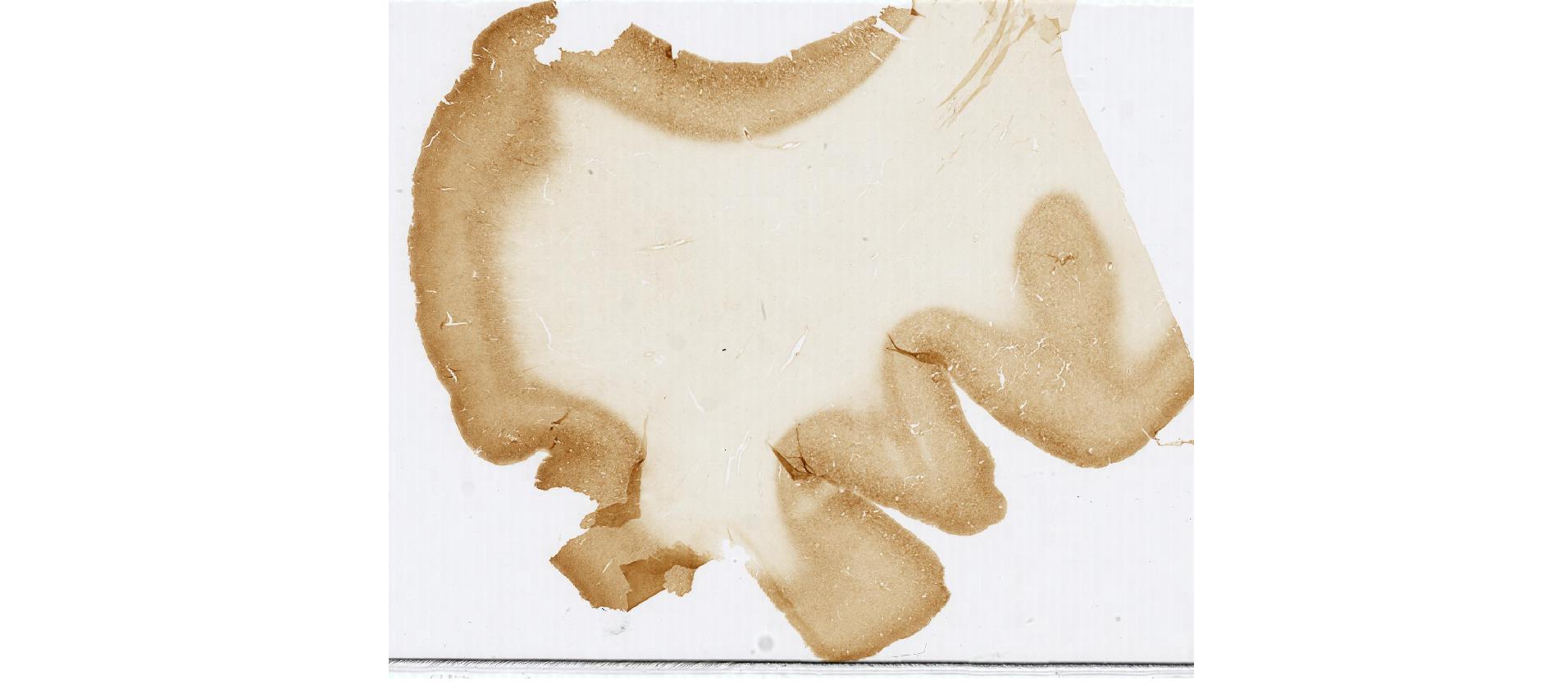

Supplement: Supplementary file 1 [file Presentation_1.ZIP › shank3-immunohistochemistry/case-14/5-postcentral cortex.jpg]

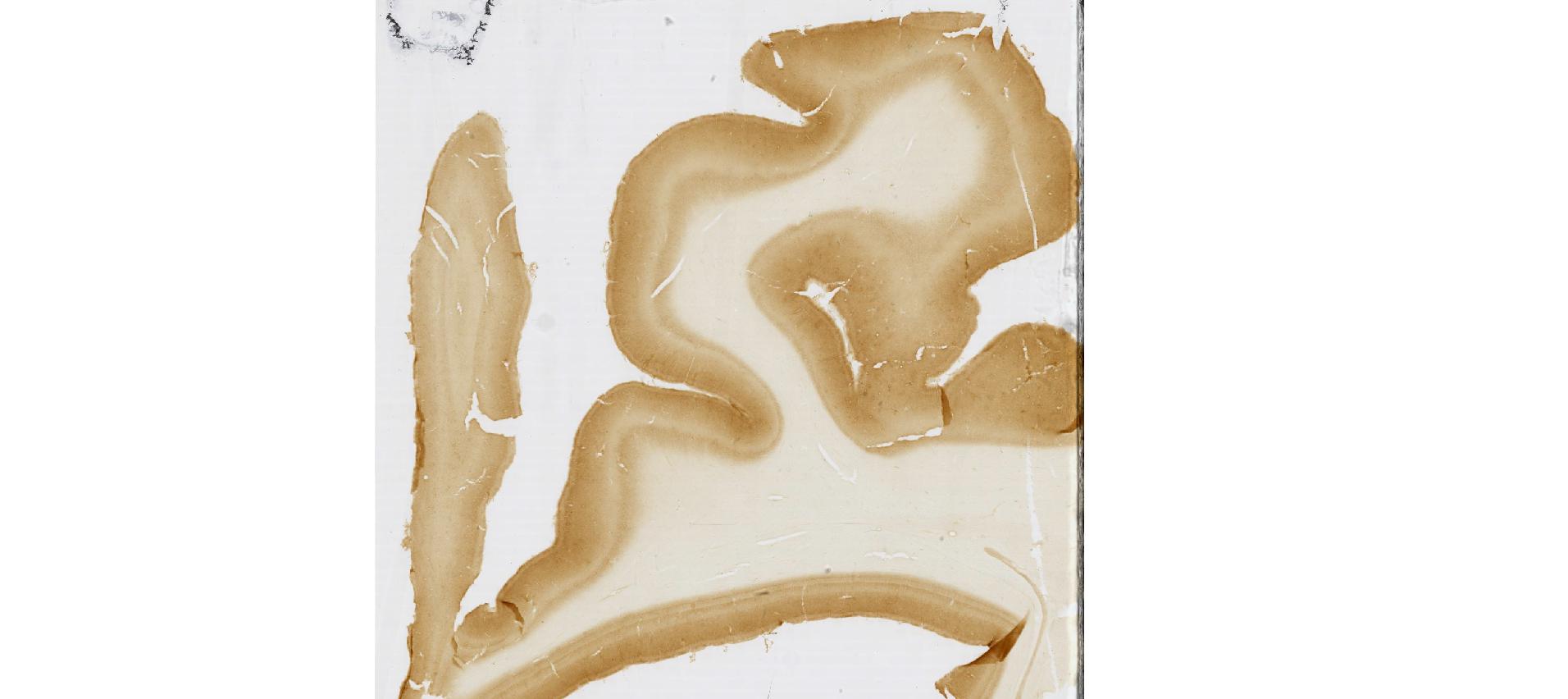

Supplement: Supplementary file 1 [file Presentation_1.ZIP › shank3-immunohistochemistry/case-14/6- visual cortex.jpg]

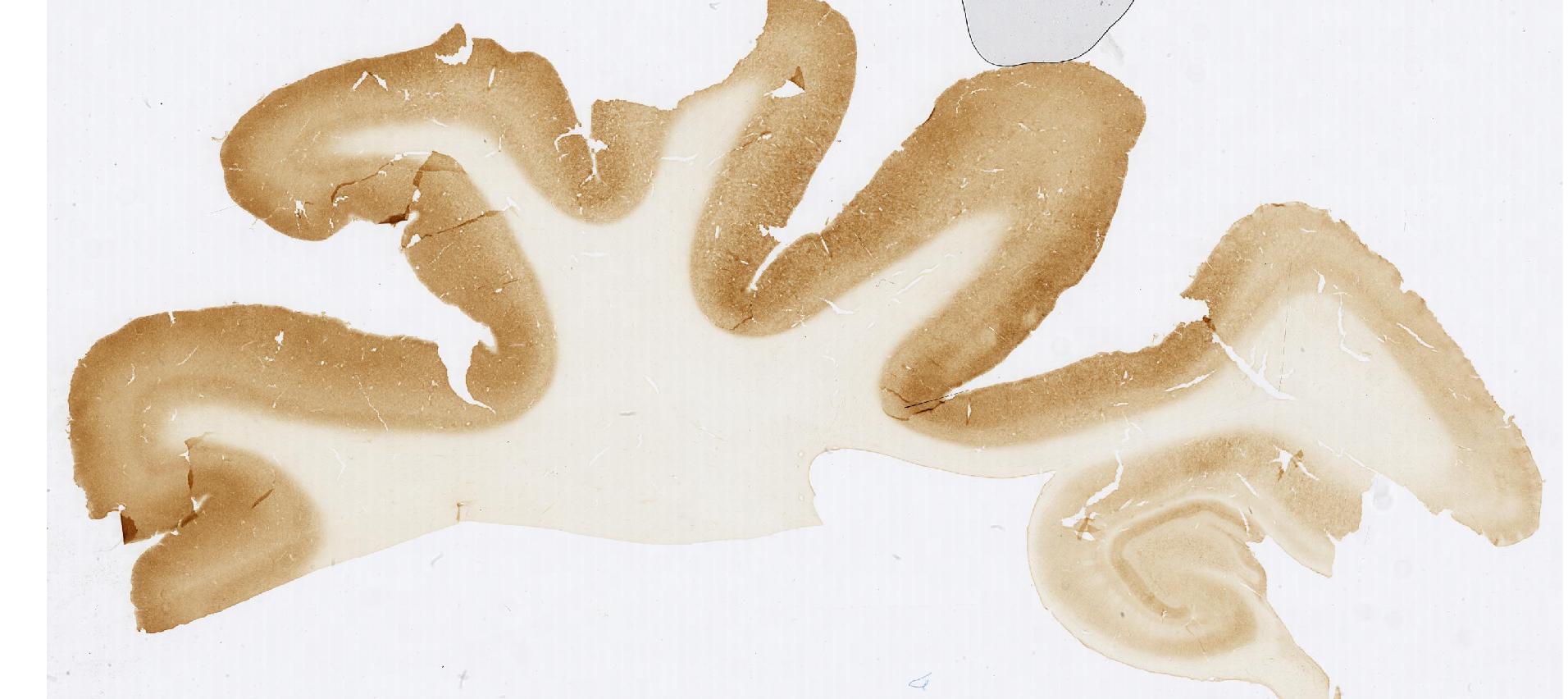

Supplement: Supplementary file 1 [file Presentation_1.ZIP › shank3-immunohistochemistry/case-14/7- hippocampal formation.jpg]

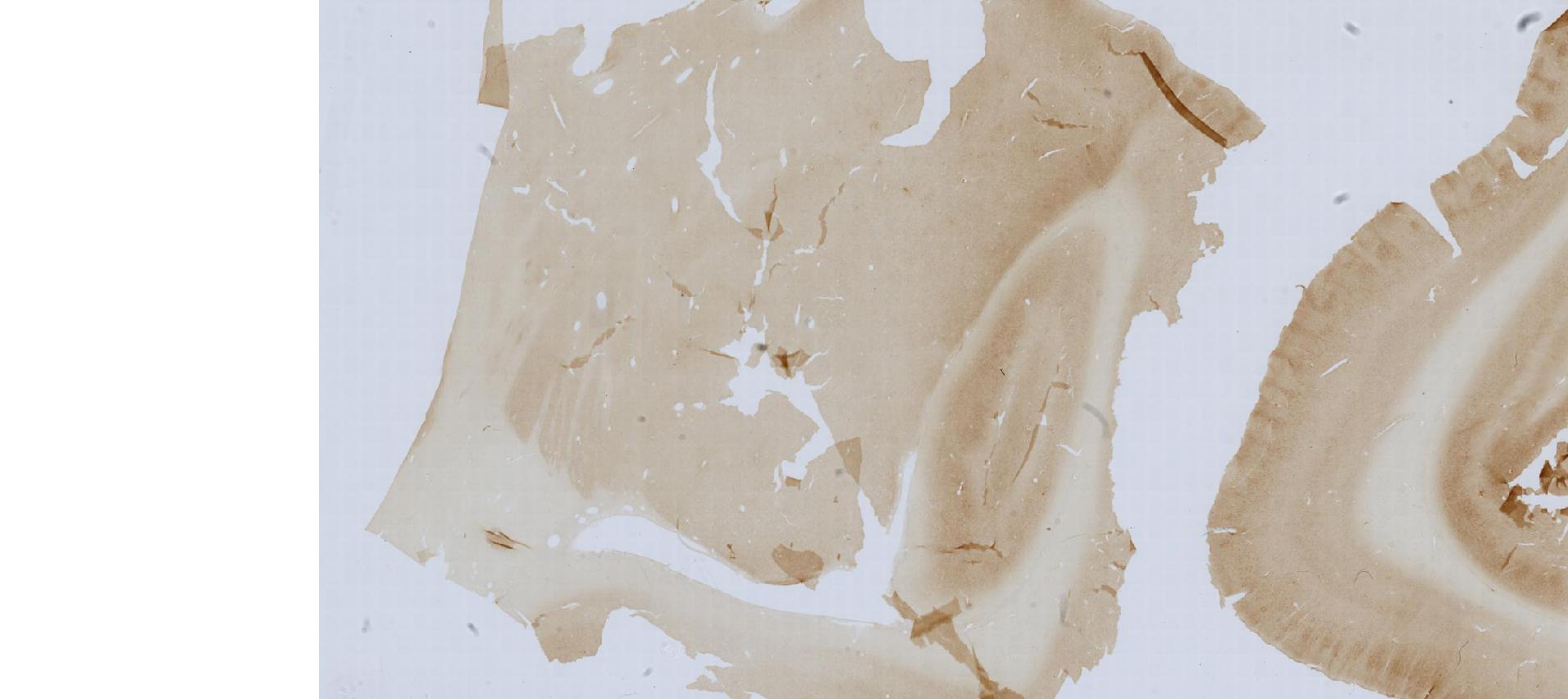

Supplement: Supplementary file 1 [file Presentation_1.ZIP › shank3-immunohistochemistry/case-14/8-amygdalar complex.jpg]

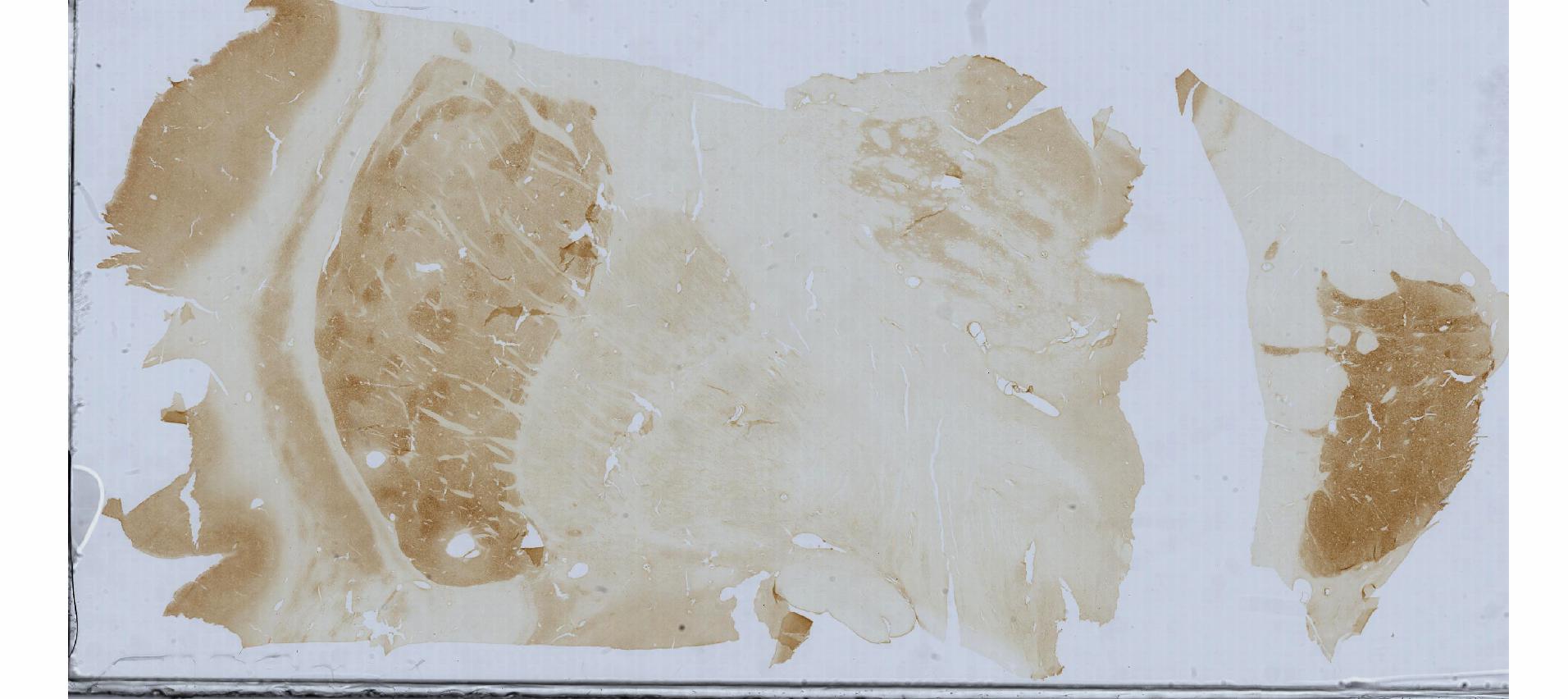

Supplement: Supplementary file 1 [file Presentation_1.ZIP › shank3-immunohistochemistry/case-14/9-basal ganglia and diencephalon.jpg]

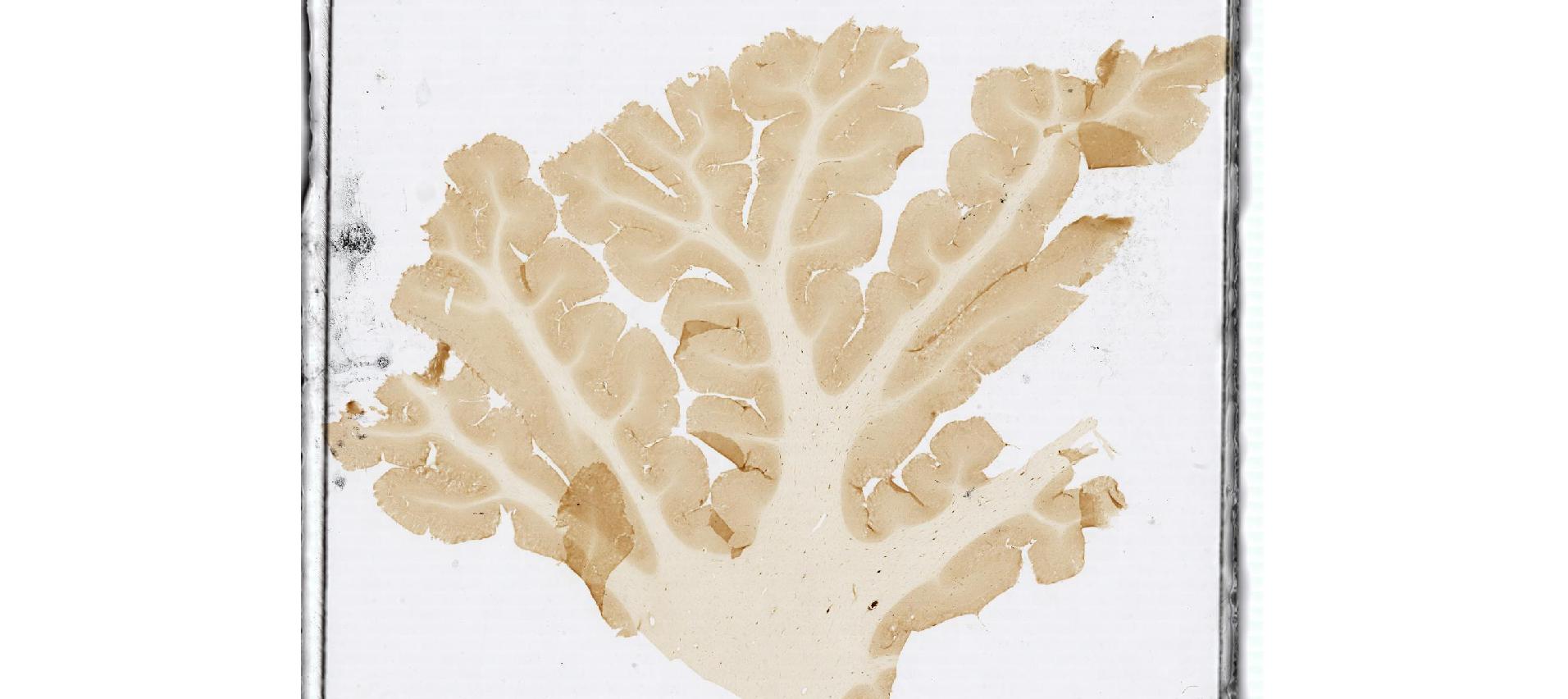

Supplement: Supplementary file 1 [file Presentation_1.ZIP › shank3-immunohistochemistry/case-15/cerebellum.jpg]

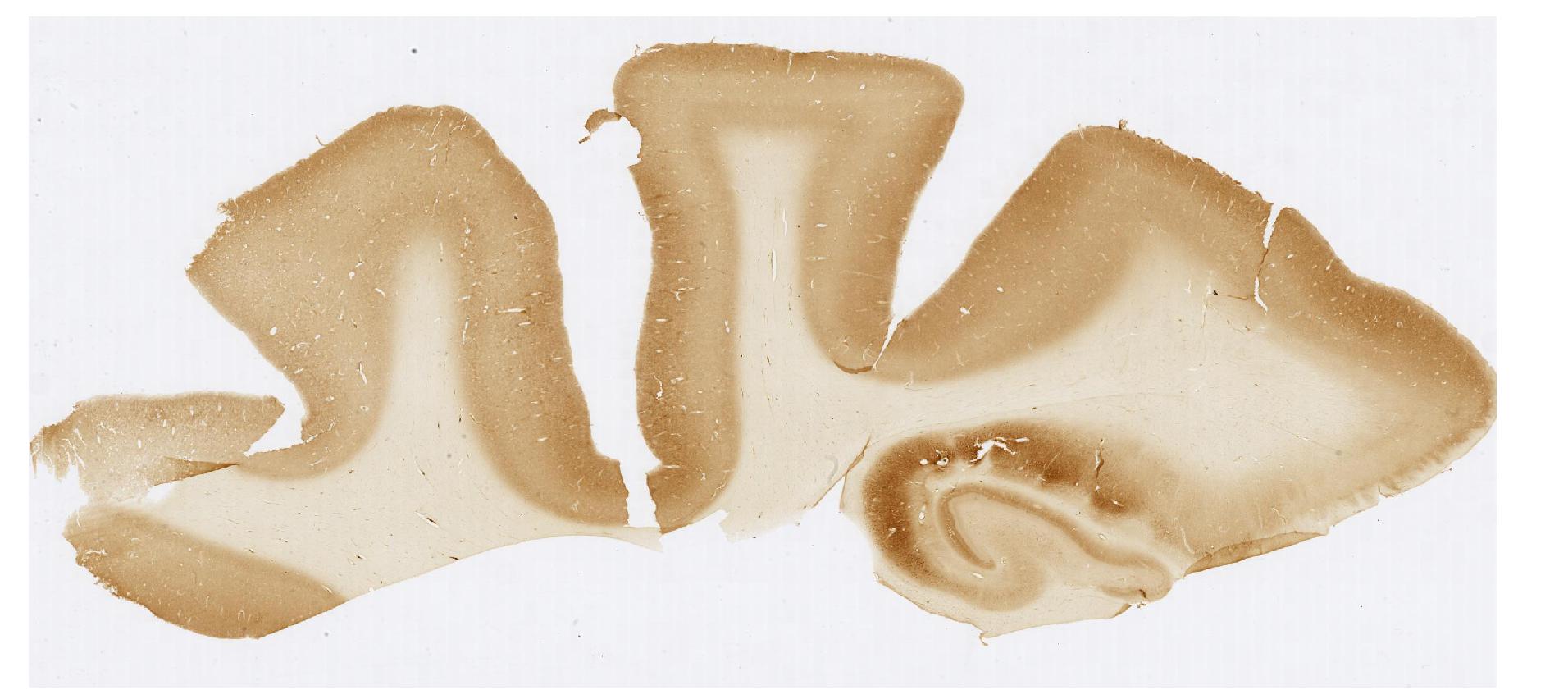

Supplement: Supplementary file 1 [file Presentation_1.ZIP › shank3-immunohistochemistry/case-15/hippocampal formation.jpg]

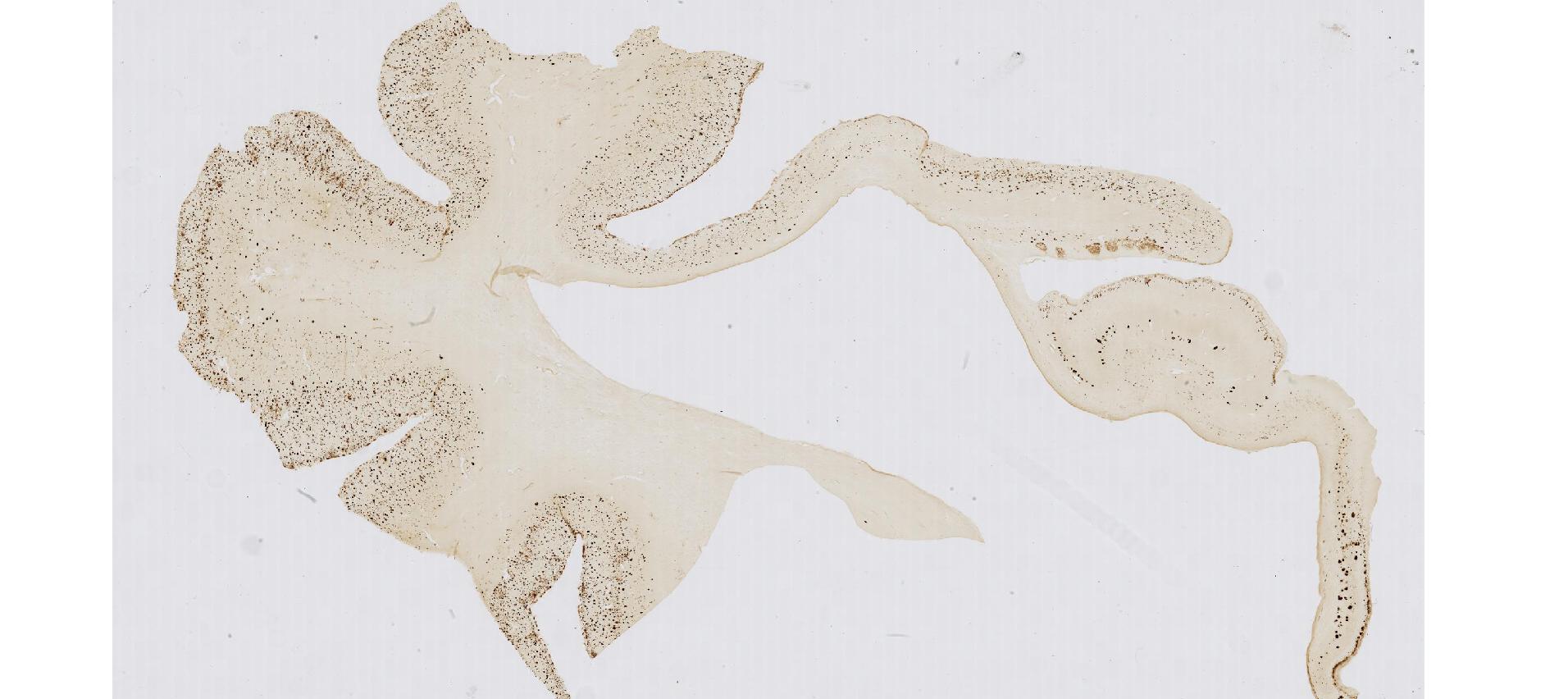

Supplement: Supplementary file 1 [file Presentation_1.ZIP › shank3-immunohistochemistry/case-16/HP-6E10.jpg]

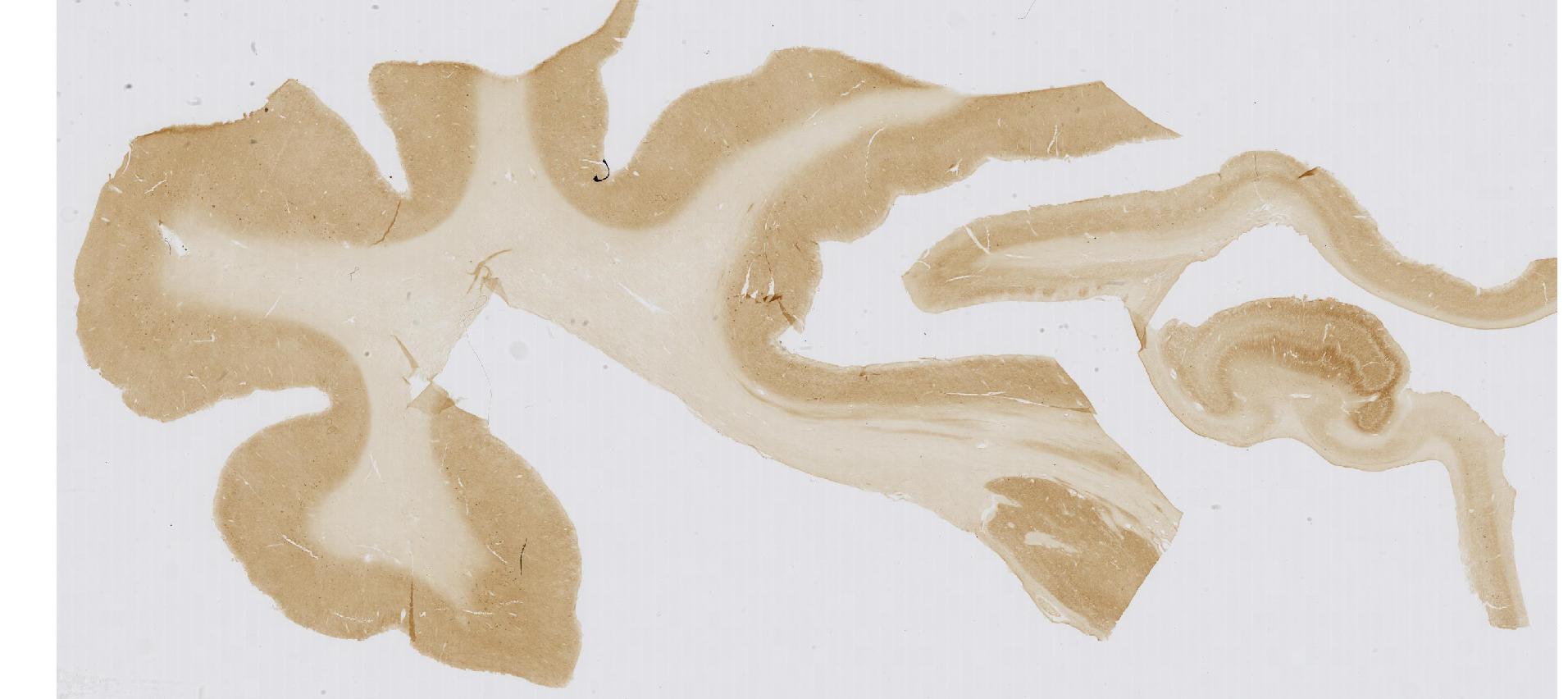

Supplement: Supplementary file 1 [file Presentation_1.ZIP › shank3-immunohistochemistry/case-16/HP-bace1.jpg]

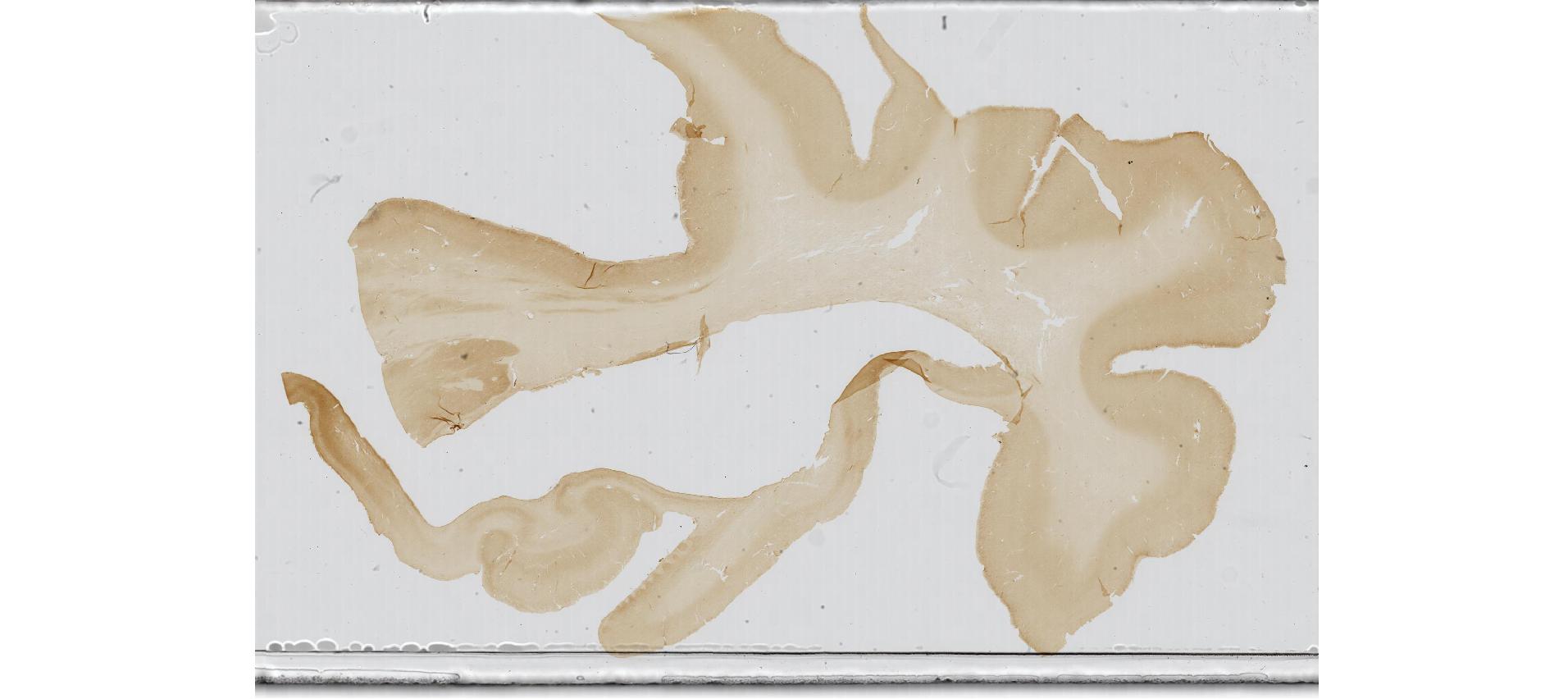

Supplement: Supplementary file 1 [file Presentation_1.ZIP › shank3-immunohistochemistry/case-16/HP-shank3.jpg]

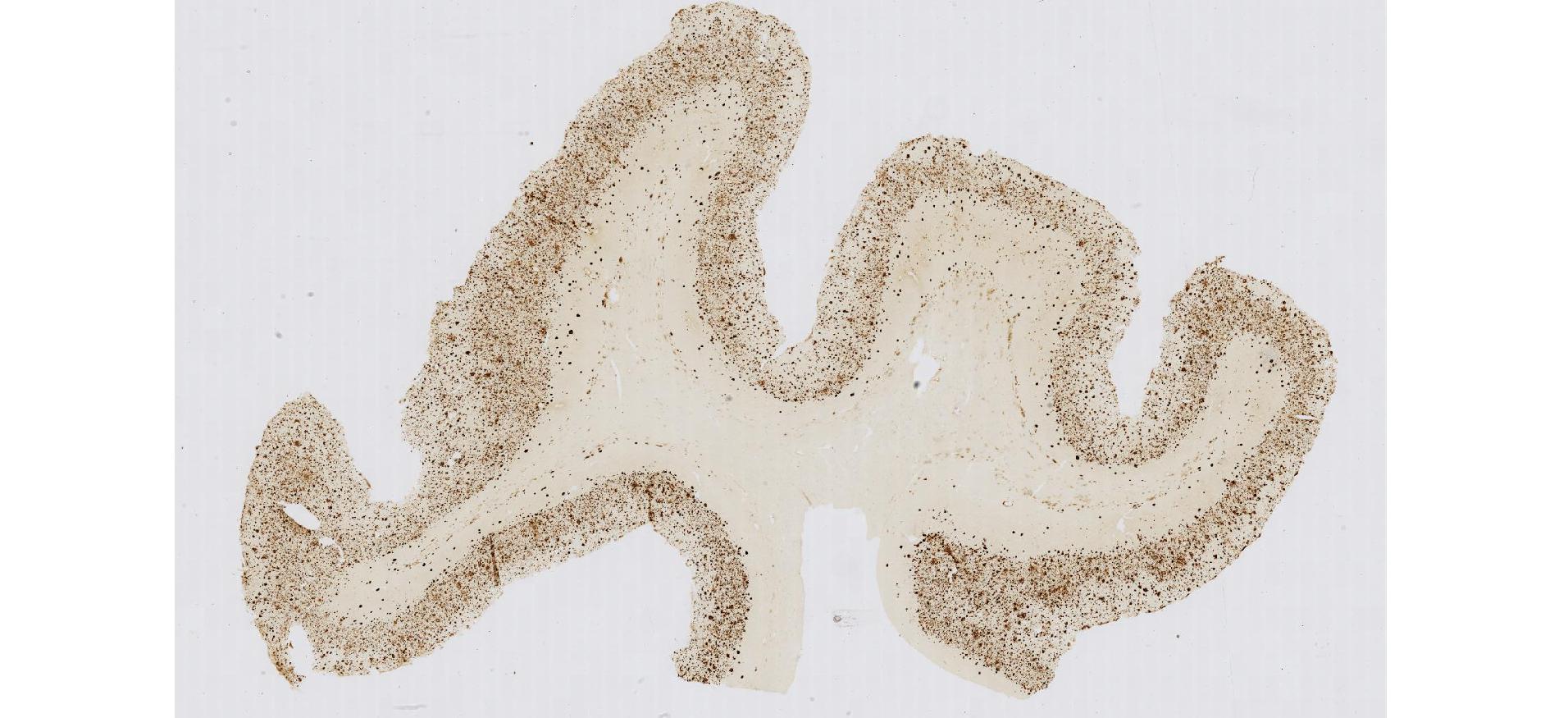

Supplement: Supplementary file 1 [file Presentation_1.ZIP › shank3-immunohistochemistry/case-16/PFC-6E10.jpg]

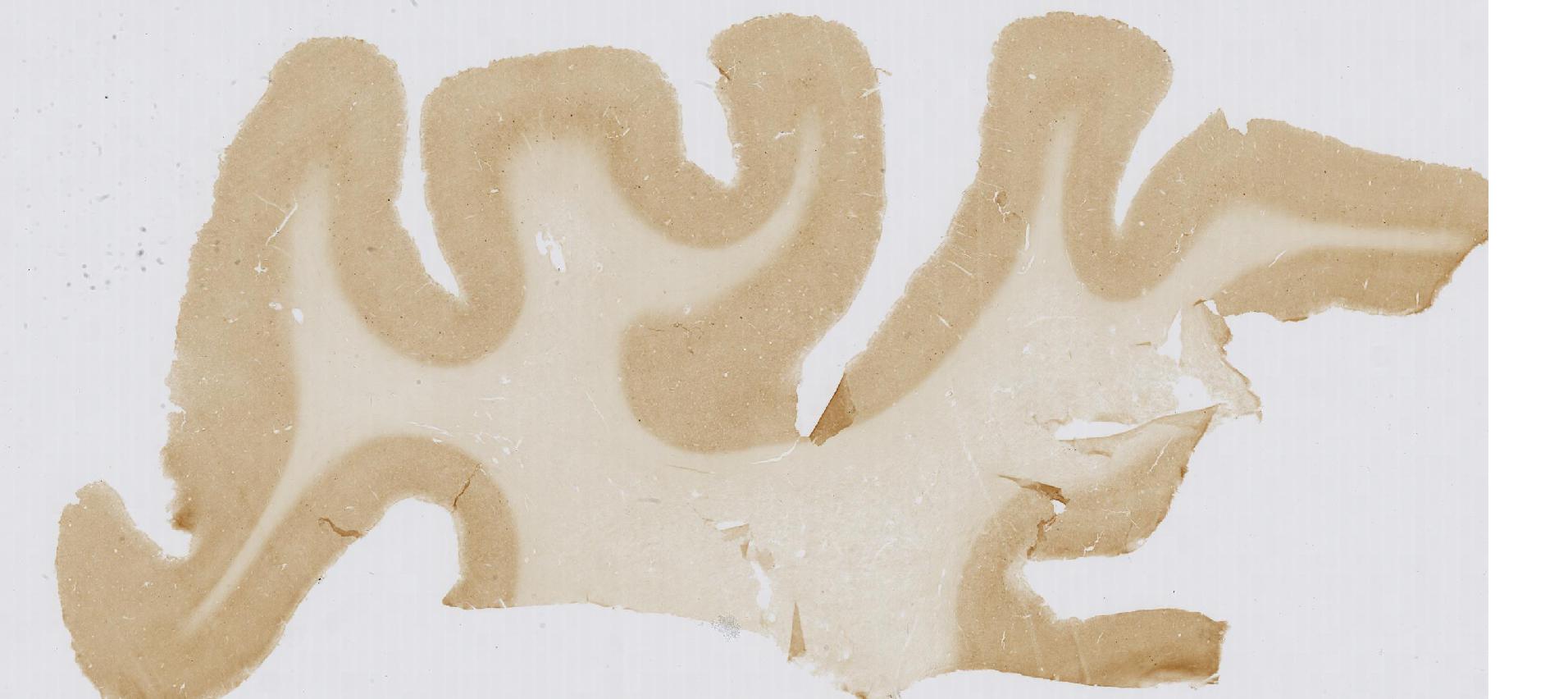

Supplement: Supplementary file 1 [file Presentation_1.ZIP › shank3-immunohistochemistry/case-16/PFC-bace1.jpg]

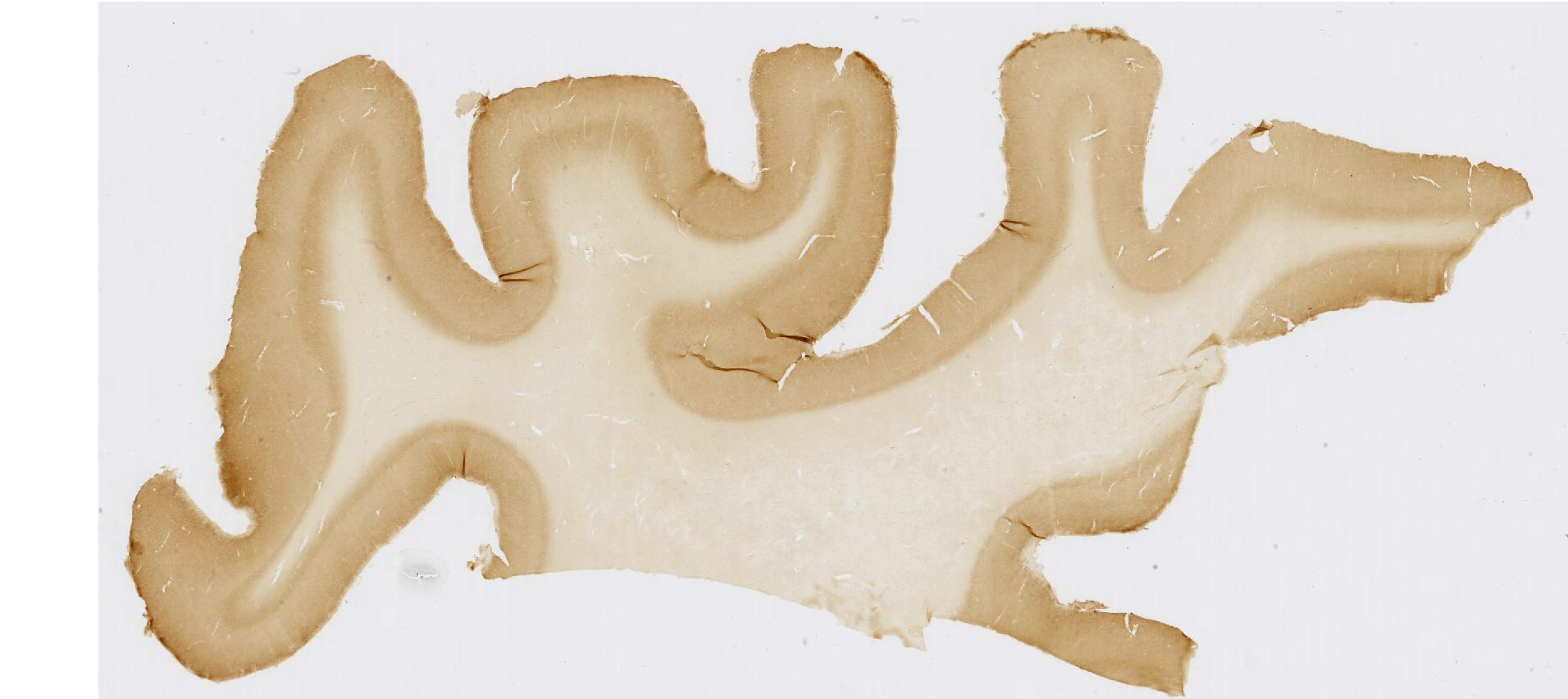

Supplement: Supplementary file 1 [file Presentation_1.ZIP › shank3-immunohistochemistry/case-16/PFC-shank3.jpg]

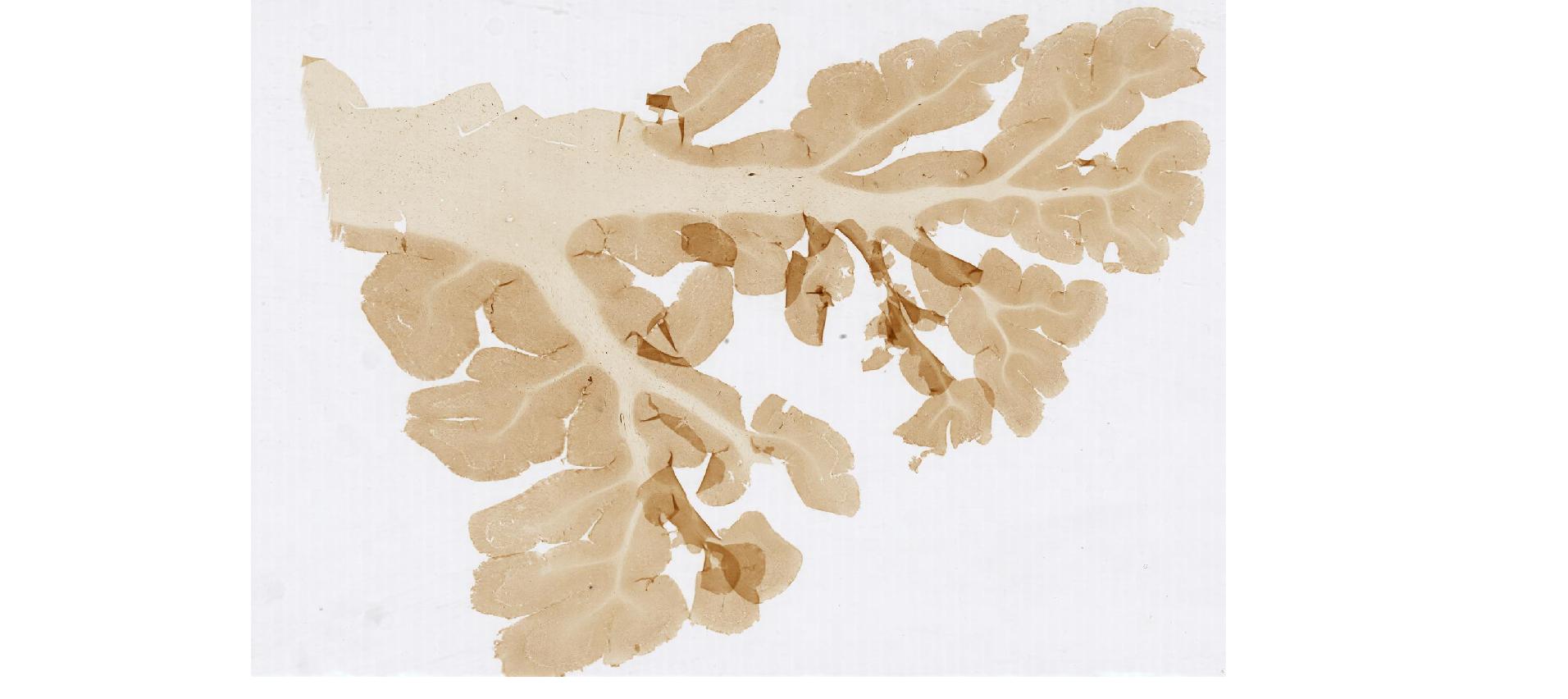

Supplement: Supplementary file 1 [file Presentation_1.ZIP › shank3-immunohistochemistry/case-16/cerebellum-shank3.jpg]

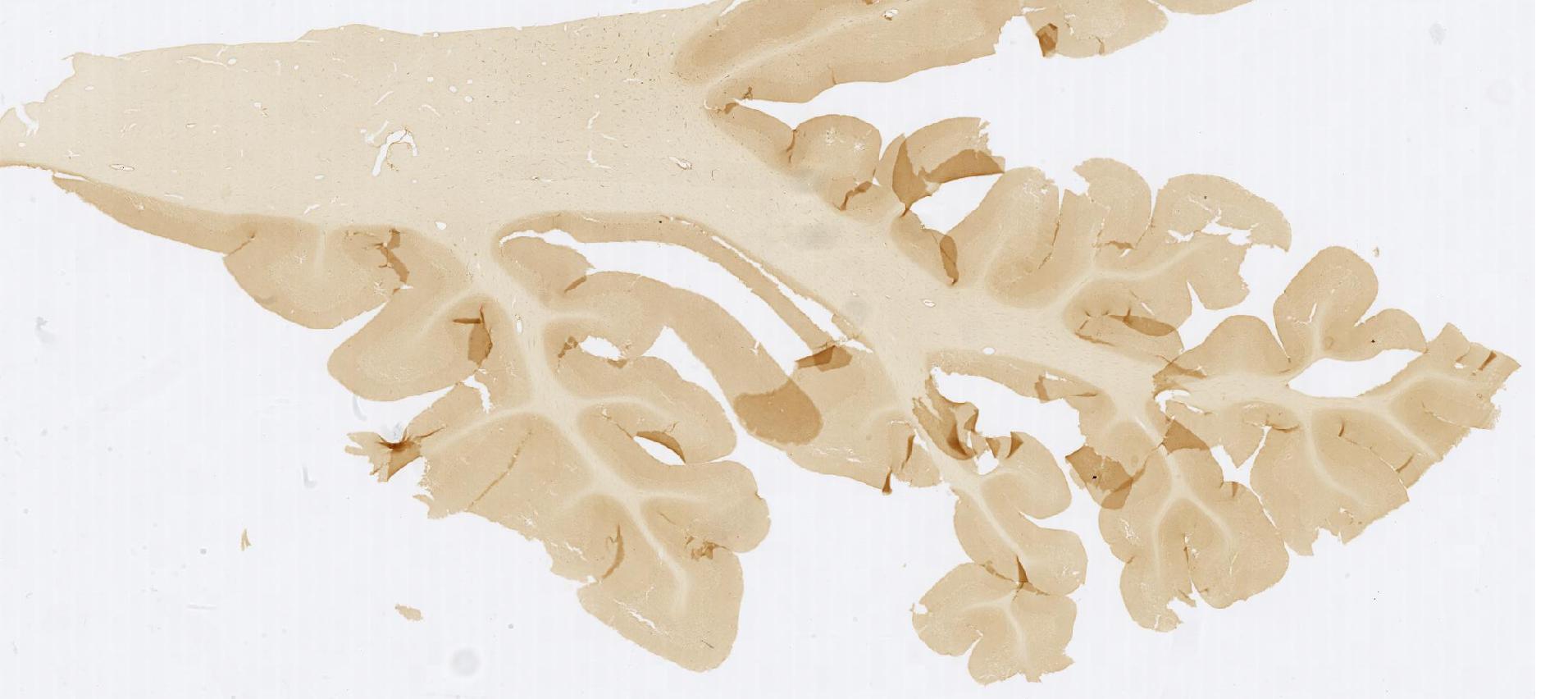

Supplement: Supplementary file 1 [file Presentation_1.ZIP › shank3-immunohistochemistry/case-17/cerebellum.jpg]

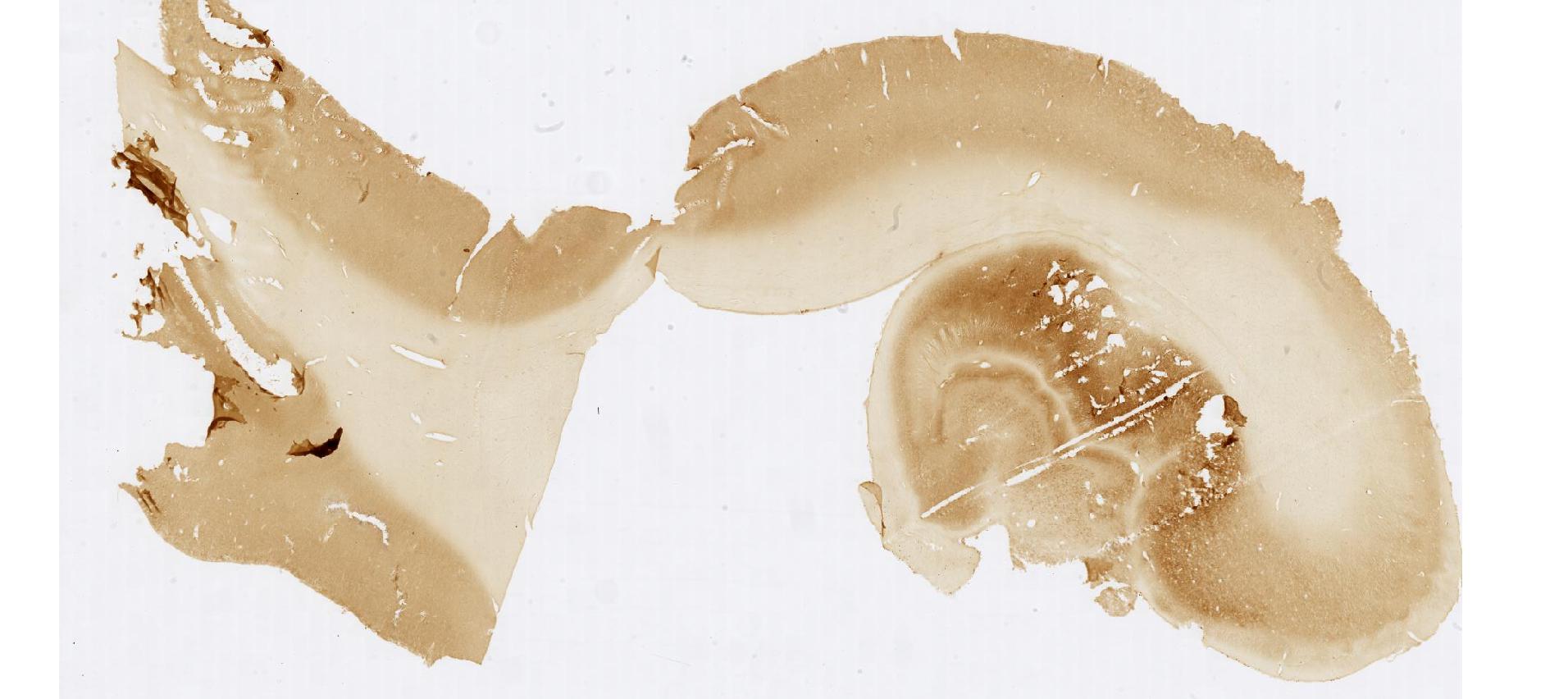

Supplement: Supplementary file 1 [file Presentation_1.ZIP › shank3-immunohistochemistry/case-17/hippocampal formation.jpg]

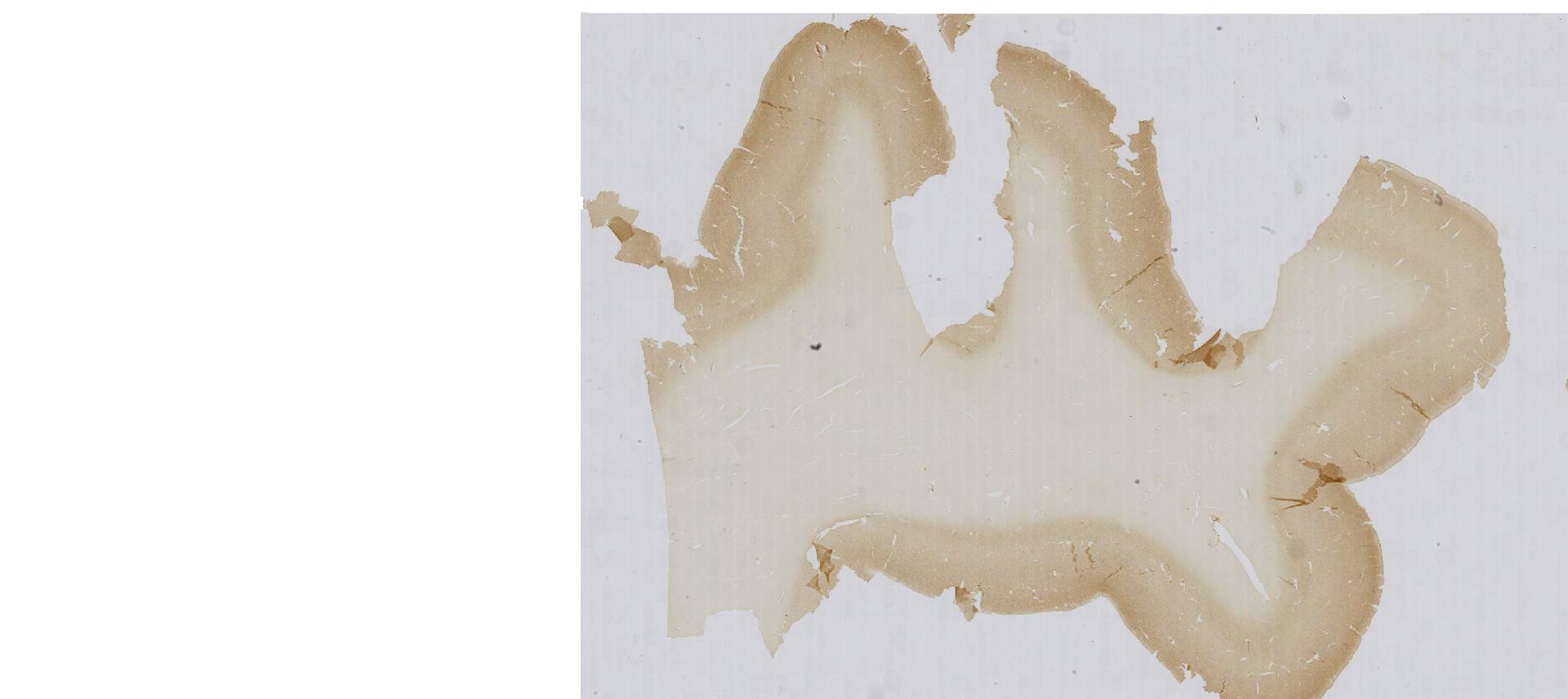

Supplement: Supplementary file 1 [file Presentation_1.ZIP › shank3-immunohistochemistry/case-17/prefrontal cortex.jpg]

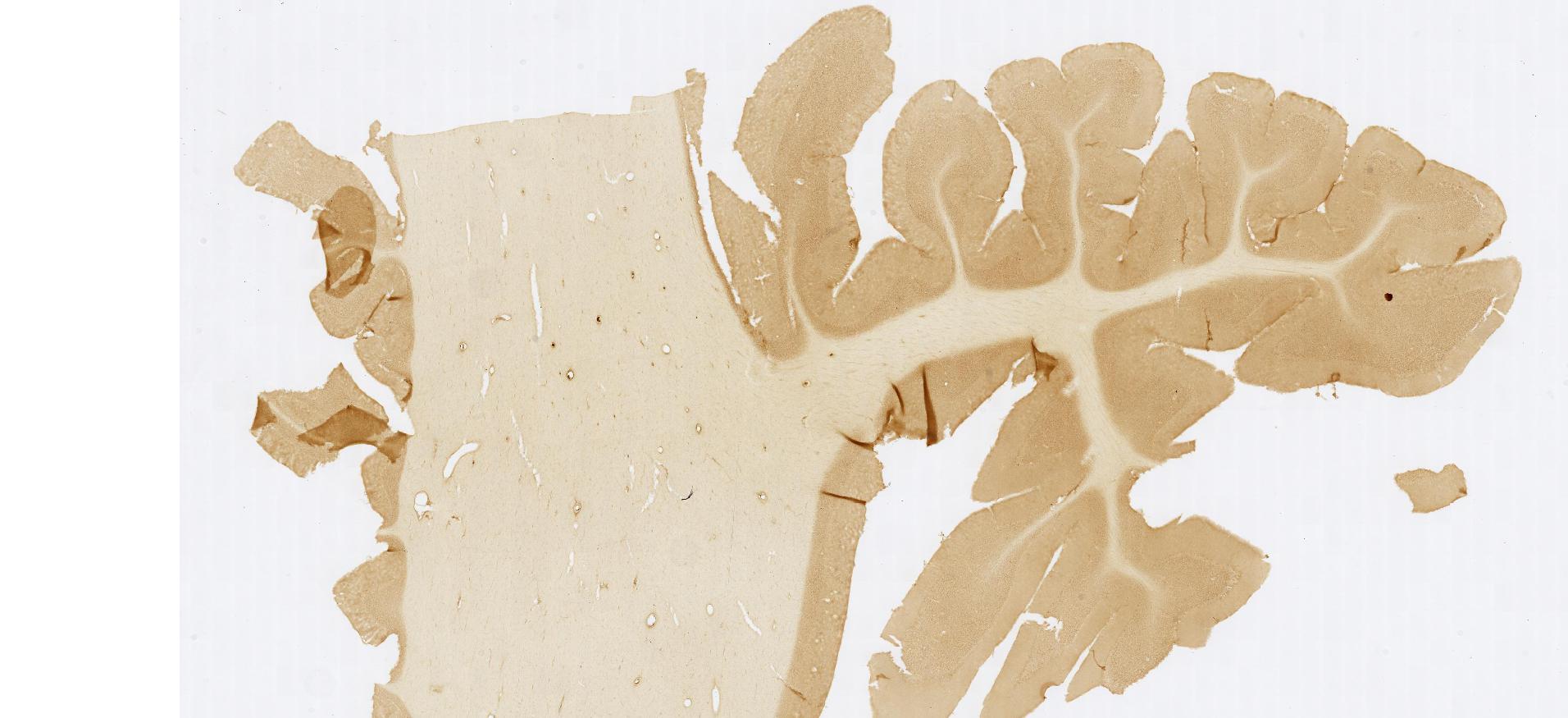

Supplement: Supplementary file 1 [file Presentation_1.ZIP › shank3-immunohistochemistry/case-18/cerebellum.jpg]

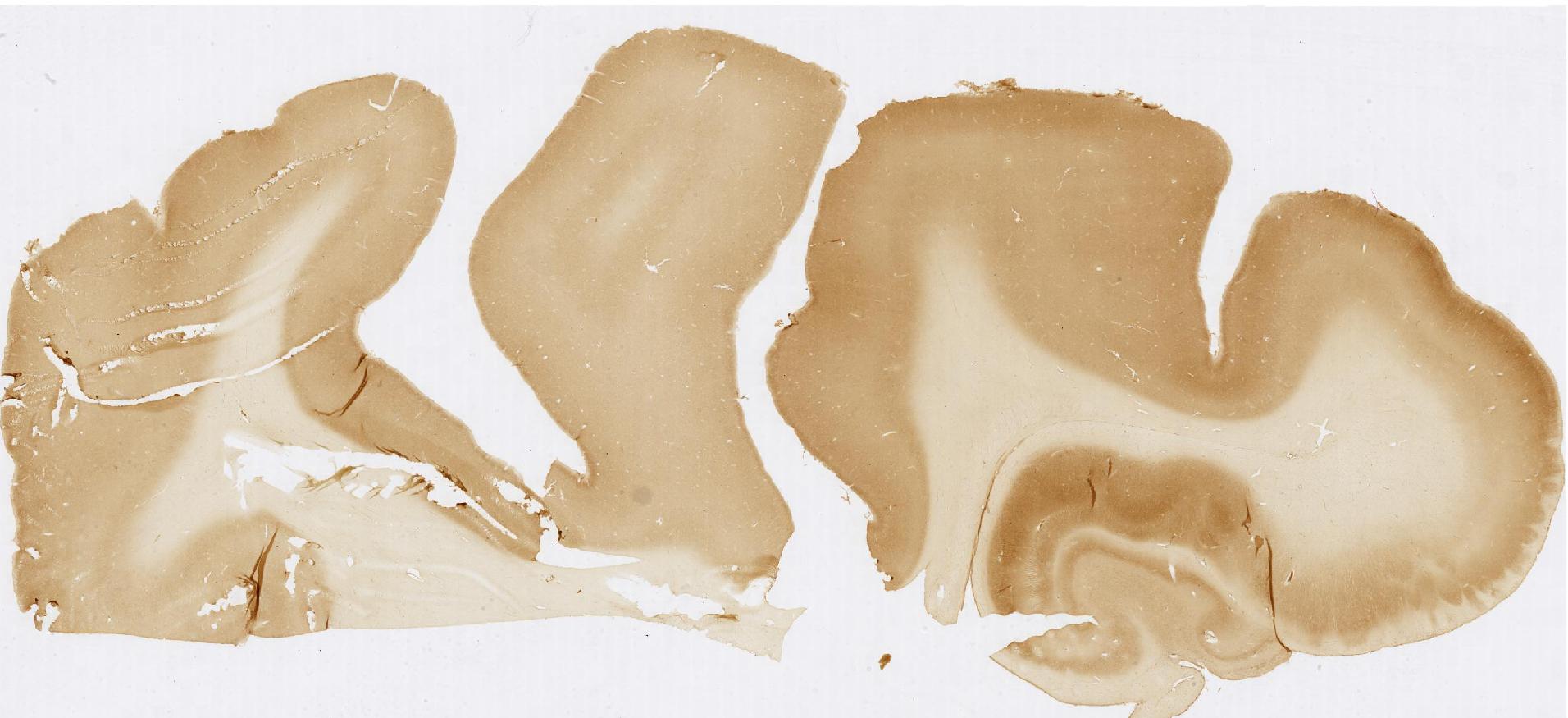

Supplement: Supplementary file 1 [file Presentation_1.ZIP › shank3-immunohistochemistry/case-18/hippocampal formation.jpg]

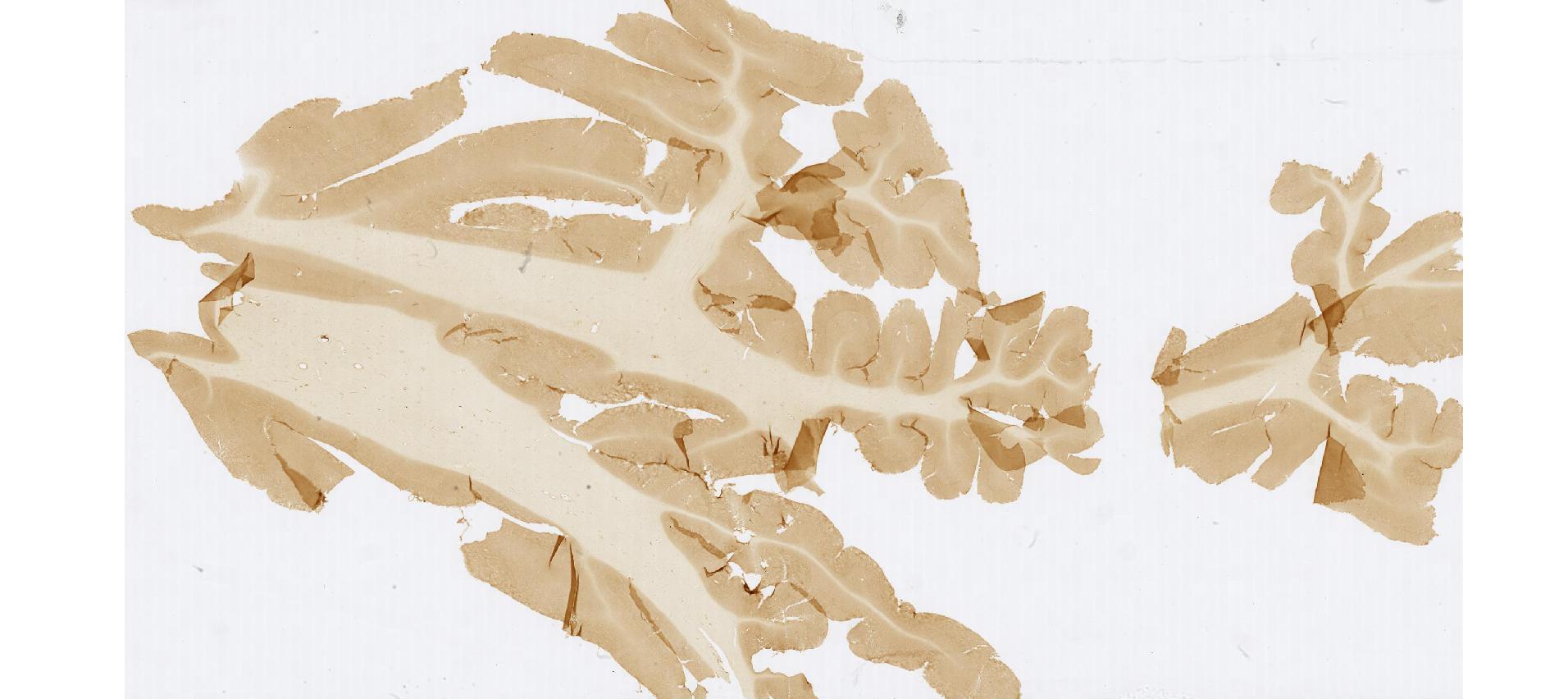

Supplement: Supplementary file 1 [file Presentation_1.ZIP › shank3-immunohistochemistry/case-19/cerebellum.jpg]

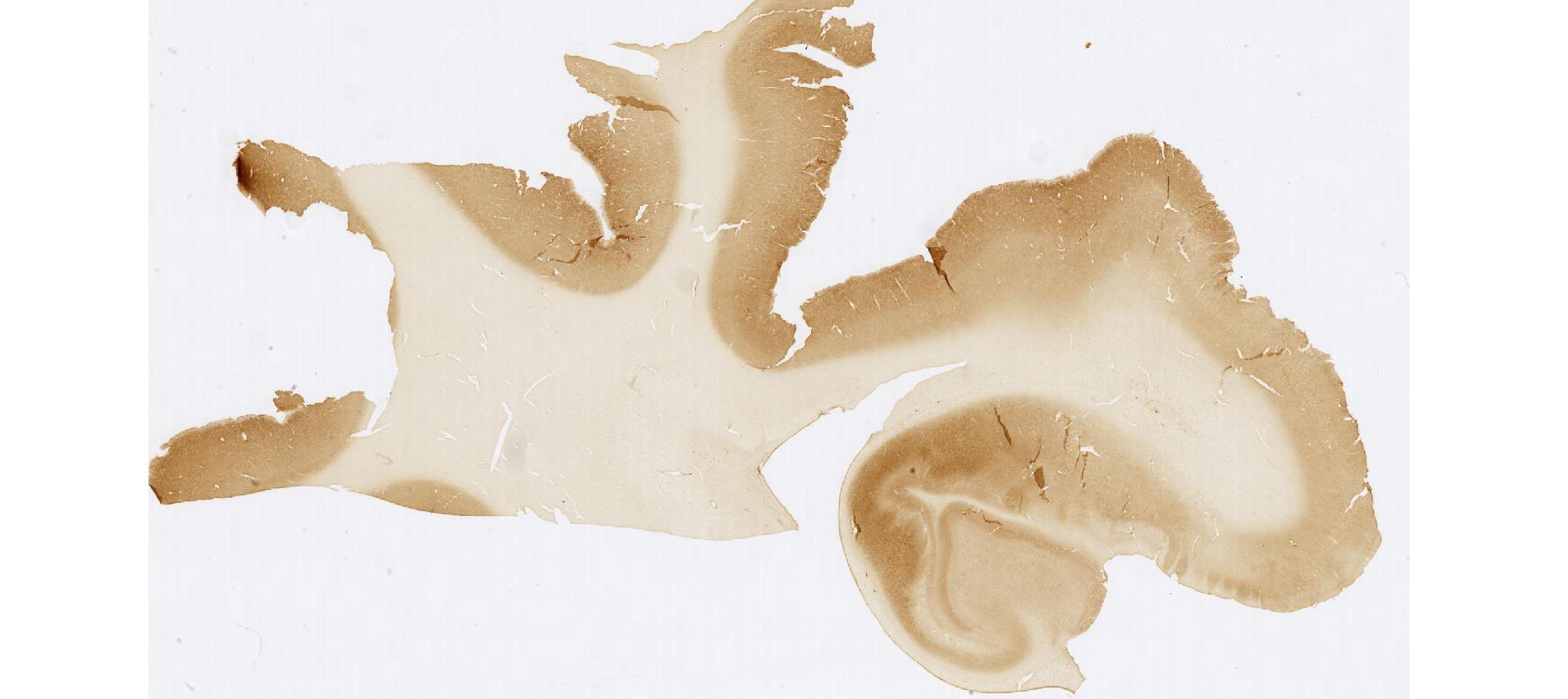

Supplement: Supplementary file 1 [file Presentation_1.ZIP › shank3-immunohistochemistry/case-19/hippocampal formation.jpg]

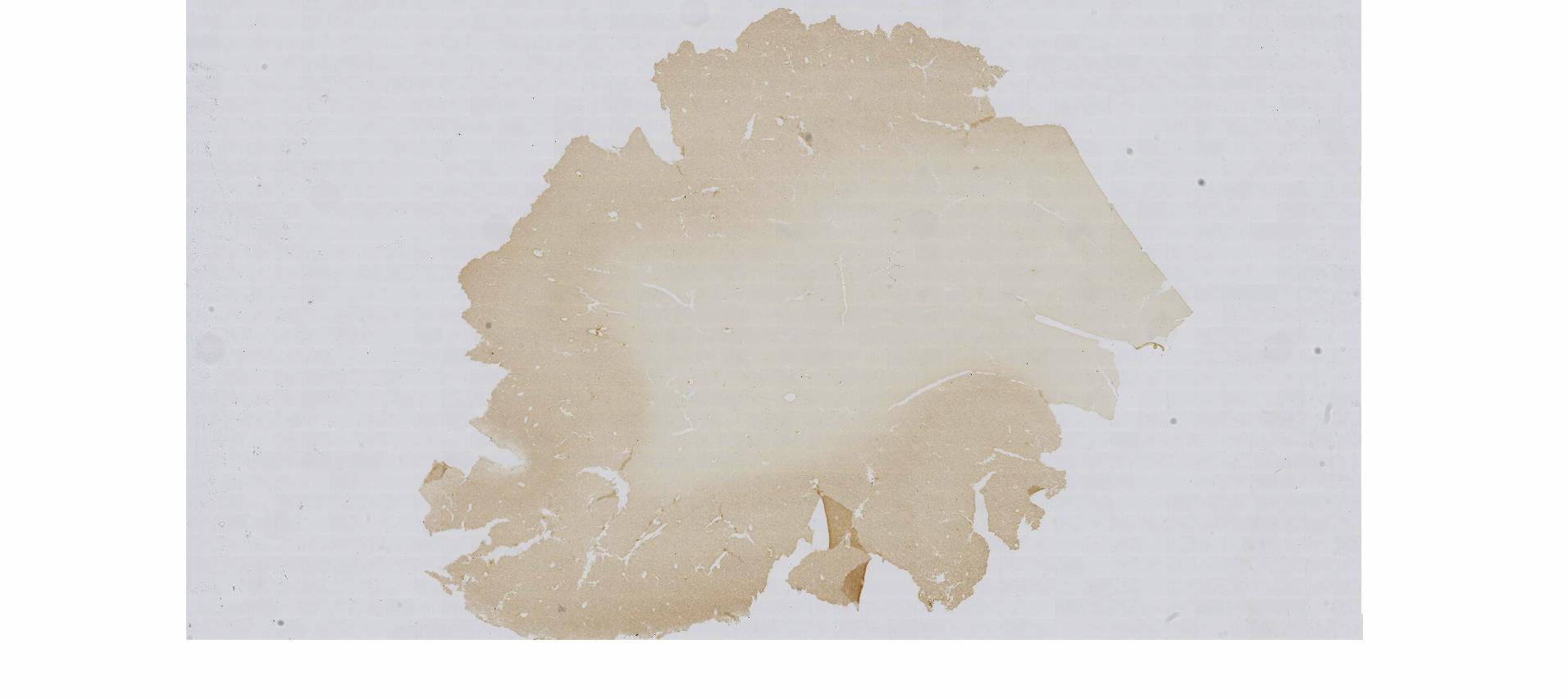

Supplement: Supplementary file 1 [file Presentation_1.ZIP › shank3-immunohistochemistry/case-19/prefrontal cortex.jpg]

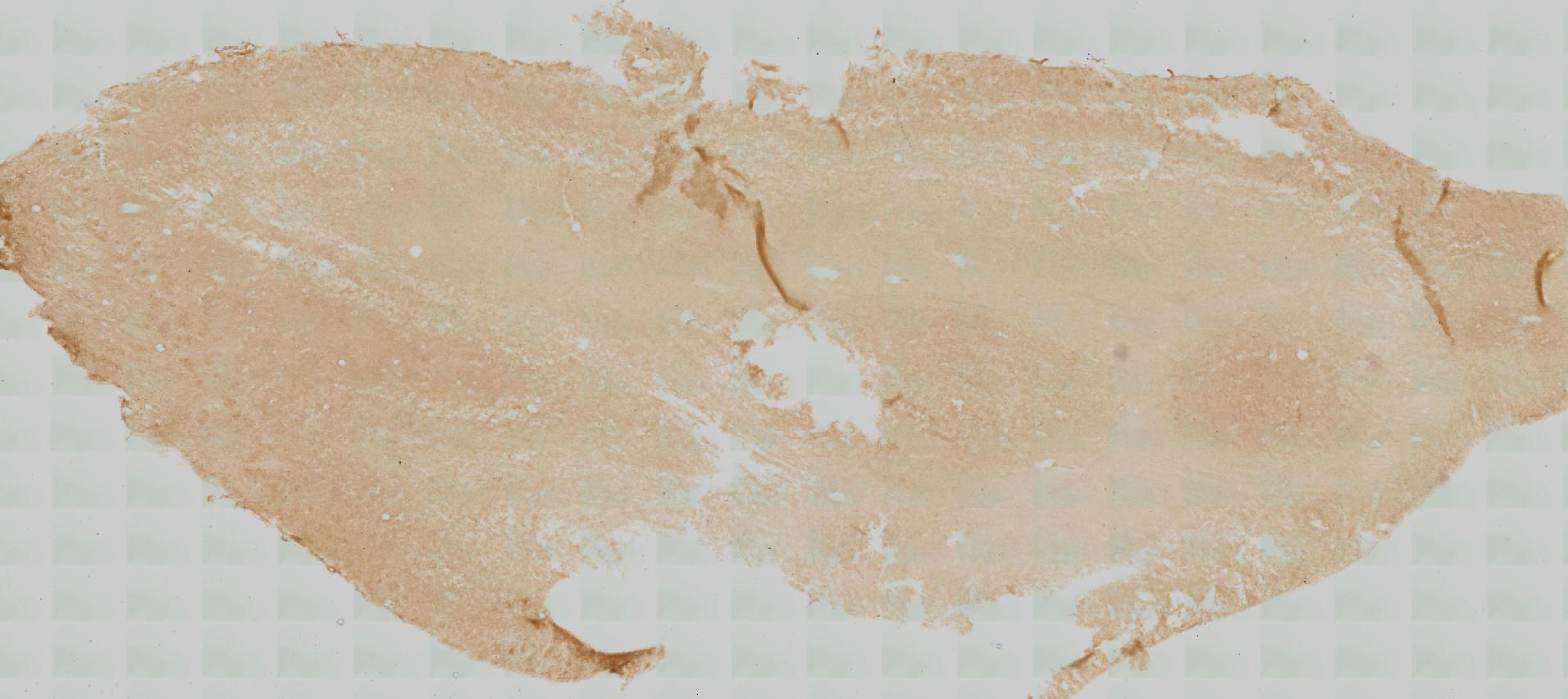

Supplement: Supplementary file 1 [file Presentation_1.ZIP › shank3-immunohistochemistry/case-2/1-olfactory bulb.jpg]

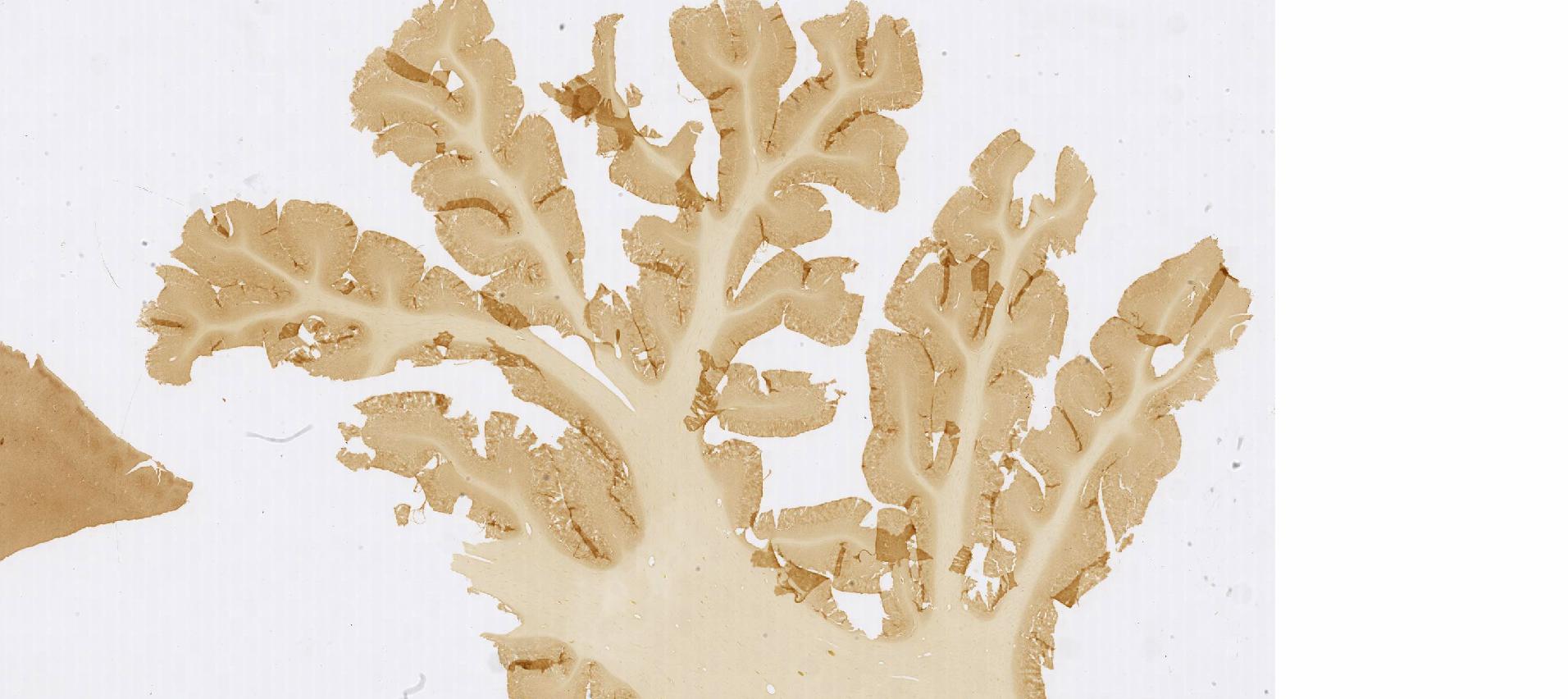

Supplement: Supplementary file 1 [file Presentation_1.ZIP › shank3-immunohistochemistry/case-2/10-cerebellum.jpg]

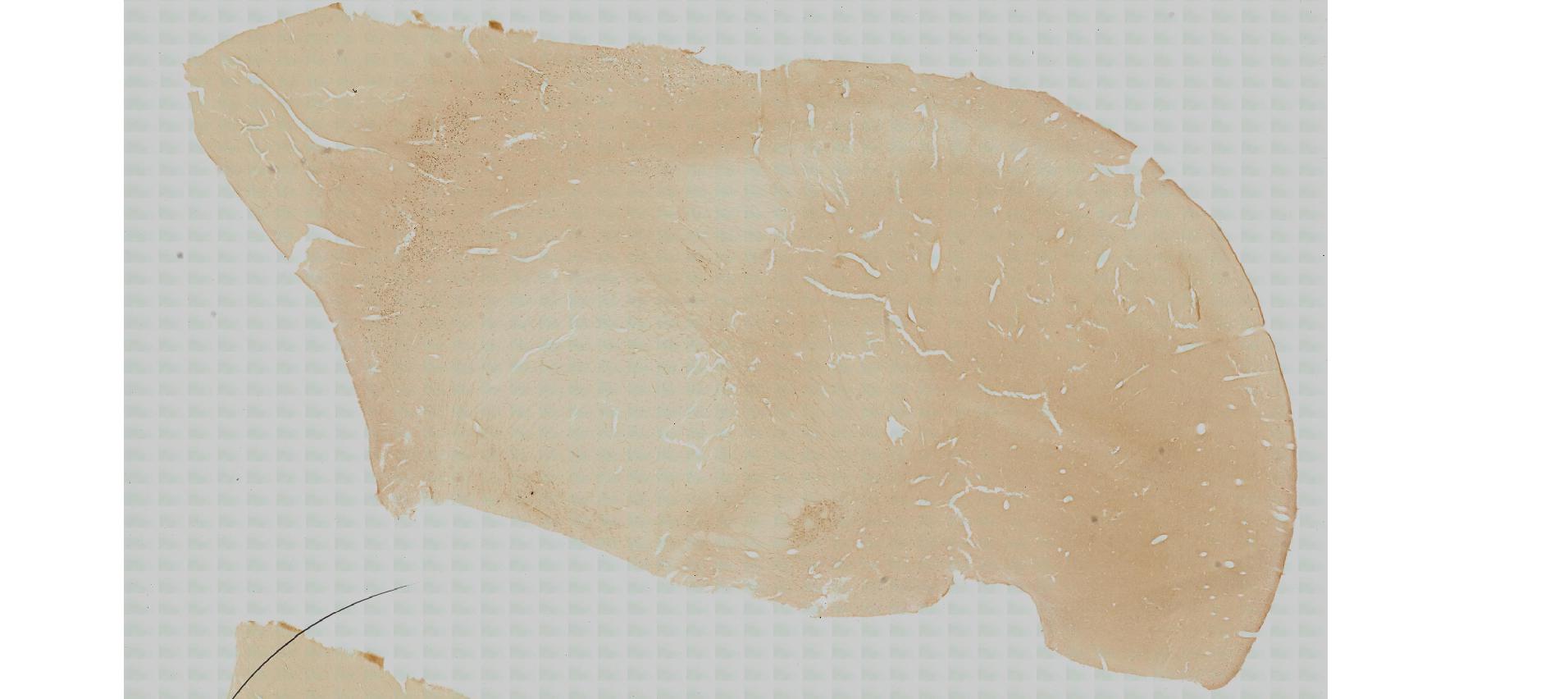

Supplement: Supplementary file 1 [file Presentation_1.ZIP › shank3-immunohistochemistry/case-2/11-midbrain.jpg]

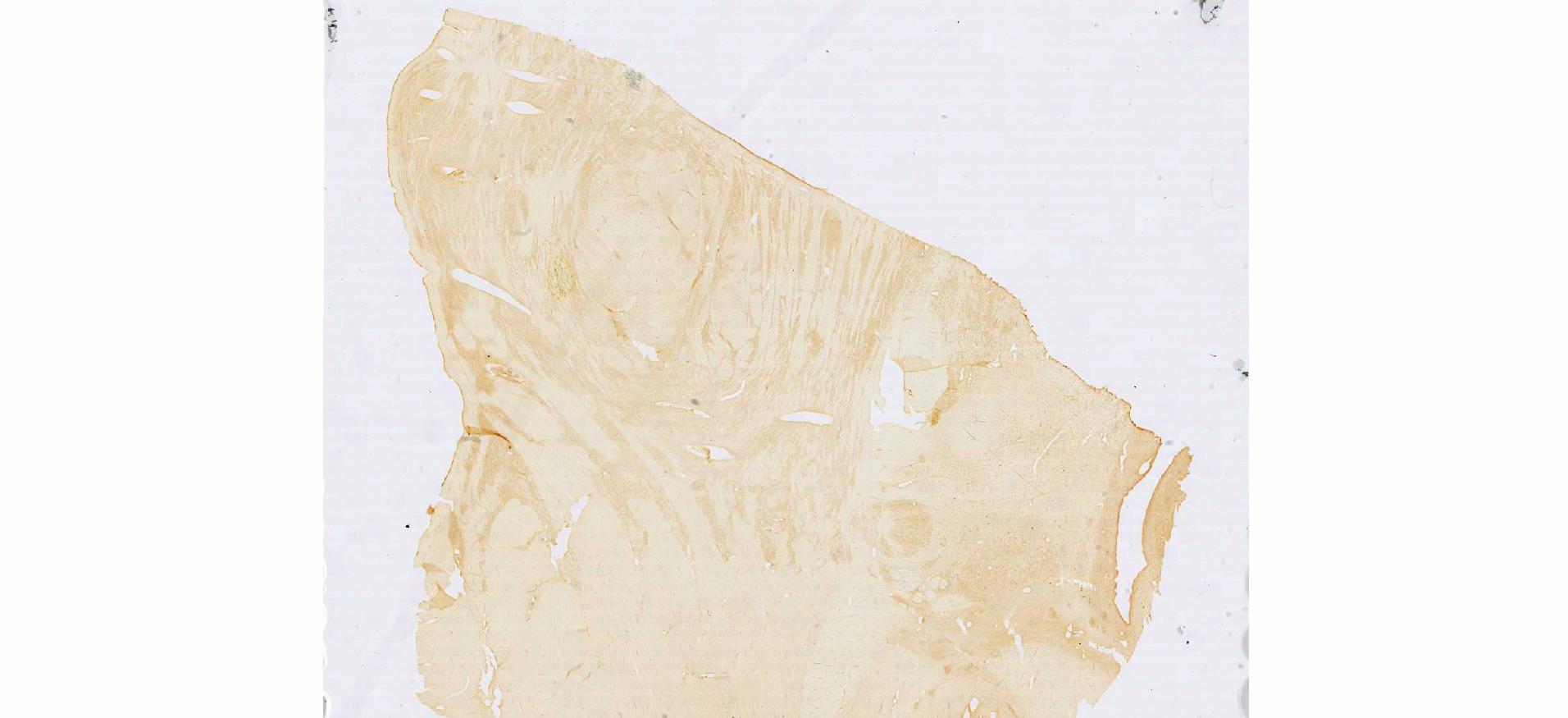

Supplement: Supplementary file 1 [file Presentation_1.ZIP › shank3-immunohistochemistry/case-2/12-pons.jpg]

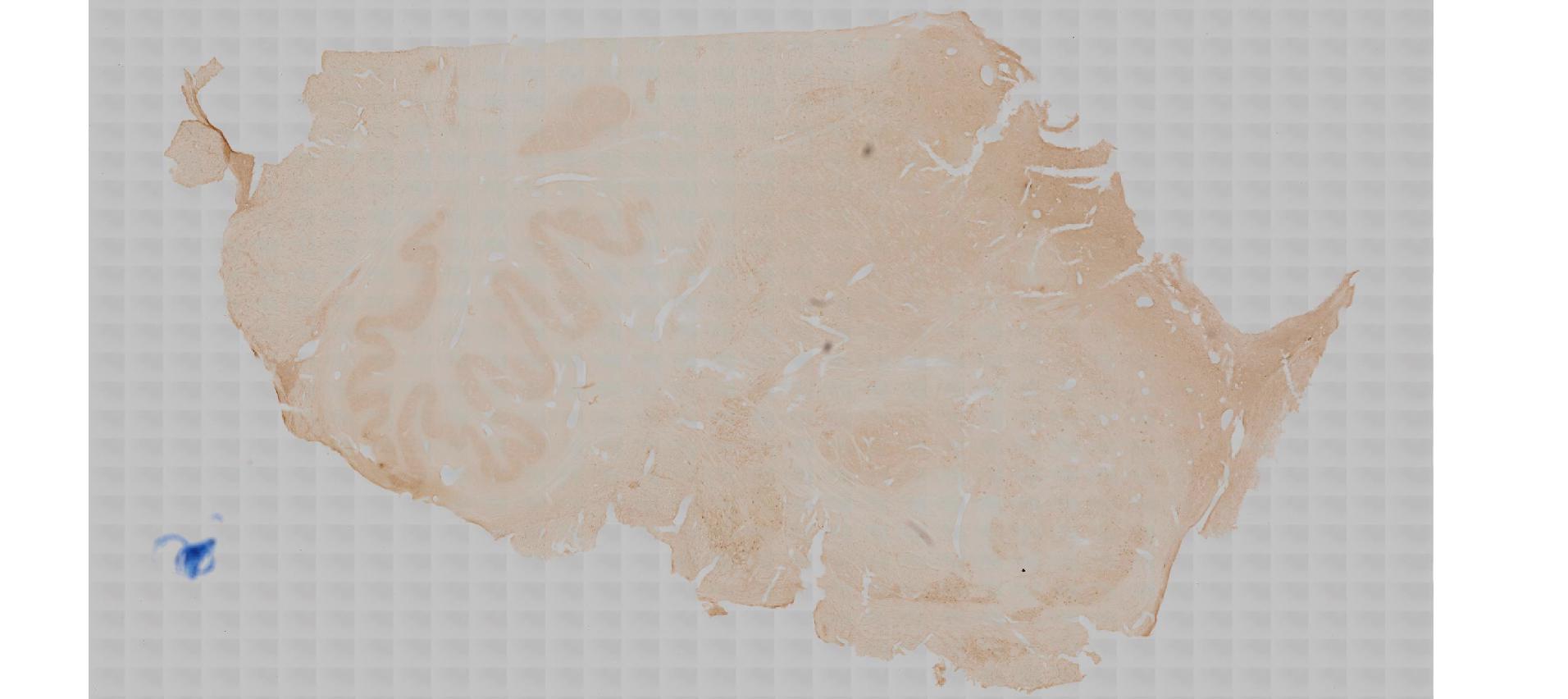

Supplement: Supplementary file 1 [file Presentation_1.ZIP › shank3-immunohistochemistry/case-2/13-medulla oblongata.jpg]

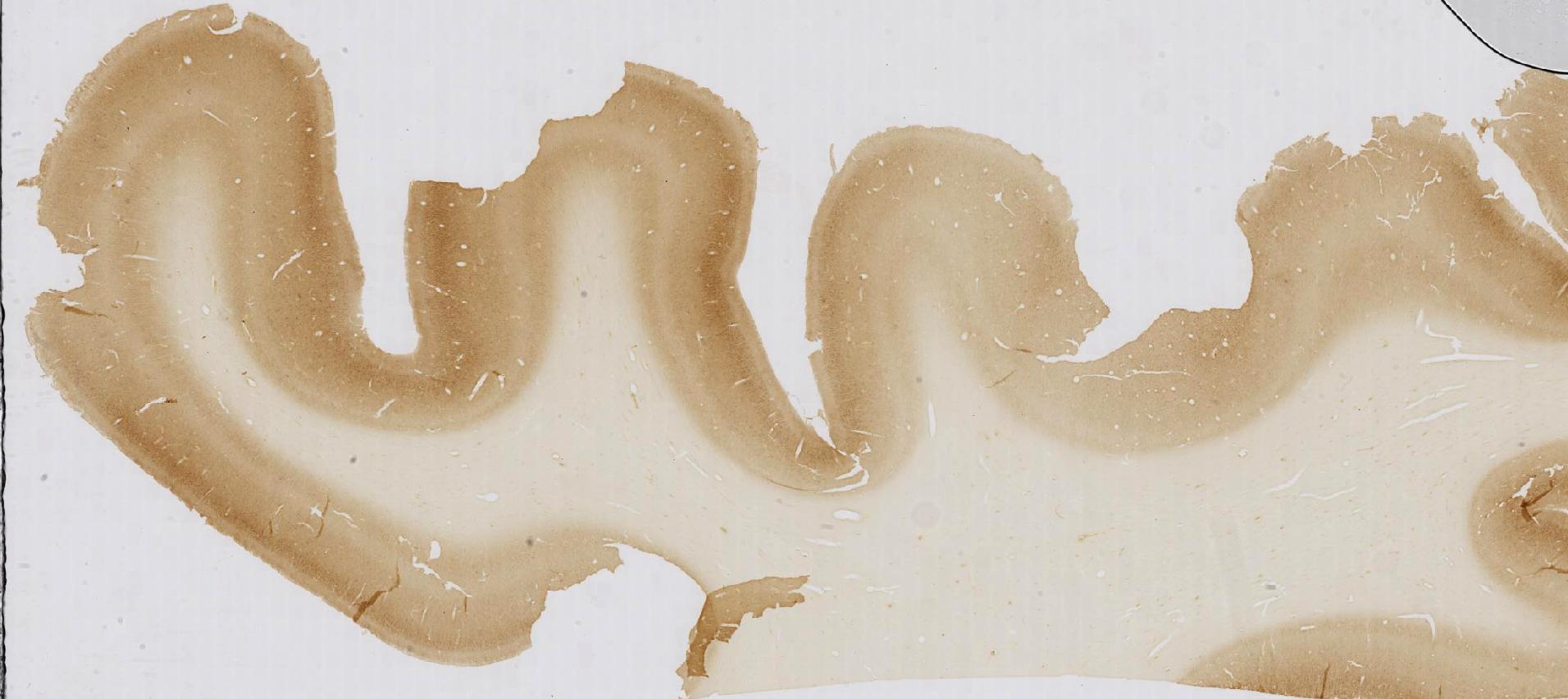

Supplement: Supplementary file 1 [file Presentation_1.ZIP › shank3-immunohistochemistry/case-2/2-prefrontal cortex.jpg]

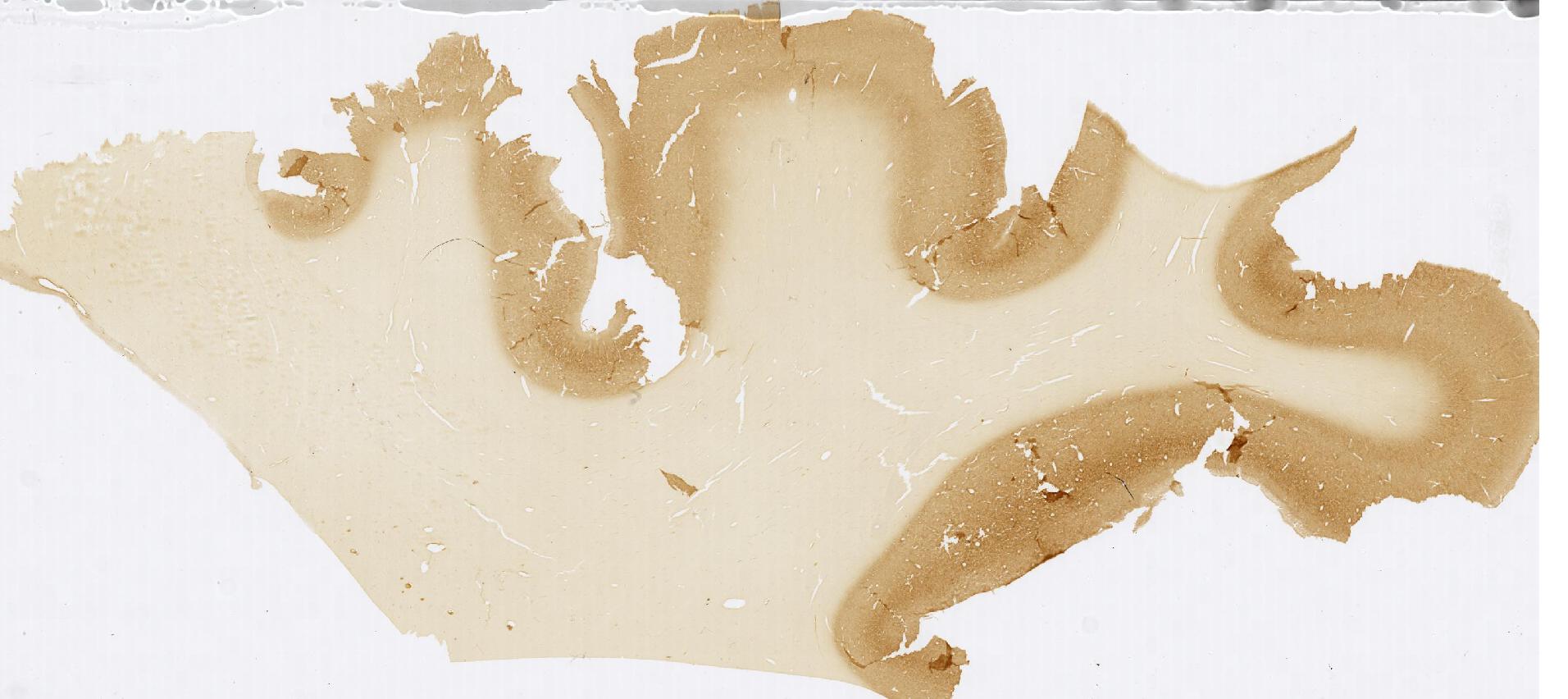

Supplement: Supplementary file 1 [file Presentation_1.ZIP › shank3-immunohistochemistry/case-2/3-anterior cingulate neocortex.jpg]

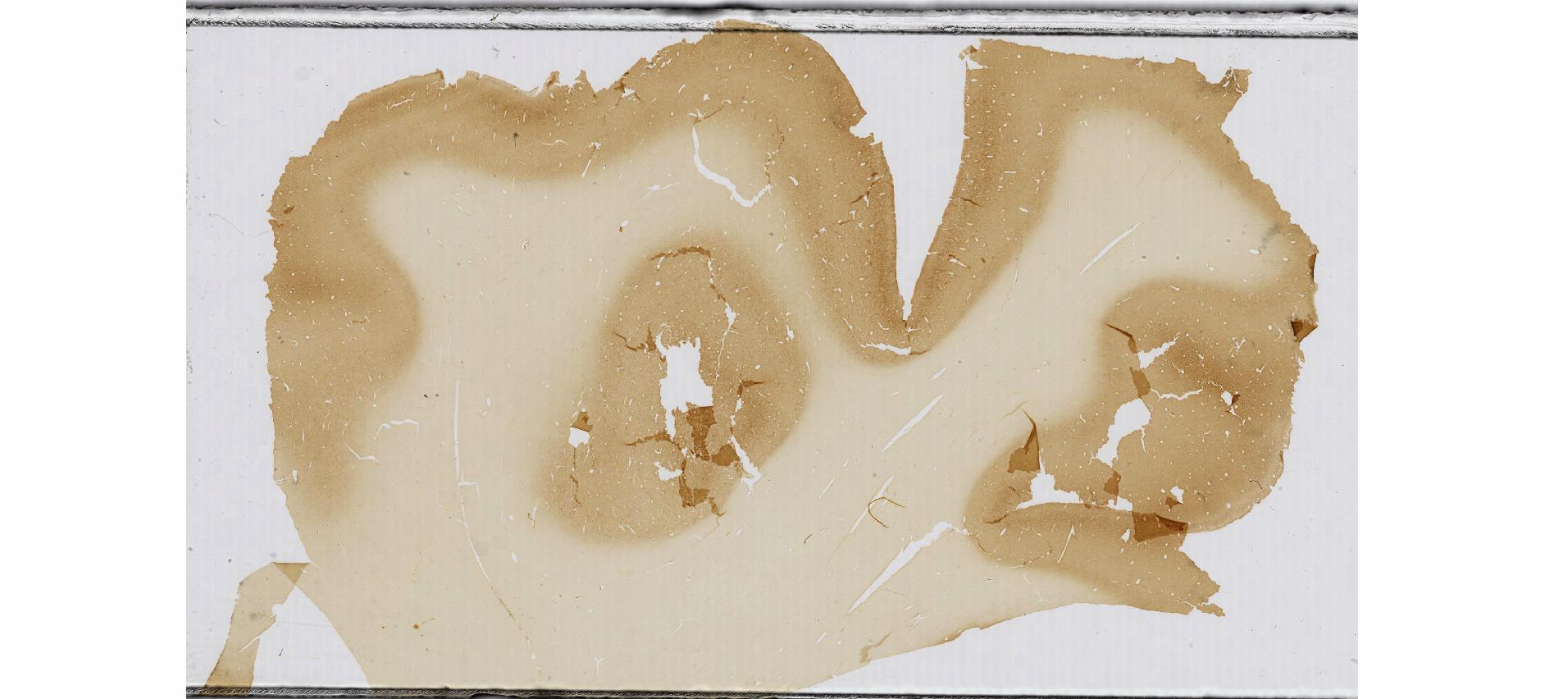

Supplement: Supplementary file 1 [file Presentation_1.ZIP › shank3-immunohistochemistry/case-2/4- precentral cortex.jpg]

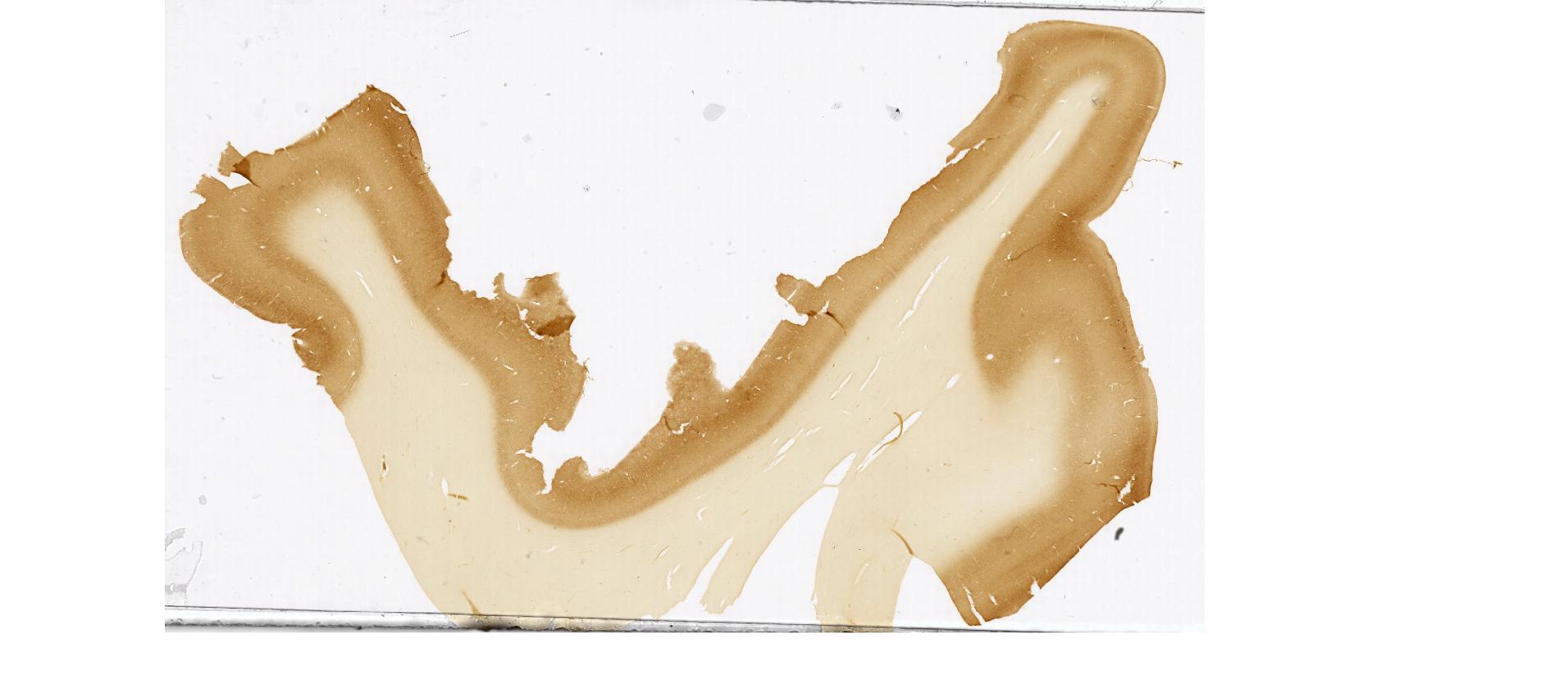

Supplement: Supplementary file 1 [file Presentation_1.ZIP › shank3-immunohistochemistry/case-2/5-postcentral cortex.jpg]

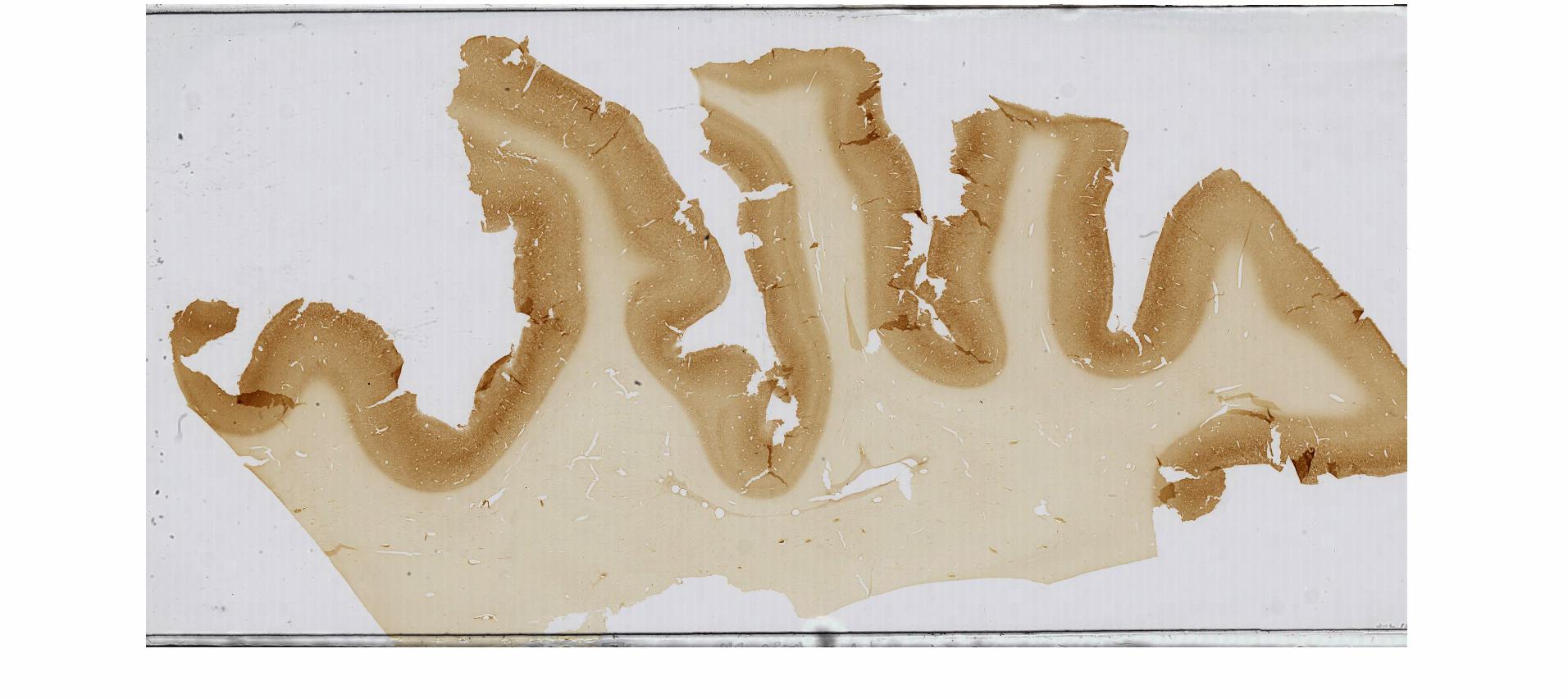

Supplement: Supplementary file 1 [file Presentation_1.ZIP › shank3-immunohistochemistry/case-2/6- visual cortex.jpg]

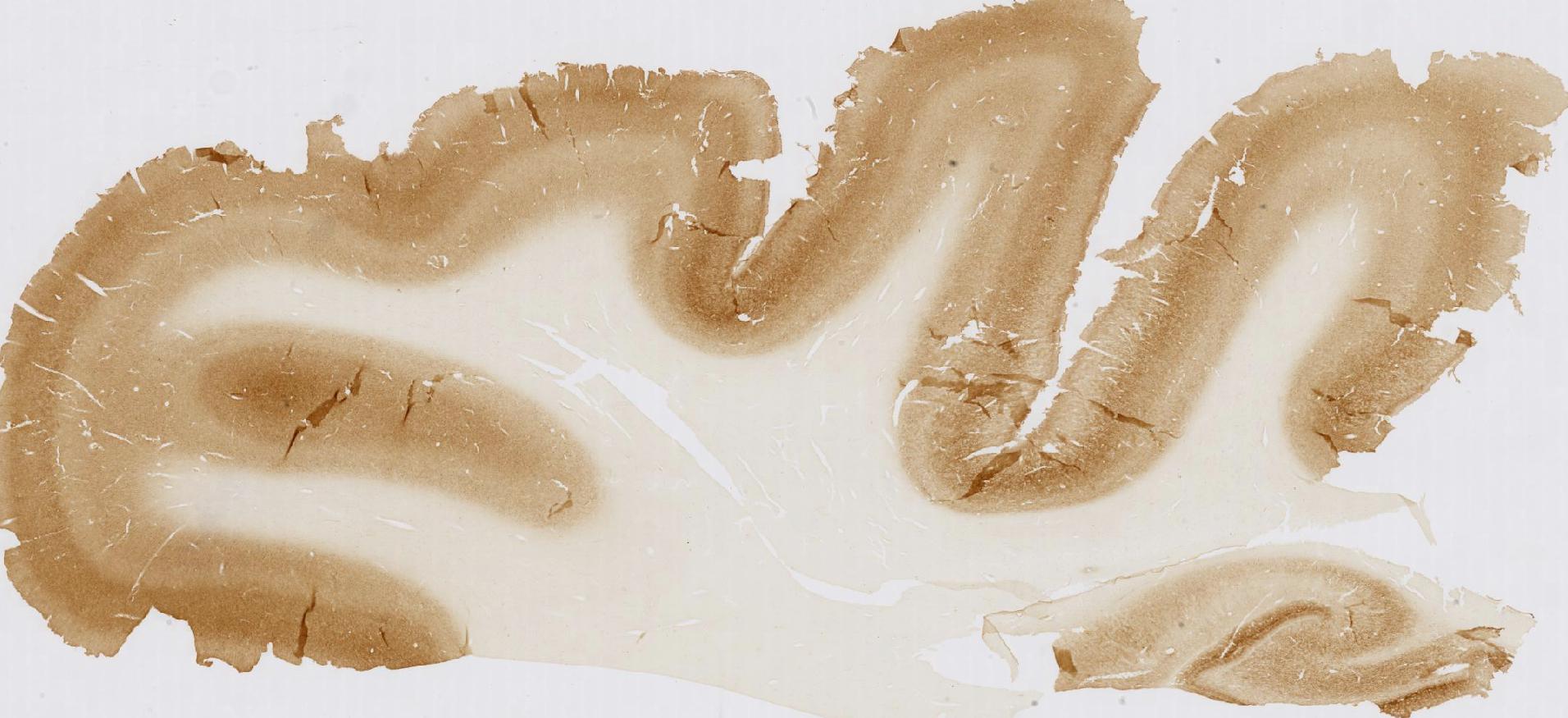

Supplement: Supplementary file 1 [file Presentation_1.ZIP › shank3-immunohistochemistry/case-2/7- hippocampal formation.jpg]

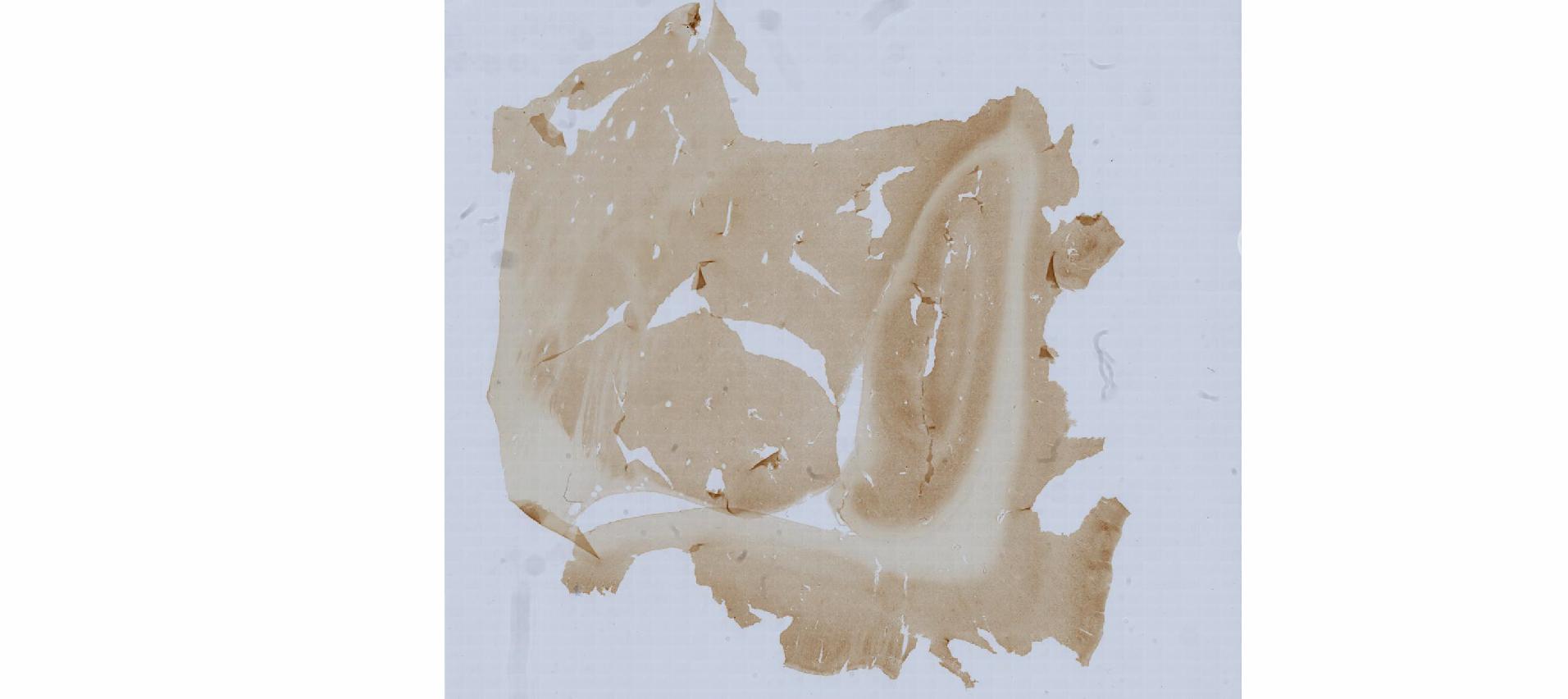

Supplement: Supplementary file 1 [file Presentation_1.ZIP › shank3-immunohistochemistry/case-2/8-amygdalar complex.jpg]

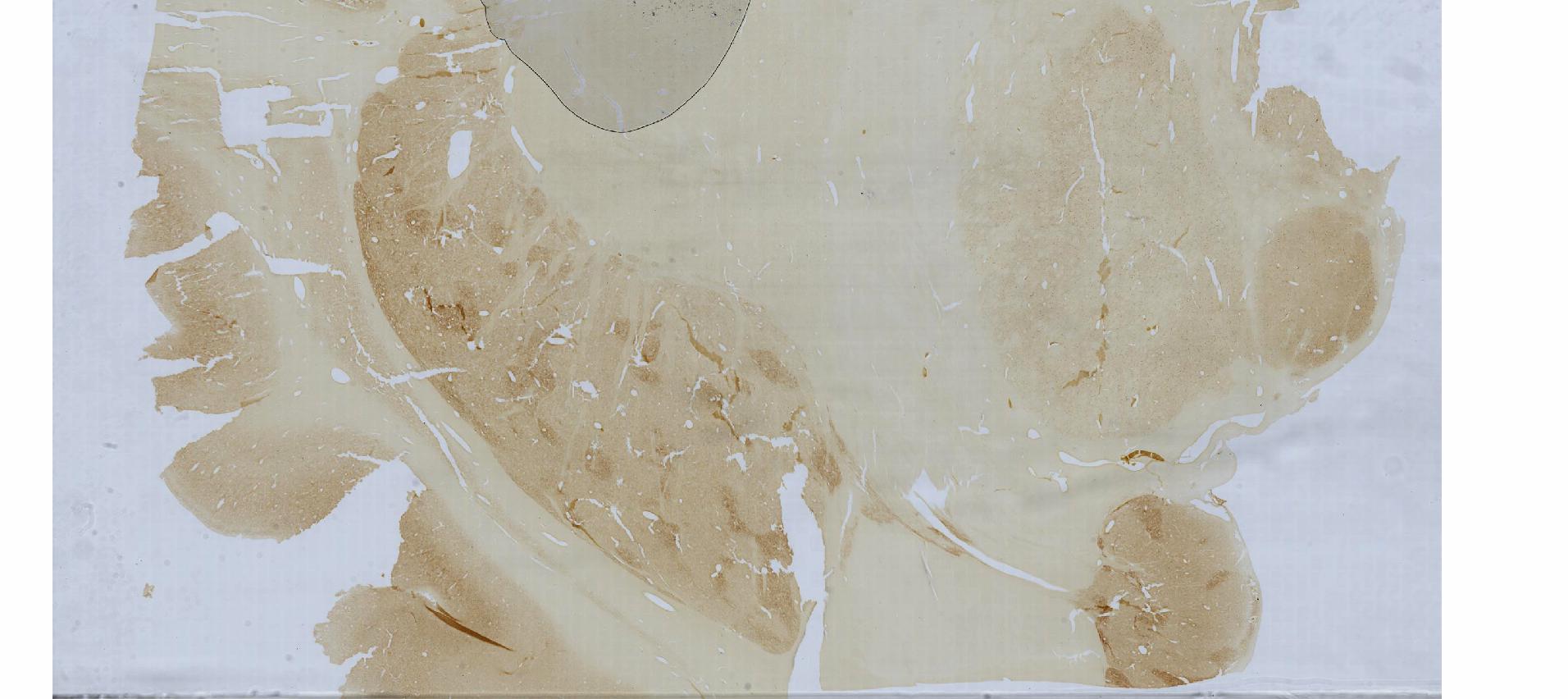

Supplement: Supplementary file 1 [file Presentation_1.ZIP › shank3-immunohistochemistry/case-2/9-basal ganglia and diencephalon.jpg]

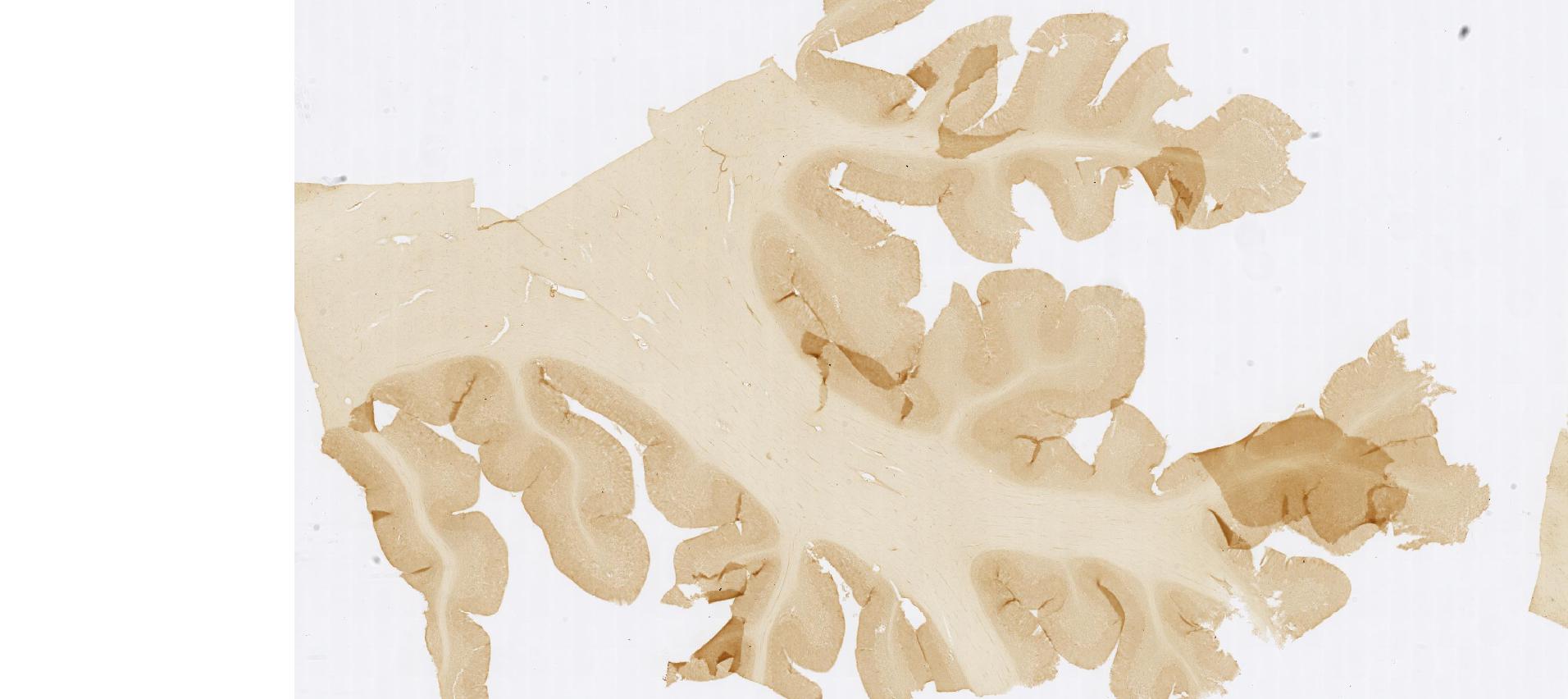

Supplement: Supplementary file 1 [file Presentation_1.ZIP › shank3-immunohistochemistry/case-20/cerebellum.jpg]

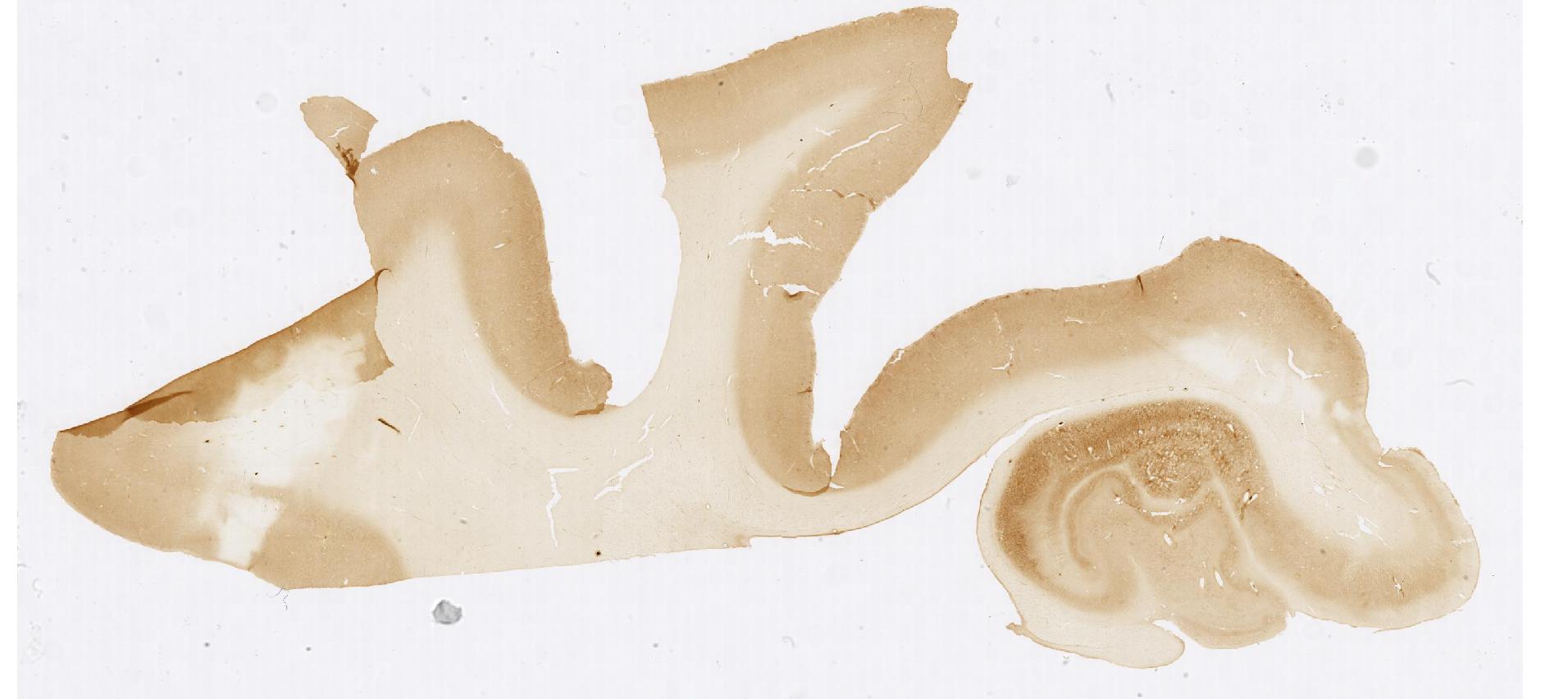

Supplement: Supplementary file 1 [file Presentation_1.ZIP › shank3-immunohistochemistry/case-20/hippocampal foramtion.jpg]

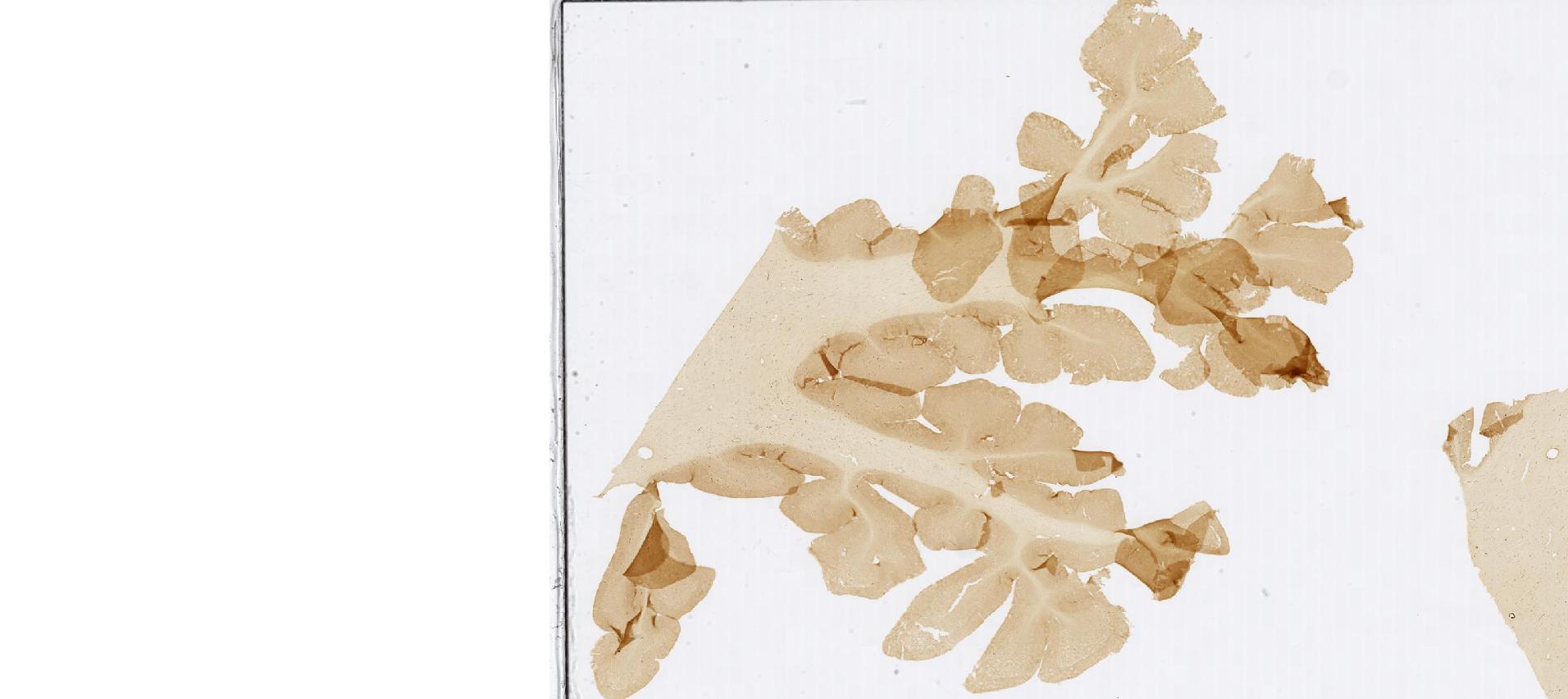

Supplement: Supplementary file 1 [file Presentation_1.ZIP › shank3-immunohistochemistry/case-21/cerebellum.jpg]

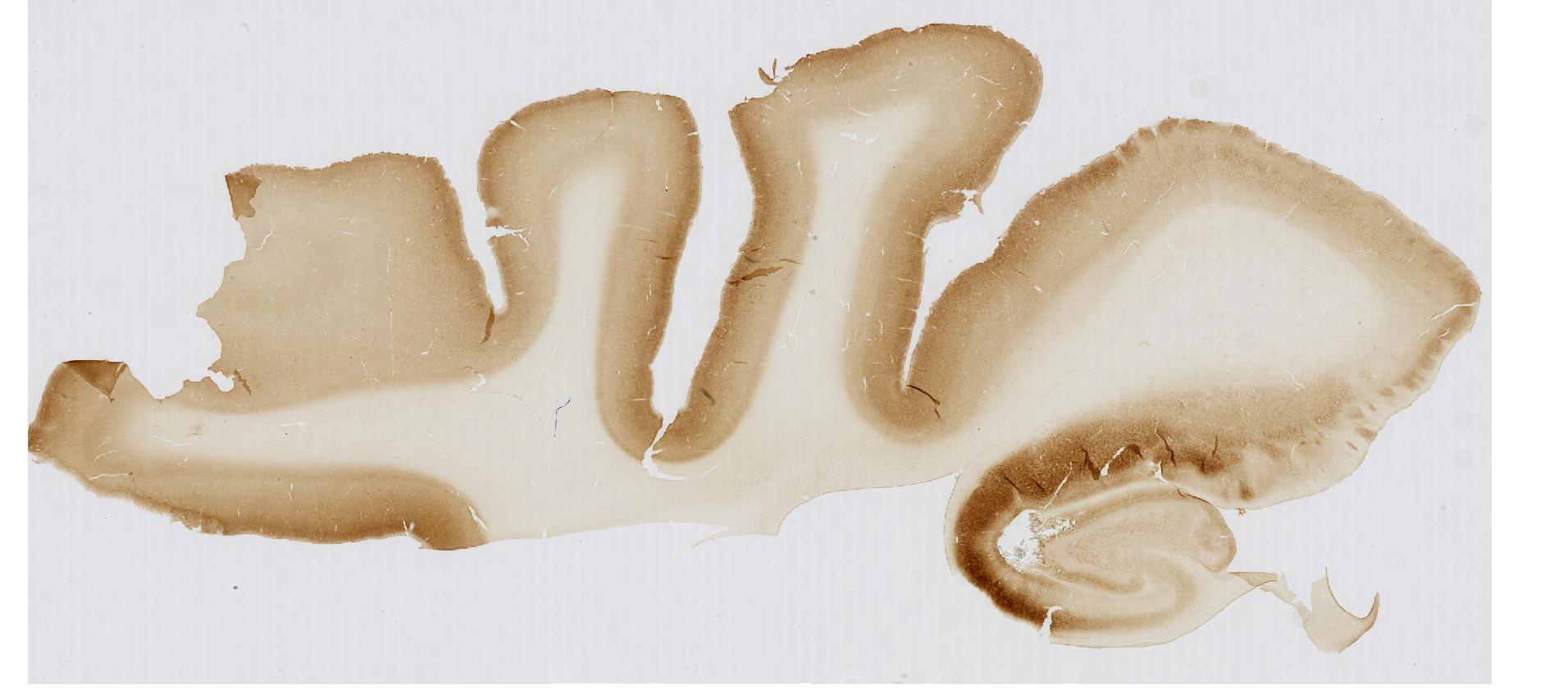

Supplement: Supplementary file 1 [file Presentation_1.ZIP › shank3-immunohistochemistry/case-21/hippocampal formation.jpg]

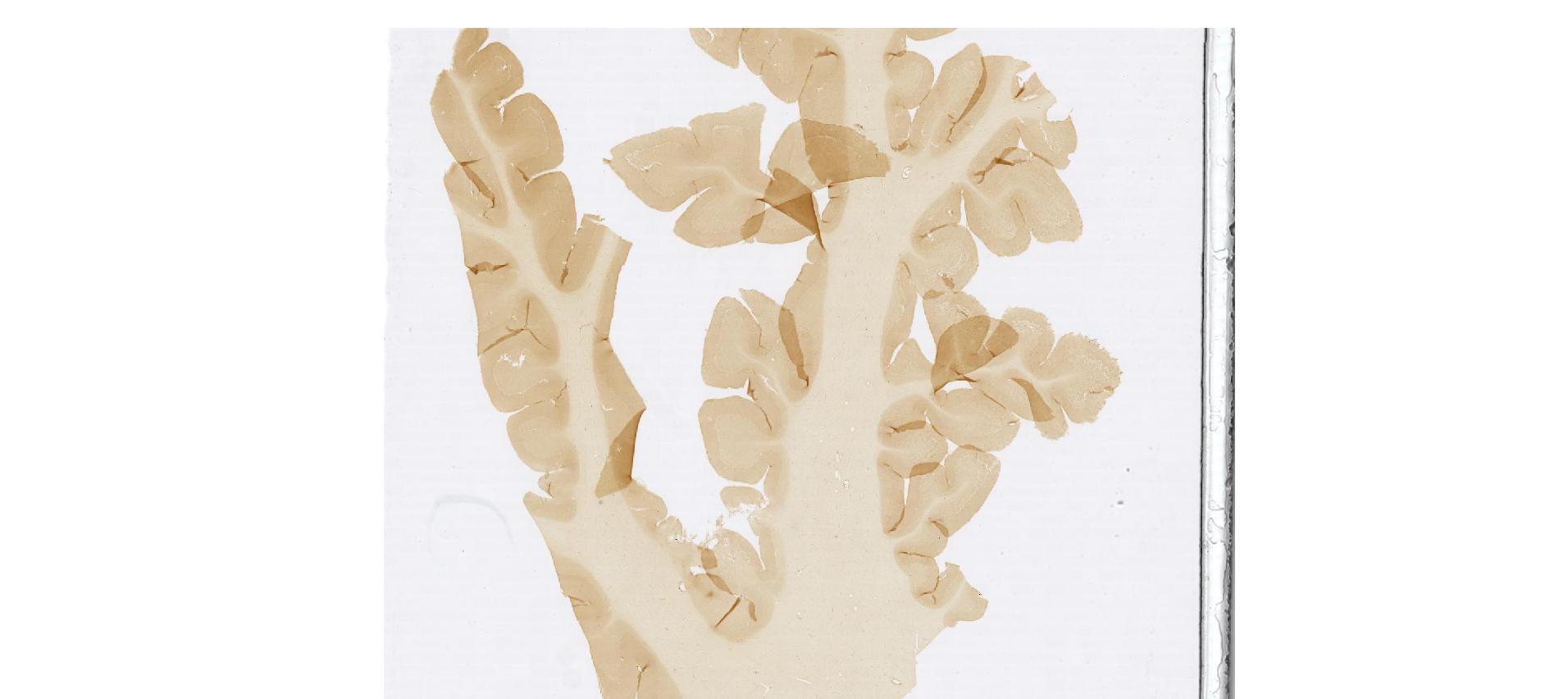

Supplement: Supplementary file 1 [file Presentation_1.ZIP › shank3-immunohistochemistry/case-22/cerebellum.jpg]

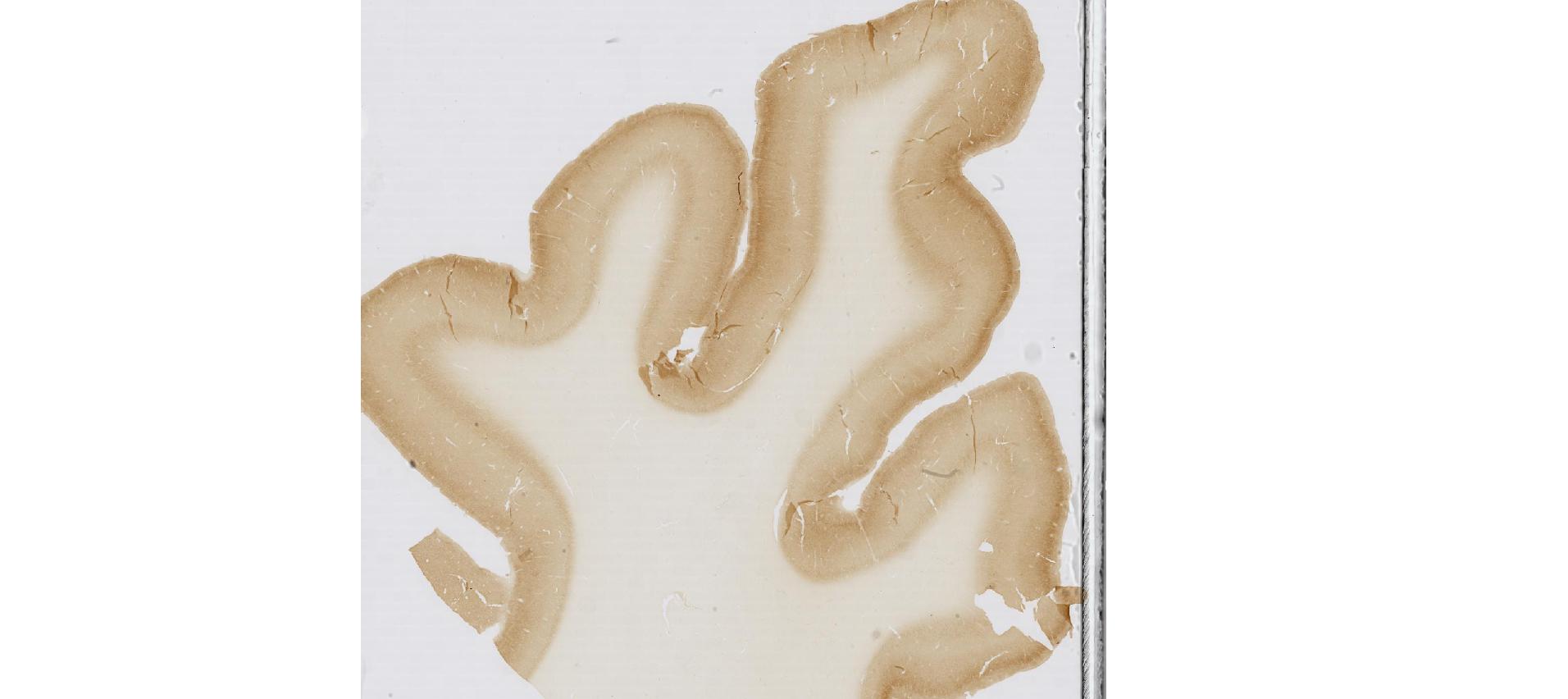

Supplement: Supplementary file 1 [file Presentation_1.ZIP › shank3-immunohistochemistry/case-22/frontal cortex.jpg]

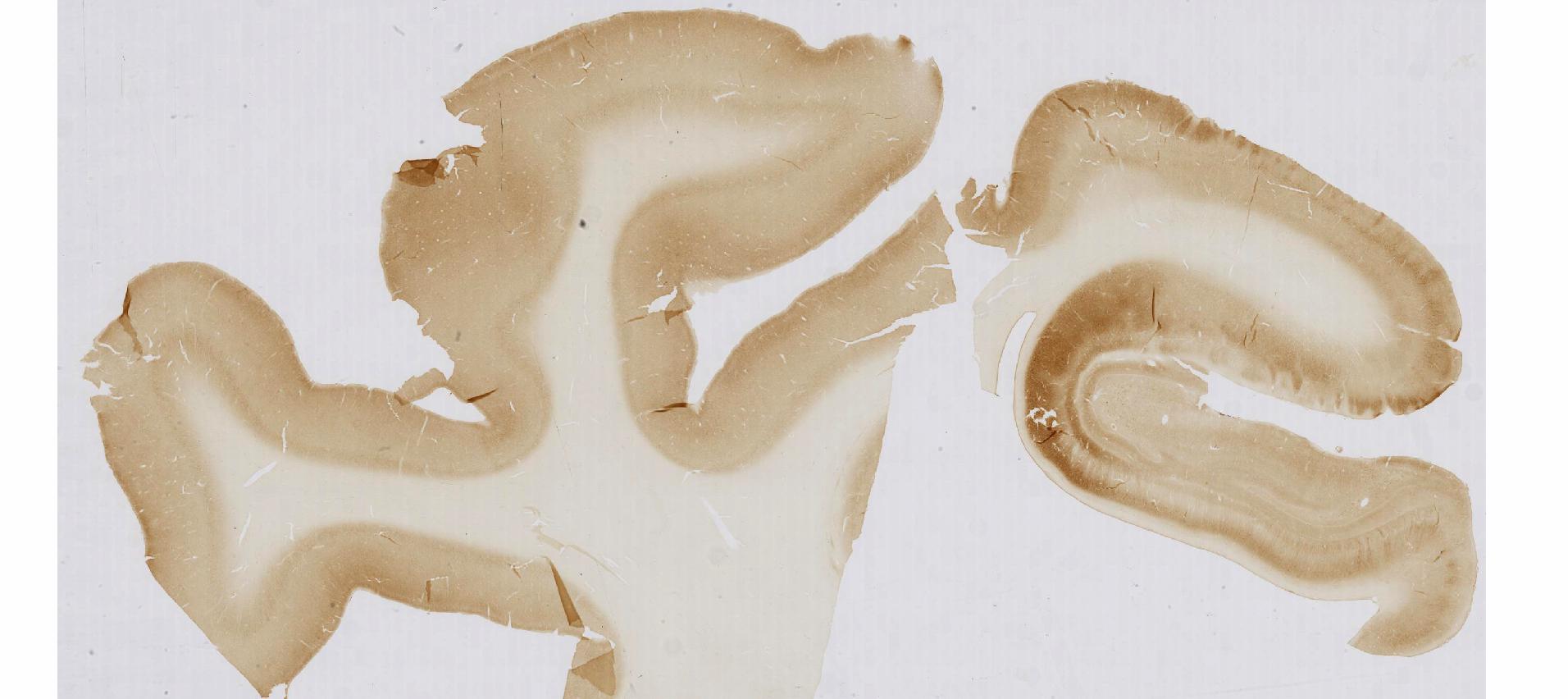

Supplement: Supplementary file 1 [file Presentation_1.ZIP › shank3-immunohistochemistry/case-22/hippocampal formation.jpg]

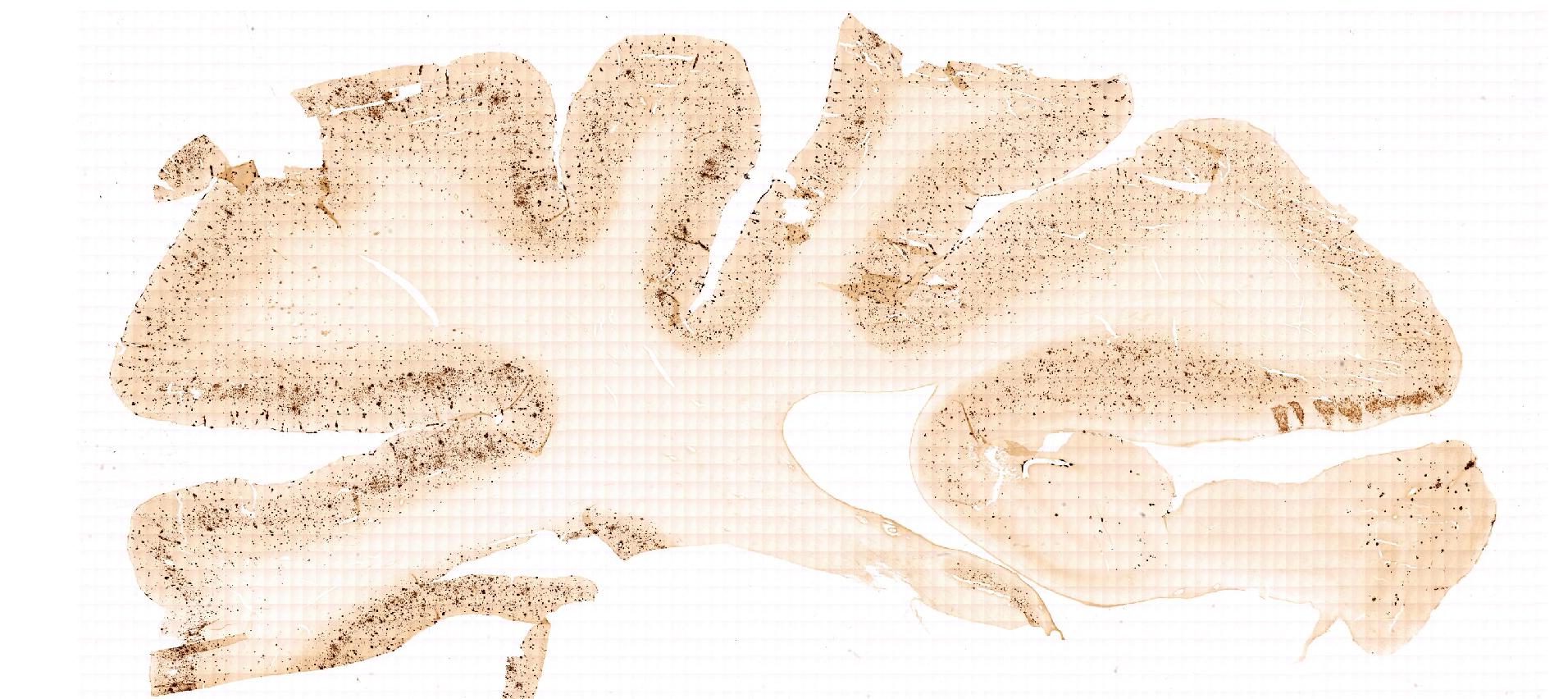

Supplement: Supplementary file 1 [file Presentation_1.ZIP › shank3-immunohistochemistry/case-23/HP-6E10.jpg]

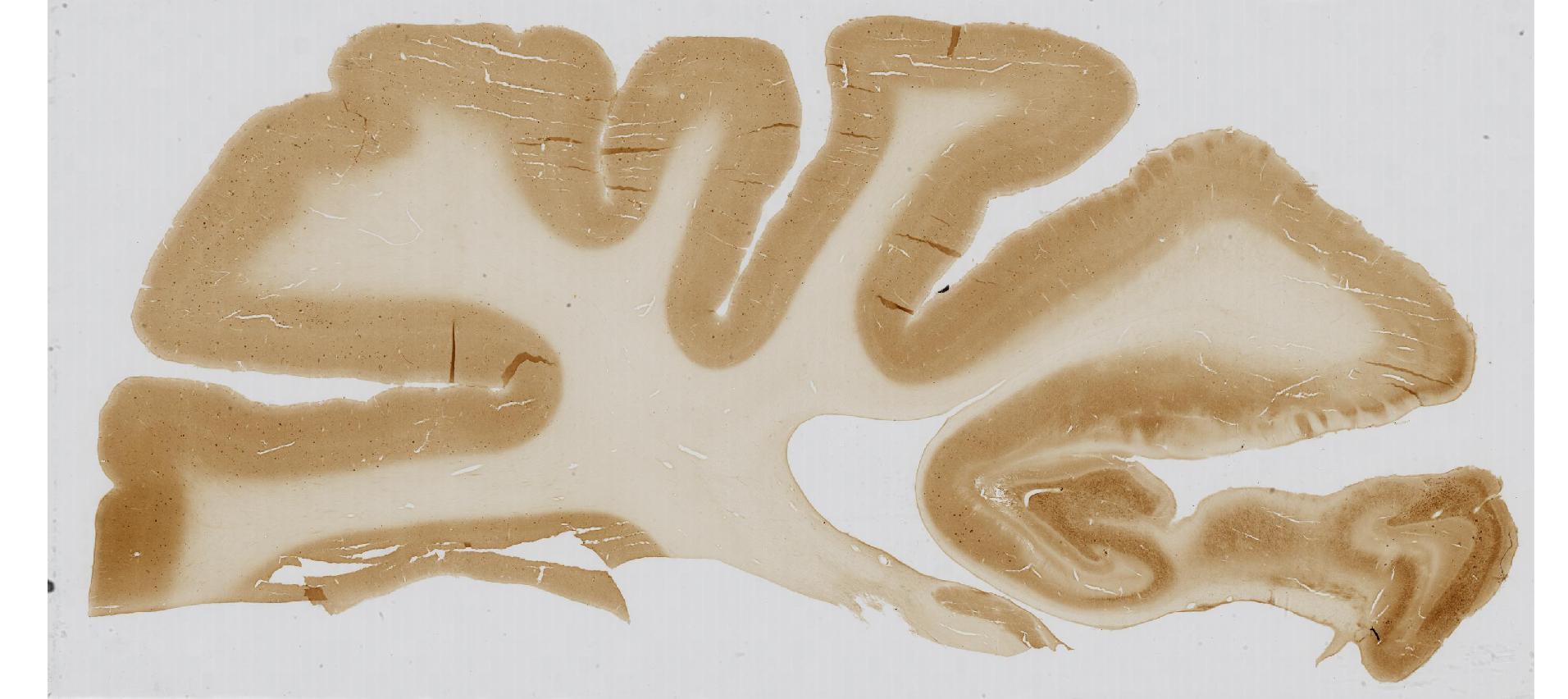

Supplement: Supplementary file 1 [file Presentation_1.ZIP › shank3-immunohistochemistry/case-23/HP-bace1.jpg]

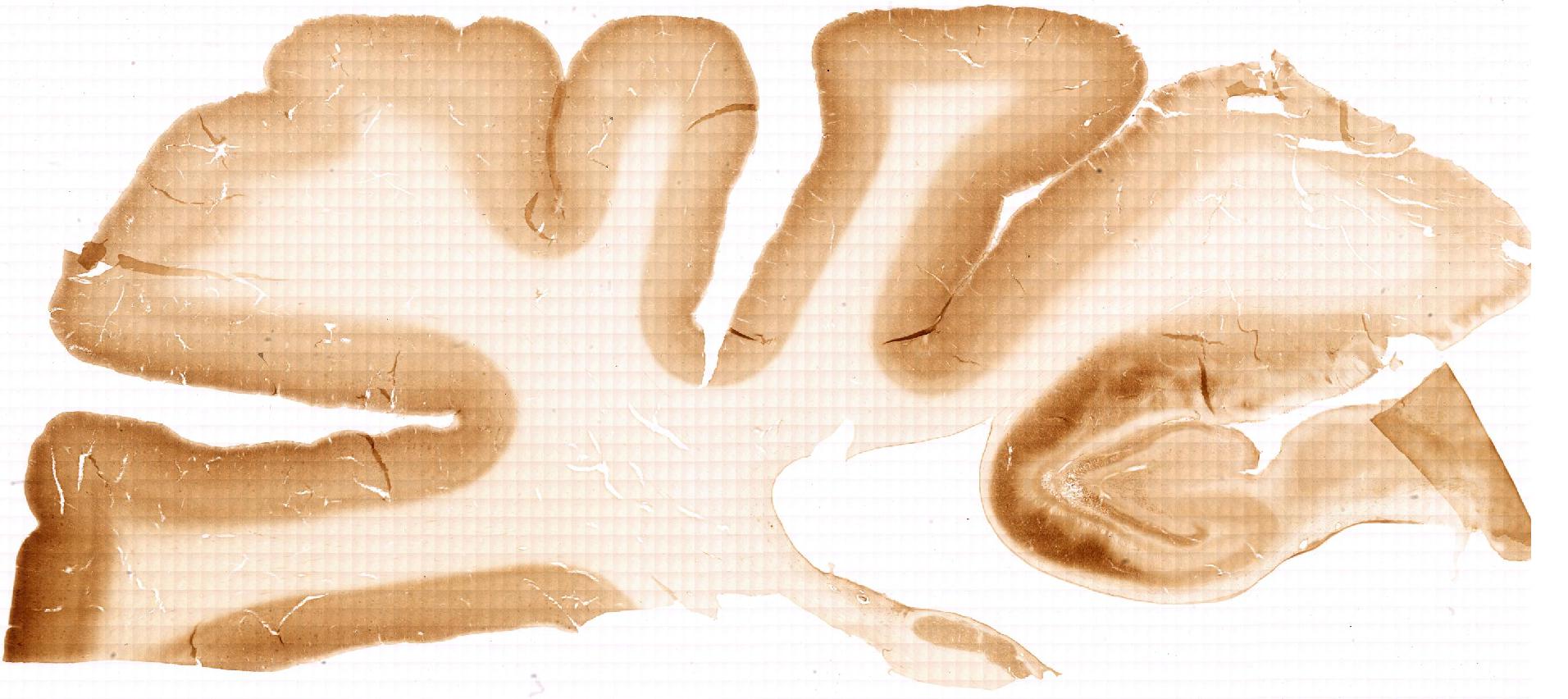

Supplement: Supplementary file 1 [file Presentation_1.ZIP › shank3-immunohistochemistry/case-23/HP-shank3.jpg]

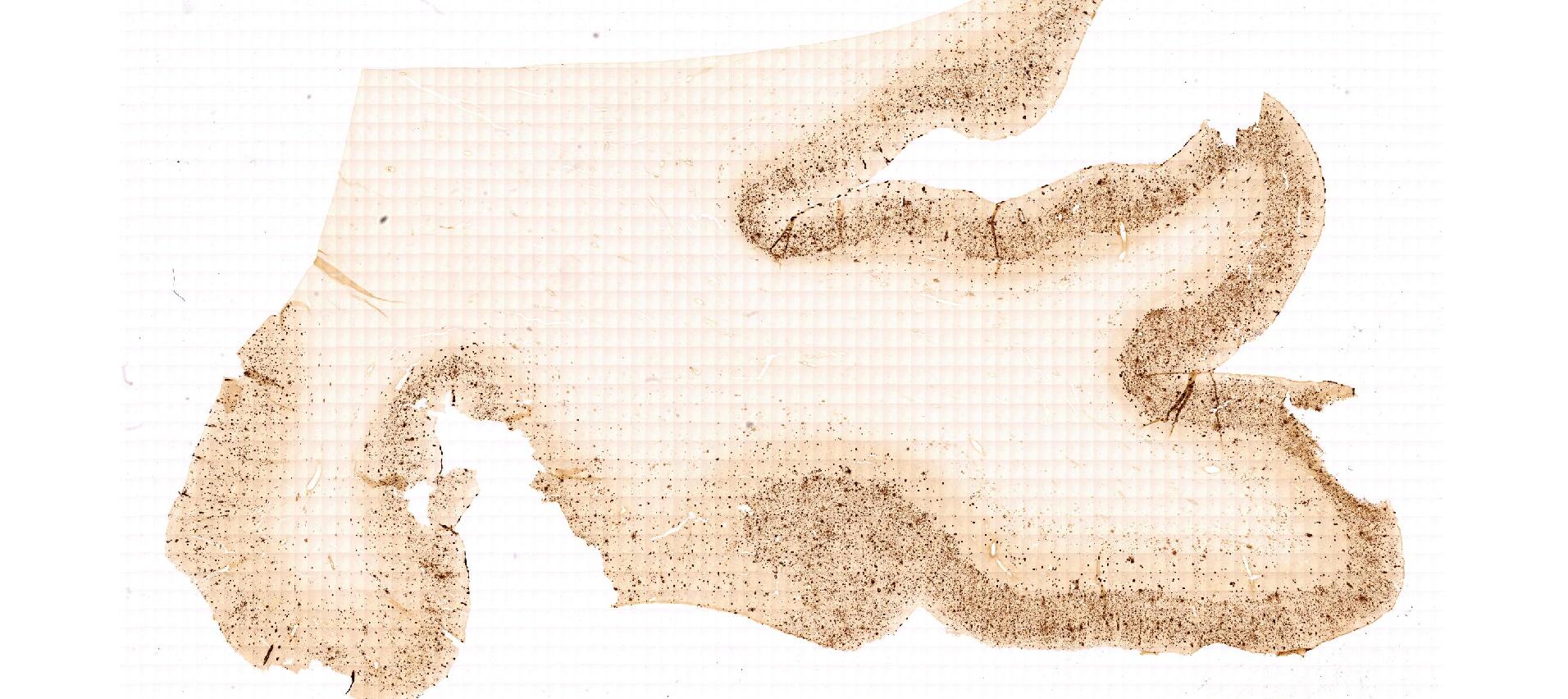

Supplement: Supplementary file 1 [file Presentation_1.ZIP › shank3-immunohistochemistry/case-23/PFC-6E10.jpg]

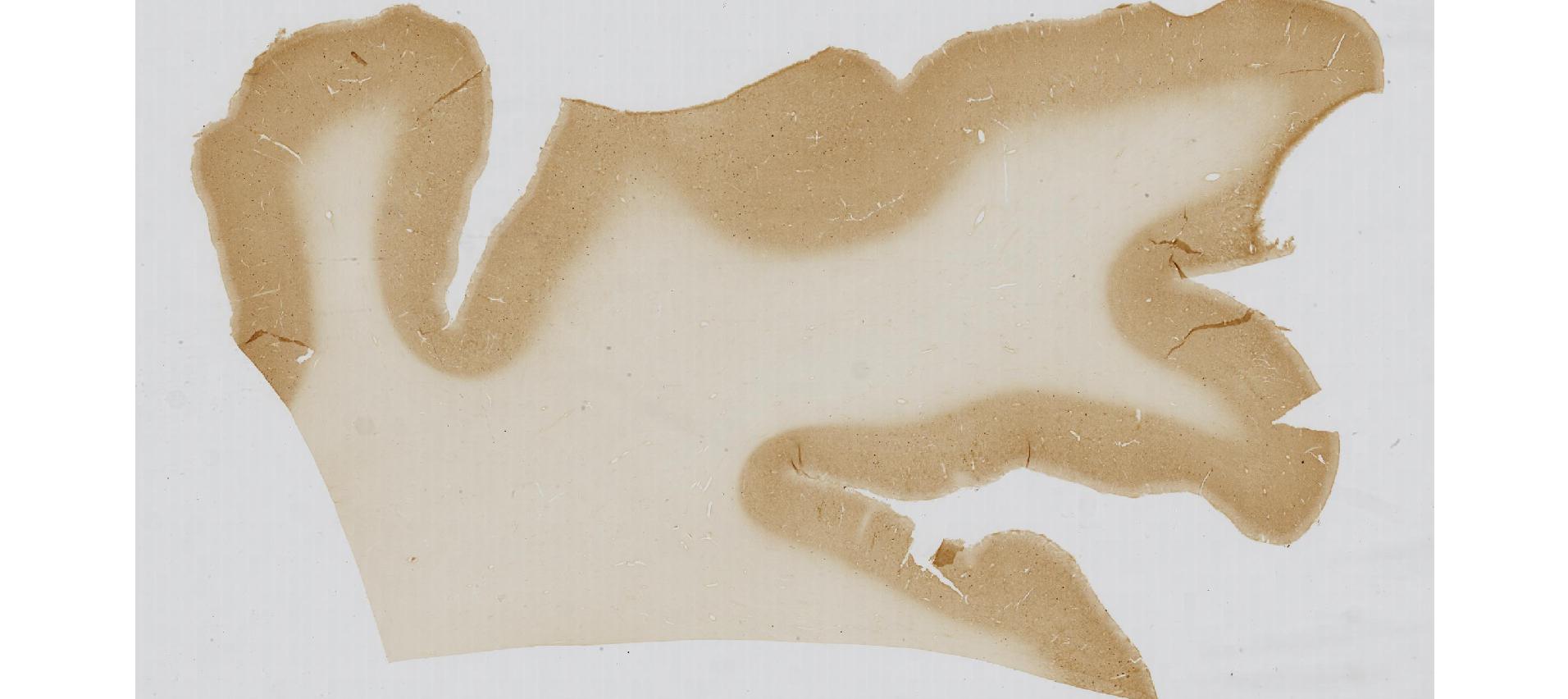

Supplement: Supplementary file 1 [file Presentation_1.ZIP › shank3-immunohistochemistry/case-23/PFC-bace1.jpg]

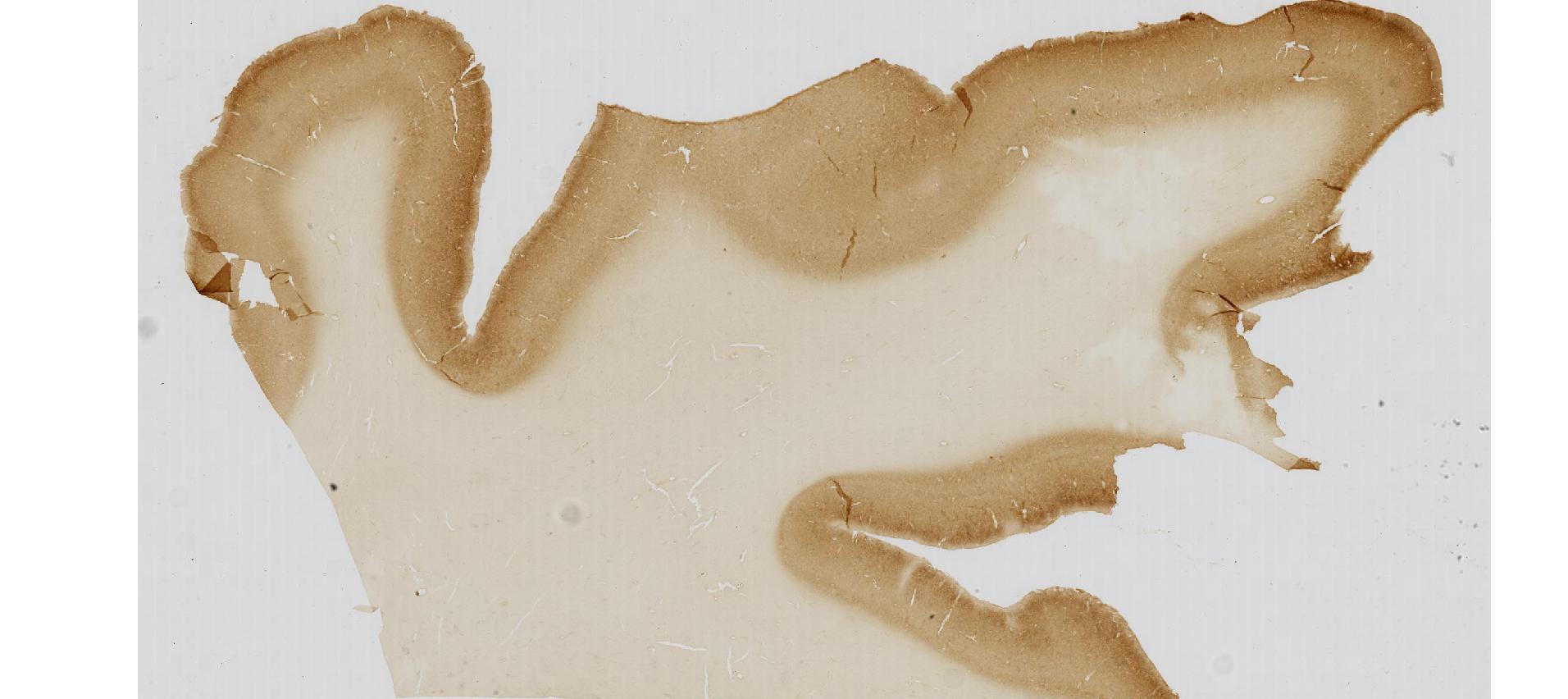

Supplement: Supplementary file 1 [file Presentation_1.ZIP › shank3-immunohistochemistry/case-23/PFC-shank3.jpg]

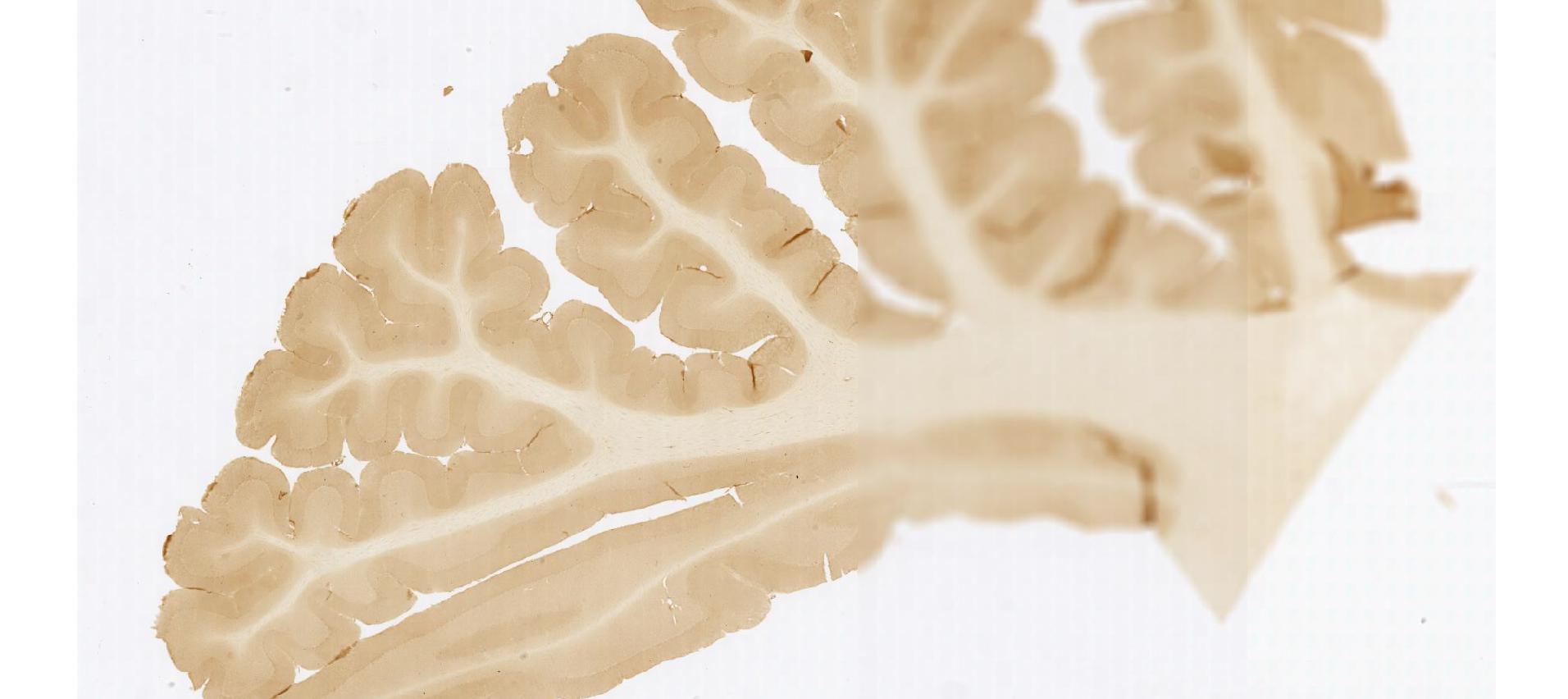

Supplement: Supplementary file 1 [file Presentation_1.ZIP › shank3-immunohistochemistry/case-23/cerebellum-shank3.jpg]
