# Supplementary figures and images for: Expression of the Excitatory Postsynaptic Scaffolding Protein, Shank3, in Human Brain: Effect of Age and Alzheimer’s Disease (part 3 of 3)
Source: Front Aging Neurosci. 2021 Aug 24;13:717263. doi: 10.3389/fnagi.2021.717263 (PMC8421777; doi:10.3389/fnagi.2021.717263)

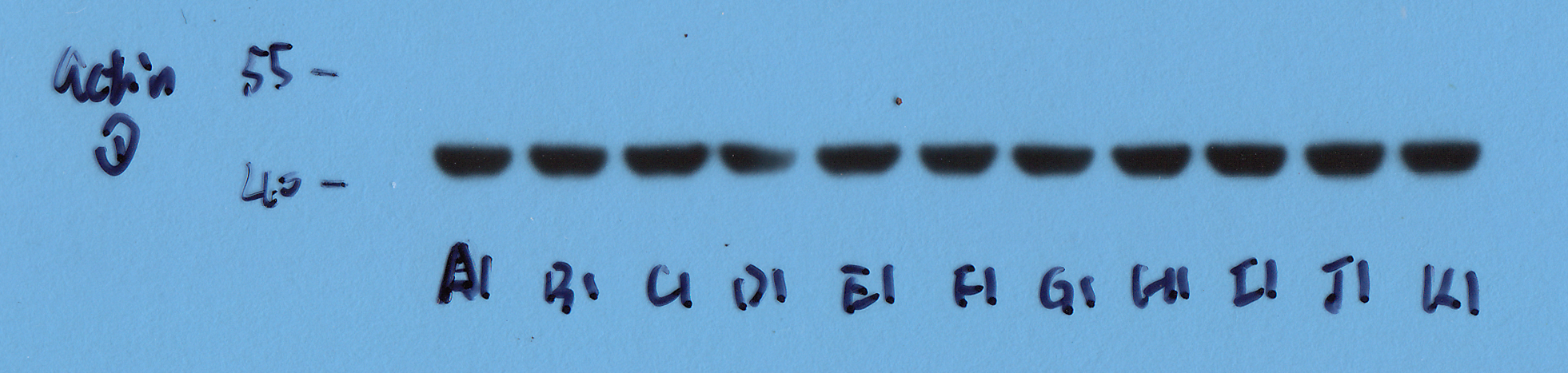

Supplement: Supplementary file 3 [file Presentation_3.ZIP › Figure 10D-Imaging with film exposure/Figure 10D-actin-case#6.tif]

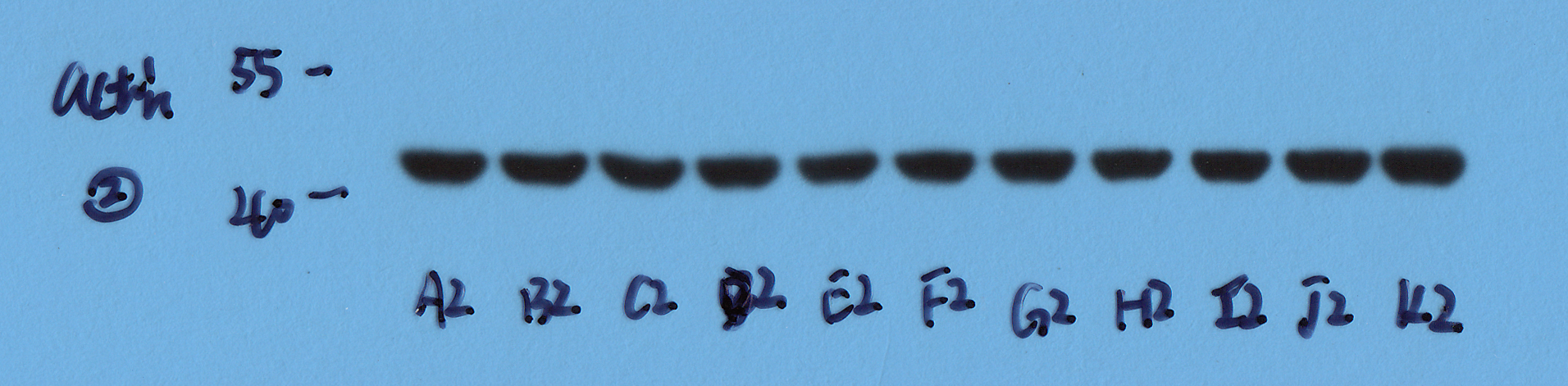

Supplement: Supplementary file 3 [file Presentation_3.ZIP › Figure 10D-Imaging with film exposure/Figure 10D-actin-case#9.tif]

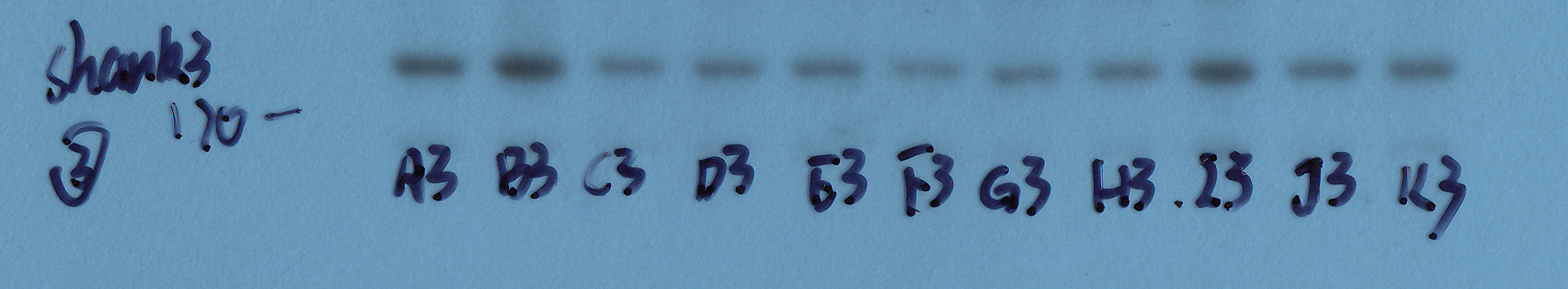

Supplement: Supplementary file 3 [file Presentation_3.ZIP › Figure 10D-Imaging with film exposure/Figure 10D-shank3-case#14.tif]

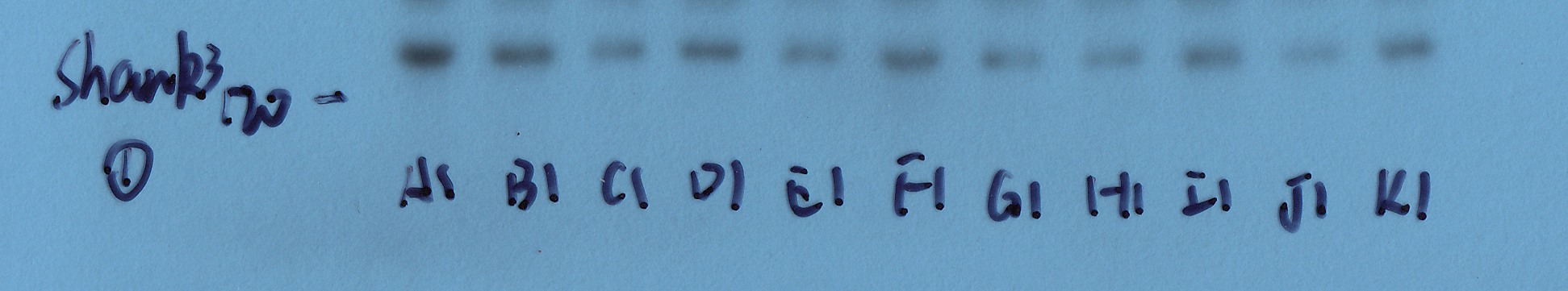

Supplement: Supplementary file 3 [file Presentation_3.ZIP › Figure 10D-Imaging with film exposure/Figure 10D-shank3-case#6.tif]

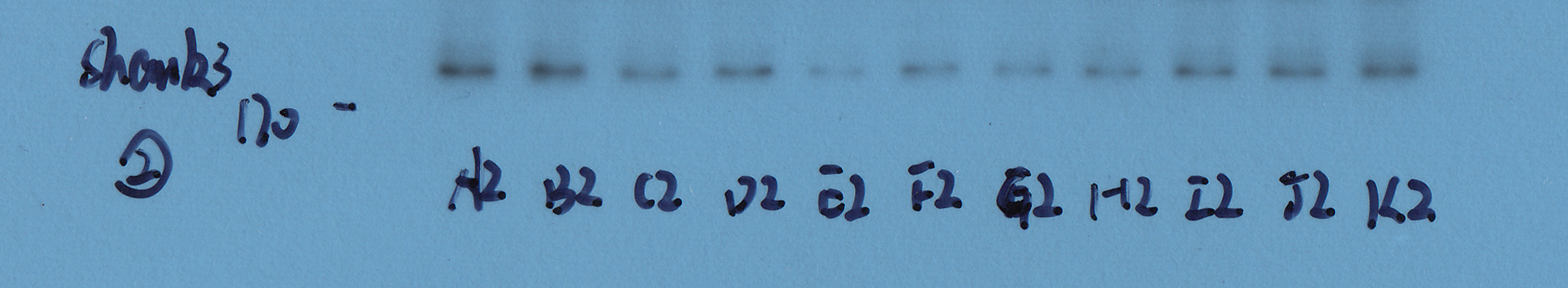

Supplement: Supplementary file 3 [file Presentation_3.ZIP › Figure 10D-Imaging with film exposure/Figure 10D-shank3-case9.tif]

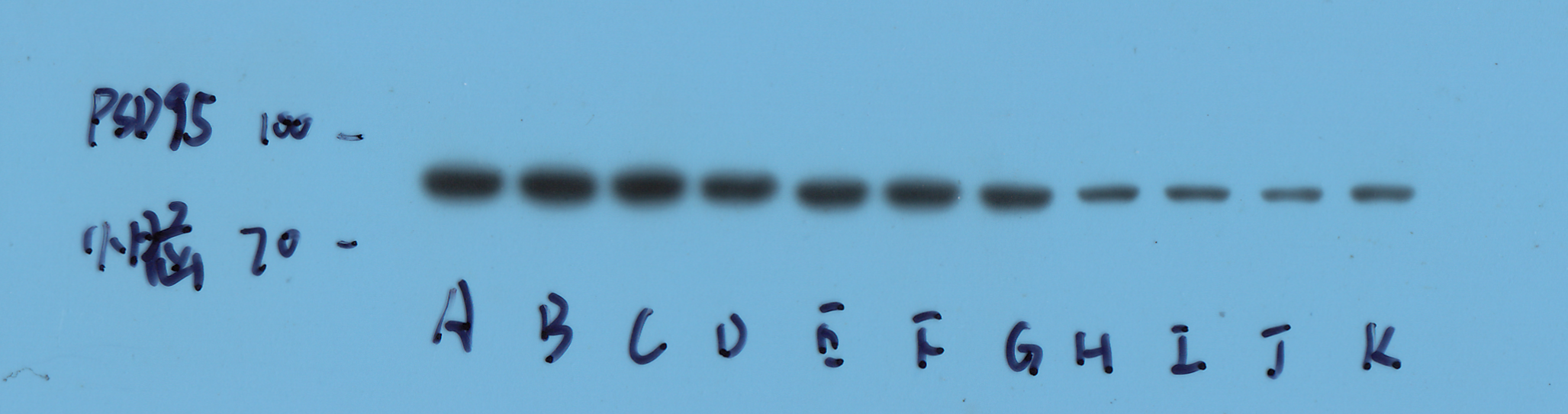

Supplement: Supplementary file 4 [file Presentation_4.ZIP › Figure 14-20210725/Figure 14A-different age-Imaging with film exposure/Figure 14A-PSD95-CBL.tif]

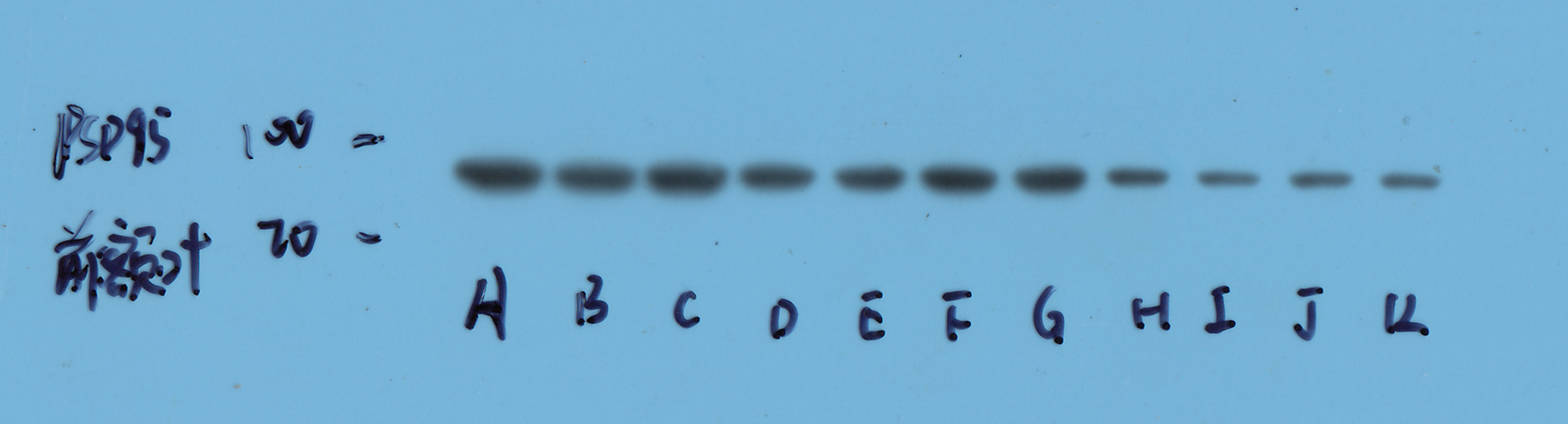

Supplement: Supplementary file 4 [file Presentation_4.ZIP › Figure 14-20210725/Figure 14A-different age-Imaging with film exposure/Figure 14A-PSD95-PFC.tif]

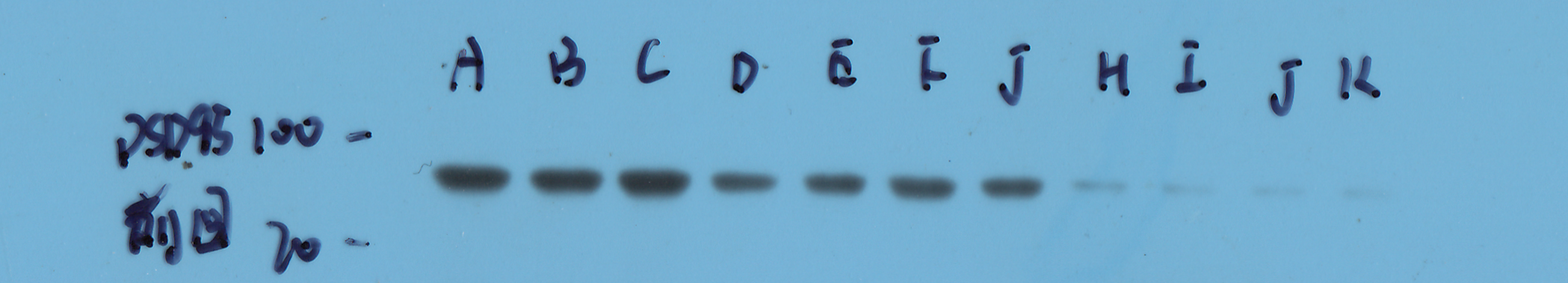

Supplement: Supplementary file 4 [file Presentation_4.ZIP › Figure 14-20210725/Figure 14A-different age-Imaging with film exposure/Figure 14A-PSD95-PrC.tif]

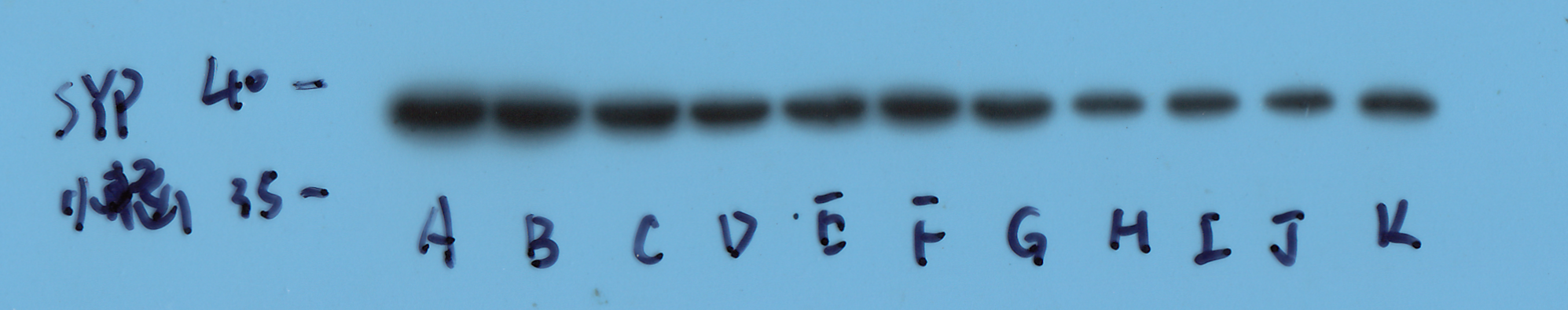

Supplement: Supplementary file 4 [file Presentation_4.ZIP › Figure 14-20210725/Figure 14A-different age-Imaging with film exposure/Figure 14A-SYP-CBL.tif]

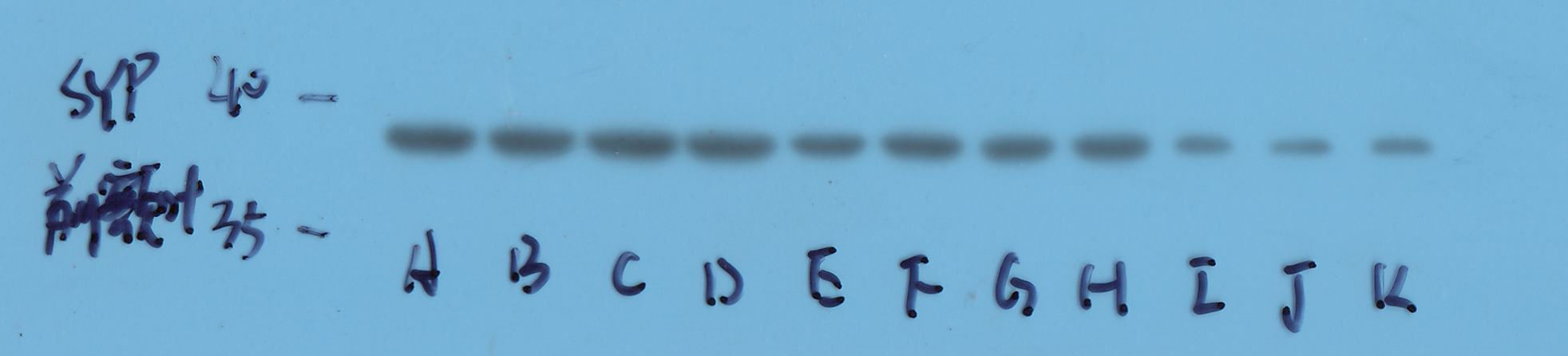

Supplement: Supplementary file 4 [file Presentation_4.ZIP › Figure 14-20210725/Figure 14A-different age-Imaging with film exposure/Figure 14A-SYP-PFC.tif]

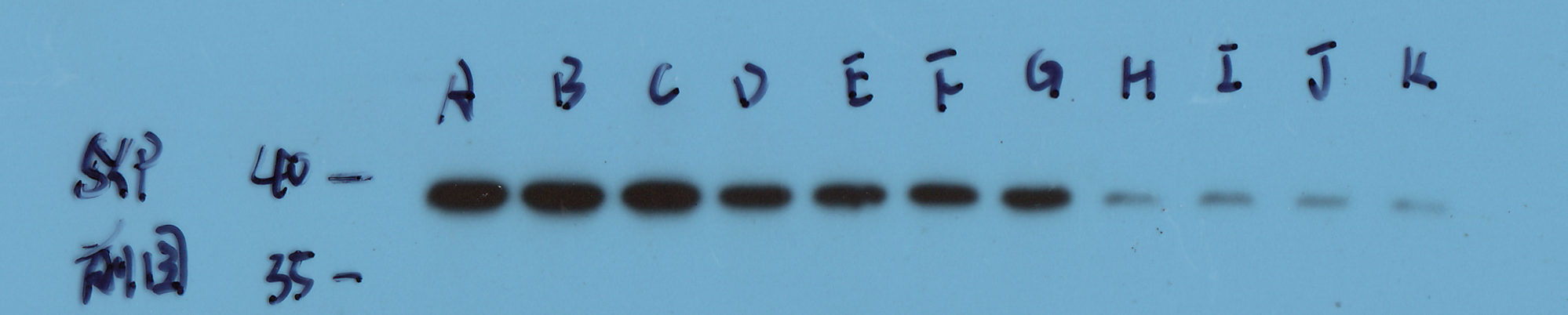

Supplement: Supplementary file 4 [file Presentation_4.ZIP › Figure 14-20210725/Figure 14A-different age-Imaging with film exposure/Figure 14A-SYP-PrC.tif]

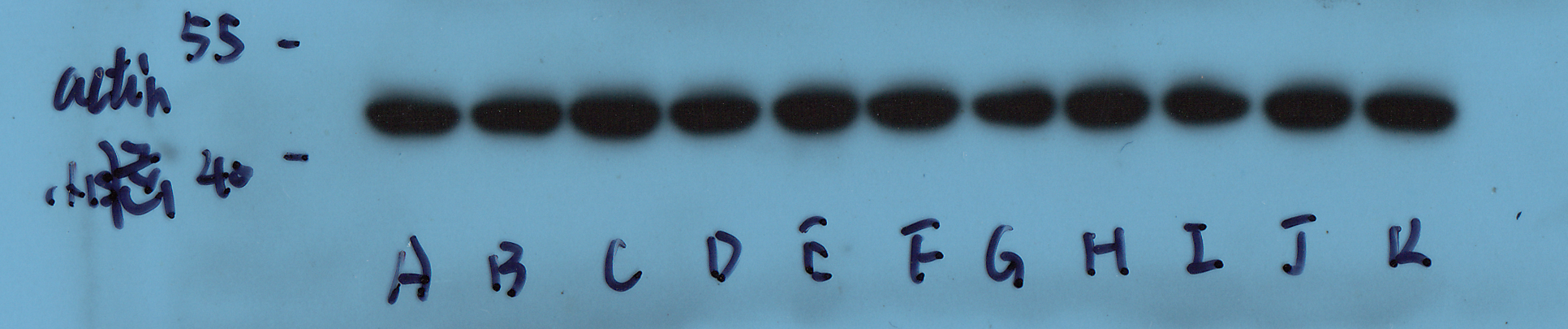

Supplement: Supplementary file 4 [file Presentation_4.ZIP › Figure 14-20210725/Figure 14A-different age-Imaging with film exposure/Figure 14A-actin-CBL.tif]

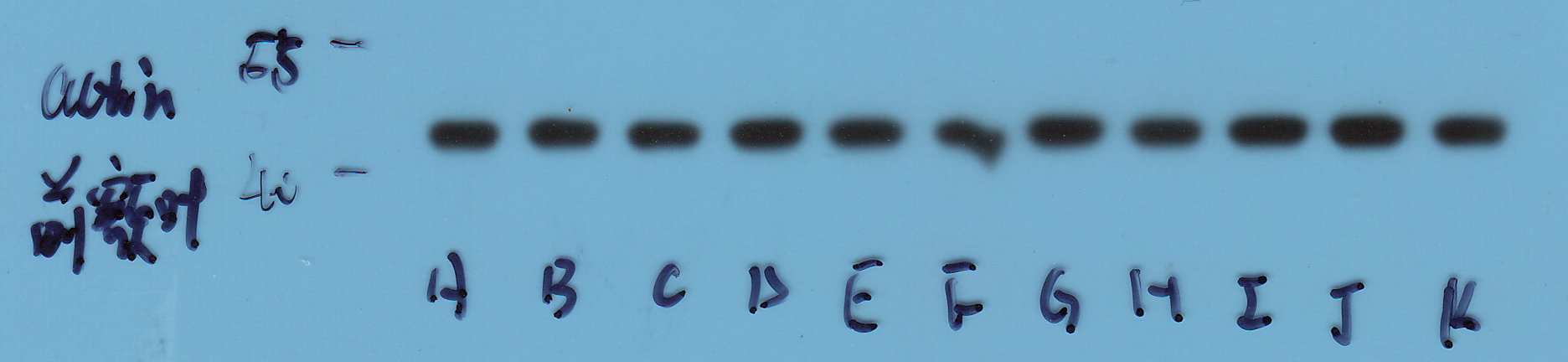

Supplement: Supplementary file 4 [file Presentation_4.ZIP › Figure 14-20210725/Figure 14A-different age-Imaging with film exposure/Figure 14A-actin-PFC.tif]

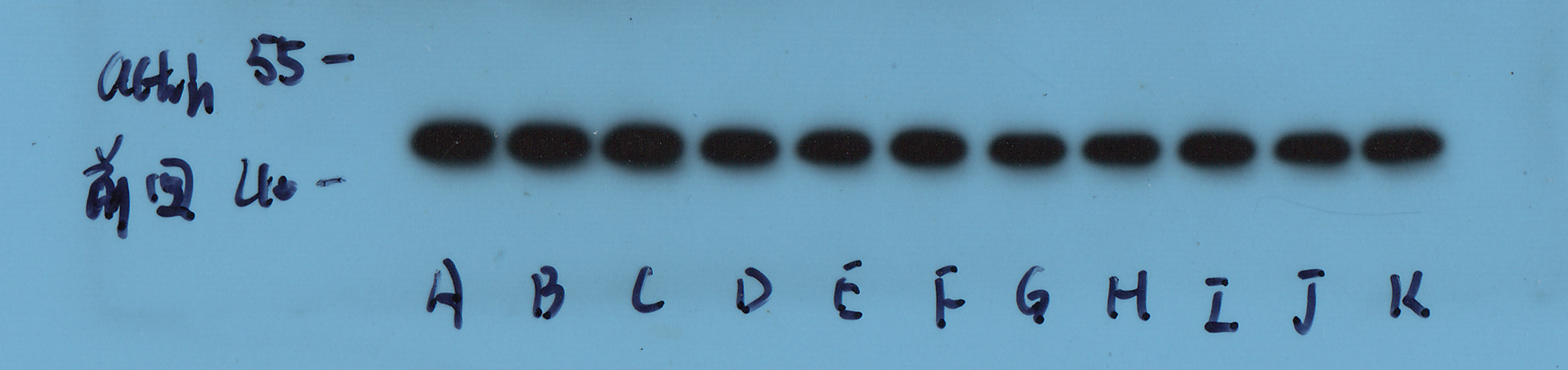

Supplement: Supplementary file 4 [file Presentation_4.ZIP › Figure 14-20210725/Figure 14A-different age-Imaging with film exposure/Figure 14A-actin-PrC.tif]

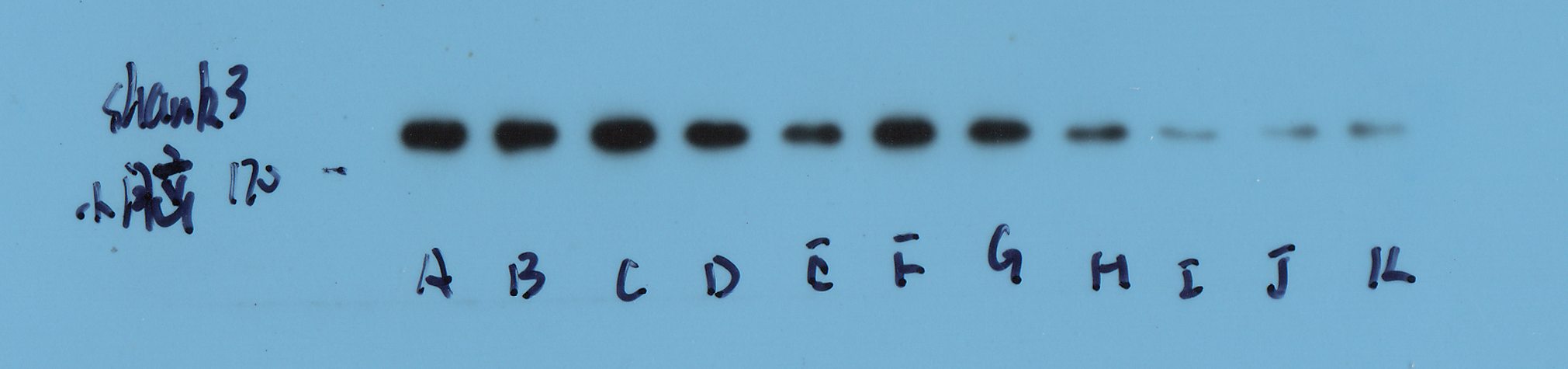

Supplement: Supplementary file 4 [file Presentation_4.ZIP › Figure 14-20210725/Figure 14A-different age-Imaging with film exposure/Figure 14A-shank3-CBL.tif]

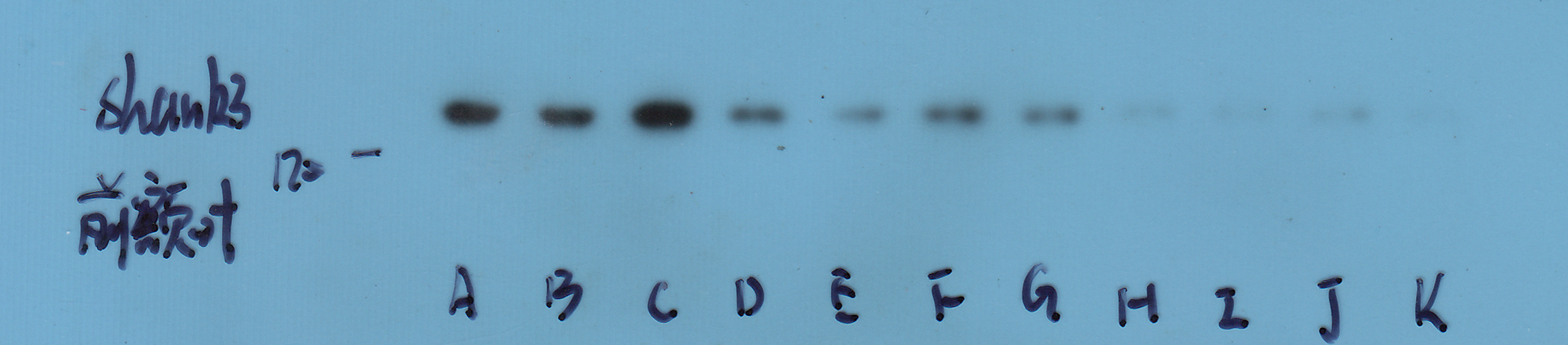

Supplement: Supplementary file 4 [file Presentation_4.ZIP › Figure 14-20210725/Figure 14A-different age-Imaging with film exposure/Figure 14A-shank3-PFC.tif]

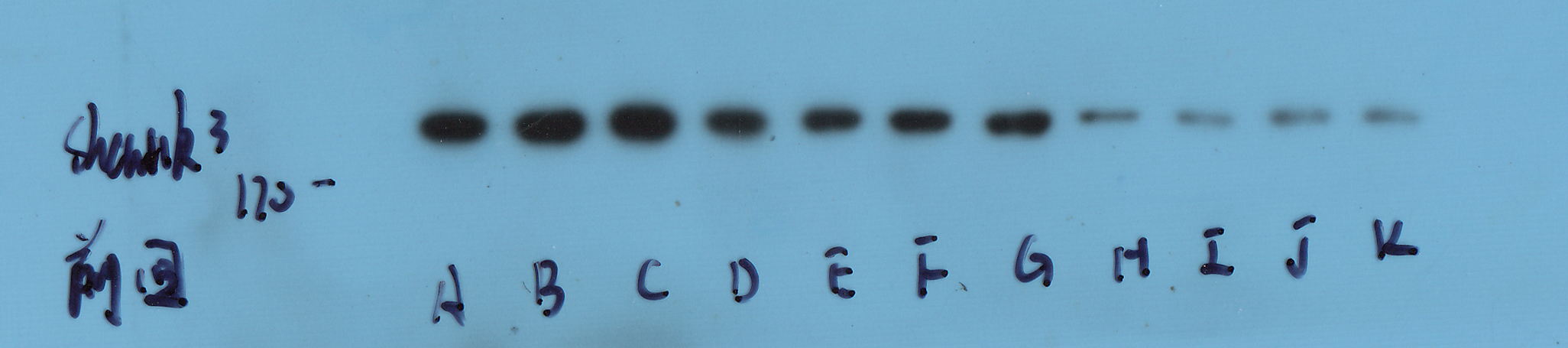

Supplement: Supplementary file 4 [file Presentation_4.ZIP › Figure 14-20210725/Figure 14A-different age-Imaging with film exposure/Figure 14A-shank3-PrC.tif]

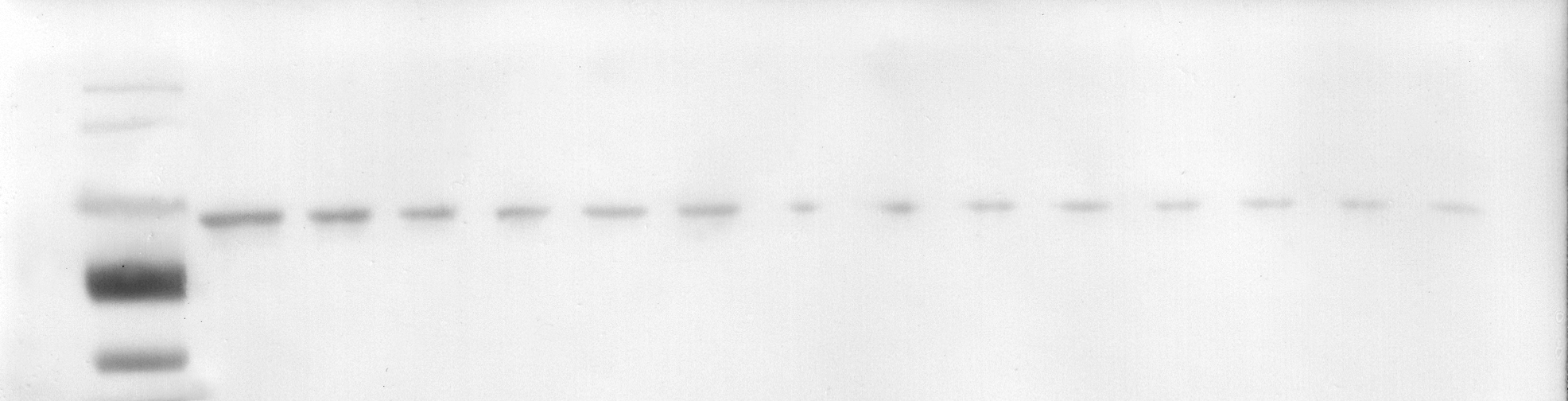

Supplement: Supplementary file 4 [file Presentation_4.ZIP › Figure 14-20210725/Figure 14B-AD-Imaging with the UVP ChemStudio PLUS device/Figure 14B-PSD95.tif]

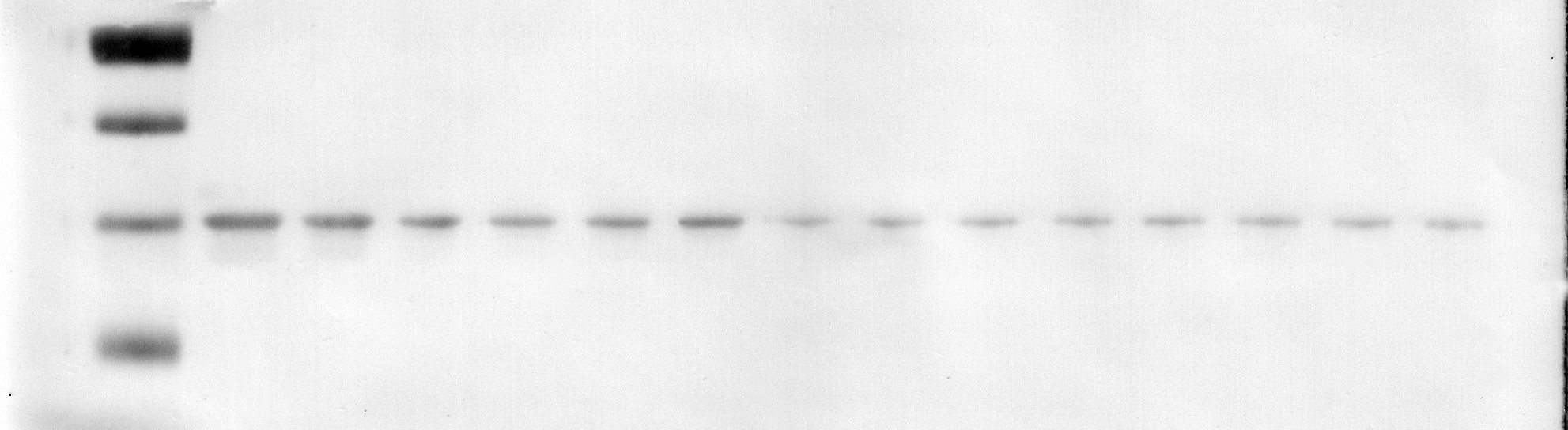

Supplement: Supplementary file 4 [file Presentation_4.ZIP › Figure 14-20210725/Figure 14B-AD-Imaging with the UVP ChemStudio PLUS device/Figure 14B-SYP.tif]

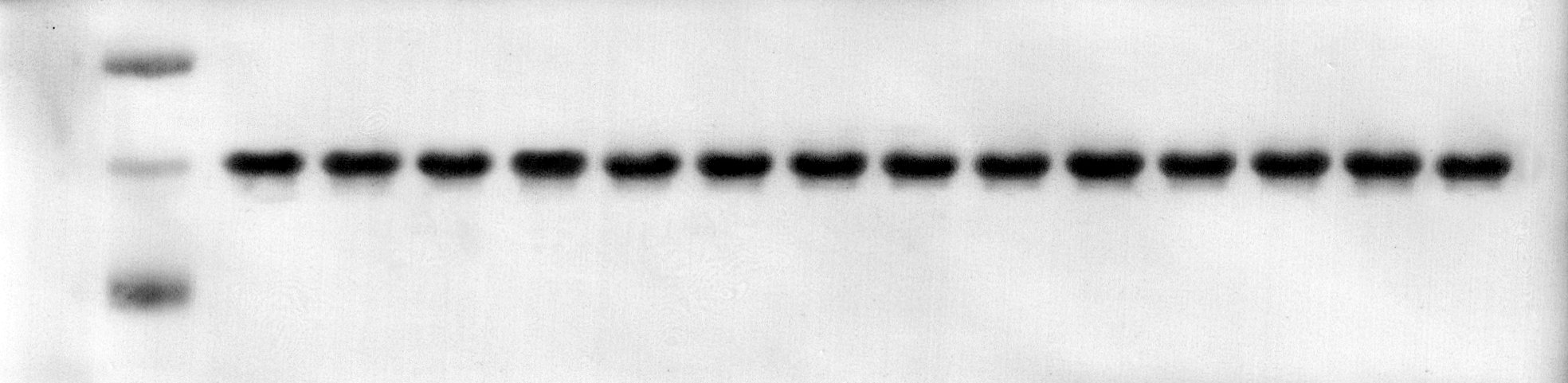

Supplement: Supplementary file 4 [file Presentation_4.ZIP › Figure 14-20210725/Figure 14B-AD-Imaging with the UVP ChemStudio PLUS device/Figure 14B-actin.tif]

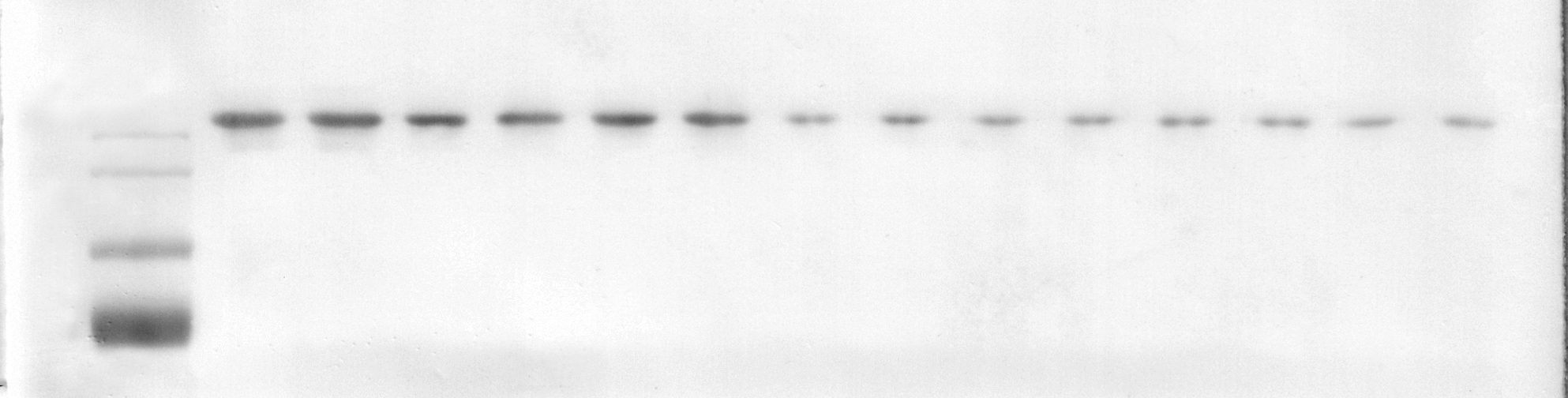

Supplement: Supplementary file 4 [file Presentation_4.ZIP › Figure 14-20210725/Figure 14B-AD-Imaging with the UVP ChemStudio PLUS device/Figure 14B-shank3.tif]
