# Supplementary material for: Expression of the Excitatory Postsynaptic Scaffolding Protein, Shank3, in Human Brain: Effect of Age and Alzheimer’s Disease
Source: Front Aging Neurosci. 2021 Aug 24;13:717263. doi: 10.3389/fnagi.2021.717263 (PMC8421777; doi:10.3389/fnagi.2021.717263)

1. Original WB images for Figure 10D (Imaging with film exposure)

β-actin

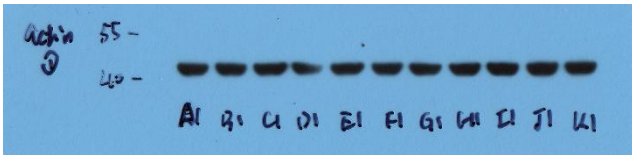

β-actin

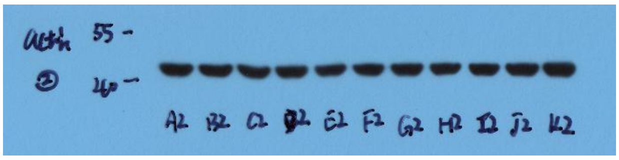

β-actin

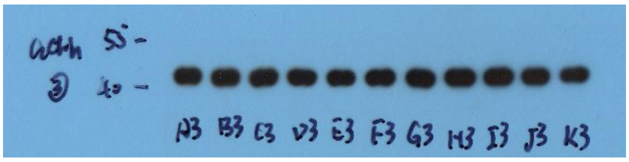

Shank3

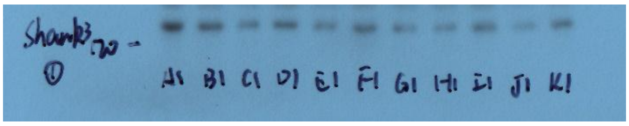

Shank3

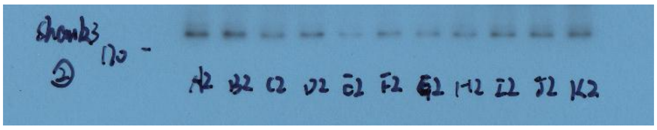

Shank3

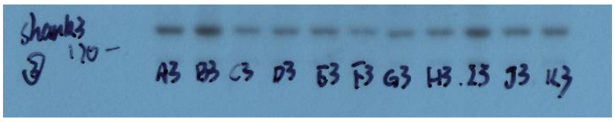

PSD95

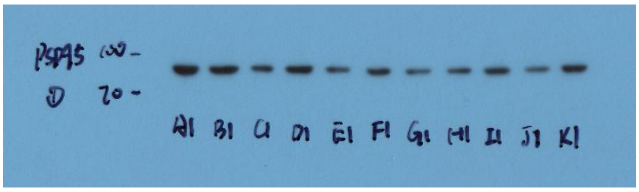

PSD95

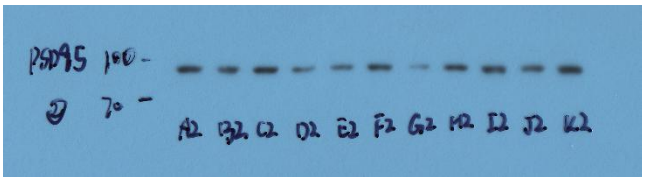

PSD95

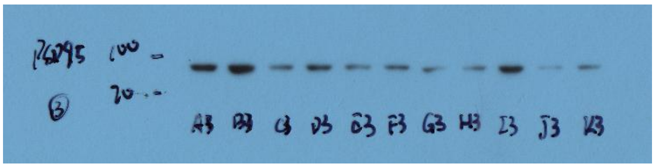

2. Original WB images for Figure 14A (Imaging with film exposure)

Prefrontal ctx

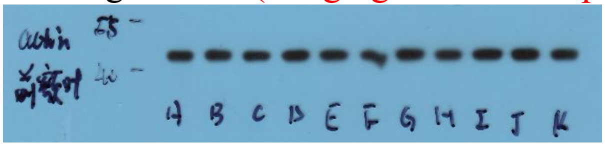

β-actin

Precentral ctx

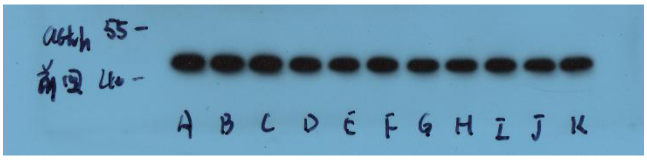

β-actin

Cerebellar ctx

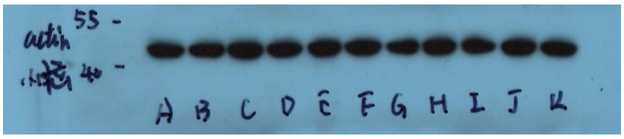

β-actin

Prefrontal ctx

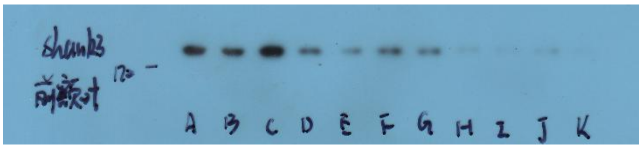

Shank3

Precentral ctx

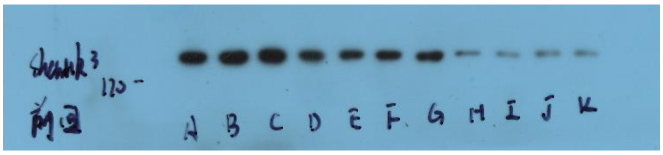

Shank3

Cerebellar ctx

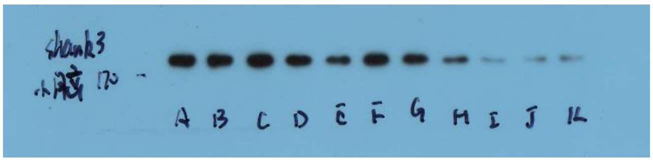

Shank3

Prefrontal ctx

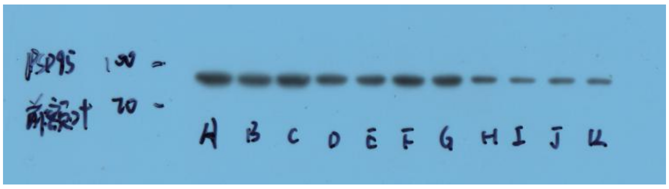

PSD95

Precentral ctx

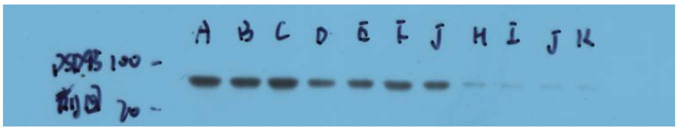

PSD95

Cerebellar ctx

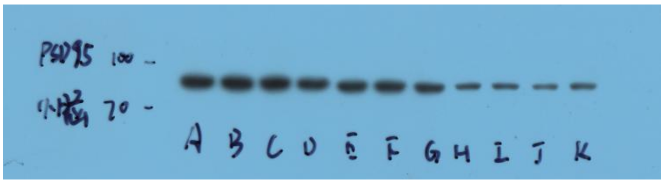

PSD95

Prefrontal ctx

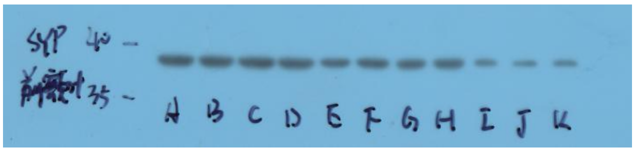

SYP

Precentral ctx

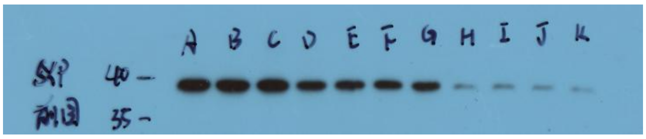

SYP

cerebellar ctx

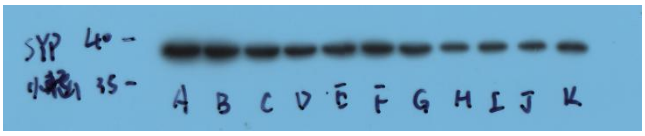

SYP

3. Original WB images for Figure 14B (Imaging with the UVP ChemStudio/ PLUS device)

β-actin

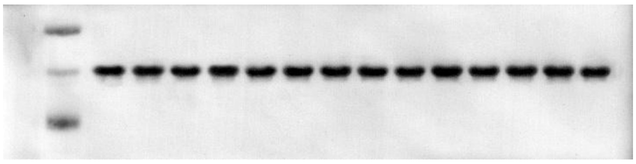

Shank3

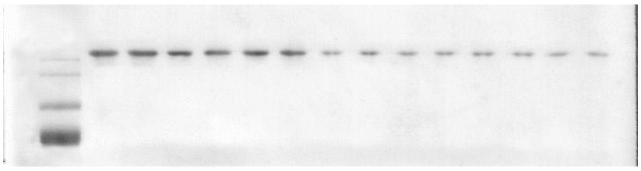

PSD95

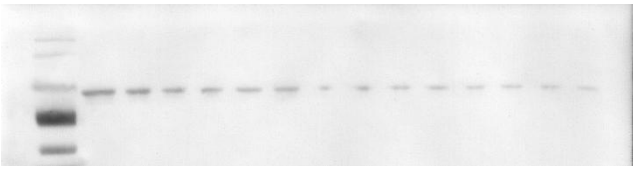

SYP

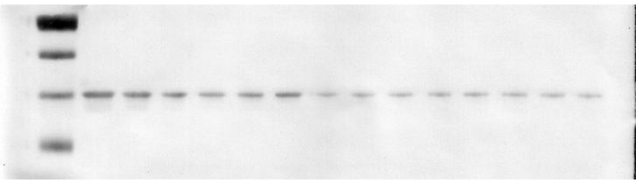

Supplement: Supplementary file 5 [file Presentation_5.PDF]
